# Mass spectrometry-based analyses showing the effects of secretor and blood group status on salivary *N*-glycosylation

**DOI:** 10.1186/s12014-015-9100-y

**Published:** 2015-12-30

**Authors:** Matthew E. Albertolle, Maria E. Hassis, Connie Jen Ng, Severino Cuison, Katherine Williams, Akraporn Prakobphol, Andrew B. Dykstra, Steven C. Hall, Richard K. Niles, H. Ewa Witkowska, Susan J. Fisher

**Affiliations:** Department of Obstetrics, Gynecology, and Reproductive Sciences, University of California San Francisco, San Francisco, CA 94143 USA; Sandler-Moore Mass Spectrometry Core Facility, University of California San Francisco, San Francisco, CA 94143 USA

**Keywords:** Saliva, Secretor, Glycosylation, *N*-glycosite, Lectin, Mass spectrometry

## Abstract

**Background:**

The carbohydrate portions of salivary glycoproteins play important roles, including mediating bacterial and leukocyte adhesion. Salivary glycosylation is complex. Many of its glycoproteins present ABO and Lewis blood group determinants. An individual’s genetic complement and secretor status govern the expression of blood group antigens. We queried the extent to which salivary glycosylation varies according to blood group and secretor status. First, we screened submandibular/sublingual and parotid salivas collected as ductal secretions for reactivity with a panel of 16 lectins. We selected three lectins that reacted with the largest number of glycoproteins and one that recognized uncommon lactosamine-containing structures. Ductal salivas representing a secretor with complex blood group expression and a nonsecretor with a simple pattern were separated by SDS-PAGE. Gel slices were trypsin digested and the glycopeptides were individually separated on each of the four lectins. The bound fractions were de-*N*-glycosylated. LC–MS/MS identified the original glycosylation sites, the peptide sequences, and the parent proteins.

**Results:**

The results revealed novel salivary *N*-glycosites and glycoproteins not previously reported. As compared to the secretor, nonsecretor saliva had higher levels of *N*-glycosylation albeit with simpler structures.

**Conclusions:**

Together, the results suggested a molecular basis for inter-individual variations in salivary protein glycosylation with functional implications for oral health.

**Electronic supplementary material:**

The online version of this article (doi:10.1186/s12014-015-9100-y) contains supplementary material, which is available to authorized users.

## Background

Saliva—the product of the parotid, submandibular, sublingual and minor salivary glands—is a complex biological fluid that exists in two phases. The fluid phase flows over oral surfaces and is swallowed. Salivary components can also be immobilized within the oral cavity, e.g, the pellicle that coats the tooth surface. In both phases, saliva plays many critical roles in general aspects of health (reviewed in [1, 2]). Examples include regulation of the oral microbiome by modulating the adherence, growth and/or viability of a diverse array of organisms. Other important functions include promoting tissue homeostasis, taste and lubrication. The critical roles of this body fluid are illustrated by the myriad pathologies that accompany xerostomia (abnormally low salivary flow), which include dental caries and an increased susceptibility to oral infections, e.g., candidiasis.

Saliva has inorganic as well as protein, glycoprotein, peptide and carbohydrate constituents. During the past decade, several compilations of the salivary proteome have been published. In total, more than 3000 proteins/peptides have been identified (reviewed in Amado et al. [3]) with ductal saliva having about 400 major constituents [4]. Specific salivary functions are parsed among these components. In addition to its well-known enzymatic activity, fluid phase salivary amylase binds to streptococci mediating their clearance from the oral cavity [5]. Saliva also contains a rich repertoire of peptides—members of the histatin, statherin, proline rich protein, and cystatin families—that regulate bacterial adhesion, have microbicidal activity and modulate calcium phosphate chemistry [6, 7]. In general, the role of the carbohydrate motifs in body fluids is much less well understood.

Salivary glycoproteins play a particularly important role in oral health because their carbohydrate constituents interact with a wide array of bacteria. Interestingly, their glycan repertoire is, in part, genetically determined as these structures include the carbohydrate epitopes that comprise the ABO, Lewis (Le) and other blood group determinants. Secretor status further complicates glycosylation patterns. Non-secretors have an inactive form of fucosyltranferase 2 (FUT2), which provides the glycan scaffold for Le^b/y^ and blood-type motifs [8]. Thus, individuals who lack this transferase should have less fucosylated glycoforms than secretors who have an active FUT2. What are the biological consequences? Many of these glycans serve as receptors that mediate adhesion of bacteria that colonize the oral cavity—e.g., the T-Ag for *Actinomyces naeslundii*; the sT-Ag for several streptococcal strains [9]; and the Le determinants, sLe^a^, Le^b^, and Le^x^, for *H. pylori* strains [10, 11]. The major glycan carried by the glycosylated proline rich protein (gPRP) is a biantennary oligosaccharide with a difucosylated lactosamine (Le^y^) sequence on one antenna and an unsubstituted lactosamine sequence on the other. *Fusobacterium nucleatum*, a microorganism associated with periodontal disease, binds to the gPRP via terminal unmodified lactosamine sequences; fucosylation of this disaccharide blocks bacterial binding [12].

Here we asked, at a global level, whether secretor status affects the arrangement of carbohydrate chains along the amino acid backbone of salivary proteins. To answer this question we used a modification of a workflow that our group devised [13]. First, we identified the lectins (carbohydrate binding proteins) that best discriminated between salivary samples from secretors vs. nonsecretors. To do so, we screened parotid and submandibular/sublingual (SMSL) salivas collected as the ductal secretions from the two groups with a large panel of lectins that recognized various aspects of glycan structure. To compare the *N*-glycosylation sites of secretors and nonsecretors, we used columns formed from the lectins with the highest discriminating power or unusual specificities to fractionate electrophoretically separated and trypsin digested saliva samples from the two donor groups. After elution of the bound glycopeptides, the samples were treated with peptide-*N*-glycosidase F (PNGase F), an amidase, and the original *N*-glycosylation sites (NXS/T; X $\ne$ proline) were identified by ESI HPLC–MS/MS via a +1 Da mass shift at the modified Asn. Previously reported and novel *N*-glycosites were identified. Incidentally, we also detected genetic polymorphisms along the peptide backbones some of which were novel. The impact of secretor status on *N*-oligosacharide composition was demonstrated by MALDI MS analyses of the released glycans, which showed higher degrees of fucosylation among the secretor oligosaccharides. Interestingly, more sites were identified when the saliva sample was from a nonsecretor. However, studies on larger number of individuals are needed to further investigate the potential impact of a secretor status on *N*-glycosite occupancy.

## Results and discussion

### Lectin selection

**Fig. 1:**
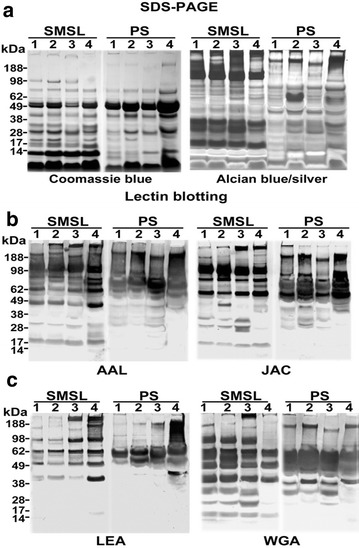
Selection of lectins for capturing human salivary *N*-glycosites. **a** The electrophoretic banding patterns of submandibular/sublingual (SMSL) and parotid (PS) salivary proteins and glycoproteins as visualized by Coomassie blue or Alcian blue silver staining, respectively. Replicate blots were screened against a panel of 16 lectins (Additional file 1: Figure S1), 4 of which are shown in this figure (**b**, **c**). The patterns of reactive bands for three lectins, which in the aggregate, reacted with salivary components over a broad molecular weight range (AAL, JAC and WGA) are shown. LEA was selected due to its reactivity with extended lactosamine units, potential sites of terminal saccharide modifications related to blood group status

As a first step, we screened SMSL and parotid saliva samples collected as the ductal secretions from twenty individuals for reactivity with a 16-lectin panel (Additional file 1: Figure S1). The donors were chosen as representing the spectrum of glycosylation commonly observed in the general US population with regard to the addition of carbohydrate blood group determinants to the core oligosaccharide structures of glycoproteins. We also evaluated relative expression of L-selectin carbohydrate ligands, e.g., MECA-79 reactivity, which are added to salivary components [14]. From the original twenty individuals, we selected four with the following characteristics: donor (1) secretor with blood type O, Le^a+^, Le^b+^, Le^y+^, low MECA-79; donor (2) secretor with blood type B, Le^a+^, Le^b+^, Le^y+^, low MECA-79; donor (3) secretor with blood type O, Le^a+^, Le^b+^, Le^y+^, low MECA-79; and donor (4) nonsecretor with blood type A, Le^a–^, Le^b–^, Le^y–^, high MECA-79. The 16 lectins were chosen based on their carbohydrate specificity, which spanned a wide spectrum from elements that are commonly found in the majority of *N*-linked structures to unusual sugar sequences and/or linkages (Table 1). The results are shown in Fig. 1. Panel A shows the electrophoretic banding patterns of SMSL and parotid, proteins (Coomassie blue staining) and glycoproteins (Alcian blue silver staining), respectively. Of the lectins that were screened, AAL, jacalin (JAC) and wheat germ agglutinin (WGA) reacted with the largest number of bands spanning the greatest molecular weight range (compare Fig. 1b with Additional file 1: Figure S1). Thus, using the rationale that they would capture the highest number of *N*-linked glycopeptides, they were chosen for the separation experiments. In this regard, we also included the *Lycopersicon esculentum* agglutinin (LEA) lectin, which has a much narrower specificity consistent with the banding pattern shown in Fig. 1c, but includes lactosamine units that could be substrates for the addition of the Le antigens.

**Table 1:** Lectins and their carbohydrate binding specificities used to screen SMSL and parotid saliva

Name	Carbohydrate sequence(s)	References
*Artocarpus integrifolia* (Jacalin)	Galβ1-3GalNAc, Galα1-6Gal	[62]
*Erythrina christagalli* (ECA)	Galβ1-4GlcNAc	[63]
*Arachis hypogaea* (peanut, PNA)	Galβ1-3GalNAc	[64]
*Glycine max* (soybean, SBA)	GalNAcα1-3GalNAc GalNAcα/β1-3/4Gal	[65]
*Vicia villosa* (VVA)	GalNAc-Ser	[66]
*Datura stramonium* (DSA)	(GlcNAcβ1-4)2-4, Galβ1-4GlcNAc	[67]
*Lycopersicon esculentum* (tomato, LEA)	(GlcNAcβ1-4)1-4	[68]
*Triticum vulgare* (wheat germ, WGA)	(GlcNAcβ1-4)2-5, Neu5Ac	[69, 70]
	Manβ1-4GlcNAcβ1-4GlacNAc	[71]
*Canavalia ensiformis* (Con A)	Branched *N*-linked hexa-saccharide	[72]
*Galanthus nivalis* (GNA)	Manα1-3Man	[73]
*Lens culinaris* (LCA)	Fucα1-6GlcNAc-N-Asn containing *N*-linked oligosaccharides	[72]
*Aleuria aurantia* (AAL)	Fucα1-6/3GlcNAc	[74]
*Lotus tetragonolobus* (LTA)	Fucα1-2Galβ1-4(Fucα1-3)GlcNAc	[75]
*Sambucus nigra* (SNA)	Neu5Acα2-6Gal/GalNAc	[76]
*Evonymus europaeus* (EEA)	Galα1-3(Fucα1-2)Galβ1-3/4GlcNAc	[77]
*Phaseolus vulgaris erythroagglutinating* (PHA-E)	*N*-linked bi-antennary	[78]

### Piloting the workflow

**Fig. 2:**
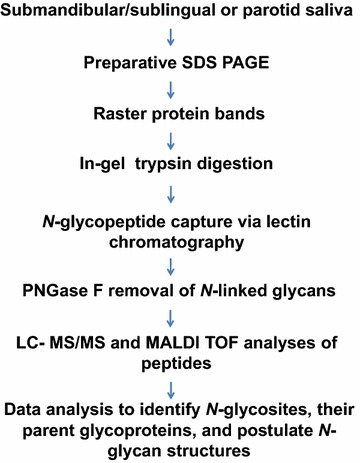
Experimental workflow used to identify salivary *N*-glycosites. Parotid and SMSL salivas collected as the ductal secretions were separated by preparative SDS-PAGE followed by rastering of the gel bands and in-gel trypsin digestion. *N*-glycopeptides were captured from the peptide extracts via their ability to bind to at least one of four lectins (AAL, JAC, WGA and/or LEA). After elution and PNGase F removal of *N*-linked glycans, LC–MS/MS was performed to identify the original sites of oligosaccharide attachments and the protein scaffolds

An overview of the general method that we devised is shown in Fig. 2. SMSL or parotid salivas were separated by preparative SDS-PAGE. The entire gel was horizontally rastered, macerated and subjected to trypsin digestion. The resulting mixture of peptides and glycopeptides was separated on an immobilized lectin column. The bound glycopeptides were eluted and treated with PNGase F, removing the *N*-linked oligosaccharides and converting the asparagines to aspartic acids. LC–MS/MS of the digest enabled sequencing of the peptides and identification of the original sites of carbohydrate attachment.

**Fig. 3:**
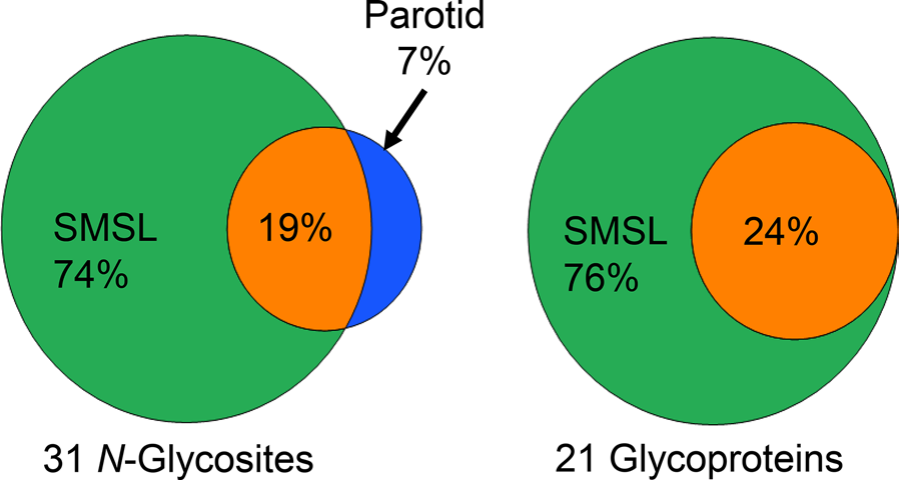
Distribution of *N*-glycosites and glycoproteins between parotid and SMSL salivas in the pilot experiment. The results are derived from analysis of a single secretor (blood type O, Le^a−^, Le^b−^, Le^y−^, and MECA-79 low). SMSL saliva yielded the majority of identifications. Many fewer were found in parotid saliva or both secretions

Prior to scaling up the analysis, we performed a pilot experiment to test the proposed workflow by analyzing 100 µL of parotid and the same amount of SMSL saliva from one donor (secretor, blood type O, Le^a-^, Le^b−^, Le^y−^, and MECA-79 low). The electrophoretically separated samples were rastered into 12 gel slices and chromatographed on immobilized AAL. LC–MS/MS analyses, a total of 24 runs, identified 31 *N*-glycosites from 21 glycoproteins (Fig. 3). To our knowledge, 4 were not previously reported in saliva: IGHG2_HUMAN@176 (Ig gamma-2 chain C region), PRB3_HUMAN@66 (Basic salivary proline-rich protein 3), PSG1_HUMAN@104 and @111 (Pregnancy-specific beta-1-glycoprotein 1), the latter two within the same tryptic peptide. The PSG1 *N*-glycosites sites were recently reported in the *N*-glycoproteome of human metastatic hepatocellular carcinoma cell lines [15]. At the glycoprotein level, IGHG2_HUMAN and PRB3_HUMAN were known salivary components [16, 17]; the detection of PSG1_Human in this body fluid was novel. This was despite the fact that the reproductive age, female donor was not pregnant at the time of sample collection. Nevertheless, this finding raises the interesting possibility that this family of glycoproteins, which are produced in large amounts by the placenta and circulate at high levels in maternal blood, can be detected in saliva during pregnancy. Whether their levels could be indicative of the placental dysfunction that is associated with complications such as preeclampsia is an interesting question that could be addressed in the future. Finally, we note that future studies are necessary to validate these new glycosites since chemical deamidation of asparagine residues during sample preparation can potentially lead to false positive identification of glycosites [18, 19].

The majority of identified *N*-glycosites and associated glycoproteins were identified in SMSL secretions, with parotid saliva contributing only two unique *N*-glycosites, *N*83 and *N*90, within the same tryptic peptide of PIGR_HUMAN (Polymeric Ig receptor). The undersampling limitations of the data-dependent ‘shotgun’ mode of data acquisition that was utilized did not allow for unequivocal conclusions about the absence of specific *N*-glycosites in a sample [20], a question that would best be answered with targeted analyses. Of note, a recently published library containing selected reaction monitoring assays that were developed to enable antibody-independent MS-based analyses of potential *N*-glycosylation sites in body fluids included a number of peptide targets identified in this study (see Additional file 2: Table S1) [21]. In conclusion, the results of the pilot experiment demonstrated the feasibility of applying, to SMSL and parotid salivas, a workflow combining protein fractionation (via SDS-PAGE), in-gel trypsin digestion, lectin capture of glycopeptides carrying *N*-linked oligosaccharides, and LC–MS/MS identification for profiling glycosylation patterns in saliva.

### The effects of secretor status on *N*-glycosylation patterns

Salivary glycoproteins display a diverse carbohydrate repertoire due, in part, to the addition of blood group determinants to many oligosaccharides. Therefore, we were interested, at a global level, in the effects of this phenomenon on *N*-linked glycosylation patterns. To address this point, we applied the method outlined in Fig. 2 to 500 µL of SMSL or parotid saliva, expanding the rastering of the SDS-PAGE gels to 18 slices and the LC–MS/MS runs to 300, for the analysis of SMSL and parotid samples at both ends of the glycosylation spectrum. All results presented in this section were generated using saliva samples from two donors, a secretor and a nonsecretor.

**Fig. 4:**
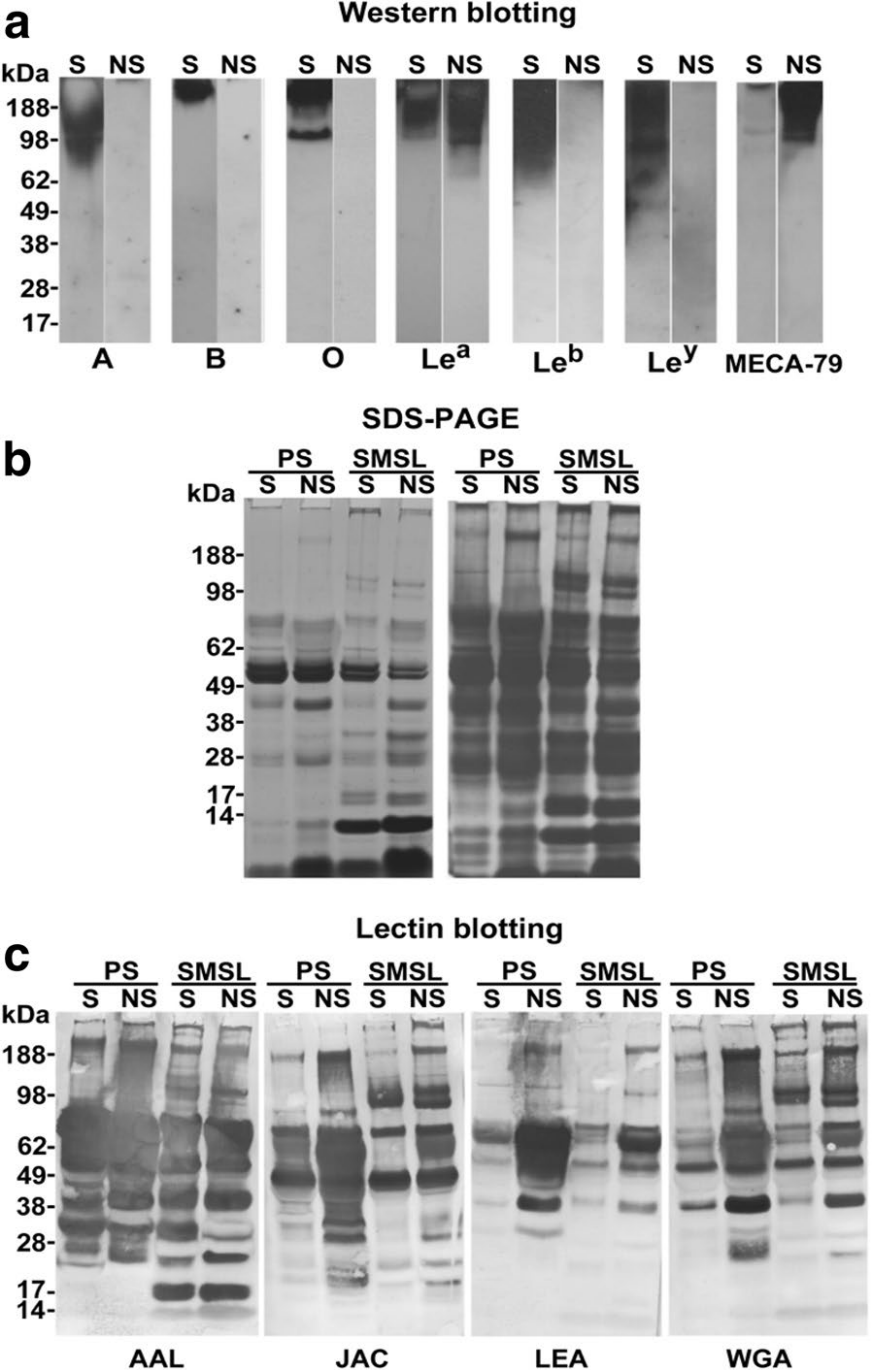
Selection of salivary samples for the *N*-glycosite profiling experiments. Salivas from 20 donors were screened for blood group and selectin ligand (MECA-79) expression. Samples that were representative of secretors (S) and nonsecretors (NS) were chosen. **a** Glycoproteins in the “S” sample reacted with antibodies that were specific for many blood group determinants: A, B, Le^a^, Le^b^, Le^y^. Secretors tended to have low MECA-79 immunoreactivity as was the case with this donor. Glycoprotein expression of blood group determinants in the “NS” sample was limited to Le^a^ (**a**). Nonsecretors tended to have high MECA-79 immunoreactivity as exemplified by this donor. **b** The SDS-PAGE banding patterns of the donor salivas stained with Coomassie blue and Alcian silver (left and right, respectively) were similar to those of the salivas analyzed in the initial screen (see Fig. 1a). **c** Lectin blotting showed different patterns of reactivity among the samples

The first donor was a secretor with blood type AB and complex patterns of terminal fucosylation as determined by broad Le immunoreactivity (anti-Le^a^, anti-Le^b^ and anti-Le^y^) and relatively low levels of the MECA-79 epitope (Fig. 4a). The second donor with the simplest pattern was a nonsecretor with blood type O. Immunoblotting revealed that the salivary secretions displayed Le^a+^, but were Le^b−^ and Le^y−^ with high levels of MECA-79 immunoreactivity (Fig. 4a). An overview of the protein composition of the samples, as determined by SDS-PAGE showed similar patterns to those observed in saliva samples from the other donors (compare Figs. 4b and 1a), which was also true for the lectin blotting results (AAL, jacalin, LEA, and WGA; compare Figs. 4c and 1b). Thus, we glycotyped the donors and showed that, in general terms, their SMSL and parotid samples had the expected repertoire of proteins and glycoproteins.

**Fig. 5:**
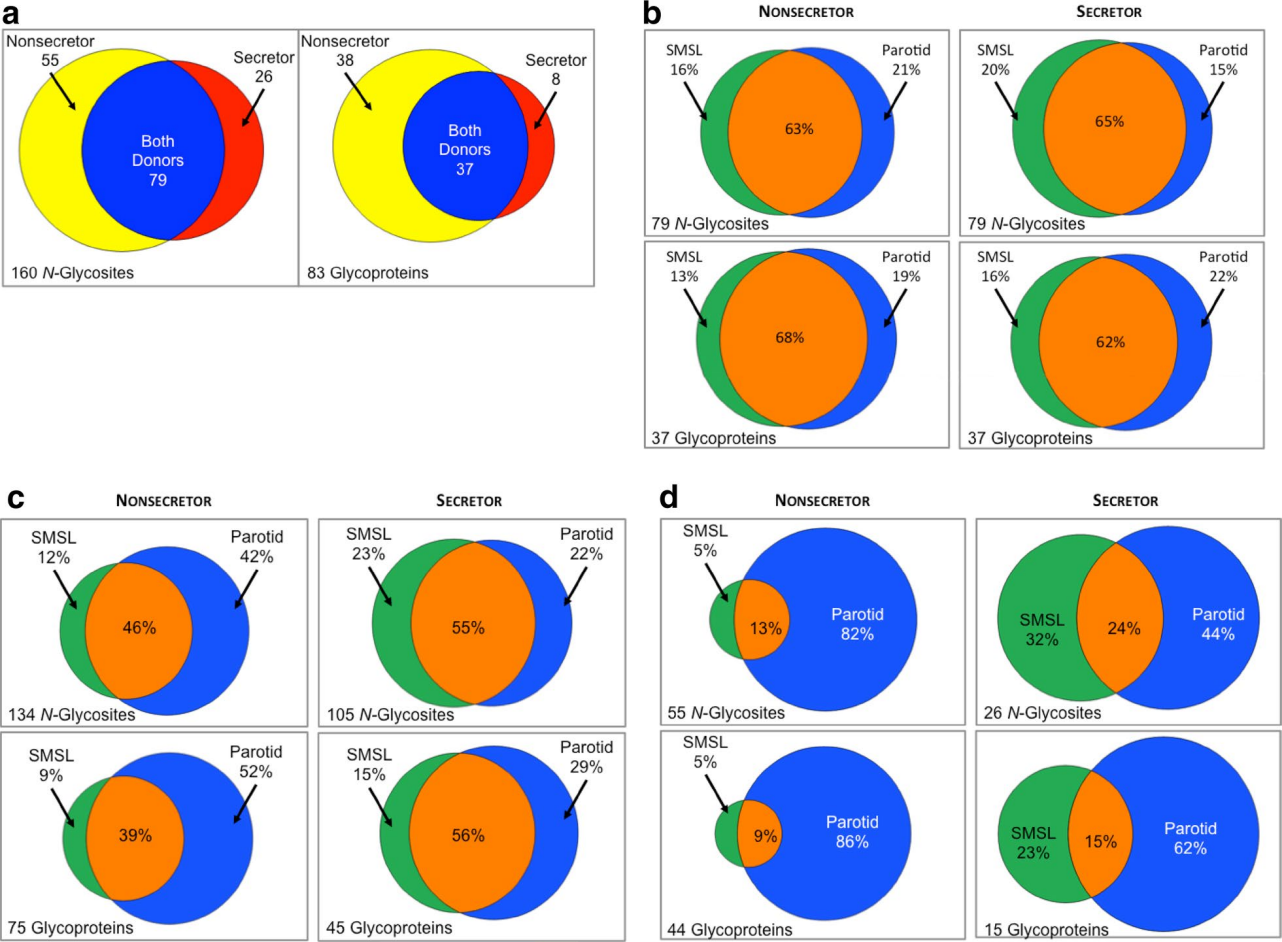
Comparison of *N*-glycosites and glycoproteins by donor and saliva type. Results of analysis of a single secretor and a single nonsecretor are shown. **a** In total, 160 distinct *N*-glycosites within 83 glycoproteins were identified. Of those, 79 *N*-glycosites and 37 glycoproteins were common for both donors vs. species that were unique based on secretor status. **b** Overall, many more *N*-glycosites and glycoproteins were detected in the nonsecretor vs. the secretor sample. The majority of *N*-glycosites observed in the nonsecretor sample were found in parotid saliva while they were more evenly distributed between parotid and SMSL in the secretor sample. Both saliva types shared a large portion of detected *N*-glycosites. These trends were also observed at the glycoprotein level. **c** In the donor-common category, more than 60 % of *N*-glycosites and their parent glycoproteins were observed for both salivas with relatively equal contributions of SMSL and parotid salivas to the remainder. **d** In the donor-unique category, parotid saliva was the major source of *N*-glycosites and glycoproteins in both the nonsecretor and the secretor samples

With regard to an overview of the LC–MS/MS results, Additional file 2: Table S1 lists all peptide identifications and their associated *N*-glycosites, including the sites identified exclusively in the pilot experiment. The spectra are shown in Additional file 3: Figure S2. Overall, 2296 distinct peptides (54,298 spectral counts) were identified in the SMSL and parotid salivas of the nonsecretor vs. 1928 distinct peptides (43,197 spectral counts) in salivas from the secretor. Using our established criteria (see “Methods”), a total of 160 distinct *N*-glycosites within 83 glycoproteins were identified. First, we assessed the results in terms of the *N*-glycosites and their parent glycoproteins that were common for the donors vs. species that were unique based on secretor status. In the common category, 79 *N*-glycosites (Fig. 5a, left) and 37 glycoproteins (Fig. 5a, right) were detected in both donors. More donor-unique *N*-glycosites were identified in the nonsecretor samples (55 from 38 proteins) as compared to those of the secretor (26 from 8 proteins).

We then examined, in detail, the distribution of *N*-glycosites across both donors and saliva types. Out of 160 distinct *N*-glycosites, 134 (4249 spectral counts) were confirmed in the nonsecretor salivas as compared to 105 distinct *N*-glycosites (4562 spectral counts) in the secretor samples (Fig. 5b). In 8 cases, 7 involving both nonsecretor and secretor samples and 1 instance in the analysis of the nonsecretor sample, the two *N*-glycosites resided within a single tryptic peptide. Some *N*-glycosites were observed within peptides of different lengths—due to truncation, proteolytic cleavage or missed cleavage sites—thus increasing the confidence of their assignments (examples shown in Additional file 2: Table S1, Additional file 3: Figure S2).

As shown in Fig. 5b, more *N*-glycosite species (upper panels) were found in the nonsecretor samples (134 total, upper left) than in those of the secretor (105 total, right). This was also true at the level of the glycoproteins from which they were derived (75 vs. 45; bottom panels). However, spectral counting suggested that higher copy numbers of *N*-glycosites were detected in the secretor vs. the nonsecretor sample (Additional file 4: Figure S3A). This was despite the fact that the spectral count distribution of non-glycosylated peptides was virtually identical for both samples types (Additional file 4: Figure S3B). Parotid saliva was a major source of *N*-glycosites in the nonsecretor samples (Fig. 5b, upper left) while a more balanced contribution of SMSL and parotid salivas was observed for the secretor (Fig. 5b, upper right). It is interesting to note that this result was consistent with the lectin binding patterns of the electrophoretically separated samples (Fig. 4c). For both donors, a large fraction of detected *N*-glycosites was common to the two saliva types.

In the donor-common category, more than 60 % of *N*-glycosites and their parent glycoproteins were observed for both salivas with relatively equal contributions of SMSL and parotid salivas to the remainder (Fig. 5c, upper and lower panels). In contrast, parotid saliva was the major source of donor-unique *N*-glycosites and glycoproteins in both the nonsecretor and the secretor samples (Fig. 5d, upper and lower panels).

In a cumulative analysis of the entire data set, the relative abundance of each *N*-glycosite was estimated by the number of spectral counts observed for all deglycosylated peptides encompassing that site. The counts varied significantly from 1 to more than 400 (433 for ZA2G_HUMAN@109 [Zinc-alpha-2-glycoprotein] in the nonsecretor and 445 for AMY1_HUMAN@476 [Amylase] the secretor. The *N*-glycosites were binned into 7 groups based on the number of associated spectral counts (Additional file 5: Figure S4). *N*-glycosites that were common among the donors had similar distributions in nonsecretor (median/average = 15/49) and secretor samples (median/average = 16/59, left Panel). *N*-glycosites that were unique to a particular donor were present in lower abundances (Additional file 5: Figure S4, right Panel).

In addition, by virtue of our experimental design, we also observed protein isoforms and mutations. For example, an N-glycosite unique for isoform 3 of C1q tumor necrosis factor-related protein 3 (C1QT3_HUMAN) was observed in both donors. Interestingly, we failed to detect the canonical structure for the carbonic anhydrase 6 (CAH6_Human) tryptic peptide encompassing the N-glycosite at position 67. Rather, a common mutation, 68 M→L (dbSNP ID: rs2274328), was found in both donor samples (Additional file 3: Figure S2, spectrum 26a). In addition, the nonsecretor carried another mutation at 90S→G (dbSNP ID: rs2274329) that resided with 68 M→L in the same tryptic deglyco-peptide (Additional file 3: Figure S2, spectrum 26b), suggesting compound heterozygosity for these variants. To the best of our knowledge, this double mutant was not previously reported. A number of recent studies addressed the impact of polymorphic CAH6 structures on salivary parameters with the goal of discerning associated phenotypes of potential clinical significance [22–25].

Together, these data pointed to the potential impact of secretor status on utilization of *N*-glycosites. Namely, these simpler glycosylation patterns of the nonsecretor could render the peptide backbone of salivary glycoproteins more accessible to glycosyltransferases, which would have the net effect of increasing the number of unique *N*-glycosylation sites [26]. However, the secretor sample had more oligosaccharides, in absolute terms, which could interact with the lectins we used, e.g., fucose residues that bind to AAL. This finding is consistent with the ability of secretors to add additional fucose resides to the simpler glycan termini typical of nonsecretors. Given that one glycosylation site can carry oligosaccharides with many different structures, the net effect would likely be an increased probability of any given secretor glycopeptide binding to AAL.

### Lectin performance

**Fig. 6:**
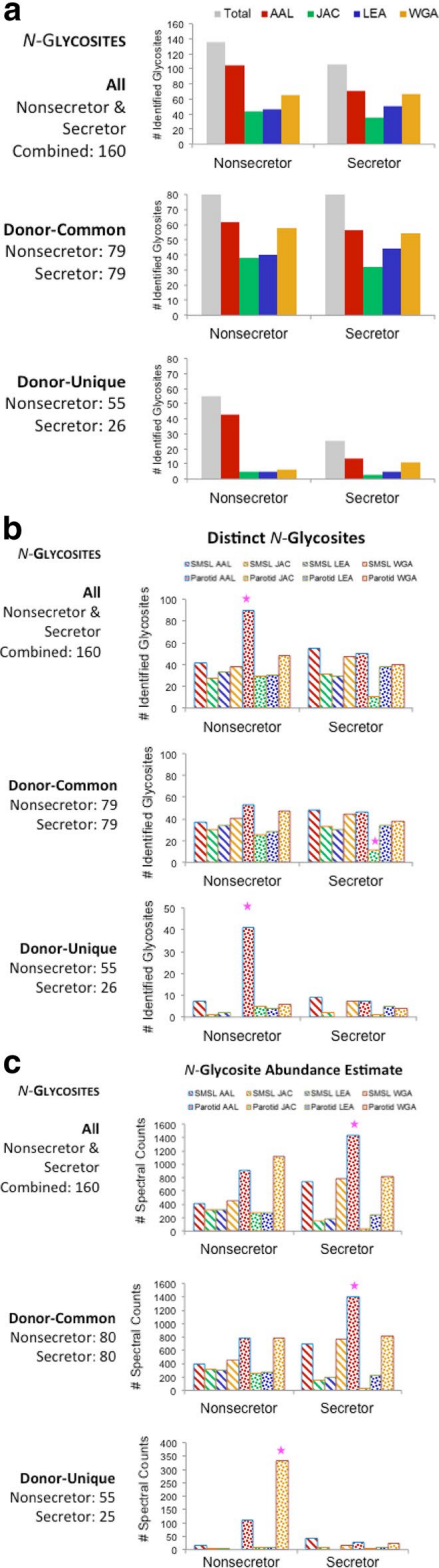
Lectin capture efficiency, in terms of number of *N*-glycosites identified and relative abundance. Results shown are of analysis of a single secretor and a single nonsecretor. **a** (*upper*) Overall, AAL enrichment tended to yield the greatest number of *N*-glycosites. **a** (*middle*) With regard to *N*-glycosites that were common among donors, lectin performance did not depend on secretor status (**a**, *lower*) whereas more *N*-glycosites tended to be captured by AAL from the nonsecretor sample. **b** (*upper*) Overall, AAL demonstrated the greatest capture efficiency, enriching more *N*-glycosites from the parotid saliva sample of the nonsecretor (*starred*). **b** (*middle*) With regard to *N*-glycosites that were common among donors, the low level of jacalin capture of parotid sites from the secretor sample was evident (*starred*). **b** (*lower*) With regard to donor-unique *N*-glycosites, AAL capture from parotid saliva of the nonsecretor was once again most productive in terms of a number of identified *N*-glycosites (*starred*). **c** (*upper*) As to relative abundances in terms of spectral counts and overall performance, WGA and AAL capture tended to have the highest efficiently. **c** (*middle*) In terms of donor-common species, the highest number of *N*-glycosites tended to be found in the AAL bound fraction of the secretor parotid saliva sample. **c** (*lower*) In terms of donor-unique sites, WGA captured the highest numbers from the nonsecretor parotid saliva sample (*starred*)

Parotid and SMSL salivas from a single secretor and a single nonsecretor of the glycotypes described above were studied in this series of experiments. Figure 6a illustrates the efficiency of the lectins employed in terms of the number of *N*-glycosites identified summed for SMSL and parotid salivas. None of the results achieved statistical significance in terms of being greater than 2 SD above or below the mean values. However, trends were observed. With regard to the total numbers, AAL enrichment tended to lead to the highest number of identifications (top panels). There was no difference in lectin performance in terms of *N*-glycosites that were common among donors (middle panel). A trend was observed in which a higher number of donor-unique *N*-glycosites tended to be captured by AAL from the nonsecretor sample (lower panel). Next, we explored these results in terms of each saliva type (Fig. 6b). Overall, AAL was the only lectin that outperformed the others, capturing significantly more *N*-glycosites from the parotid saliva sample of the nonsecretor (upper Panel). With regard to *N*-glycosites that were common among donors, the same trend was observed (middle Panel). However, we also noted the low recovery of *N*-glycosites from the secretor parotid sample following jacalin capture. With regard to donor-unique sites, AAL capture from the parotid sample of the nonsecretor once again led to the highest number of unique identifications (lower panel). AAL separation also led to the greatest number of unique *N*-glycosite identifications per sample-lectin combination (Additional file 6: Figure S5; left Panel). However, it was the secretor-SMSL-jacalin combination that resulted in the highest fraction of recovered unique glycopeptides (Additional file 6: Figure S5; right Panel).

Finally, we used relative abundance (e.g., spectral counts) to compare lectin performance (Fig. 6c). Overall, AAL tended to capture the highest number of *N*-glycosites from the parotid sample of the donor who was a secretor; WGA followed by AAL had the highest efficiently in terms of enrichment from parotid saliva of the nonsecretor (upper panel). In terms of donor-common species (middle panel), the highest number of *N*-glycosites was observed in the AAL bound fraction of the secretor parotid saliva sample. Finally, in terms of donor-unique sites, WGA captured the highest numbers from the nonsecretor parotid saliva sample (lower panel). Of note, the latter result was largely driven by the preferential isolation of FIBB_HUMAN@394.

The overall performance of AAL was in keeping with the fact that this lectin binds fucose-containing oligosaccharides such as the Le determinants that differed among our donors. In contrast, *N*-glycosites that were common among donors were more likely to have broader lectin affinity perhaps due to their less specialized underlying carbohydrate structures. The poor performance of jacalin was in accord with its *N*-acetylgalactosamine specificity, a sugar that is typically a component of *O*-linked oligosaccharides, which this study did not interrogate.

Together these results suggested that the choice of lectins for separating saliva samples depends on the type of information that is being sought. If maximizing the number of unique identifications is the goal, then AAL capture is the best discriminator of *N*-glycosites as applied to parotid saliva samples from nonsecretors vs. secretors. If measures of relative abundance are of interest, then WGA ± AAL should be used for enrichment.

### Differences in *N*-glycosite occupancy between the donors

**Fig. 7:**
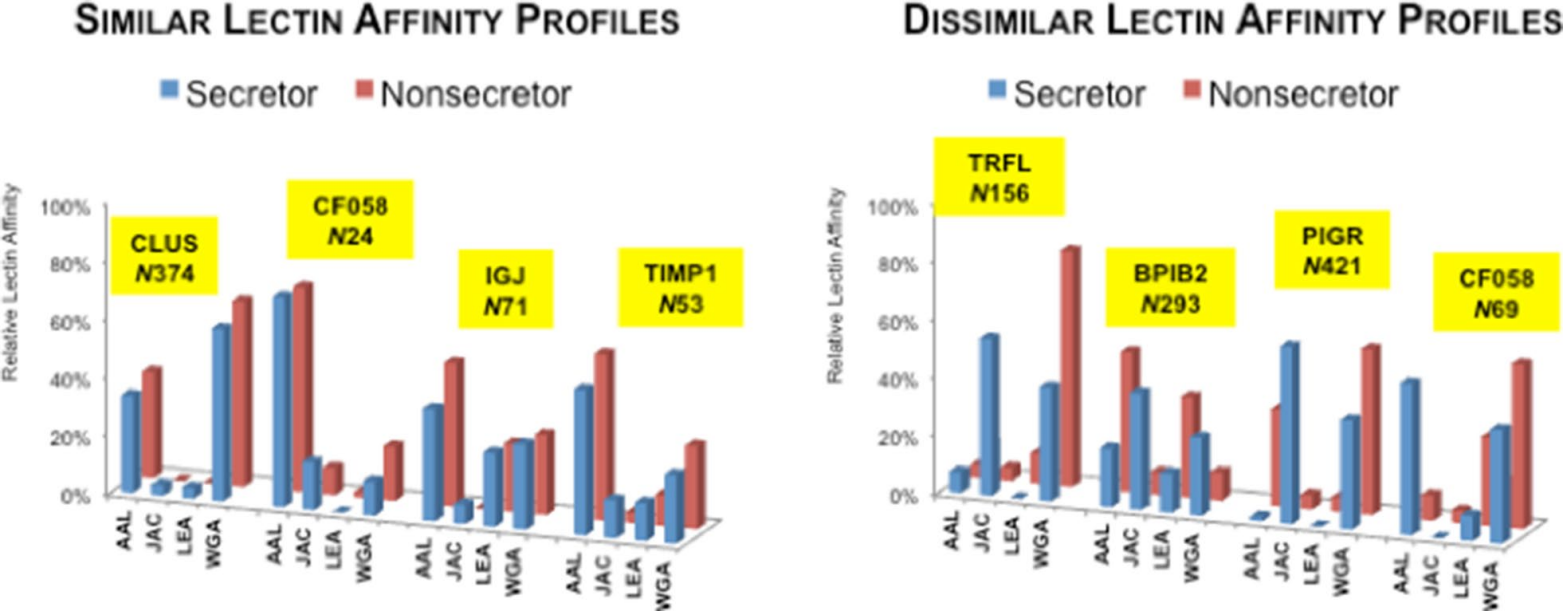
Comparison of the relative lectin capture efficiency for *N*-glycosites that were detected in the secretor and nonsecretor samples across the lectins employed. Spectral counts of glycopeptides encompassing an *N*-glycosite captured by all four lectins were summed and a percentage contribution of each lectin to this pool was calculated to represent the relative capture efficiency of a specific lectin for a given *N*-glycosite. (*Left panel*) Examples of four *N*-glycosites that showed similar lectin binding profiles regardless of secretor status. (*Right panel)* Examples of four *N*-glycosites that demonstrated disparate lectin binding profiles according to secretor status

For this analysis, we compared one secretor and one nonsecretor and considered only common sites with ≥30 counts per donor. Of the 45 *N*-glycosites that met these criteria, most were highly correlated between the nonsecretor and the secretor samples (r > 0.69 for 60 % and 0.97 for 26.7 %). Figure 7 (left Panel) shows four examples according to their lectin binding profiles. To the right are four examples of *N*-glycosites that varied in their lectin enrichment levels according to secretor status. In addition to differences in individual *N*-glycosite lectin interactions according to secretor status, this analysis demonstrated interesting differences in glycosylation between the sites along the same peptide backbone, e.g., CF058_HUMAN (N24 vs. N69; r = 0.99 and 0.24, respectively).

Other examples included sites that were unique among donors (not shown). These included FIBB_HUMAN@394 (Fibrinogen beta chain), which was observed with 304 counts only in parotid saliva of the non-secretor. Likewise, FIBG_HUMAN@78 (Fibrinogen gamma chain) was unique to parotid saliva of the nonsecretor, albeit at a much lower relative abundance (18 spectral counts). Both peptides primarily interacted with WGA. The other relatively abundant *N*-glycosites that were specific to the nonsecretor samples included DMBT1_HUMAN@1889 (Deleted in malignant brain tumors 1 protein), RNT2_HUMAN@212 (Ribonuclease T2) and LG3BP_HUMAN@125 (Galectin-3-binding protein). Of the 25 secretor-unique *N*-glycosites, 8 resided in FCGBP_HUMAN (IgGFc-binding protein) detected in the AAL and/or WGA-retained fractions of SMSL.

Thus, the results of these experiments show that secretor status might play an important role in determining glycosylation patterns along the peptide backbone. The numerous examples of this phenomenon that we observed suggested that there could be interesting and important biological consequences. Examples include the display of carbohydrate receptors that are recognized by bacterial adhesins, which could play a role in specifying the oral ecology. In this context, it is interesting to note that non-secretors are predisposed to chronic periodontitis [27] and Sjögren’s syndrome [28]. Perhaps these “hot spots” of variable glycosylation could be used as diagnostics to predict oral health. Additionally, secretor status correlates with susceptibility to or protection from numerous systemic conditions, including those with an autoimmune etiology such as Crohn’s disease (e.g., [29]). Thus, the information that can be gained from analyzing salivary expression of blood group antigens could have translational potential in terms of the clinical practice of medicine and/or dentistry.

### Oligosaccharide profiling, nonsecretor vs. secretor salivas

**Fig. 8:**
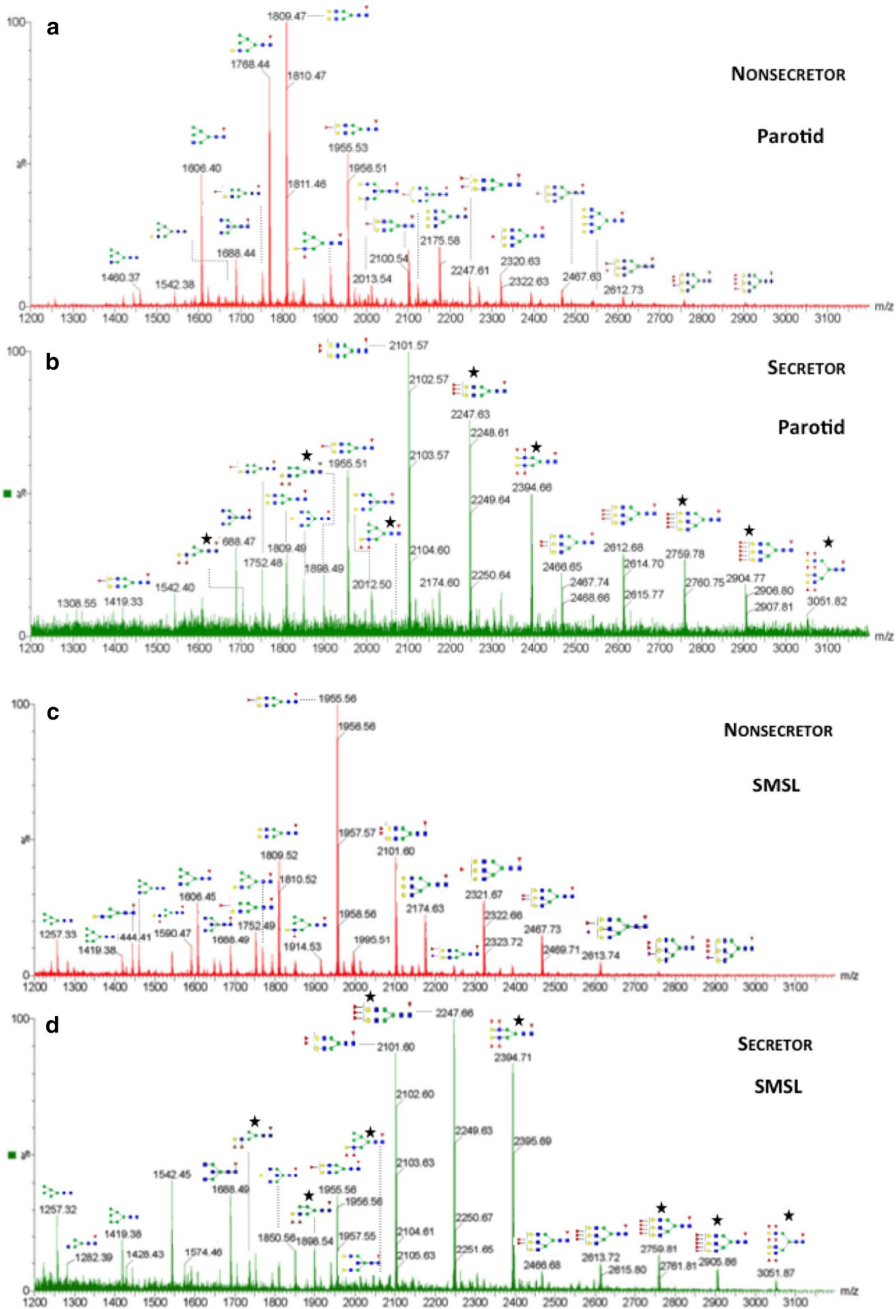
Glycan profiles of nonsecretors vs. secretors for parotid (**a** and **b**) and SMSL (**c** and **d**) salivas. *N*-linked oligosaccharides were released by PNGase F digestion and analyzed by MALDI MS. Putative structures of several glycans that were detected are shown. Overall, both salivas contained high mannose and hybrid bi- and tri-antennary structures. Higher levels of fucosylation, consistent with the presence of blood group H-antigen structure (marked with *black stars*), were observed in the secretor samples. SMSL glycans had a higher level of fucosylation as compared to those observed in parotid saliva. Monoisotopic masses of detected glycans and their structure assignments are listed in Additional file 7: Table S2. Monosaccharides are indicated using symbols defined by the Consortium for Functional Glycomics [61]

Samples from a single secretor and a single nonsecretor were analyzed. Based on donor blood group status, we theorized that the glycan profiles of the nonsecretor samples were characterized by saccharides with fewer fucosylated species. MS-based analyses confirmed this result. The parotid profiles are shown in Fig. 8a, b (nonsecretor and secretor, respectively); the SMSL profiles are shown in Fig. 8c, d (nonsecretor and secretor, respectively). Parotid saliva contained high mannose as well as hybrid, bi- and tri-antennary structures with higher levels of fucosylation in the secretor as compared to the nonsecretor sample (marked with black stars). In general, the structures we observed (Additional file 7: Table S2) were highly correlated with the core fucosylated oligosaccharides that were described by Guile et al. in their analysis of the human parotid gland glycome [30]. The same general pattern was observed in the SMSL sample with even higher levels of fucosylation. Thus, our structural analyses confirmed that, in saliva, the blood group expression patterns are borne out at the oligosaccharide structural level. We note that other groups have reported differential glycan complexity of secretors compared to nonsecretors [31, 32]. Thomsson et al. detected ABO(H) blood group specific structures that decorated *O*-glycans of salivary MUC5B obtained from nonsecretors demonstrating a higher degree of sialylation compared to secretors [31]. Similarly, an absence of glycan masses corresponding to H-antigen structures in saliva collected from a nonsecretor blood group A individual was observed by Everest-Dass et al. [32].

Glycosylation is a post-translational modification that affects up to 50 % of secreted and cellular proteins [33]. Oligosaccharide addition adds structural diversity to proteins, and a broad range of glycan functions have been reported including roles in innate immunity [34, 35] and cancer [36]. There have been many studies of the salivary proteome [4, 37], the salivary glycome [32, 38, 39] and glycoproteome [16, 35, 40–45]. However, the majority have not taken secretor status and blood group antigen expression into account. Given the general lack of information about the impact of these variables on inter-individual variations in protein glycosylation, this is a very fruitful future direction for further work focused on saliva and other body fluids.

## Conclusions

We employed a workflow that combined SDS-PAGE protein fractionation, in-gel tryptic digestion, lectin affinity capture of *N*-linked glycopeptides and LC–MS/MS to interrogate, at a global level, human ductal salivary *N*-glycosylation. We considered two variables—blood group (ABO and Lewis) and secretor status. As expected, saliva from a nonsecretor (as compared to a secretor) carried lower level of fucosylation, as confirmed by mass spectrometry analyses of the released *N*-glycans. Intriguingly, a significantly higher number of occupied *N*-glycosites was found in a nonsecretor. Thus, the results of this analysis suggested that blood group and secretor status are important sources of inter-individual variations in salivary glycosylation. The consequences likely include significant effects on the oral ecology.

## Methods

Unless otherwise noted, all reagents were purchased from Thermo-Fisher.

### Sample collection

The protocols for collecting human saliva were approved by the University of California, San Francisco Committee on Human Research and written informed consent was obtained from all participants. Briefly, the donors thoroughly rinsed their mouths with water prior to sample collection, which was done at the same time in all cases (early afternoon) to mitigate the possible confounding effects of diurnal variation on salivary composition [46]. Salivary flow was stimulated by the application of citric acid to the tongue. Parotid secretions were obtained by using a Lashley cup [12, 47]. SMSL saliva was obtained by using a Block and Brotman collector that fit around the gland openings [4, 48]. The secretions were collected on ice. Immediately thereafter, a proteinase inhibitor cocktail (Pierce) was added. Then the samples were briefly vortexed, divided into 1 mL aliquots and frozen at −80 °C.

### Analysis of blood group type and secretor status

SMSL and parotid saliva samples from 20 individuals were electrophoretically separated (4–12 % polyacrylamide gels) and transferred to nitrocellulose. The blots were probed with antibodies that recognize the A, B, Le^y^ (Abcam), Le^a^, Le^b^ (Neomarkers) blood group antigens and sulfated sialyl Le^x^ (the L-selectin ligand, MECA-79, BD Biosciences). The method that we used has been published [49].

### Lectin screening

We used our published method [50]. Each blot consisted of electrophoretically separated samples from 4 individuals. In all, 16 lectins were screened. They are listed in Table I along with the carbohydrate determinants that they recognize.

### Gel fractionation and digestion

Preparative SDS-PAGE was carried out using a Novex 4–12 % mini-gel and an X-Cell electrophoresis apparatus (Life Technologies). Either 80 (pilot experiment) or 500 µLs of saliva was loaded onto the gel and electrophoresis was performed at 100 V for 2.5 h. Protein bands were visualized by staining with SimplyBlue SafeStain Coomassie G-250 (Invitrogen, Life Technologies, Carlsbad, CA, USA). The entire gel was rastered into 12 (pilot experiment) or 18 slices. Each slice was diced into 1–2 mm pieces, transferred to 2.0-mL Eppendorf tubes and in-gel digestion was performed using a modification of the method described by Shevchenko et al. [51]. Specifically, the first supernatant was removed and gel pieces were incubated with 25 mM ammonium bicarbonate (ABC)/50 % acetonitrile (ACN) to extract peptides. The second supernatant was removed and combined with the first followed by concentration to 25–50 µL in a SpeedVac. Then the samples were brought to a volume of 110 µL with Lectin Buffer A (25 mM Tris, pH 7.4, 50 mM sodium chloride, 10 mM calcium chloride, and 10 mM magnesium chloride; Sigma Aldrich, St. Louis, MO, USA) and passed through 0.2 µm cellulose acetate spin filters (Agilent Technologies, Santa Clara, CA, USA) to remove the remaining gel fragments.

### Lectin column assembly and glycopeptide enrichment

Columns were produced/packed and lectin affinity enrichment was performed as previously described [13]. Briefly, *Artocarpus integrifolia* (jacalin, JAC), wheat germ agglutinin (WGA), *Aleuria aurantia* lectin (AAL), or *Lycopersicon esculentum* agglutinin (LEA) lectins (Vector Labs, Burlingame, CA) were suspended 10–20 mg/mL in PBS. One hundred mg POROS AL 20 µm beads (Applied Biosystems, Foster City, CA, USA) were washed twice with 1 mL PBS. Then the lectin solution was added, followed by NaBH_3_CN to a final concentration of 50 mM and the suspension was shaken overnight at room temperature. The beads were washed with 1 mL Tris–HCl pH 7.4, then incubated for 30 min in the same buffer containing 50 mM NaBH_3_CN to block any remaining aldehyde groups. Five washes with 1 M NaCl were performed. Then the beads were packed into 4.6 × 50-mm PEEK HPLC columns by using PBS pumped with a maximum flow rate that was equal to 2000 psi.

Lectin enrichment was accomplished by using a three-step isocratic separation method. One hundred µL of each trypsin digest was applied to a lectin column. First, the column was equilibrated with Lectin Buffer A at a flow rate of 50 µL/min. Second, the samples were loaded and the column was washed for 9 min. Finally, the bound glycopeptides were eluted from the column by using 0.5 M acetic acid for WGA, LEA, and AAL or 100 mM melibiose for jacalin at a flow rate of 500 µL/min for 5.0 min. Then the column was re-equilibrated with Lectin Buffer A for 6 min at a flow rate of 2.5 mL/min. The bound fraction, collected between 9 and 14.2 min, was desalted by using Oasis HLB solid phase extraction (SPE) cartridges. Briefly, an SPE cartridge was wetted with 3 mL of 80 % ACN/0.1 % formic acid (FA) and equilibrated with 3 mL of aqueous 0.1 % FA. Then the sample was loaded and the column washed with 3 mL 0.1 % FA. Next, the peptides were eluted with 1.8 mL 80 % ACN/0.1 % FA into 2-mL eppendorf tubes, neutralized with 200 µL 1 M ABC and vacuum centrifuged to a volume of ~100 µL.

### PNGase F deglycosylation

The eluted *N*-linked glycopeptides were deglycosylated by overnight incubation with 1000 Units of PNGase F (Glycerol-Free, New England Biolabs) as described previously [13]. Then the samples were desalted by using MicroSpin Columns, 5–200 µL loading volume (The Nest Group, Inc.; Southborough, MA, USA). Samples were centrifugally concentrated to dryness and resuspended in 40 µL 0.1 % FA.

### Peptide mass spectrometry

Four microliters from each sample of the original gel bands were separated by using a nanoLC ULtra 2D Plus system (Eksigent/AB Sciex, Foster City, CA, USA) interfaced with a LTQ Orbitrap Velos mass spectrometer (ThermoFisher Scientific). The HPLC was interfaced to the mass spectrometer using a Captive Spray tapered 20 µm I.D. tip (Michrom, Auburn, CA) and applied voltage of 1.5 kV. The peptides were initially loaded onto a guard column (Acclaim PepMap300 C18 300 µm i.d. ×5 mm, 5 µm particle size, 100 Å pore size; Thermo-Fisher) and washed with the aqueous loading solvent that consisted of 2 % Solvent B (98 % ACN/0.1 % FA) in Solvent A (2 % ACN/0.1 % FA), flow rate 10 µL/min for 10 min. Then the peptides were separated on a C18 Acclaim PepMap100 column (75 μm i.d. × 150 mm, 3 µm particle size, 100 Å pore size; ThermoFisher Scientific) heated at 48 °C with a column oven. Peptides were eluted at a flow rate of 600 nL/min initially with 2–40 % Solvent B for 60 min then 40–90 % B from 60 to 65 min. The mass spectrometer was calibrated using a solution of caffeine, MRFA, and ULtramark 1621 according to the manufacturer’s specifications operating in a data dependent mode. In positive ion mode, MS scans from *m/z* 300–1500 with a full width at half-maximum resolution of 30,000 were acquired in the Orbitrap analyzer. Product ions were generated with a collision cell energy of 35 and an activation Q of 0.25. MS/MS scans of the 6 most abundant ions were acquired in the linear ion trap. A mass exclusion time was applied for 30 s with a repeat count of 2 and repeat duration of 20 s and an exclusion list size of 500.

### Database searching and *N*-glycosite assignment

Mass spectrometry data files were processed individually with integrated peak picking using Mascot (Matrix Science, Boston, MA, USA) version 2.2 and Mascot Daemon version 2.2.2 to generate MGF files in an extract_msn format. Protein identification was accomplished by using the MGF files to search the UniProt Swiss-Prot release 2012_10 with all human isoforms that included 73,982 protein sequences. ProteinPilot (version 4.5, AB Sciex, Foster City, CA, USA) was used to perform the combined database searches with all bands from a single lectin and secretion. The following ProteinPilot data filters were used: carbamidomethylation of cysteines, Orbi/FT MS (1–3 ppm), LTQ MS/MS as instrument type, and thorough search. Peptides selected for *N*-glycosite analyses had a minimum confidence of 95 %. *N*-glycosites were assigned as previously described [13], which included utilizing the motif NXS/T where X $\ne$ proline and asparagine was hydrolyzed to aspartic acid. All MS and MS/MS data were manually curated to generate the final *N*-glycosite list. ProteinProspector tools [52] were used to calculate theoretical monoisotopic masses and isotopic envelope distributions for precursors and *m*/*z* values for product ions [53]. The results of database searches for both donors and secretions were aligned using in house-generated Java-based software and analyzed in Excel. Estimates of relative abundances employed spectral counts [54] using data combined from all gel slices.

### Isolation and enrichment of *N*-linked glycans

Prior to glycan removal, 100 µL of the SMSL or parotid saliva samples was clarified by centrifugation (3000×*g*) at 4 °C for 20 min. according to the procedure described by Everest-Dass et al. [55]. The supernatant was mixed with ice-cold acetone (9:1; v/v) and incubated overnight at −20 °C. The next day, the samples were centrifuged to 13,000×*g* for 10 min. The supernatant was decanted. Then the precipitate was dried for 10 min at room temperature before dissolving in 200 µL of 3 M urea. *N*-glycan release was performed as described by Papac et al. with slight modifications [56]. Briefly, a Millipore 96-well plate with PVDF membranes (0.45 µm) was washed (3× each solvent) with 100 µl of 70 % ethanol, 100 µL of water and 100 µL of 3 M guanidine hydrochloride (Sigma-Aldrich, Inc.). All washes were performed under mild vacuum. Twenty µL of each sample was added per well, mixed with 17 mM DTT and incubated at 37 °C for 1 h. Then the wells were washed 3× with 250 µl of milliQ-water and 50 µL of 25 mM iodoacetamide (Sigma-Aldrich, Inc.) was added to each well. After 30 min incubation at room temperature, the wells were washed again with 250 µL of milliQ-water. Membrane blocking was accomplished by room temperature incubation of each well with 100 µL of 1 % PVP-360 (Sigma Aldrich, Inc.) for 30 min. Finally, the membranes were washed 3× with 250 µL milliQ-water. Then each well was filled with 13 µL milliQ-water, 2 µL of 10× reaction buffer (0.5 M sodium phosphate, pH 7.5 at 25 °C) and 5 µL of PNGase F enzyme. The plate was incubated overnight at 37 °C; empty wells were filled with water to minimize sample evaporation.

For enrichment, graphite carbon-packed tips (TopTip™ Reversed Phase C-18, Glygen) were prepared according to the manufacturer’s protocol. Each tip was washed 3× with 50 µl of 0.05 % TFA in 60 % ACN, followed by 3× washes with 50 µL of 0.05 % TFA in water. Glycan samples from several wells containing the same saliva type were pooled and applied to a single tip, which was washed 3× with 50 µL 0.05 % TFA. Captured glycans were eluted with 20 µL of 0.05 % TFA in 60 % ACN.

### MALDI-TOF analysis

The matrix (20 mg/mL) was prepared by dissolving 2′,4′,6′-trihydroxyacetophenone monohydride (THAP; Sigma Aldrich, Inc.) in 10 mM ammonium citrate/25 % ACN. Samples were mixed with an equal volume of THAP matrix and 1 µL was deposited onto a 96-well stainless steel MALDI plate (Waters). Orthogonal (o) MALDI ion mobility (IM) TOF MS analyses were performed in positive and in negative ion modes on a Synapt G2 HD mass spectrometer equipped with a TriWave™ IM analyzer (Waters) using “sensitivity” settings with a typical resolution of 10,000. Data were analyzed using MassLynx software (Waters). The glycan feature catalog was generated using DriftScope™ software to extract glycan ions and the MaxEnt3 algorithm to deconvolute the data and generate monoisotopic *m*/*z* values. Glycomod software tools [57, 58] were employed to match the experimental monoisotopic masses to potential oligosaccharide compositions using the following parameters: mass tolerance ±100 ppm; Na^+^ (positive mode) or [M−H]^−^ (negative mode); free/PNGase F released *N*-linked oligosaccharides; and monosaccharide residues defined as hexose, *N*-acetylhexosamine, deoxyhexose and sialic acid. Glycoworkbench tools were employed to generate stick figures of putative glycan structures [59, 60].

## Additional files

10.1186/s12014-015-9100-y Representative parotid and SMSL salivary protein reactivity with 16 lectins: [*Artocarpus integrifolia* (JAC), Wheat germ agglutinin (WGA), *Aleuria aurantia* lectin (AAL), *Sambucus nigra* (SNA), *Canavalia ensiformis* (Con A), *Lens culinaris* (LCA), *Phaseolus vulgaris* *erythroagglutinating* (PHA-E), *Galanthus nivalis* (GNA), *Lycopersicon esculentum* agglutinin (Tomato or LEA), *Lotus tetragonolobus* (LTA), *Erythrina christagalli* (ECA), *Datura stramonium* (DSA), *Glycine max* (soybean, SBA), *Arachis hypogaea* (peanut, PNA), *Evonymus europaeus* (EEA), *Vicia villosa* (VVA)]. AAL, JAC, and WGA interacted with the highest number of glycoproteins across a wide molecular weight range. Results are shown for parotid and SMSL saliva from 4 donors.
10.1186/s12014-015-9100-y Protein Identifications and Associated *N*-glycosites.
10.1186/s12014-015-9100-y Annotated MS and MS/MS spectra of deglycosylated *N*-linked glycopeptides identified in parotid and SMSL salivas.
10.1186/s12014-015-9100-y Spectral count distribution for *N*-glyco- and non-glycopeptides in the secretor vs. nonsecretor samples. Spectral counting suggested that higher copy numbers of *N*-glycosites were detected in the secretor vs. the nonsecretor sample (Figure S3A). This was despite the fact that the spectral count distribution of non-glycosylated peptides was virtually identical for both samples types (Figure S3B).
10.1186/s12014-015-9100-y *N*-Glycosite distributions binned according to spectral counts. (Left Panel) Similar distributions of donor-common *N*-glycosites were observed across all bins regardless of secretor status. (Right Panel) Lower abundance *N*-glycosites were unique to a particular donor.
10.1186/s12014-015-9100-y Efficiency of *N*-glycopeptide capture by lectin, saliva type and secretor status. Sites that were unique to a particular sample-lectin combination are shown in red and those that were shared among sample-lectin combinations are depicted in blue. (Left Panel) the number of *N*-glycosites and (Right Panel) the relative abundances in terms of spectral counts. Green stars denote >2 SD from the mean. In terms of unique *N*-glycosites detected, AAL captured the largest number in parotid saliva of the nonsecretor. In terms of relative abundances, JAC captured the most copies of unique *N*-glycosites from SMSL saliva.
10.1186/s12014-015-9100-y PNGase F-Released *N*-Glycans Detected by MALDI Ion Mobility MS.

## Abbreviations

Le: Lewis
FUT2: fucosyltranferase 2
gPRP: glycosylated proline rich protein
SMSL: submandibular/sublingual
PNGase F: peptide-*N*-glycosidase F
NXS/T: N = asparagine, X = any amino acid except proline, S = serine, T = threonine
MS: mass spectrometry
MS/MS: tandem mass spectrometry
ESI HPLC–MS/MS: electrospray ionization high-performance liquid chromatography/tandem mass spectrometry
MALDI MS: matrix-assisted laser desorption ionization mass spectrometry
TOF MS: time of flight mass spectrometry
LC–MS/MS: liquid chromatography-tandem mass spectrometry
Orbi/FT MS: Orbitrap/fourier transform mass spectrometry
LTQ: Linear trap Quadropole
Oasis HLB: hydrophilic-lipophilic-balanced
AAL: *Aleuria aurantia* Lectin
JAC: *Artocarpus integrifolia*
WGA: wheat germ agglutinin
LEA: *Lycopersicon esculentum* agglutinin
SDS-PAGE: sodium dodecyl sulfate polyacrylamide gel electrophoresis
MUC5B: Mucin 5B
ABC: ammonium bicarbonate
ACN: acetonitrile
SPE: solid phase extraction
FA: formic acid
MRFA: L-methionyl-arginyol-phenylalanylalanine acetate H2O
MGF: Mascot generic format
TFA: Trifluoroacetic acid
THAP: 2′,4′,6′-trihydroxyacetophenone monohydride
S: secretor
NS: nonsecretor
DTT: dithiothreitol
o: Orthogonal
SNA: *Sambucus nigra*
Con A: *Canavalia ensiformis*
LCA: *Lens culinaris*
PHA-E: *Phaseolus vulgaris**erythroagglutinating*
GNA: *Galanthus nivalis*
LTA: *Lotus tetragonolobus*
ECA: *Erythrina christagalli*
DSA: *Datura stramonium*
EEA: *Evonymus europaeus*
VVA: *Vicia villosa*

## References

[1] Bosch JA (2014). The use of saliva markers in psychobiology: mechanisms and methods. Monogr Oral Sci.

[2] Helmerhorst EJ, Oppenheim FG (2007). Saliva: a dynamic proteome. J Dent Res.

[3] Amado FM, Ferreira RP, Vitorino R (2013). One decade of salivary proteomics: current approaches and outstanding challenges. Clin Biochem.

[4] Denny P, Hagen FK, Hardt M, Liao L, Yan W, Arellanno M (2008). The proteomes of human parotid and submandibular/sublingual gland salivas collected as the ductal secretions. J Proteome Res.

[5] Scannapieco FA, Torres G, Levine MJ (1993). Salivary alpha-amylase: role in dental plaque and caries formation. Critic Rev Oral Biol Med Off Publ Am Assoc Oral Biol.

[6] de Sousa-Pereira P, Amado F, Abrantes J, Ferreira R, Esteves PJ, Vitorino R (2013). An evolutionary perspective of mammal salivary peptide families: cystatins, histatins, statherin and PRPs. Arch Oral Biol.

[7] Melino S, Santone C, Di Nardo P, Sarkar B (2014). Histatins: salivary peptides with copper(II)- and zinc(II)-binding motifs: perspectives for biomedical applications. FEBS J.

[8] Henry S, Oriol R, Samuelsson B (1995). Lewis histo-blood group system and associated secretory phenotypes. Vox Sang.

[9] Prakobphol A, Tangemann K, Rosen SD, Hoover CI, Leffler H, Fisher SJ (1999). Separate oligosaccharide determinants mediate interactions of the low-molecular-weight salivary mucin with neutrophils and bacteria. Biochemistry.

[10] Walz A, Odenbreit S, Stuhler K, Wattenberg A, Meyer HE, Mahdavi J (2009). Identification of glycoprotein receptors within the human salivary proteome for the lectin-like BabA and SabA adhesins of Helicobacter pylori by fluorescence-based 2-D bacterial overlay. Proteomics.

[11] Prakobphol A, Boren T, Ma W, Zhixiang P, Fisher SJ (2005). Highly glycosylated human salivary molecules present oligosaccharides that mediate adhesion of leukocytes and Helicobacter pylori. Biochemistry.

[12] Gillece-Castro BL, Prakobphol A, Burlingame AL, Leffler H, Fisher SJ (1991). Structure and bacterial receptor activity of a human salivary proline-rich glycoprotein. J Biol Chem.

[13] Drake PM, Schilling B, Niles RK, Braten M, Johansen E, Liu H (2011). A lectin affinity workflow targeting glycosite-specific, cancer-related carbohydrate structures in trypsin-digested human plasma. Anal Biochem.

[14] Prakobphol A, Thomsson KA, Hansson GC, Rosen SD, Singer MS, Phillips NJ (1998). Human low-molecular-weight salivary mucin expresses the sialyl lewisx determinant and has L-selectin ligand activity. Biochemistry.

[15] Li X, Jiang J, Zhao X, Wang J, Han H, Zhao Y (2013). N-glycoproteome analysis of the secretome of human metastatic hepatocellular carcinoma cell lines combining hydrazide chemistry, HILIC enrichment and mass spectrometry. PLoS One.

[16] Gonzalez-Begne M, Lu B, Liao L, Xu T, Bedi G, Melvin JE (2011). Characterization of the human submandibular/sublingual saliva glycoproteome using lectin affinity chromatography coupled to multidimensional protein identification technology. J Proteome Res.

[17] Ferreira JA, Daniel-da-Silva AL, Alves RM, Duarte D, Vieira I, Santos LL (2011). Synthesis and optimization of lectin functionalized nanoprobes for the selective recovery of glycoproteins from human body fluids. Anal Chem.

[18] Palmisano G, Melo-Braga MN, Engholm-Keller K, Parker BL, Larsen MR (2012). Chemical deamidation: a common pitfall in large-scale N-linked glycoproteomic mass spectrometry-based analyses. J Proteome Res.

[19] Hao P, Ren Y, Datta A, Tam JP, Sze SK (2015). Evaluation of the effect of trypsin digestion buffers on artificial deamidation. J Proteome Res.

[20] Tabb DL, Vega-Montoto L, Rudnick PA, Variyath AM, Ham AJ, Bunk DM (2010). Repeatability and reproducibility in proteomic identifications by liquid chromatography-tandem mass spectrometry. J Proteome Res.

[21] Huttenhain R, Surinova S, Ossola R, Sun Z, Campbell D, Cerciello F (2013). N-glycoprotein SRMAtlas: a resource of mass spectrometric assays for N-glycosites enabling consistent and multiplexed protein quantification for clinical applications. Mol Cell Proteomics.

[22] Frasseto F, Parisotto TM, Peres RC, Marques MR, Line SR, Nobre Dos Santos M (2012). Relationship among salivary carbonic anhydrase VI activity and flow rate, biofilm pH and caries in primary dentition. Caries Res.

[23] Koc Ozturk L, Ulucan K, Akyuz S, Furuncuoglu H, Bayer H, Yarat A (2012). The investigation of genetic polymorphisms in the carbonic anhydrase VI gene exon 2 and salivary parameters in type 2 diabetic patients and healthy adults. Mol Biol Report..

[24] Aidar M, Marques R, Valjakka J, Mononen N, Lehtimaki T, Parkkila S (2013). Effect of genetic polymorphisms in CA6 gene on the expression and catalytic activity of human salivary carbonic anhydrase VI. Caries Res.

[25] Melis M, Atzori E, Cabras S, Zonza A, Calo C, Muroni P (2013). The gustin (CA6) gene polymorphism, rs2274333 (A/G), as a mechanistic link between PROP tasting and fungiform taste papilla density and maintenance. PLoS One.

[26] Dalziel M, Crispin M, Scanlan CN, Zitzmann N, Dwek RA (2014). Emerging principles for the therapeutic exploitation of glycosylation. Science.

[27] Tabasum ST, Nayak RP (2011). Salivary blood group antigens and microbial flora. Int J Dental Hygiene.

[28] Manthorpe R, Staub Nielsen L, Hagen Petersen S, Prause JU (1985). Lewis blood type frequency in patients with primary Sjogren’s syndrome. A prospective study including analyses for A1A2BO, Secretor, MNSs, P, Duffy, Kell, Lutheran and rhesus blood groups. Scand J Rheumatol.

[29] Maroni L, van de Graaf SF, Hohenester SD, OudeElferink RP, Beuers U (2014). Fucosyltransferase 2: a genetic risk factor for primary sclerosing cholangitis and Crohn’s disease-A comprehensive review. Clin Rev Allergy Immunol.

[30] Guile GR, Harvey DJ, O’Donnell N, Powell AK, Hunter AP, Zamze S (1998). Identification of highly fucosylated N-linked oligosaccharides from the human parotid gland. Eur J Biochem/FEBS..

[31] Thomsson KA, Schulz BL, Packer NH, Karlsson NG (2005). MUC5B glycosylation in human saliva reflects blood group and secretor status. Glycobiology.

[32] Everest-Dass AV, Jin D, Thaysen-Andersen M, Nevalainen H, Kolarich D, Packer NH (2012). Comparative structural analysis of the glycosylation of salivary and buccal cell proteins: innate protection against infection by Candida albicans. Glycobiology.

[33] Khoury GA, Baliban RC, Floudas CA (2011). Proteome-wide post-translational modification statistics: frequency analysis and curation of the swiss-prot database. Scientific Reports.

[34] Prakobphol A, Xu F, Hoang VM, Larsson T, Bergstrom J, Johansson I (2000). Salivary agglutinin, which binds Streptococcus mutans and Helicobacter pylori, is the lung scavenger receptor cysteine-rich protein gp-340. J Biol Chem.

[35] Qin Y, Zhong Y, Zhu M, Dang L, Yu H, Chen Z (2013). Age- and sex-associated differences in the glycopatterns of human salivary glycoproteins and their roles against influenza A virus. J Proteome Res.

[36] Drake PM, Cho W, Li B, Prakobphol A, Johansen E, Anderson NL (2010). Sweetening the pot: adding glycosylation to the biomarker discovery equation. Clin Chem.

[37] Bandhakavi S, Stone MD, Onsongo G, Van Riper SK, Griffin TJ (2009). A dynamic range compression and three-dimensional peptide fractionation analysis platform expands proteome coverage and the diagnostic potential of whole saliva. J Proteome Res.

[38] Everest-Dass AV, Abrahams JL, Kolarich D, Packer NH, Campbell MP (2013). Structural feature ions for distinguishing N- and O-linked glycan isomers by LC-ESI-IT MS/MS. J Am Soc Mass Spectrom.

[39] Sondej M, Denny PA, Xie Y, Ramachandran P, Si Y, Takashima J (2009). Glycoprofiling of the human salivary proteome. Clin Proteomics.

[40] Bandhakavi S, Van Riper SK, Tawfik PN, Stone MD, Haddad T, Rhodus NL (2011). Hexapeptide libraries for enhanced protein PTM identification and relative abundance profiling in whole human saliva. J Proteome Res.

[41] Ramachandran P, Boontheung P, Pang E, Yan W, Wong DT, Loo JA (2008). Comparison of N-linked glycoproteins in human whole saliva, parotid, submandibular, and sublingual glandular secretions identified using hydrazide chemistry and mass spectrometry. Clin Proteomics.

[42] Larsen MR, Jensen SS, Jakobsen LA, Heegaard NH (2007). Exploring the sialiome using titanium dioxide chromatography and mass spectrometry. Mol Cell Proteomics.

[43] Sun S, Zhao F, Wang Q, Zhong Y, Cai T, Wu P (2014). Analysis of age and gender associated *N*-glycoproteome in human whole saliva. Clin Proteomics.

[44] Vitorino R, Alves R, Barros A, Caseiro A, Ferreira R, Lobo MC (2010). Finding new posttranslational modifications in salivary proline-rich proteins. Proteomics.

[45] Xu Y, Bailey UM, Punyadeera C, Schulz BL (2014). Identification of salivary *N*-glycoproteins and measurement of glycosylation site occupancy by boronate glycoprotein enrichment and liquid chromatography/electrospray ionization tandem mass spectrometry. Rapid Commun Mass Spectro RCM.

[46] Hardt M, Witkowska HE, Webb S, Thomas LR, Dixon SE, Hall SC (2005). Assessing the effects of diurnal variation on the composition of human parotid saliva: quantitative analysis of native peptides using iTRAQ reagents. Anal Chem.

[47] Lashley K (1916). Reflex secretion of the human parotid gland. J Exp Psychol.

[48] Block P, Brotman S (1962). A method of submaxillary saliva collection without cannulization. NY State Dent J.

[49] Prakobphol A, Leffler H, Fisher SJ (1993). The high-molecular-weight human mucin is the primary salivary carrier of ABH, Le(a), and Le(b) blood group antigens. Critic Rev Oral Biol Med Off Publ Am Assoc Oral Biol.

[50] Drake PM, Schilling B, Niles RK, Prakobphol A, Li B, Jung K (2012). Lectin chromatography/mass spectrometry discovery workflow identifies putative biomarkers of aggressive breast cancers. J Proteome Res.

[51] Shevchenko A, Tomas H, Havlis J, Olsen JV, Mann M (2006). In-gel digestion for mass spectrometric characterization of proteins and proteomes. Nat Protoc.

[52] Protein Prospector. http://prospector.ucsf.edu. Accessed 20 May 2014.

[53] Chalkley RJ, Hansen KC, Baldwin MA (2005). Bioinformatic methods to exploit mass spectrometric data for proteomic applications. Methods Enzymol.

[54] Liu H, Sadygov RG, Yates JR (2004). A model for random sampling and estimation of relative protein abundance in shotgun proteomics. Anal Chem.

[55] Everest-Dass AV, Abrahams JL, Kolarich D, Packer NH, Campbell MP (2013). Structural feature ions for distinguishing N- and O-linked glycan isomers by LC-ESI-IT MS/MS. J Am Soc Mass Spectrom.

[56] Papac DI, Briggs JB, Chin ET, Jones AJ (1998). A high-throughput microscale method to release N-linked oligosaccharides from glycoproteins for matrix-assisted laser desorption/ionization time-of-flight mass spectrometric analysis. Glycobiology.

[57] Cooper CA, Gasteiger E, Packer NH (2001). GlycoMod–a software tool for determining glycosylation compositions from mass spectrometric data. Proteomics.

[58] GlycoMod. http://expasy.org/glycomod. Accessed 29 Nov 2014.

[59] Ceroni A, Dell A, Haslam SM (2007). The GlycanBuilder: a fast, intuitive and flexible software tool for building and displaying glycan structures. Source Code Biol Med.

[60] Ceroni A, Maass K, Geyer H, Geyer R, Dell A, Haslam SM (2008). GlycoWorkbench: a tool for the computer-assisted annotation of mass spectra of glycans. J Proteome Res.

[61] Varki A, Cummings RD, Esko JD, Freeze HH, Stanley P, Marth JD (2009). Symbol nomenclature for glycan representation. Proteomics.

[62] Ahmed H, Chatterjee BP (1989). Further characterization and immunochemical studies on the carbohydrate specificity of jackfruit (Artocarpus integrifolia) lectin. J Biol Chem.

[63] Kaladas PM, Kabat EA, Iglesias JL, Lis H, Sharon N (1982). Immunochemical studies on the combining site of the *D*-galactose/*N*-acetyl-d-galactosamine specific lectin from Erythrina cristagalli seeds. Arch Biochem Biophys.

[64] Swamy MJ, Gupta D, Mahanta SK, Surolia A (1991). Further characterization of the saccharide specificity of peanut (Arachis hypogaea) agglutinin. Carbohydr Res.

[65] Piller V, Piller F, Cartron JP (1990). Comparison of the carbohydrate-binding specificities of seven *N*-acetyl-d-galactosamine-recognizing lectins. Eur J Biochem/FEBS.

[66] Shen ZM, Shi WX, Sun C, Yang JT (1993). Conformation and activity of mannose- and *N*-acetylgalactosamine-specific lectins from Vicia villosa seeds. Biochimie.

[67] Crowley JF, Goldstein IJ, Arnarp J, Lonngren J (1984). Carbohydrate binding studies on the lectin from Datura stramonium seeds. Arch Biochem Biophys.

[68] Nachbar MS, Oppenheim JD, Thomas JO (1980). Lectins in the US Diet. isolation and characterization of a lectin from the tomato (Lycopersicon esculentum). J Biol Chem.

[69] Allen AK, Neuberger A, Sharon N (1973). The purification, composition and specificity of wheat-germ agglutinin. Biochem J.

[70] Peters BP, Ebisu S, Goldstein IJ, Flashner M (1979). Interaction of wheat germ agglutinin with sialic acid. Biochemistry.

[71] Yamamoto K, Tsuji T, Matsumoto I, Osawa T (1981). Structural requirements for the binding of oligosaccharides and glycopeptides to immobilized wheat germ agglutinin. Biochemistry.

[72] Debray H, Decout D, Strecker G, Spik G, Montreuil J (1981). Specificity of twelve lectins towards oligosaccharides and glycopeptides related to N-glycosylproteins. Eur J Biochem/FEBS.

[73] Shibuya N, Goldstein IJ, Van Damme EJ, Peumans WJ (1988). Binding properties of a mannose-specific lectin from the snowdrop (Galanthus nivalis) bulb. J Biol Chem.

[74] Debray H, Montreuil J (1989). Aleuria aurantia agglutinin. A new isolation procedure and further study of its specificity towards various glycopeptides and oligosaccharides. Carbohydr Res.

[75] Pereira ME, Kabat EA (1974). Blood group specificity of the lectin from Lotus tetragonolobus. Ann N Y Acad Sci.

[76] Shibuya N, Goldstein IJ, Broekaert WF, Nsimba-Lubaki M, Peeters B, Peumans WJ (1987). The elderberry (Sambucus nigra L.) bark lectin recognizes the Neu5Ac(alpha 2-6)Gal/GalNAc sequence. J Biol Chem.

[77] Petryniak J, Goldstein IJ (1987). Evonymus europaea lectin. Methods Enzymol.

[78] Cummings RD, Kornfeld S (1982). Characterization of the structural determinants required for the high affinity interaction of asparagine-linked oligosaccharides with immobilized Phaseolus vulgaris leukoagglutinating and erythroagglutinating lectins. J Biol Chem.

