# Supplementary figures and images for: Mass spectrometry-based analyses showing the effects of secretor and blood group status on salivary N-glycosylation
Source: Clin Proteomics. 2015 Dec 30;12:29. doi: 10.1186/s12014-015-9100-y (PMC4696288; doi:10.1186/s12014-015-9100-y)

## Slide 1
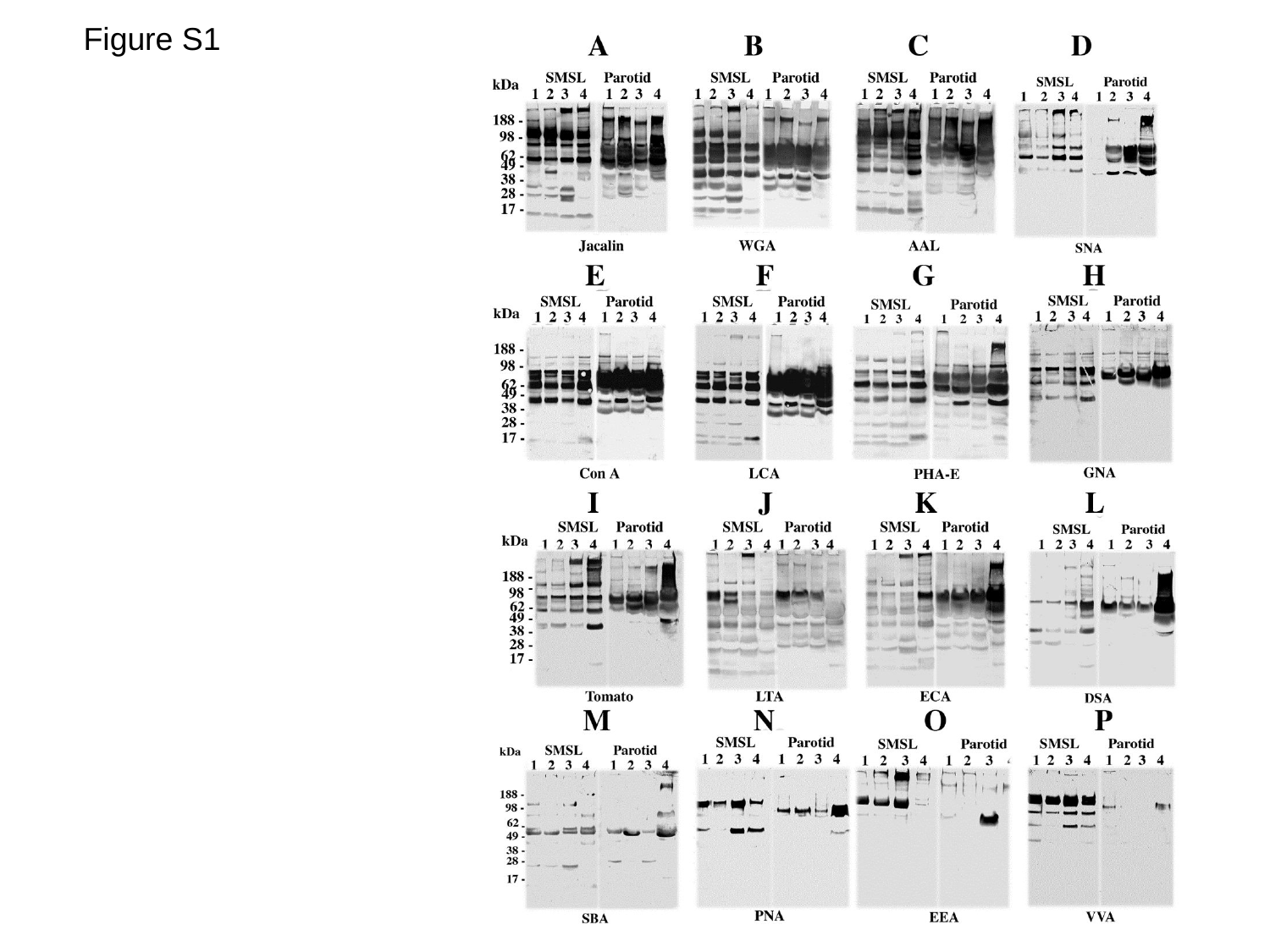

Figure S1

Supplement: Supplementary file 1 — 10.1186/s12014-015-9100-y Representative parotid and SMSL salivary protein reactivity with 16 lectins: [Artocarpus integrifolia (JAC), Wheat germ agglutinin (WGA), Aleuria aurantia lectin (AAL), Sambucus nigra (SNA), Canavalia ensiformis (Con A), Lens culinaris (LCA), Phaseolus vulgaris erythroagglutinating (PHA-E), Galanthus nivalis (GNA), Lycopersicon esculentum agglutinin (Tomato or LEA), Lotus tetragonolobus (LTA), Erythrina christagalli (ECA), Datura stramonium (DSA), Glycine max (soybean, SBA), Arachis hypogaea (peanut, PNA), Evonymus europaeus (EEA), Vicia villosa (VVA)]. AAL, JAC, and WGA interacted with the highest number of glycoproteins across a wide molecular weight range. Results are shown for parotid and SMSL saliva from 4 donors. [file 12014_2015_9100_MOESM1_ESM.pptx]
