# Supplementary material for: Mass spectrometry-based analyses showing the effects of secretor and blood group status on salivary N-glycosylation
Source: Clin Proteomics. 2015 Dec 30;12:29. doi: 10.1186/s12014-015-9100-y (PMC4696288; doi:10.1186/s12014-015-9100-y)
Supplement: Supplementary file 3 — 10.1186/s12014-015-9100-y Annotated MS and MS/MS spectra of deglycosylated N-linked glycopeptides identified in parotid and SMSL salivas. [file 12014_2015_9100_MOESM3_ESM.pptx]

## Slide 1
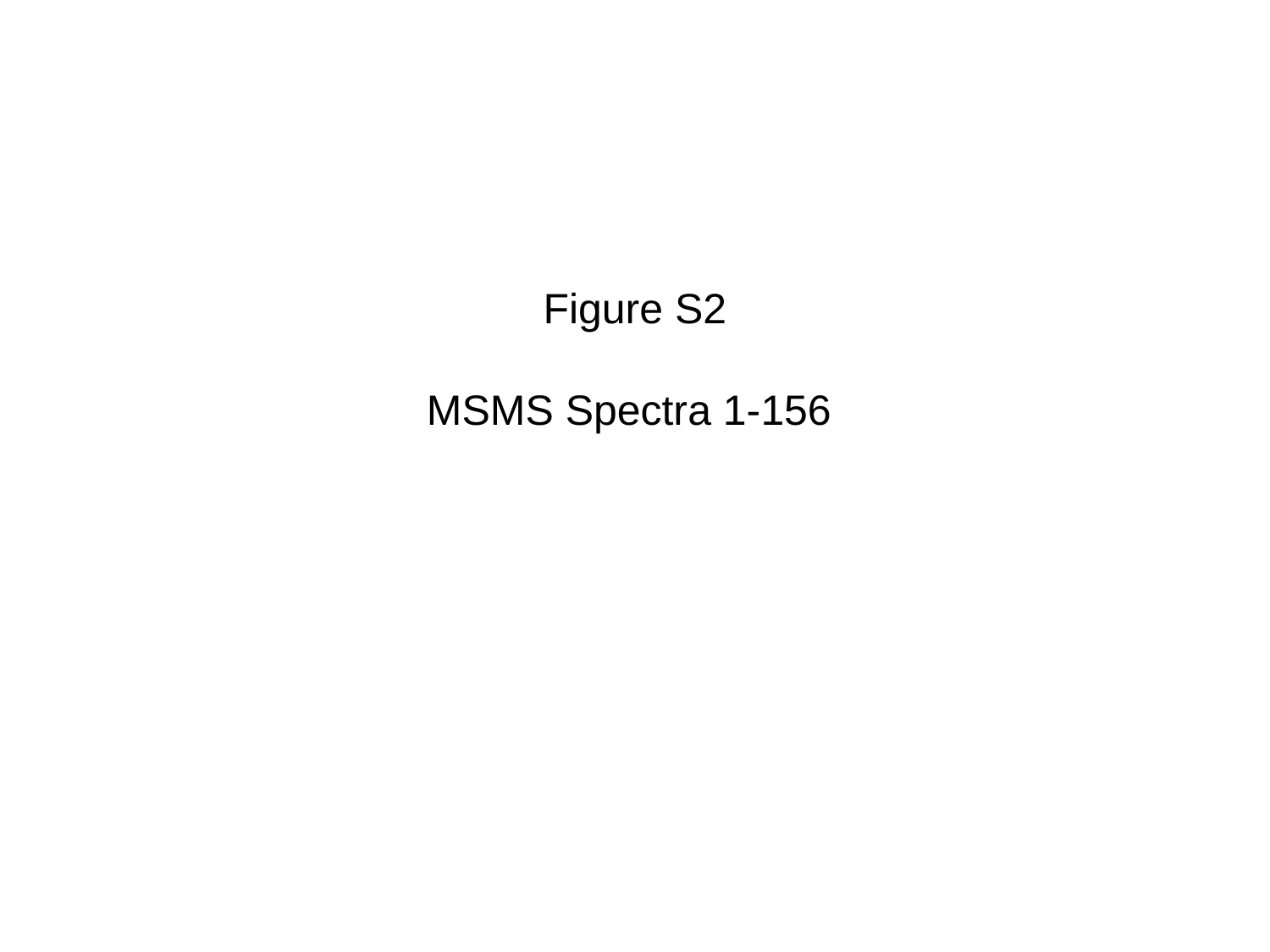

Figure S2
MSMS Spectra 1-156

## Slide 2
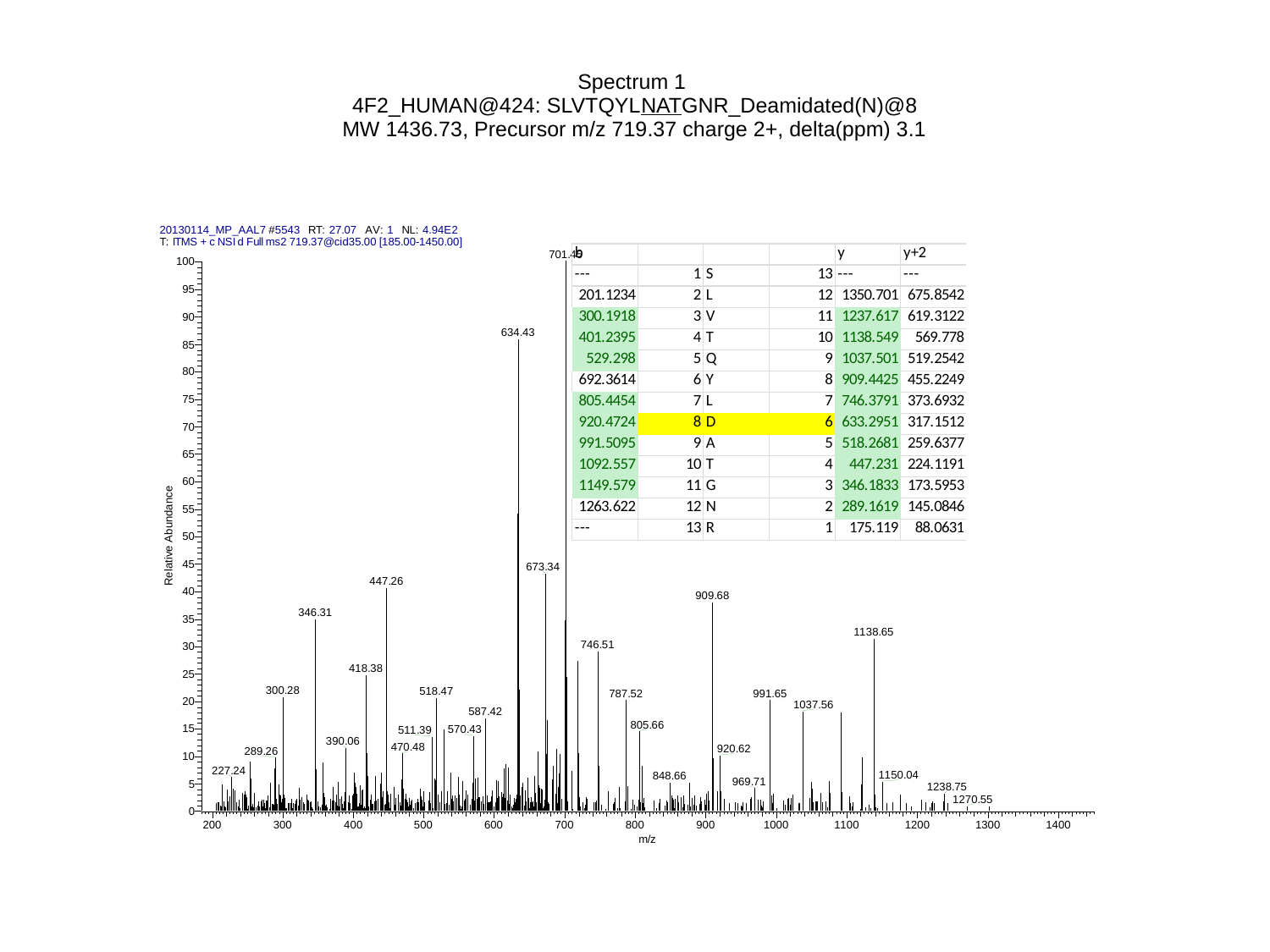

# Spectrum 1 4F2_HUMAN@424: SLVTQYLNATGNR_Deamidated(N)@8 MW 1436.73, Precursor m/z 719.37 charge 2+, delta(ppm) 3.1

## Slide 3
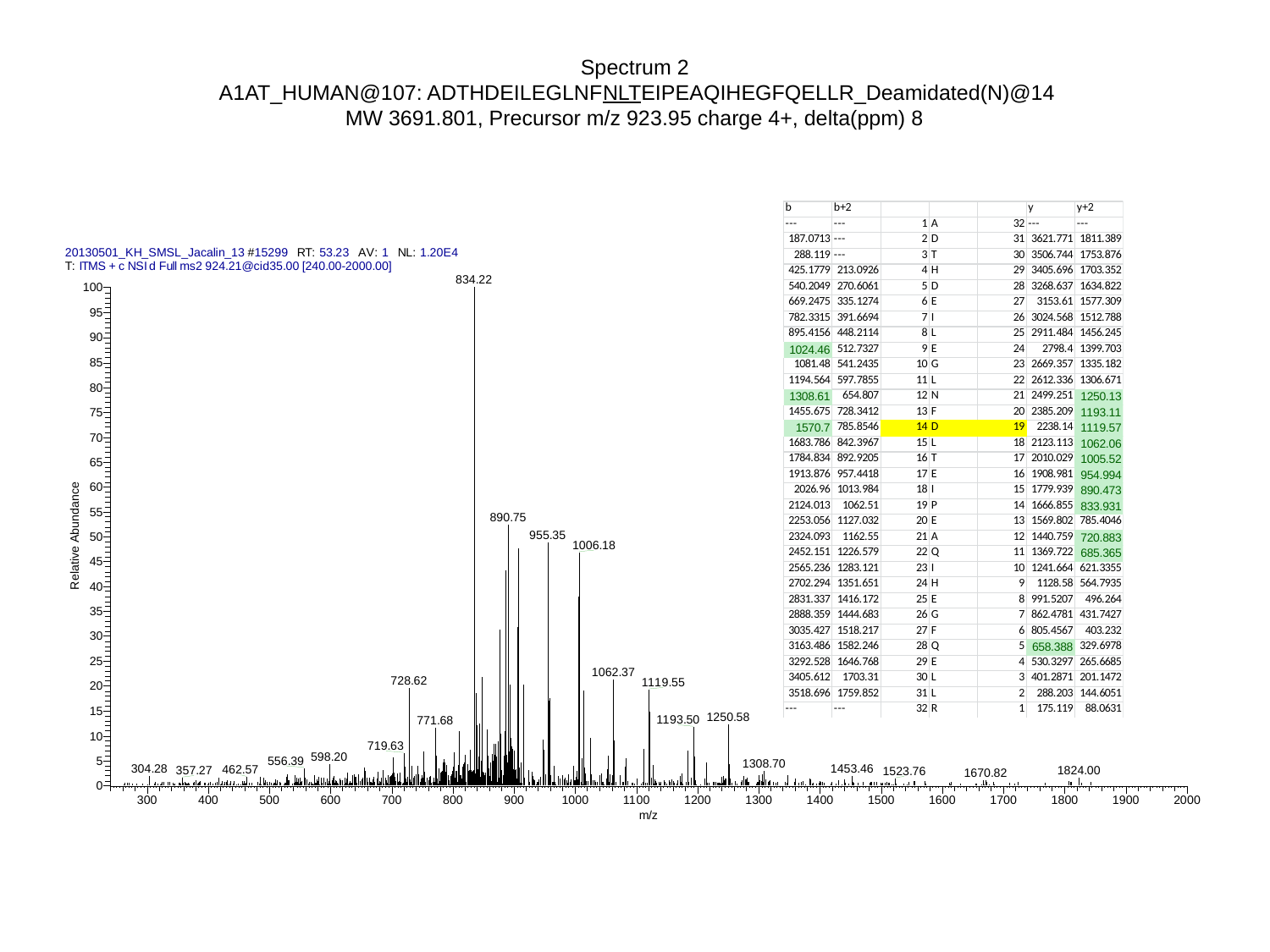

# Spectrum 2 A1AT_HUMAN@107: ADTHDEILEGLNFNLTEIPEAQIHEGFQELLR_Deamidated(N)@14 MW 3691.801, Precursor m/z 923.95 charge 4+, delta(ppm) 8

## Slide 4
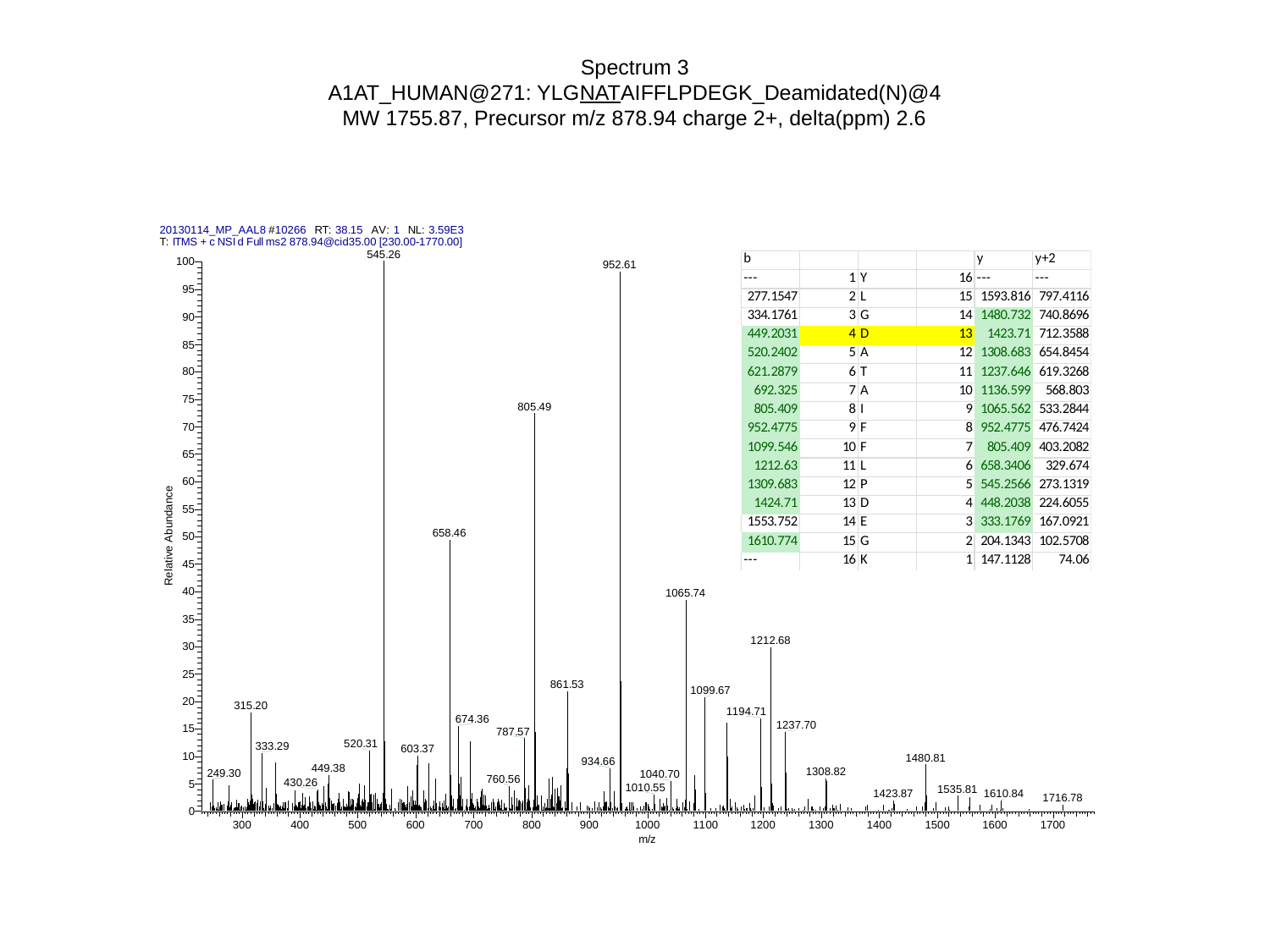

# Spectrum 3 A1AT_HUMAN@271: YLGNATAIFFLPDEGK_Deamidated(N)@4 MW 1755.87, Precursor m/z 878.94 charge 2+, delta(ppm) 2.6

## Slide 5
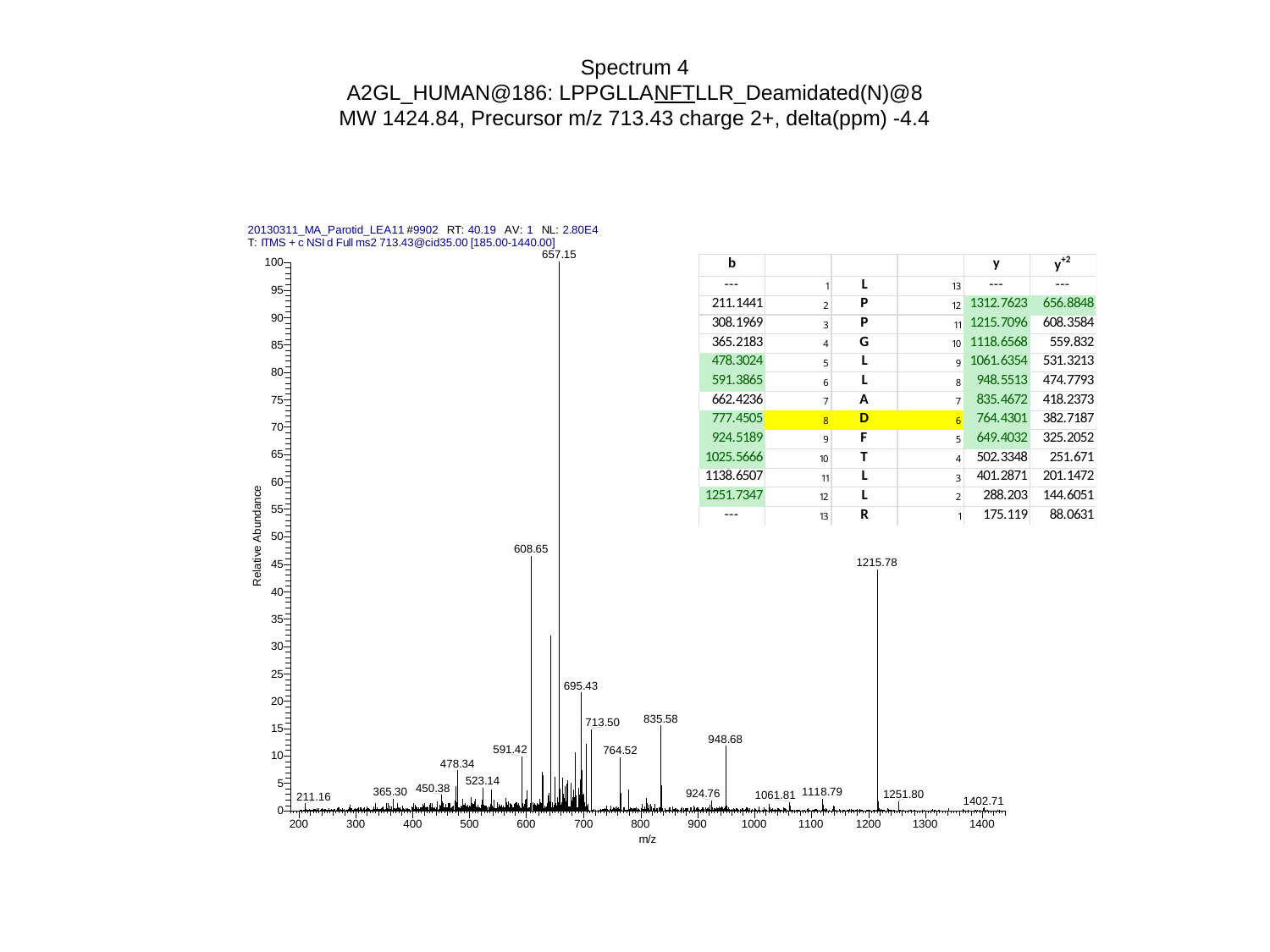

# Spectrum 4 A2GL_HUMAN@186: LPPGLLANFTLLR_Deamidated(N)@8 MW 1424.84, Precursor m/z 713.43 charge 2+, delta(ppm) -4.4

## Slide 6
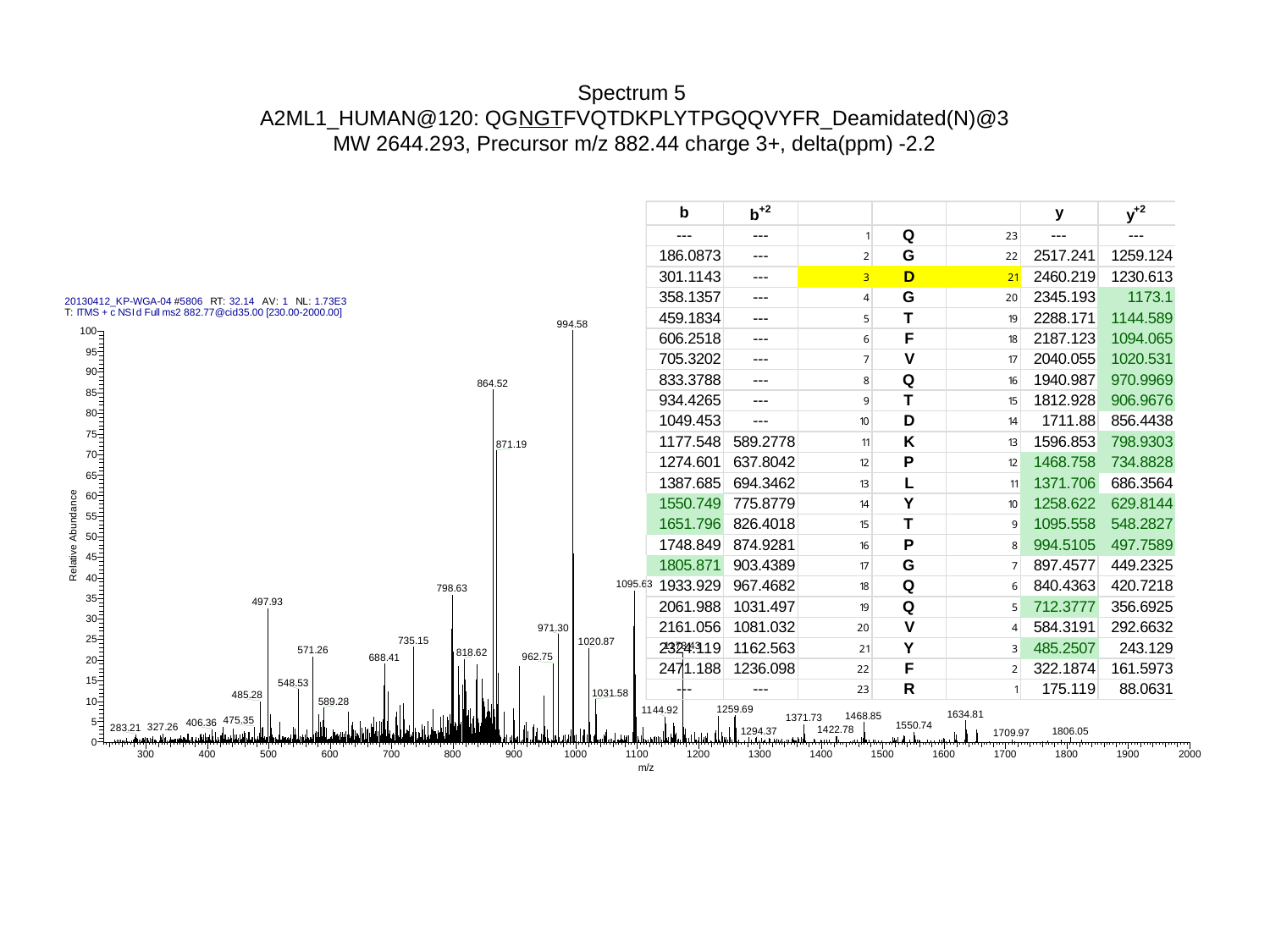

# Spectrum 5 A2ML1_HUMAN@120: QGNGTFVQTDKPLYTPGQQVYFR_Deamidated(N)@3 MW 2644.293, Precursor m/z 882.44 charge 3+, delta(ppm) -2.2

## Slide 7
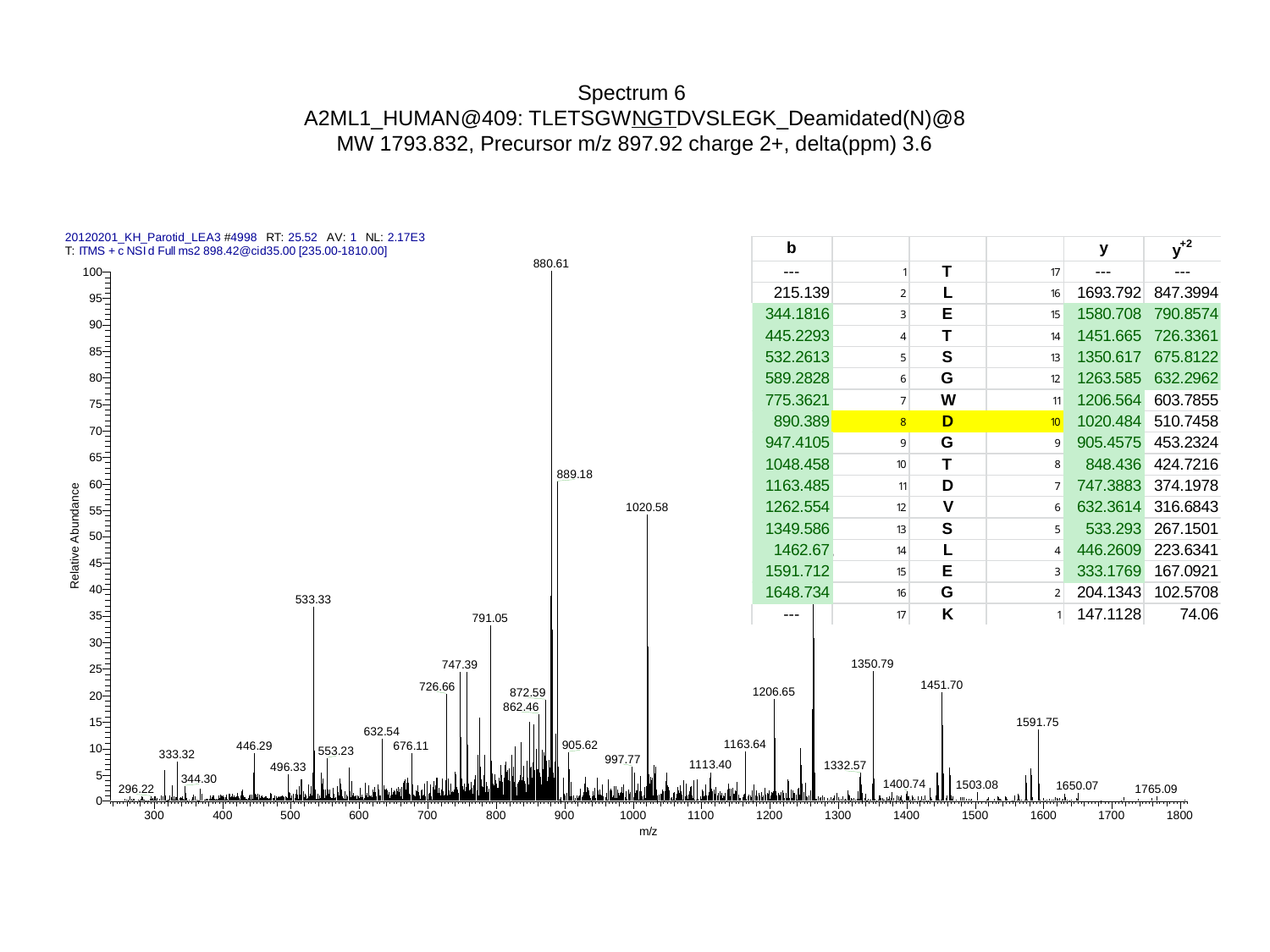

# Spectrum 6 A2ML1_HUMAN@409: TLETSGWNGTDVSLEGK_Deamidated(N)@8 MW 1793.832, Precursor m/z 897.92 charge 2+, delta(ppm) 3.6

## Slide 8
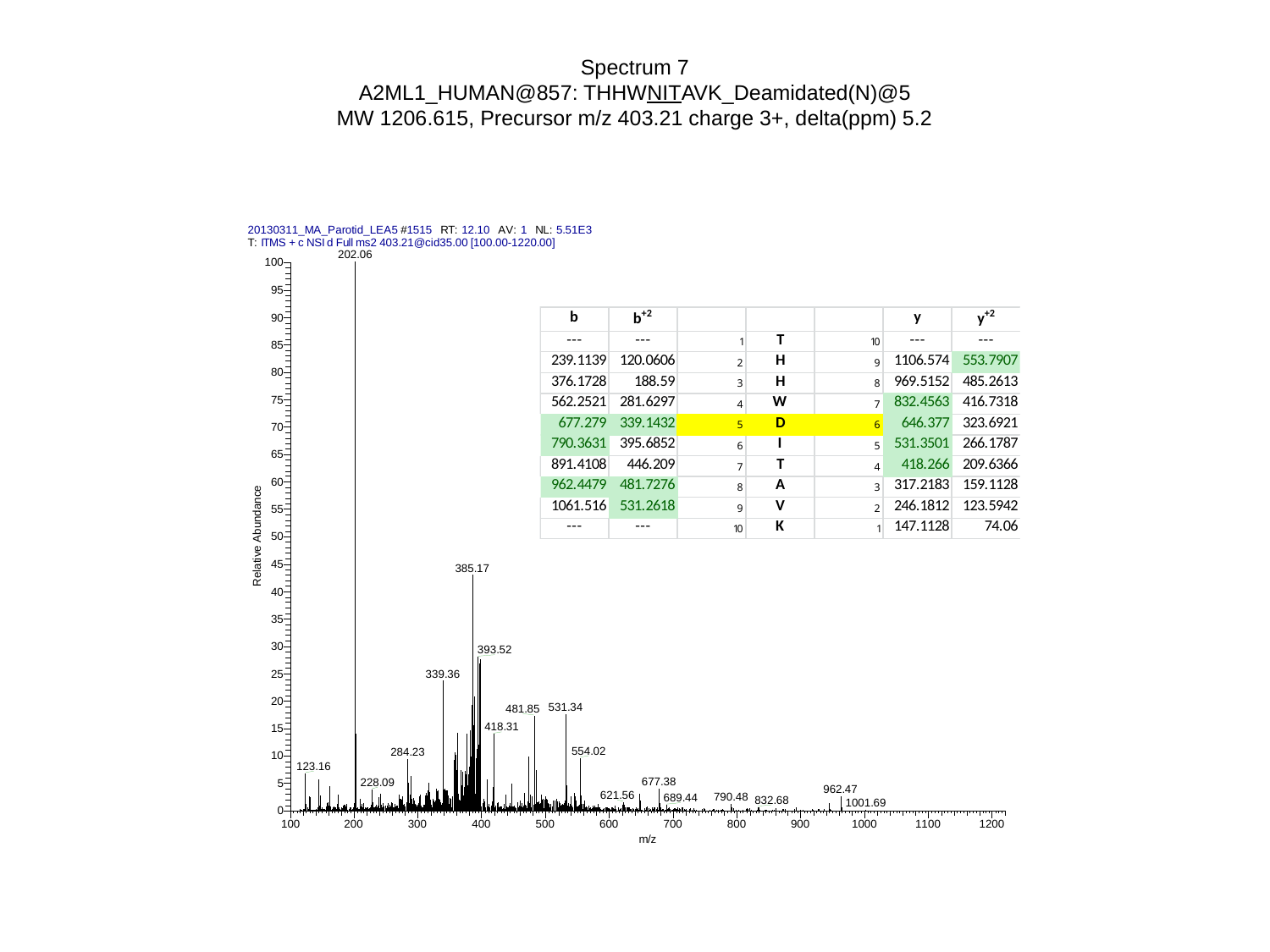

# Spectrum 7 A2ML1_HUMAN@857: THHWNITAVK_Deamidated(N)@5 MW 1206.615, Precursor m/z 403.21 charge 3+, delta(ppm) 5.2

## Slide 9
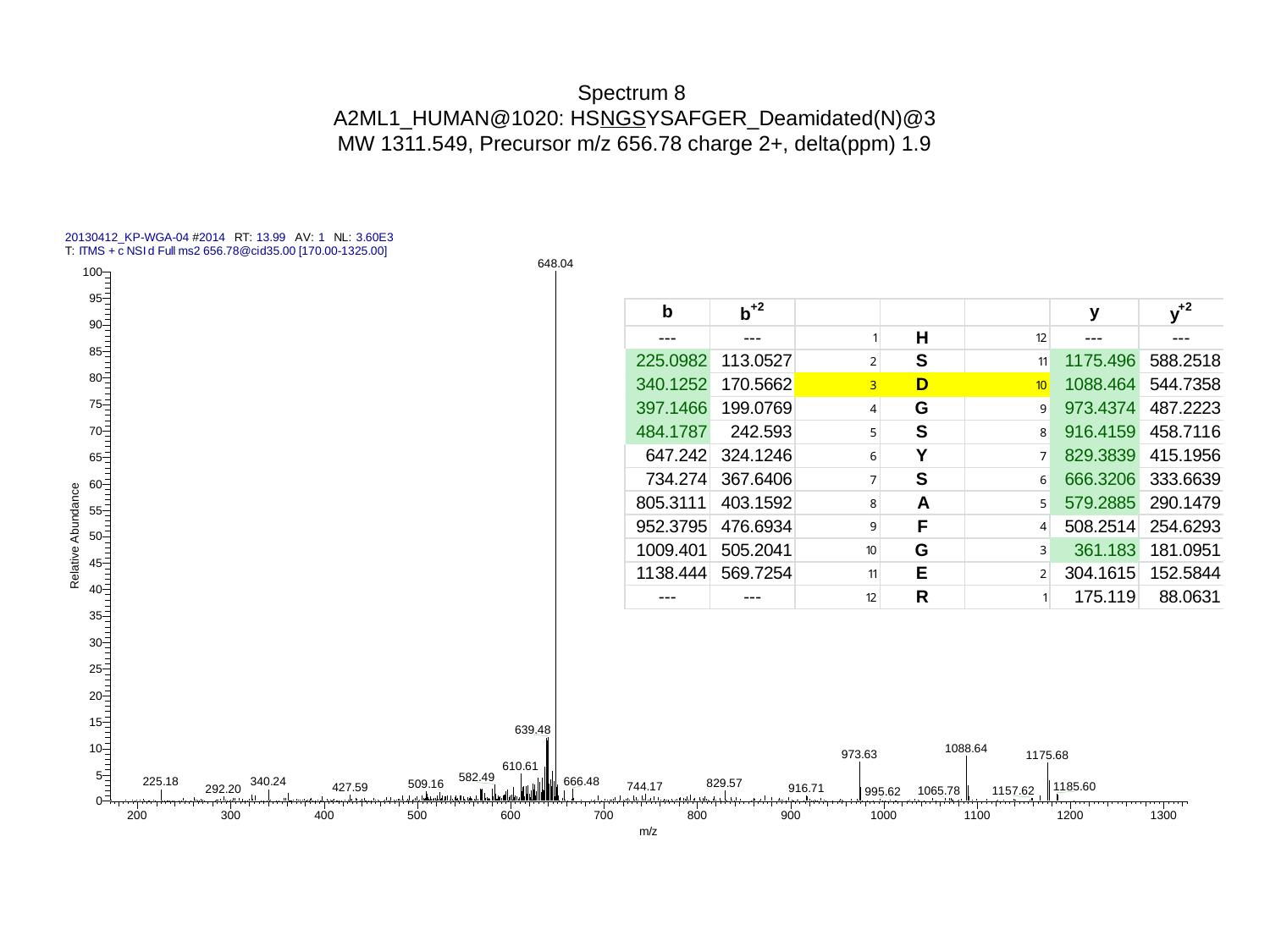

# Spectrum 8 A2ML1_HUMAN@1020: HSNGSYSAFGER_Deamidated(N)@3 MW 1311.549, Precursor m/z 656.78 charge 2+, delta(ppm) 1.9

## Slide 10
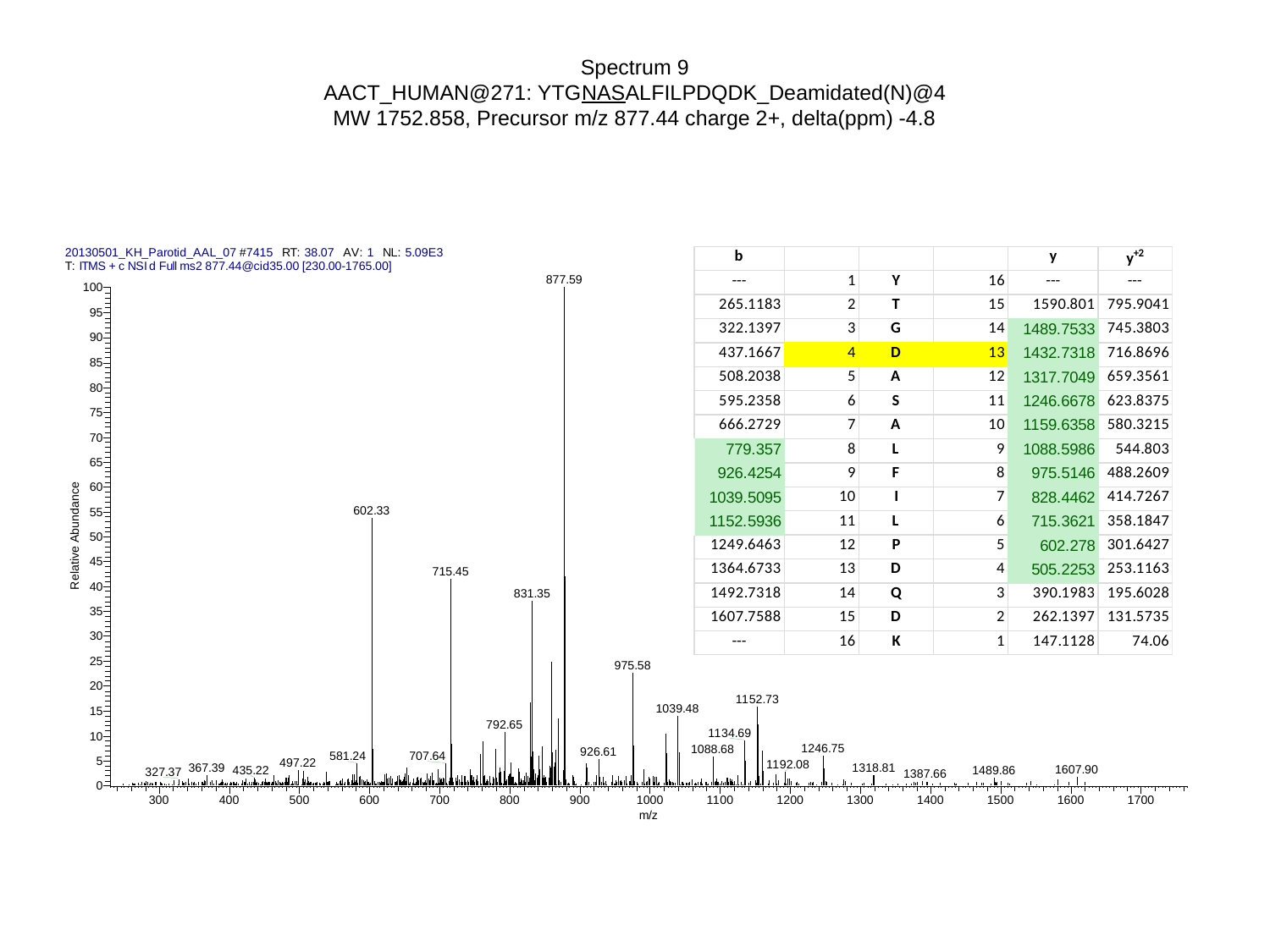

# Spectrum 9 AACT_HUMAN@271: YTGNASALFILPDQDK_Deamidated(N)@4 MW 1752.858, Precursor m/z 877.44 charge 2+, delta(ppm) -4.8

## Slide 11
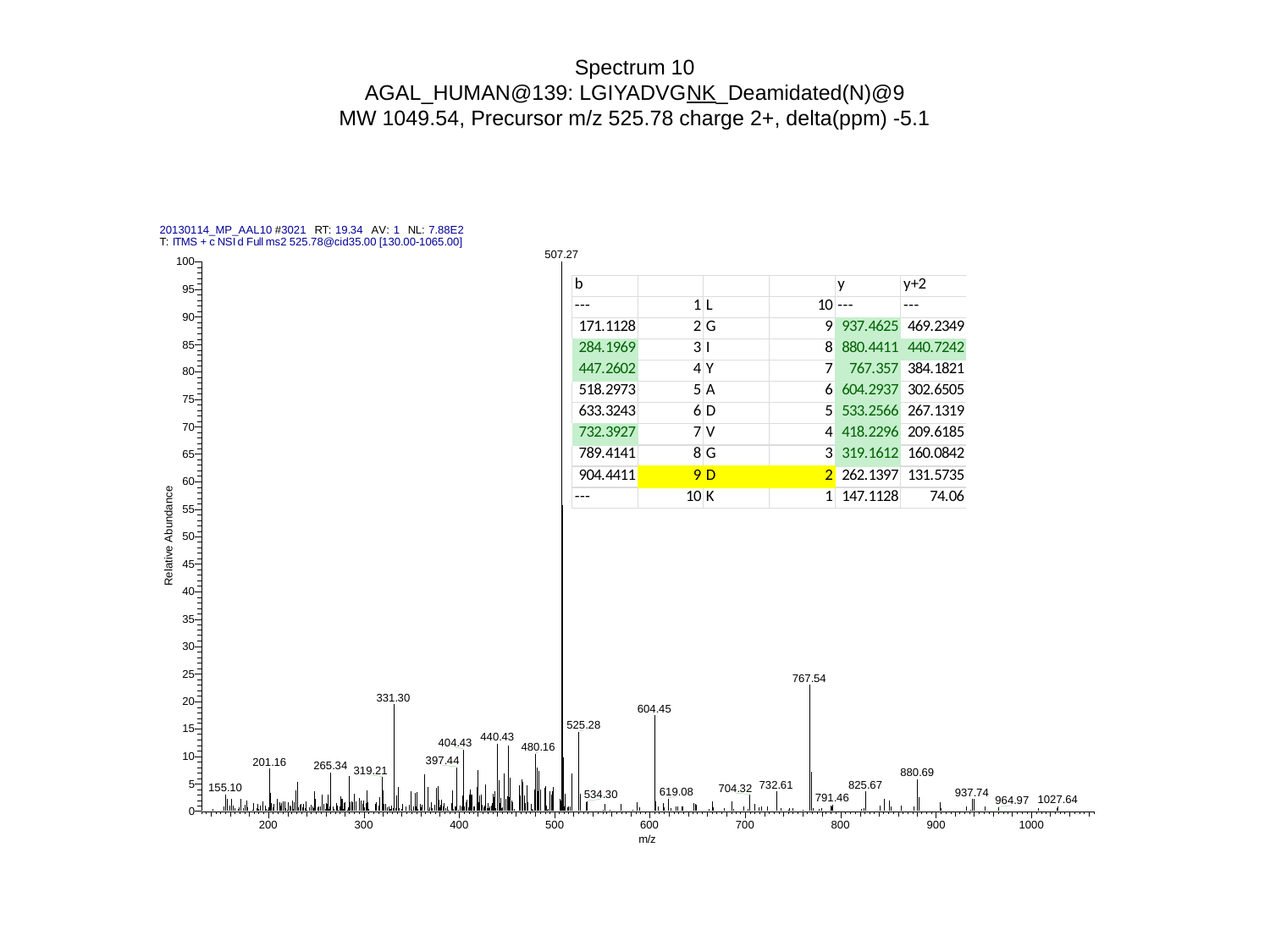

# Spectrum 10 AGAL_HUMAN@139: LGIYADVGNK_Deamidated(N)@9 MW 1049.54, Precursor m/z 525.78 charge 2+, delta(ppm) -5.1

## Slide 12
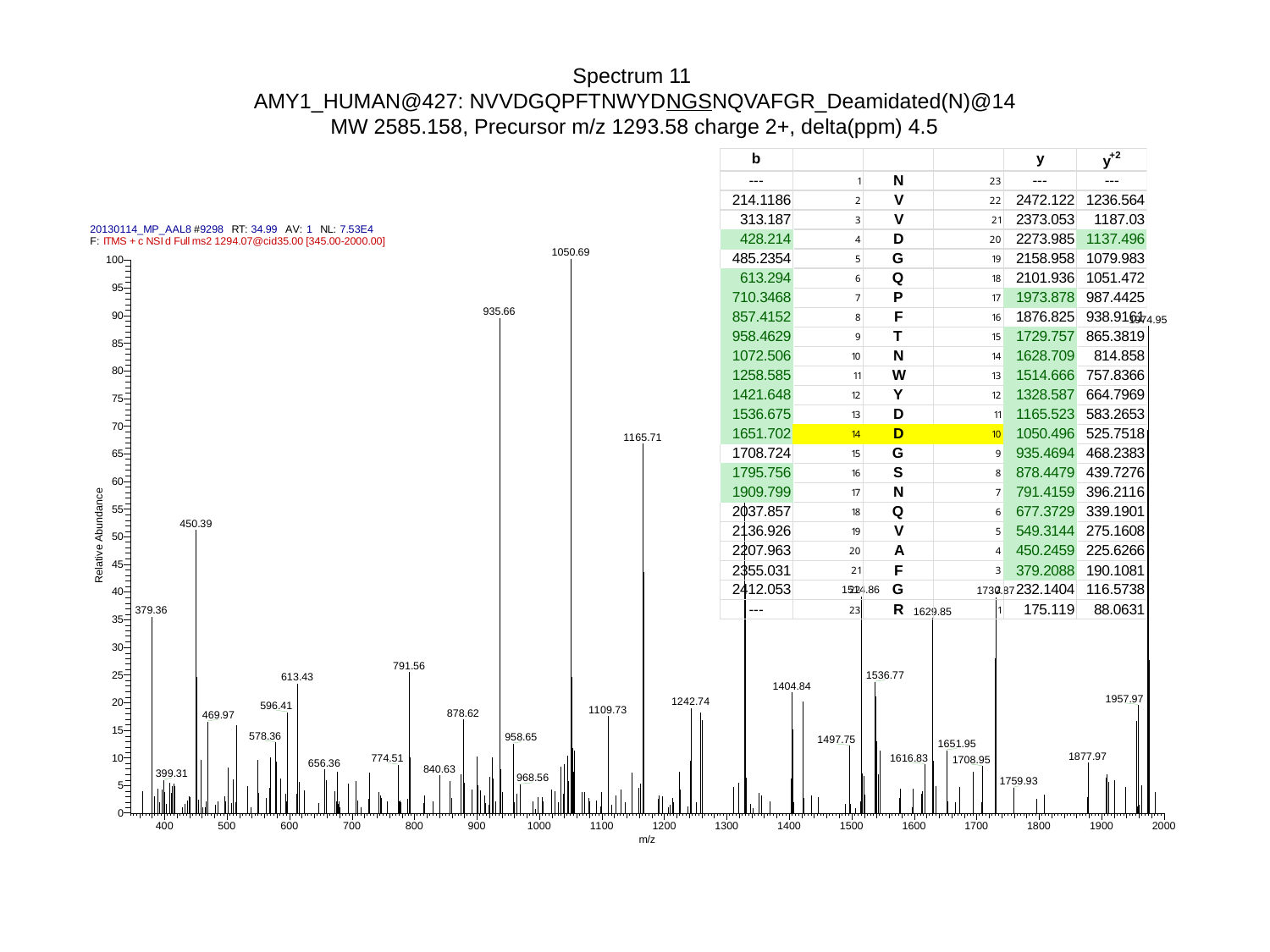

# Spectrum 11 AMY1_HUMAN@427: NVVDGQPFTNWYDNGSNQVAFGR_Deamidated(N)@14 MW 2585.158, Precursor m/z 1293.58 charge 2+, delta(ppm) 4.5

## Slide 13
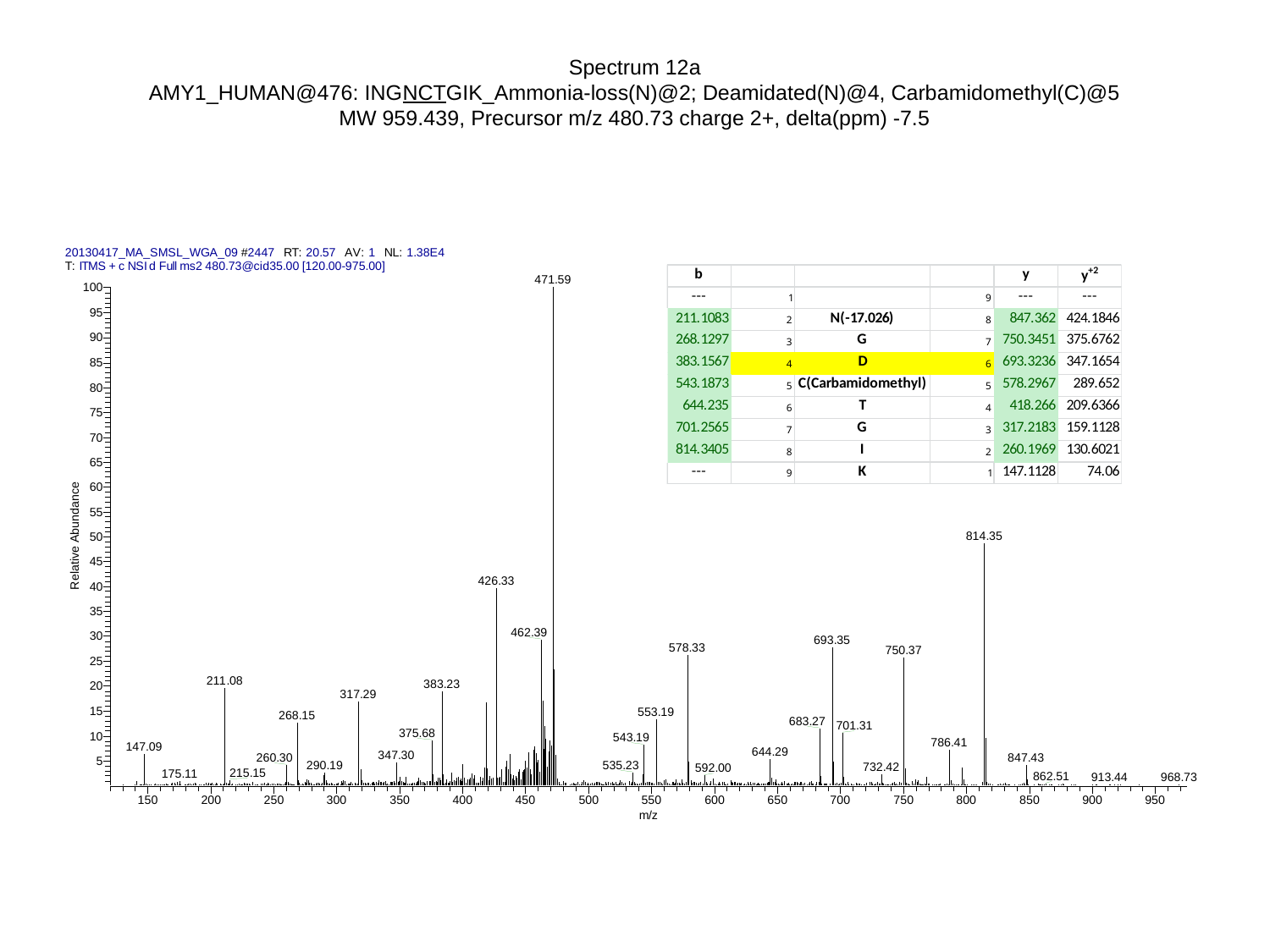

# Spectrum 12a AMY1_HUMAN@476: INGNCTGIK_Ammonia-loss(N)@2; Deamidated(N)@4, Carbamidomethyl(C)@5 MW 959.439, Precursor m/z 480.73 charge 2+, delta(ppm) -7.5

## Slide 14
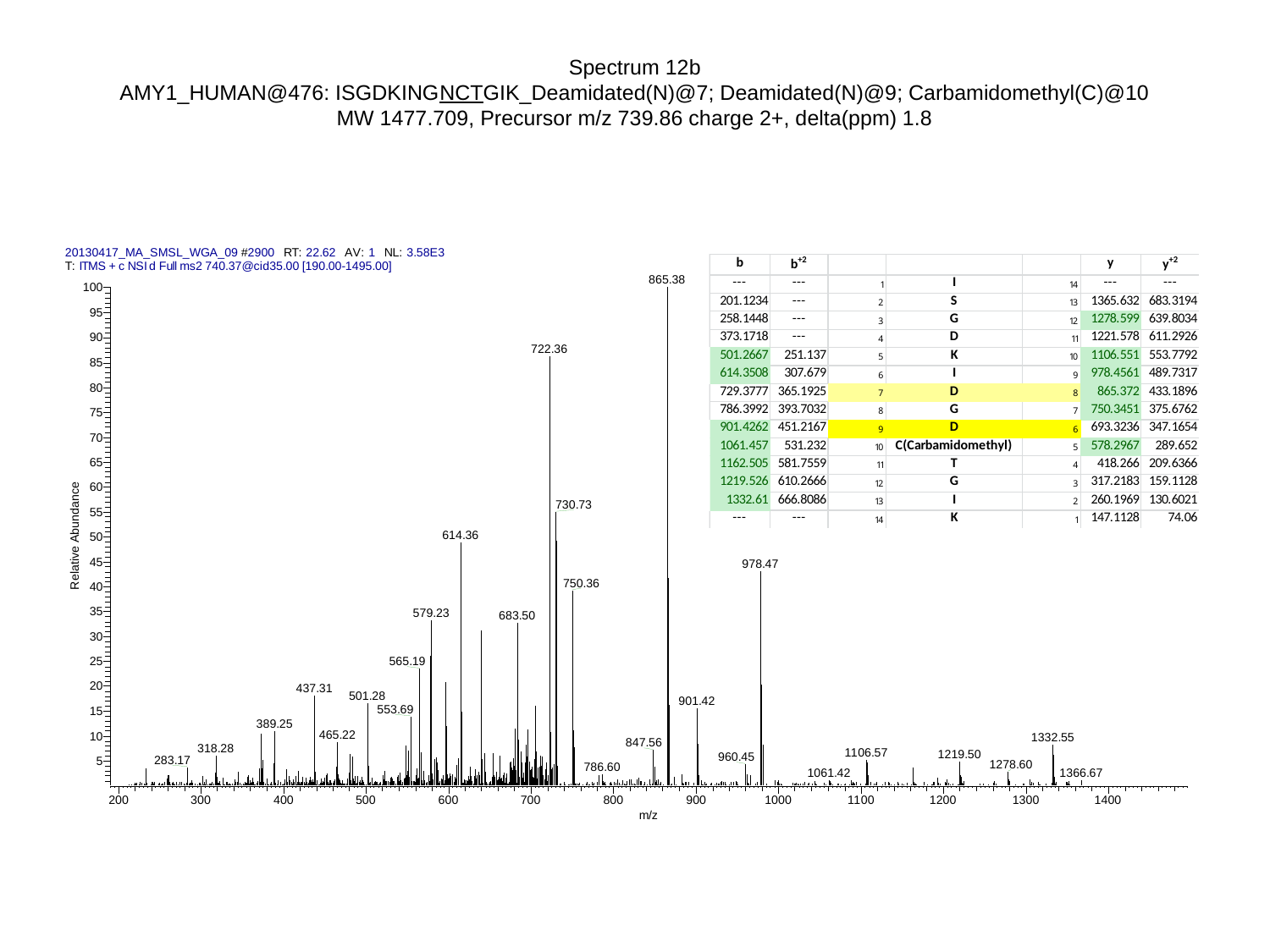

# Spectrum 12b AMY1_HUMAN@476: ISGDKINGNCTGIK_Deamidated(N)@7; Deamidated(N)@9; Carbamidomethyl(C)@10 MW 1477.709, Precursor m/z 739.86 charge 2+, delta(ppm) 1.8

## Slide 15
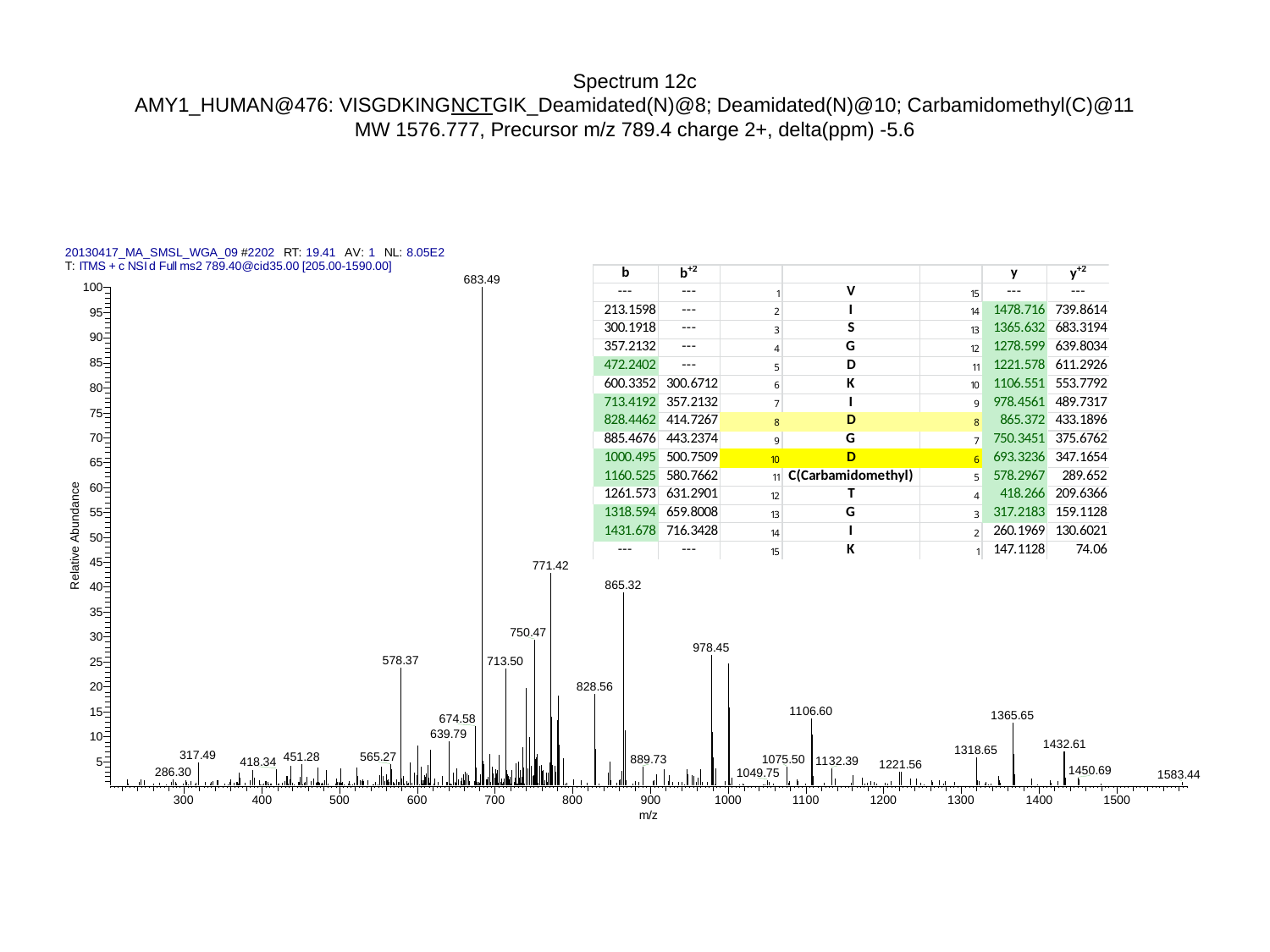

# Spectrum 12c AMY1_HUMAN@476: VISGDKINGNCTGIK_Deamidated(N)@8; Deamidated(N)@10; Carbamidomethyl(C)@11 MW 1576.777, Precursor m/z 789.4 charge 2+, delta(ppm) -5.6

## Slide 16
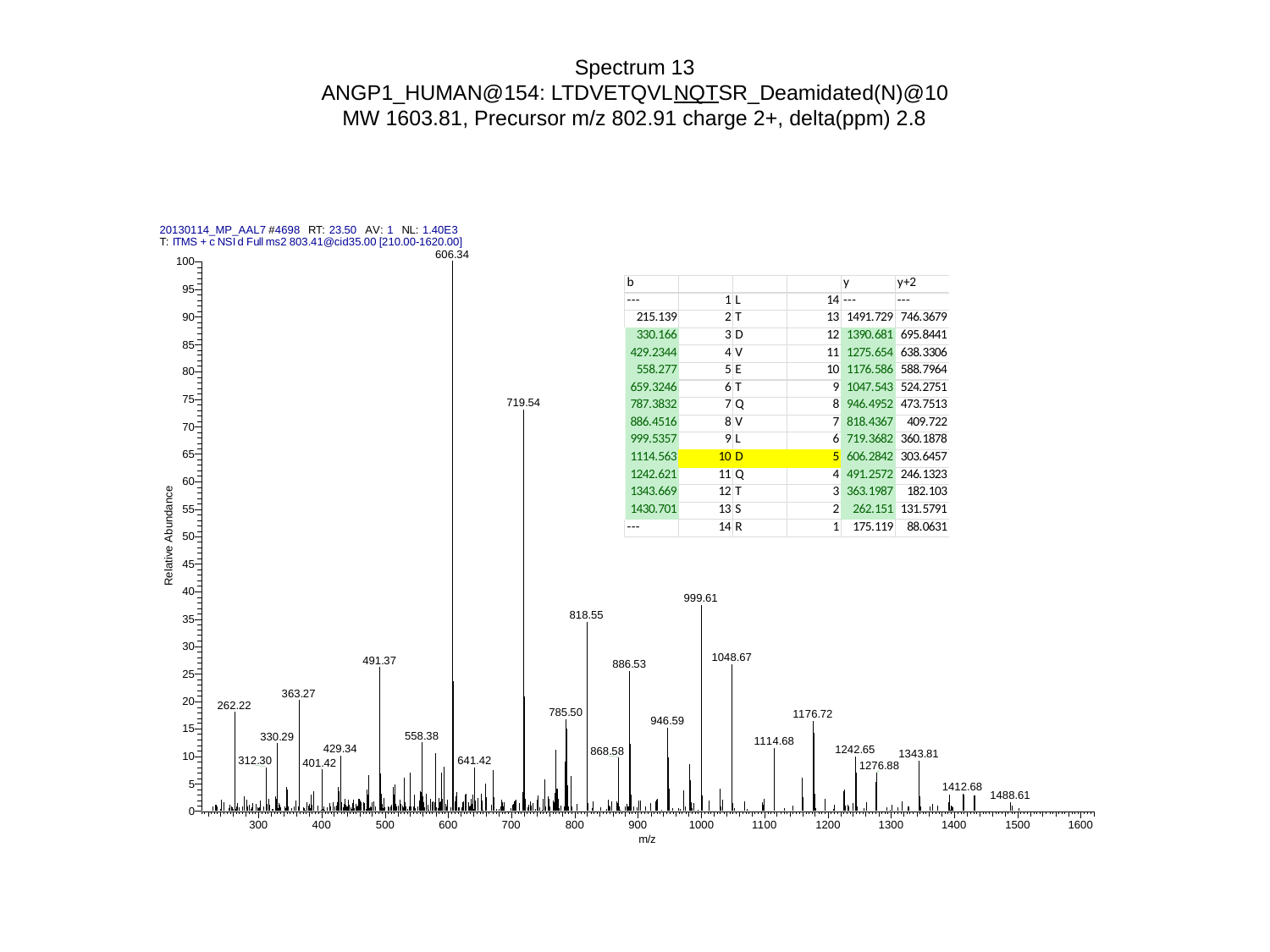

# Spectrum 13 ANGP1_HUMAN@154: LTDVETQVLNQTSR_Deamidated(N)@10 MW 1603.81, Precursor m/z 802.91 charge 2+, delta(ppm) 2.8

## Slide 17
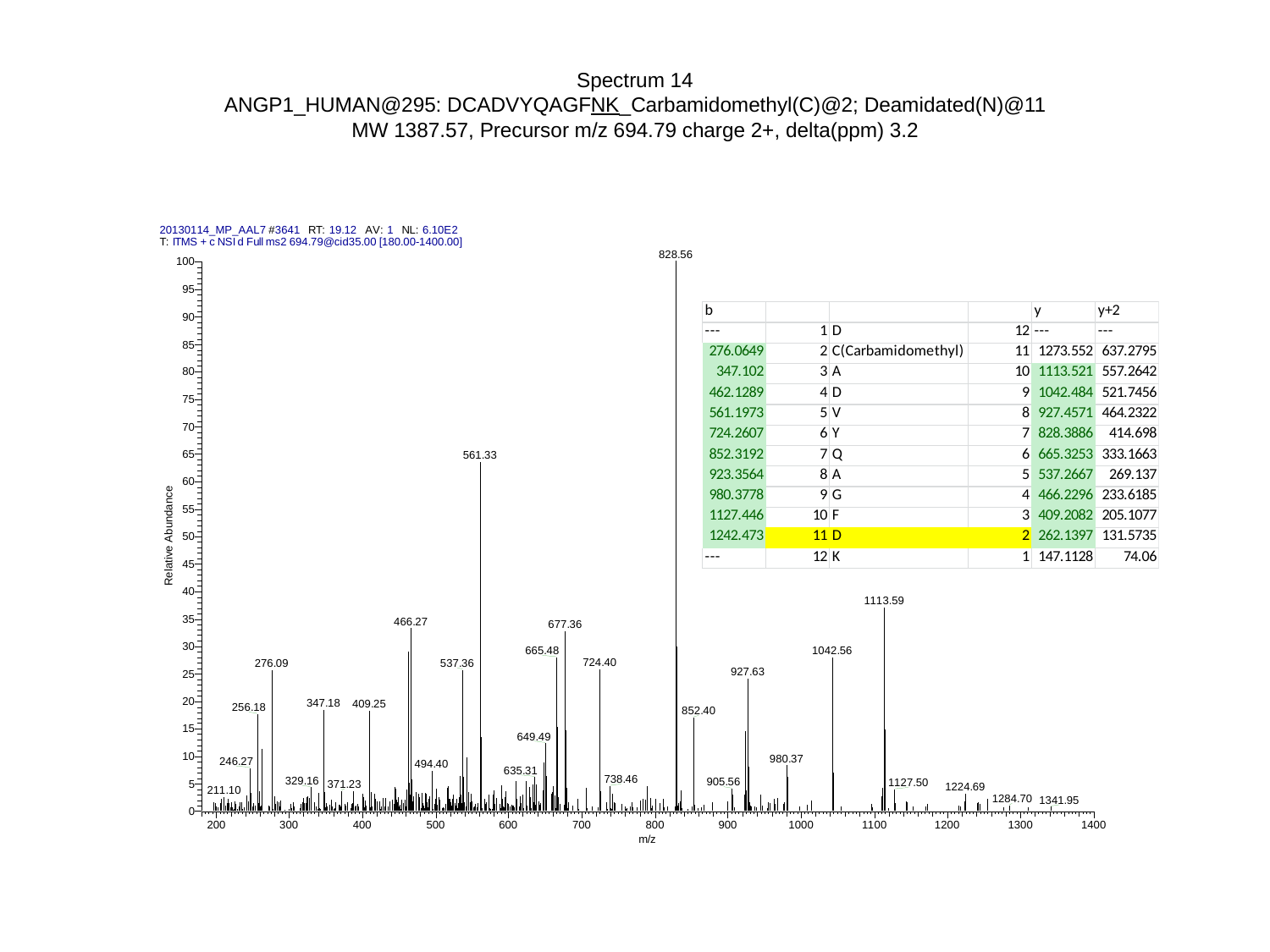

# Spectrum 14 ANGP1_HUMAN@295: DCADVYQAGFNK_Carbamidomethyl(C)@2; Deamidated(N)@11 MW 1387.57, Precursor m/z 694.79 charge 2+, delta(ppm) 3.2

## Slide 18
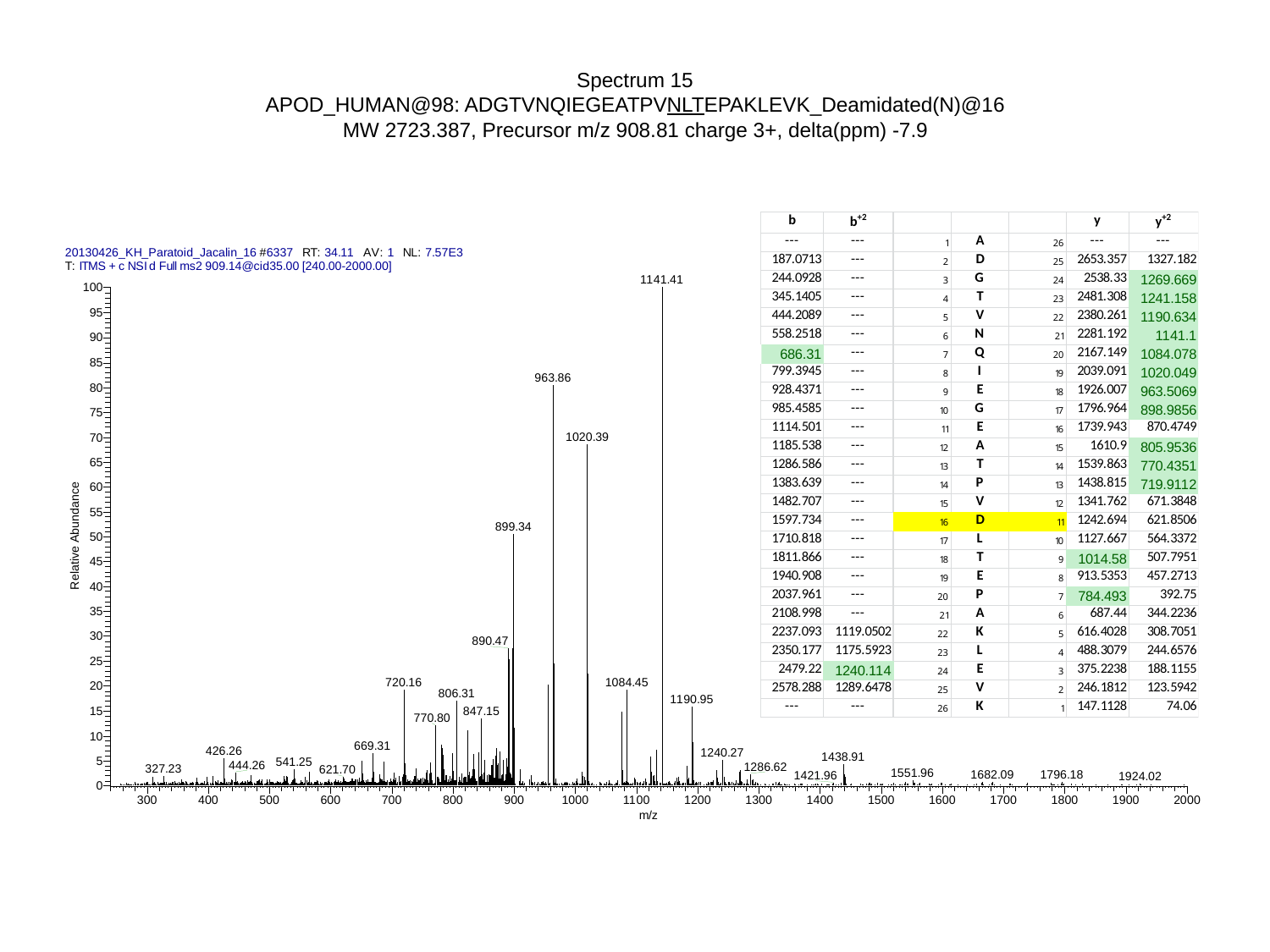

# Spectrum 15 APOD_HUMAN@98: ADGTVNQIEGEATPVNLTEPAKLEVK_Deamidated(N)@16 MW 2723.387, Precursor m/z 908.81 charge 3+, delta(ppm) -7.9

## Slide 19
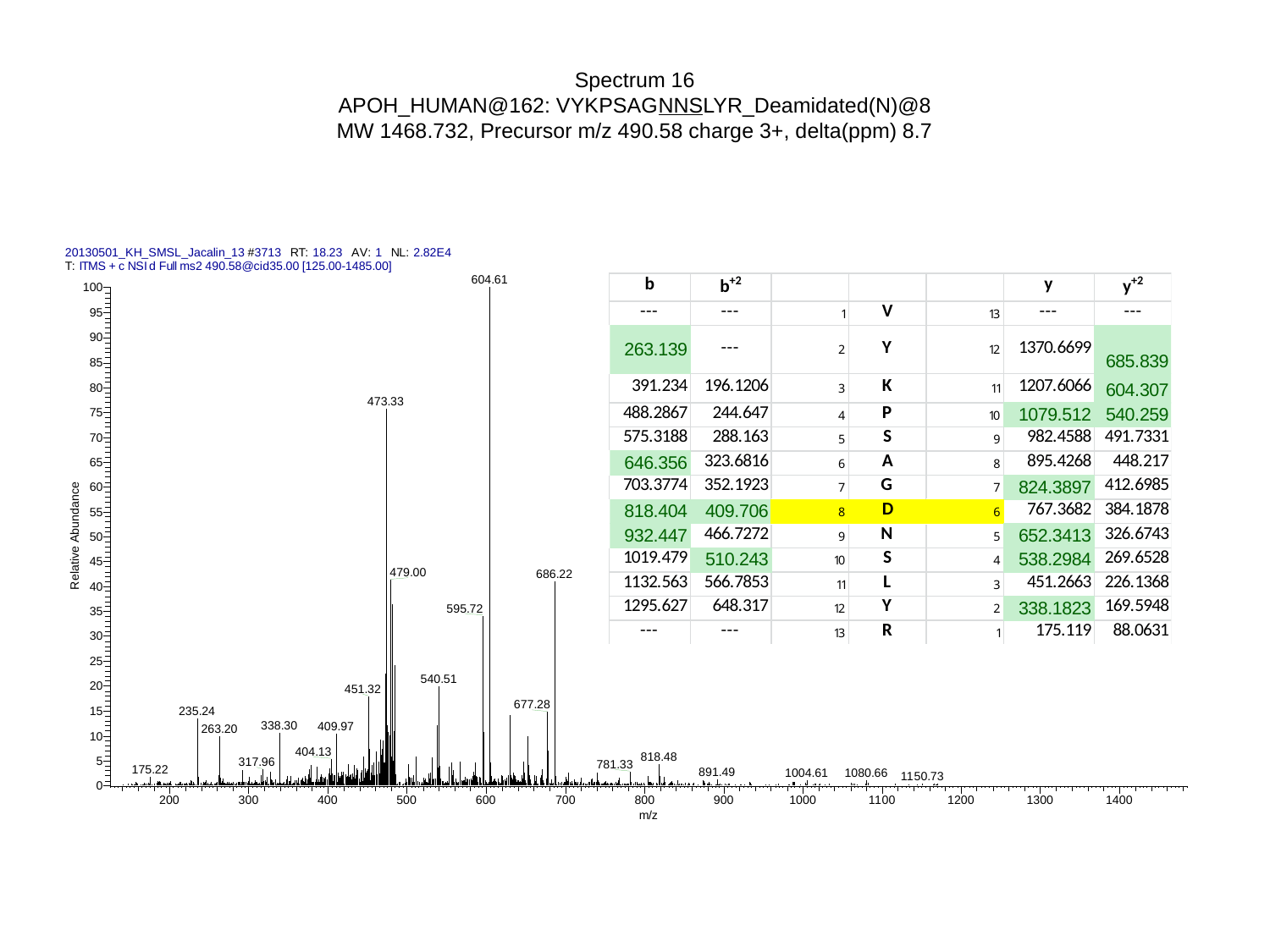

# Spectrum 16 APOH_HUMAN@162: VYKPSAGNNSLYR_Deamidated(N)@8 MW 1468.732, Precursor m/z 490.58 charge 3+, delta(ppm) 8.7

## Slide 20
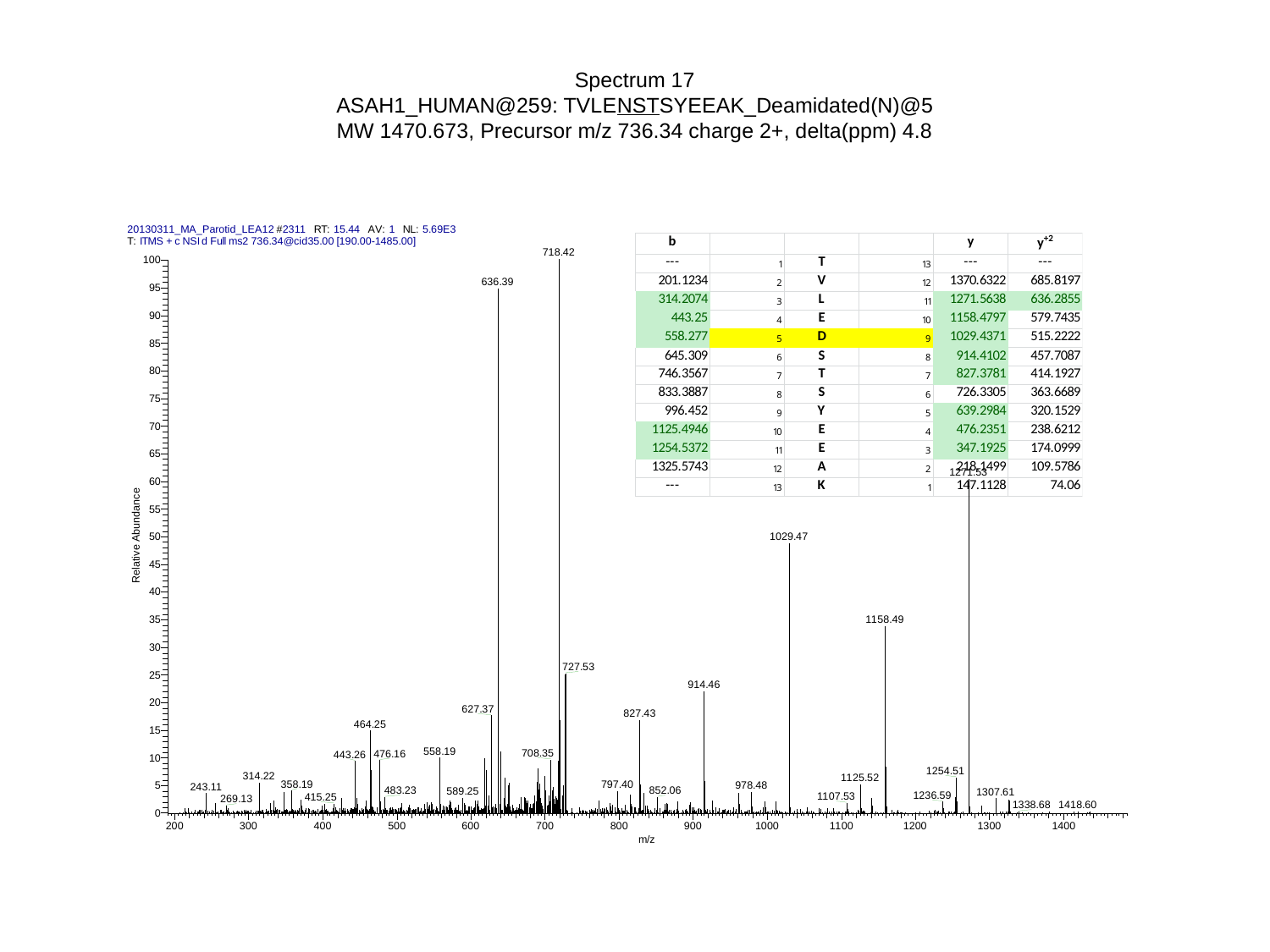

# Spectrum 17 ASAH1_HUMAN@259: TVLENSTSYEEAK_Deamidated(N)@5 MW 1470.673, Precursor m/z 736.34 charge 2+, delta(ppm) 4.8

## Slide 21
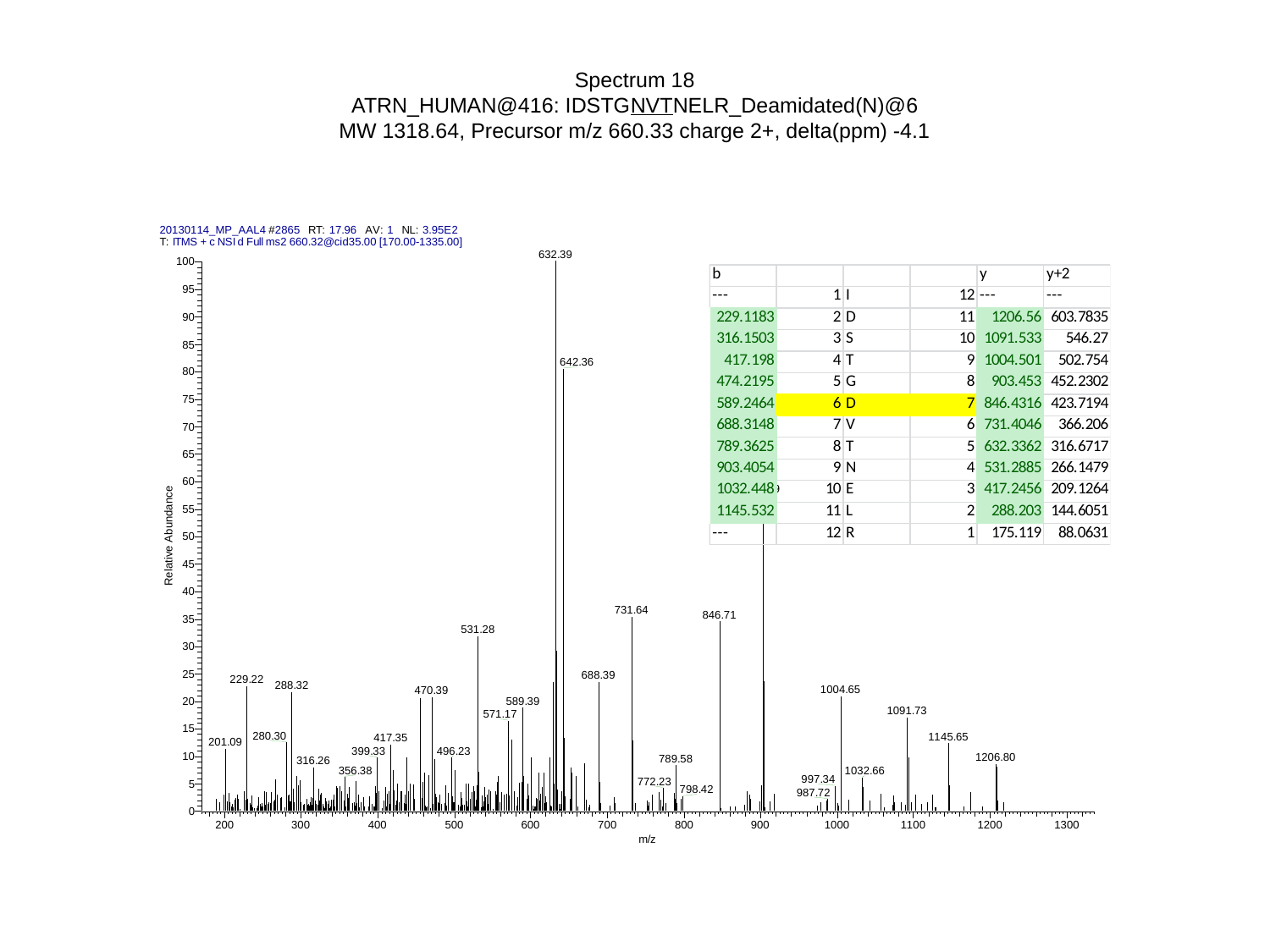

# Spectrum 18 ATRN_HUMAN@416: IDSTGNVTNELR_Deamidated(N)@6 MW 1318.64, Precursor m/z 660.33 charge 2+, delta(ppm) -4.1

## Slide 22
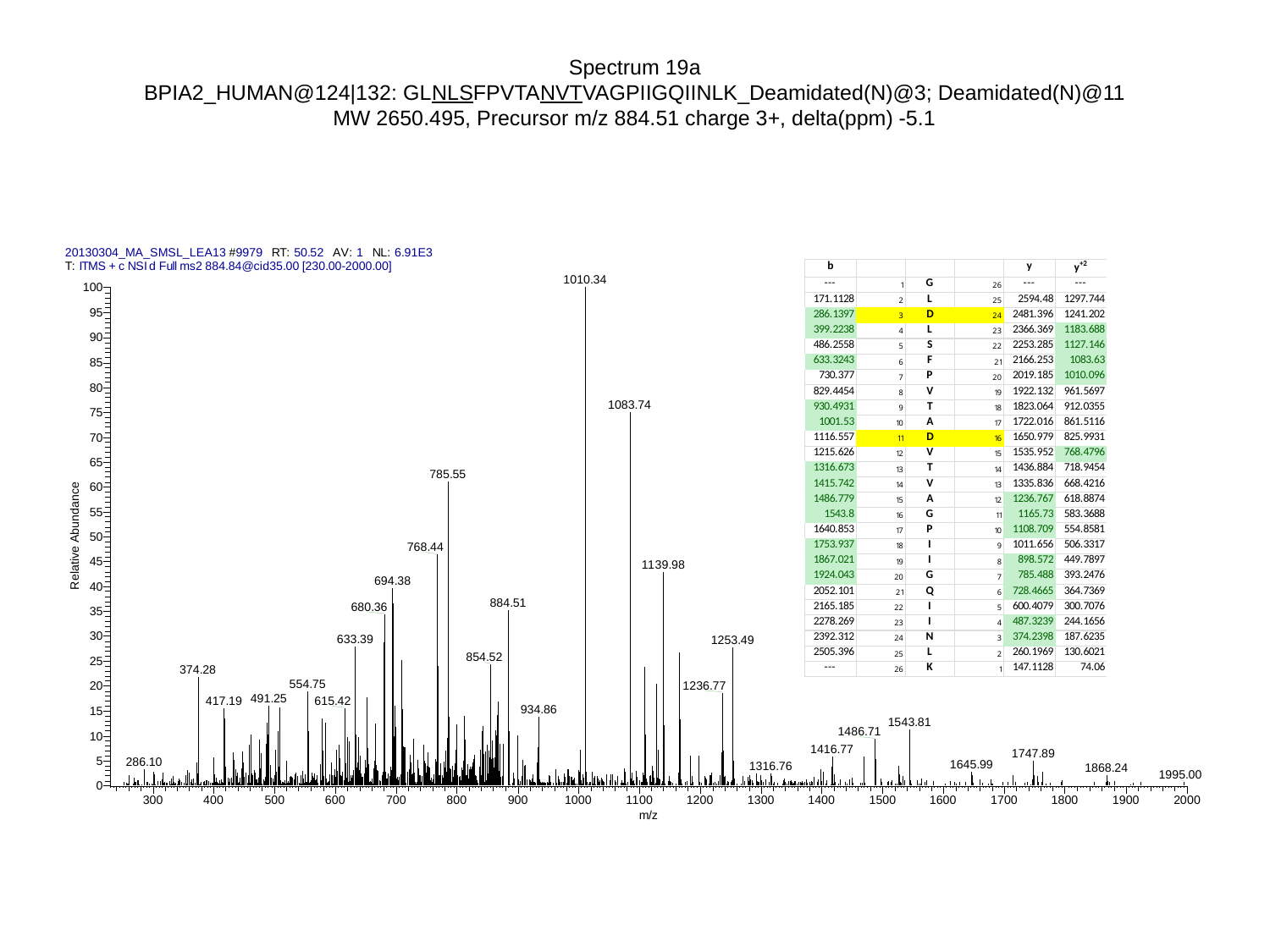

# Spectrum 19a BPIA2_HUMAN@124|132: GLNLSFPVTANVTVAGPIIGQIINLK_Deamidated(N)@3; Deamidated(N)@11 MW 2650.495, Precursor m/z 884.51 charge 3+, delta(ppm) -5.1

## Slide 23
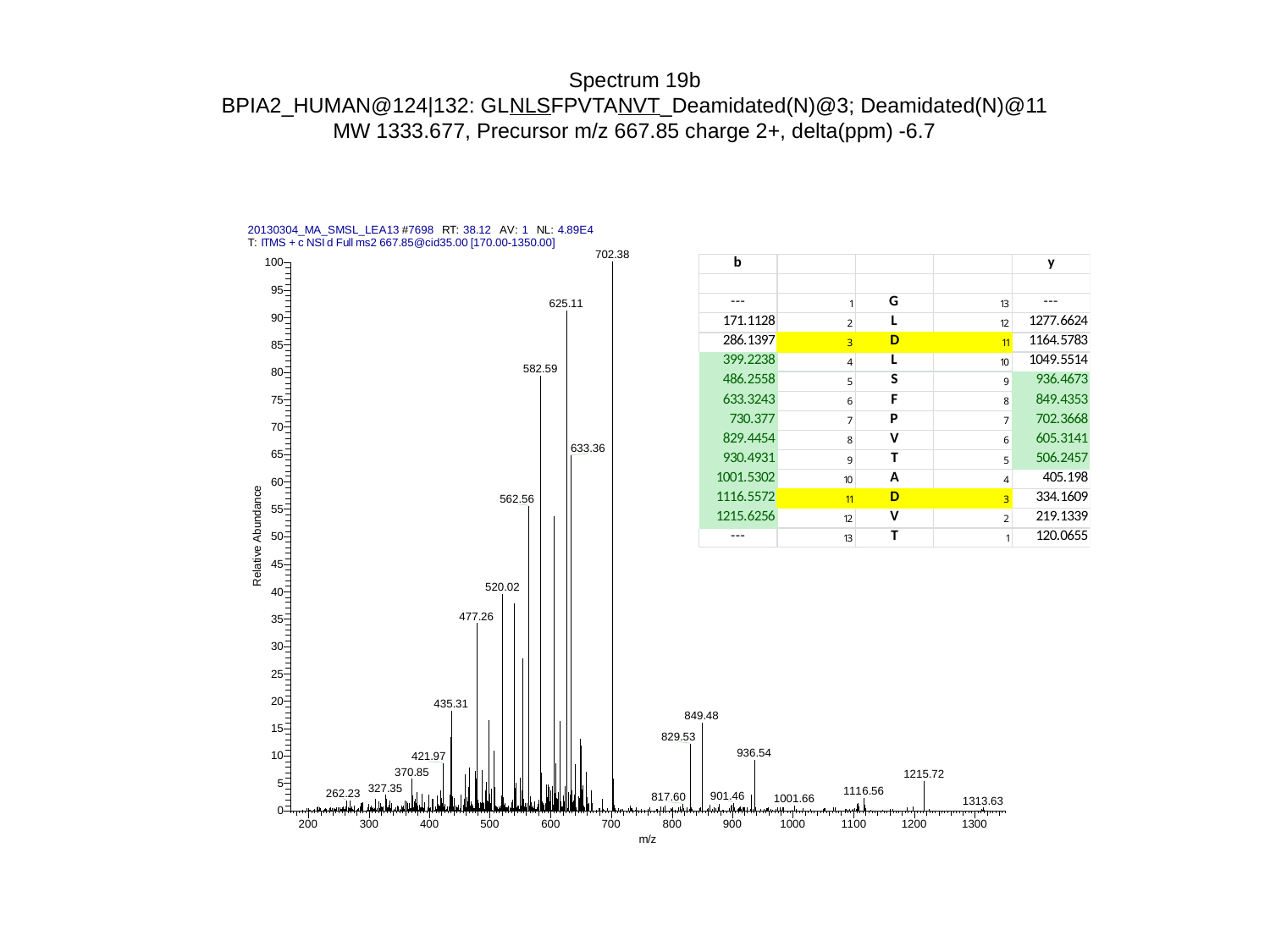

# Spectrum 19b BPIA2_HUMAN@124|132: GLNLSFPVTANVT_Deamidated(N)@3; Deamidated(N)@11 MW 1333.677, Precursor m/z 667.85 charge 2+, delta(ppm) -6.7

## Slide 24
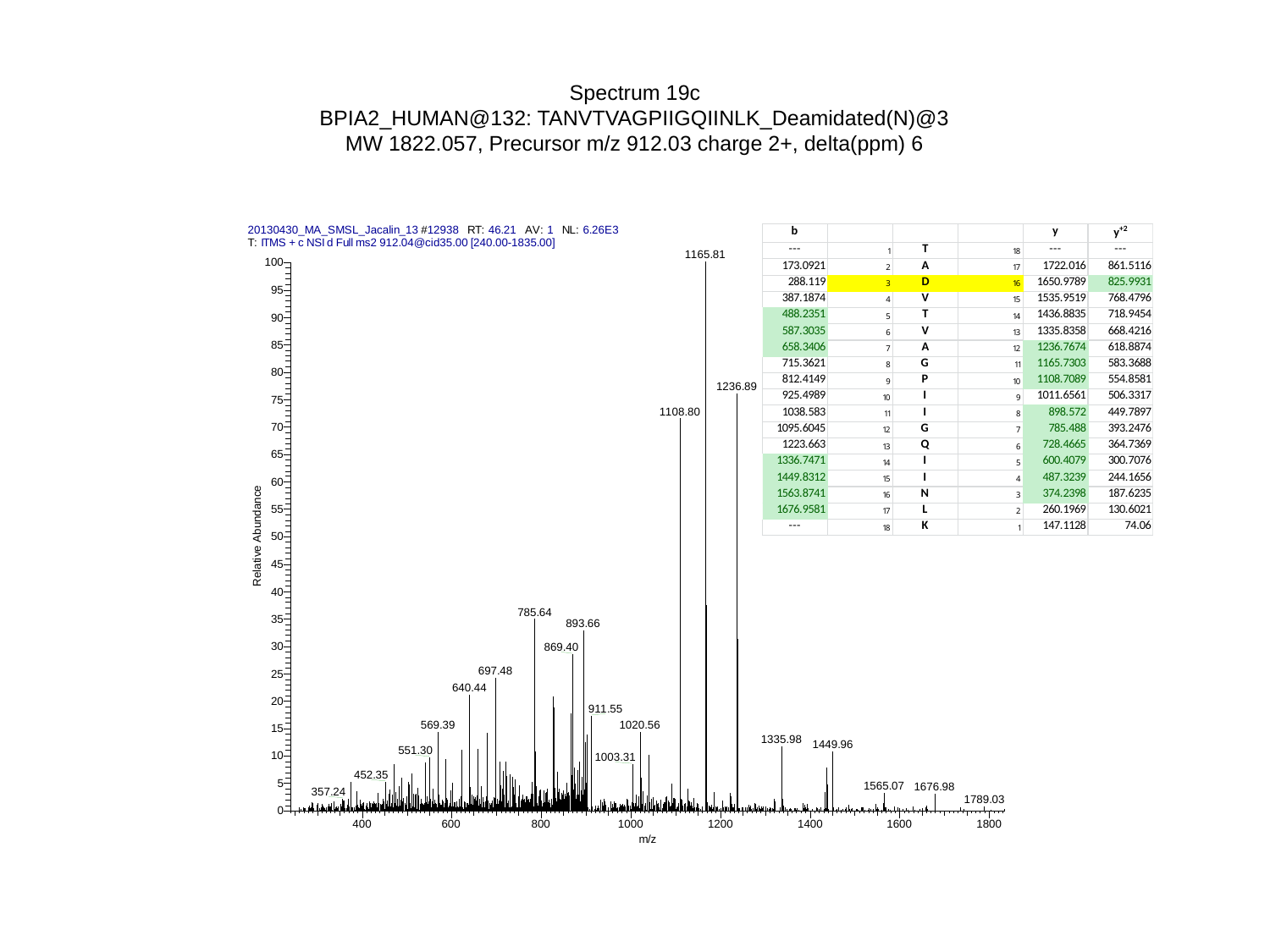

# Spectrum 19c BPIA2_HUMAN@132: TANVTVAGPIIGQIINLK_Deamidated(N)@3 MW 1822.057, Precursor m/z 912.03 charge 2+, delta(ppm) 6

## Slide 25
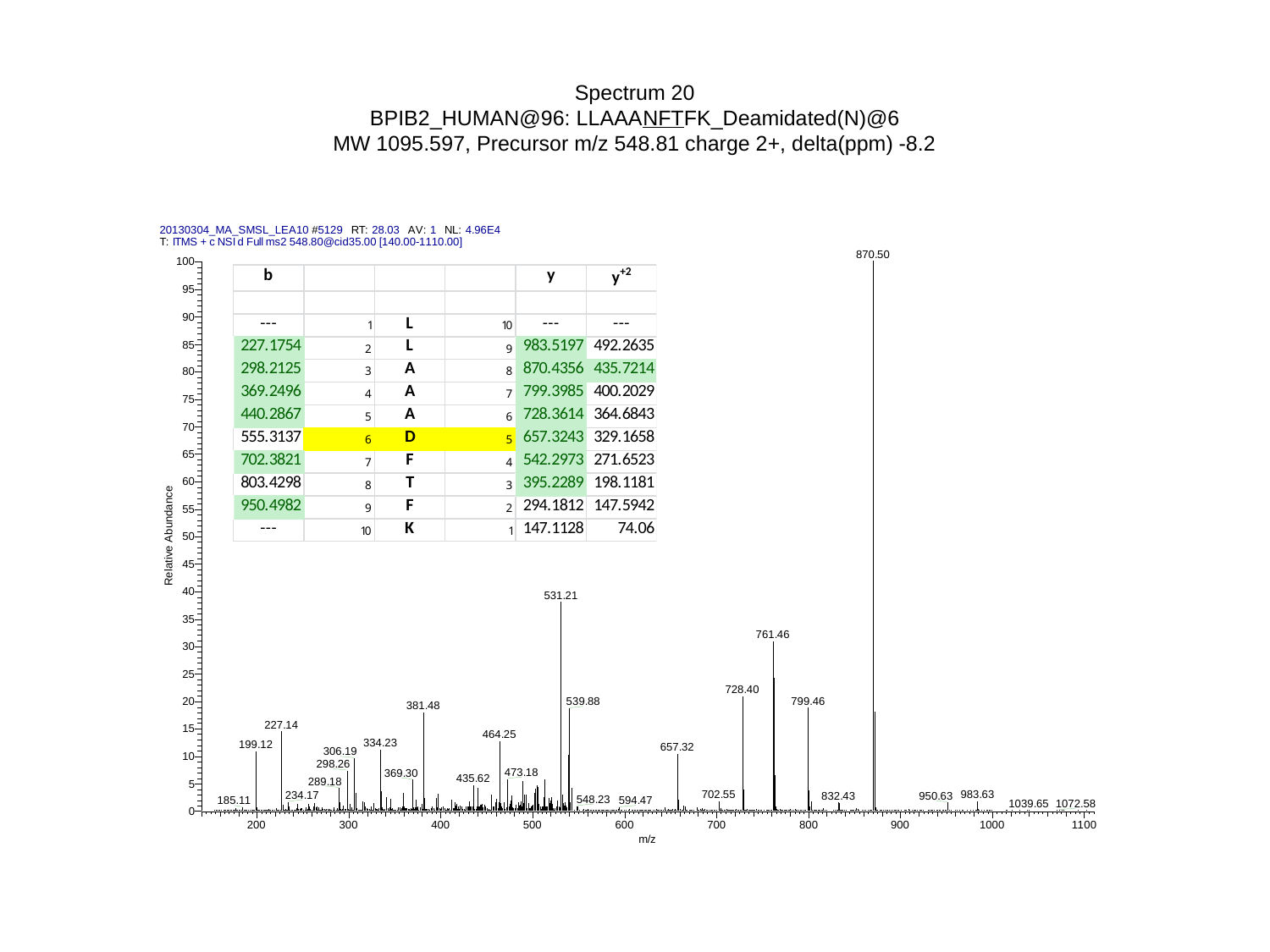

# Spectrum 20 BPIB2_HUMAN@96: LLAAANFTFK_Deamidated(N)@6 MW 1095.597, Precursor m/z 548.81 charge 2+, delta(ppm) -8.2

## Slide 26
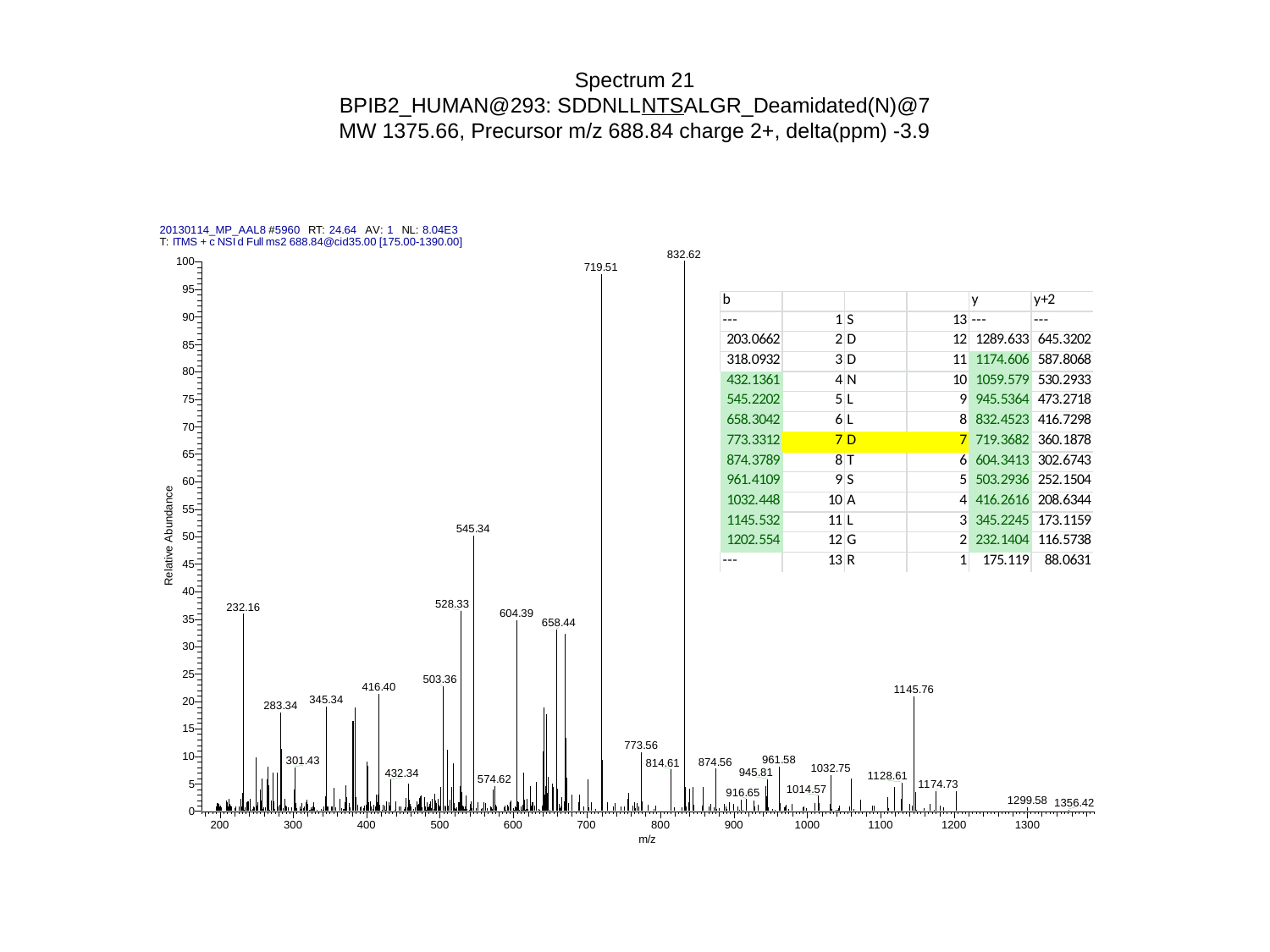

# Spectrum 21 BPIB2_HUMAN@293: SDDNLLNTSALGR_Deamidated(N)@7 MW 1375.66, Precursor m/z 688.84 charge 2+, delta(ppm) -3.9

## Slide 27
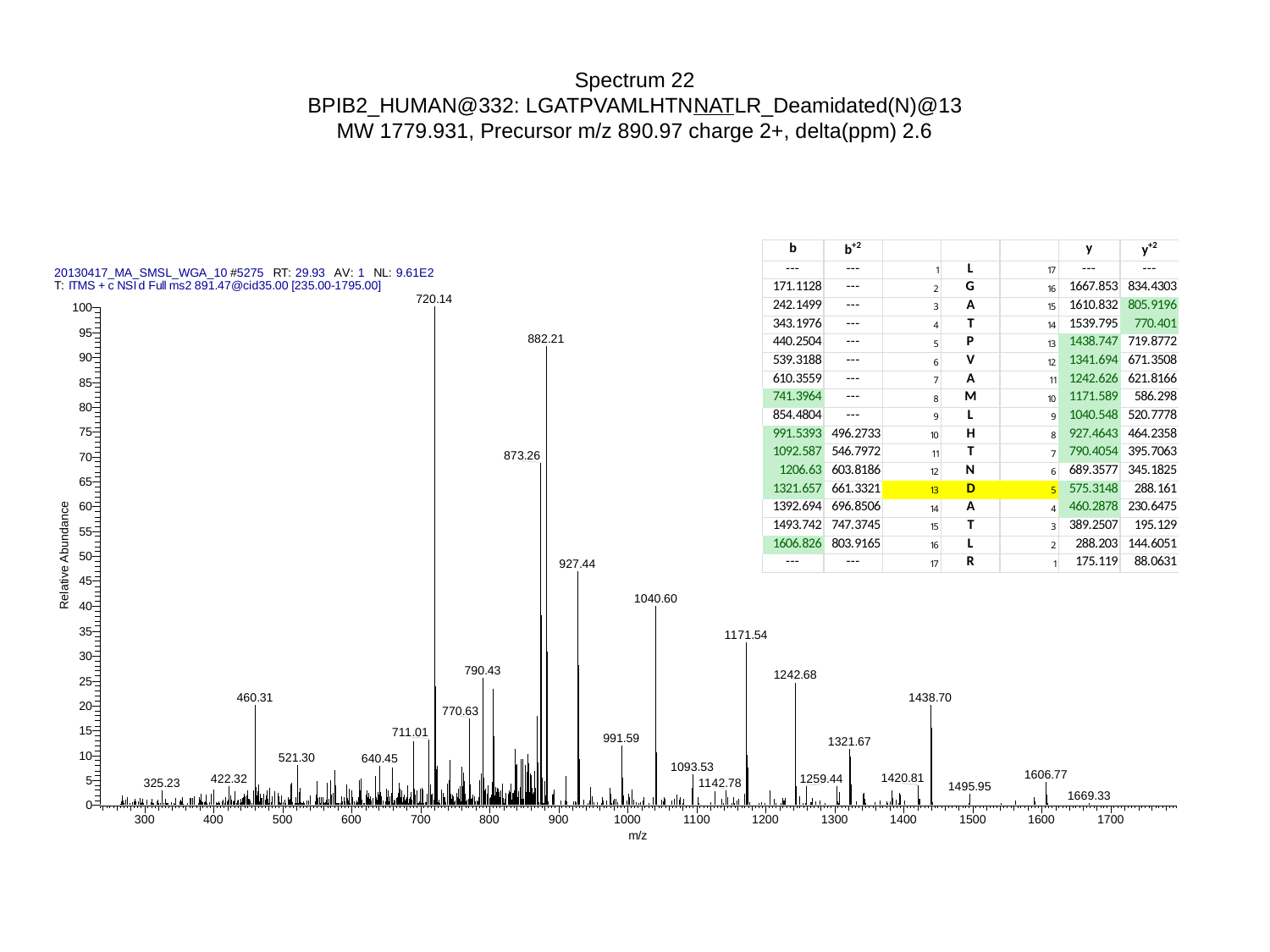

# Spectrum 22 BPIB2_HUMAN@332: LGATPVAMLHTNNATLR_Deamidated(N)@13 MW 1779.931, Precursor m/z 890.97 charge 2+, delta(ppm) 2.6

## Slide 28
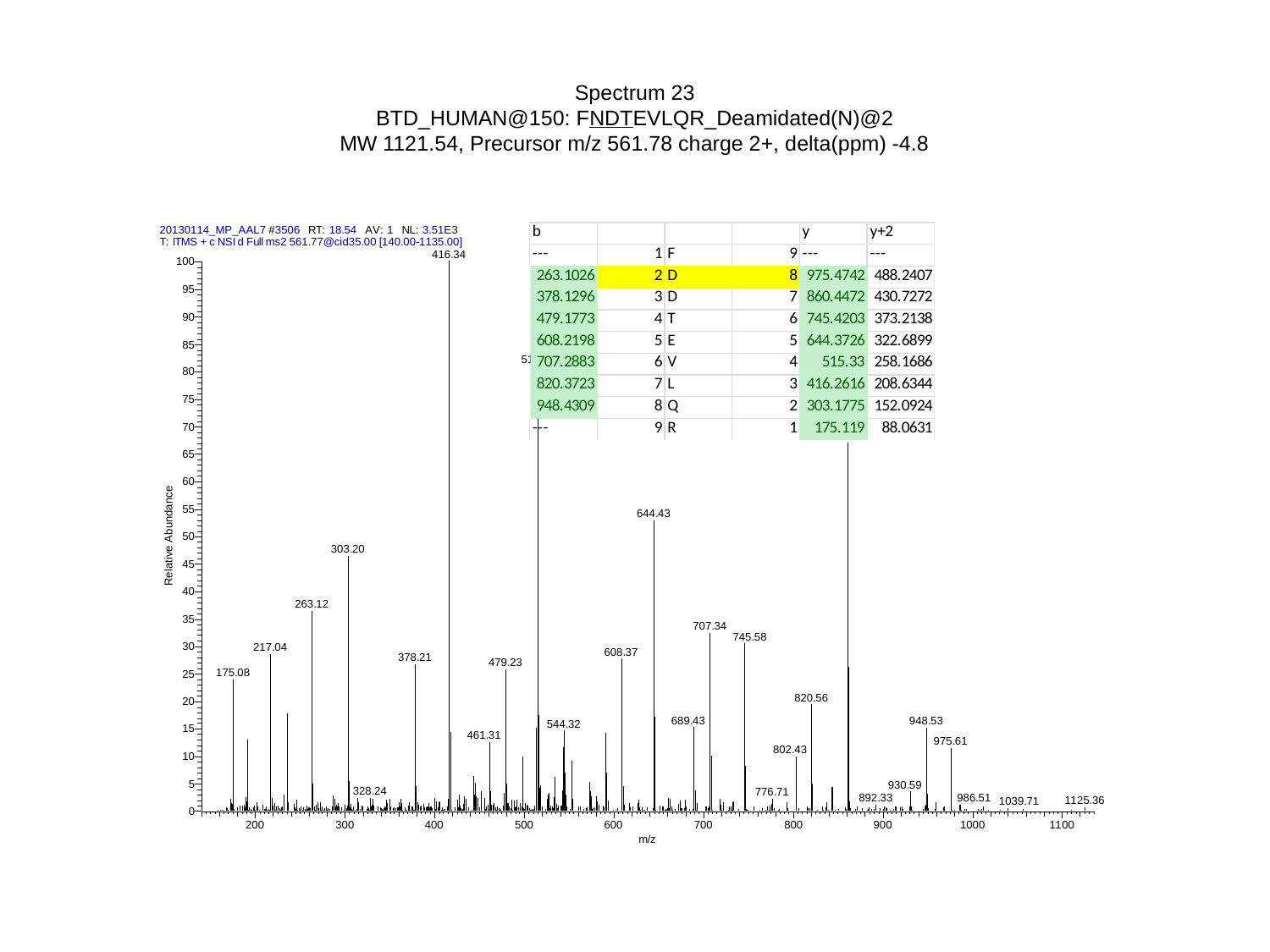

# Spectrum 23 BTD_HUMAN@150: FNDTEVLQR_Deamidated(N)@2 MW 1121.54, Precursor m/z 561.78 charge 2+, delta(ppm) -4.8

## Slide 29
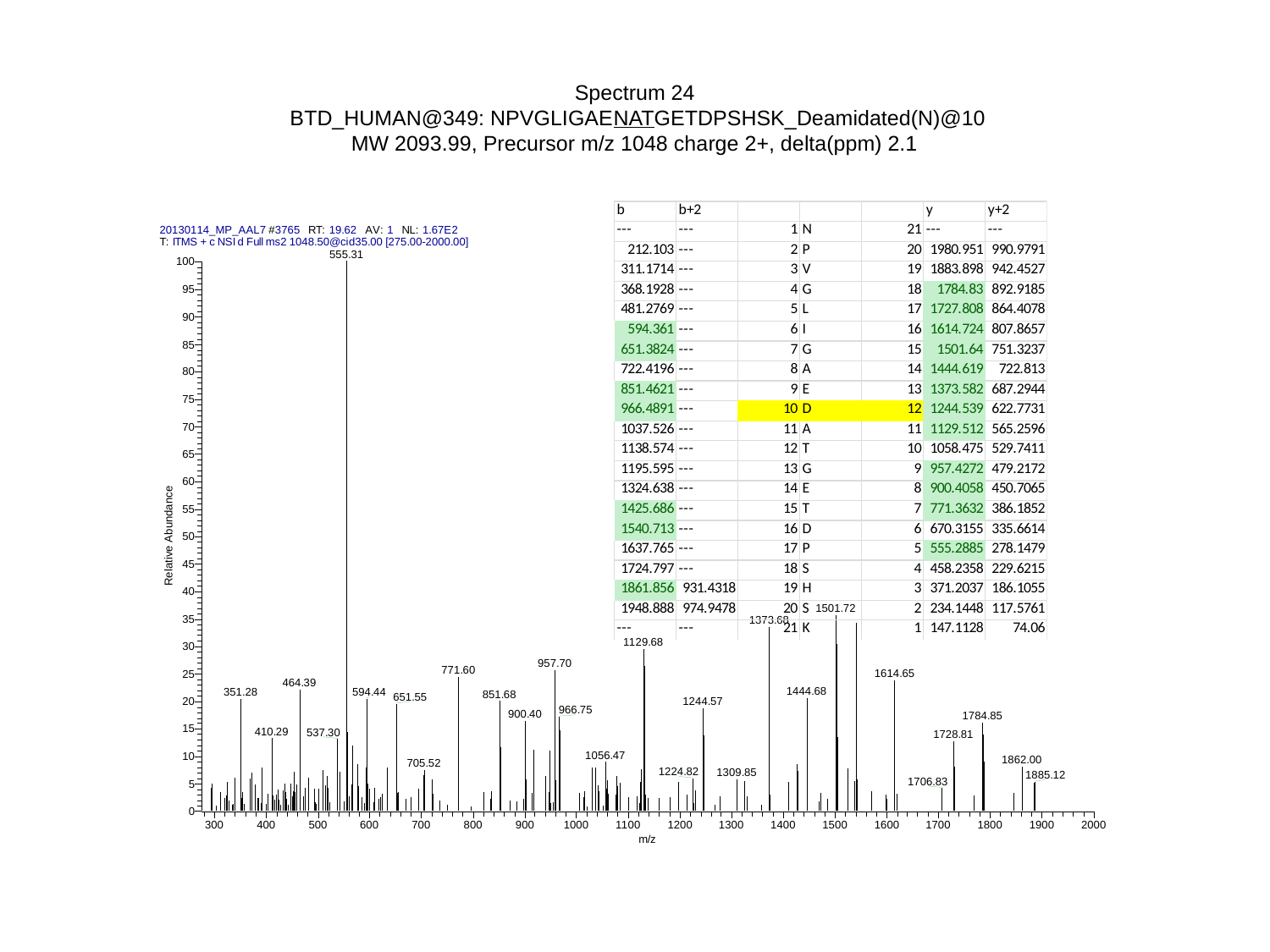

# Spectrum 24 BTD_HUMAN@349: NPVGLIGAENATGETDPSHSK_Deamidated(N)@10 MW 2093.99, Precursor m/z 1048 charge 2+, delta(ppm) 2.1

## Slide 30
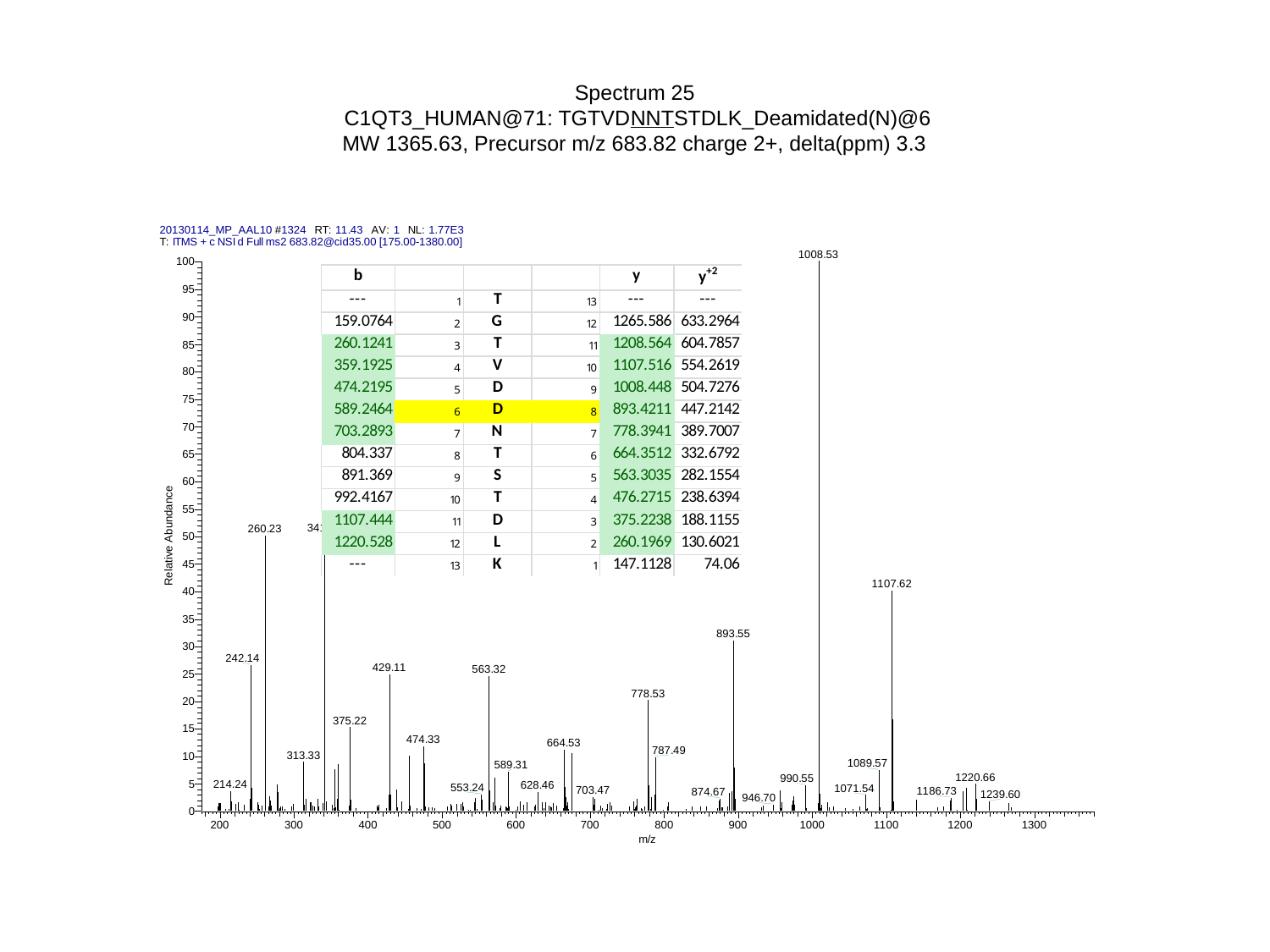

# Spectrum 25 C1QT3_HUMAN@71: TGTVDNNTSTDLK_Deamidated(N)@6 MW 1365.63, Precursor m/z 683.82 charge 2+, delta(ppm) 3.3

## Slide 31
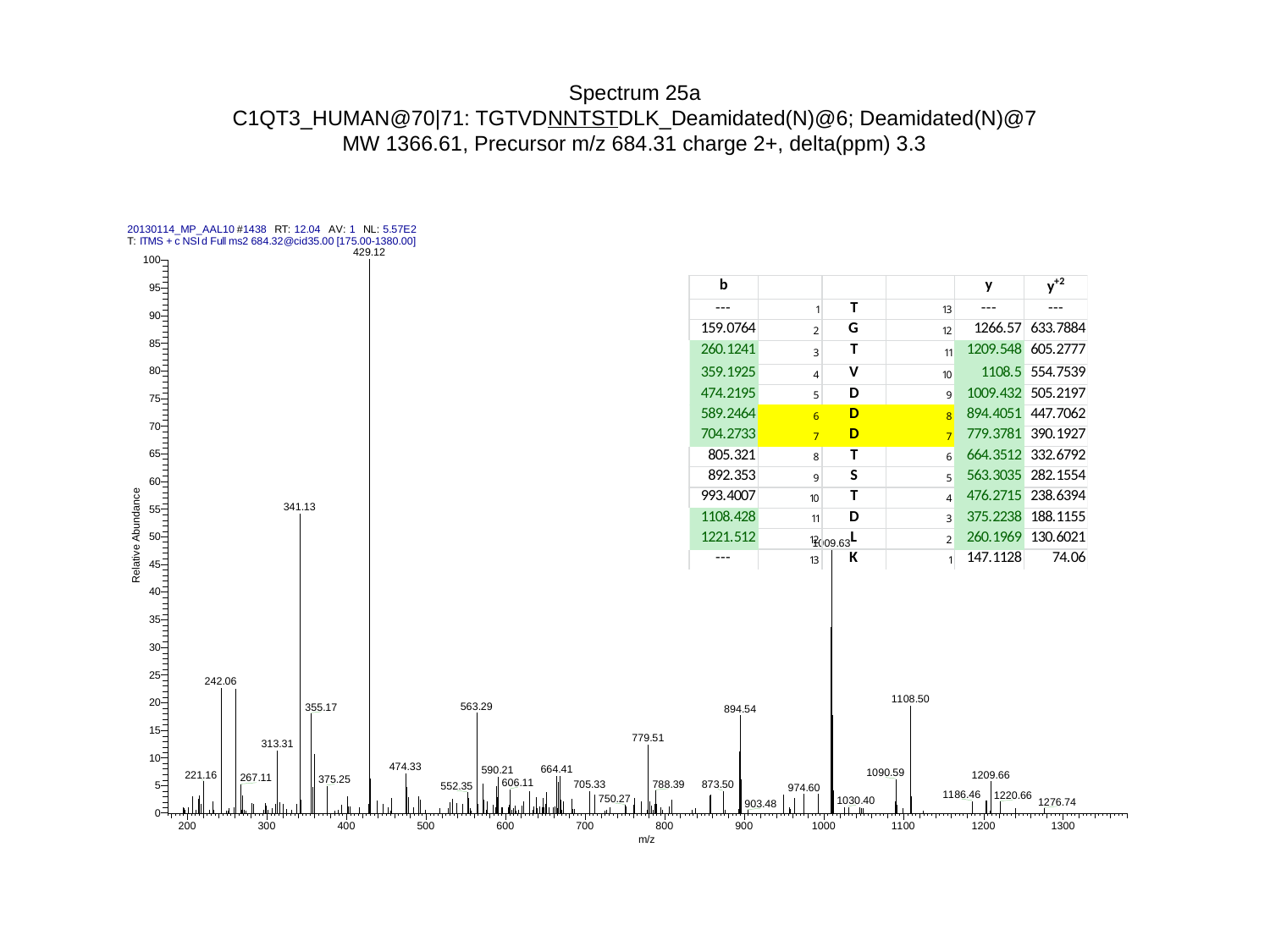

# Spectrum 25a C1QT3_HUMAN@70|71: TGTVDNNTSTDLK_Deamidated(N)@6; Deamidated(N)@7 MW 1366.61, Precursor m/z 684.31 charge 2+, delta(ppm) 3.3

## Slide 32
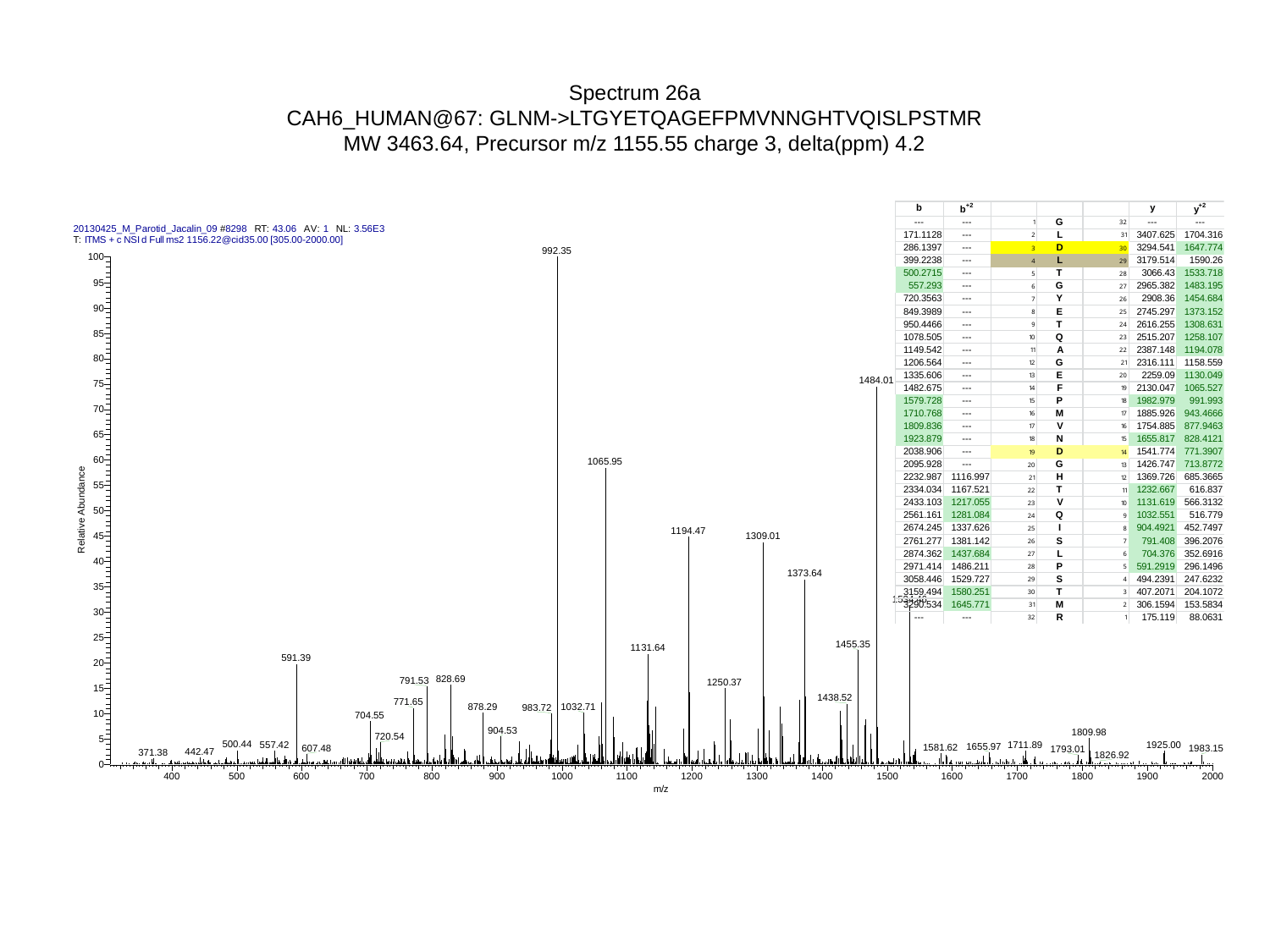

# Spectrum 26a CAH6_HUMAN@67: GLNM->LTGYETQAGEFPMVNNGHTVQISLPSTMR MW 3463.64, Precursor m/z 1155.55 charge 3, delta(ppm) 4.2

## Slide 33
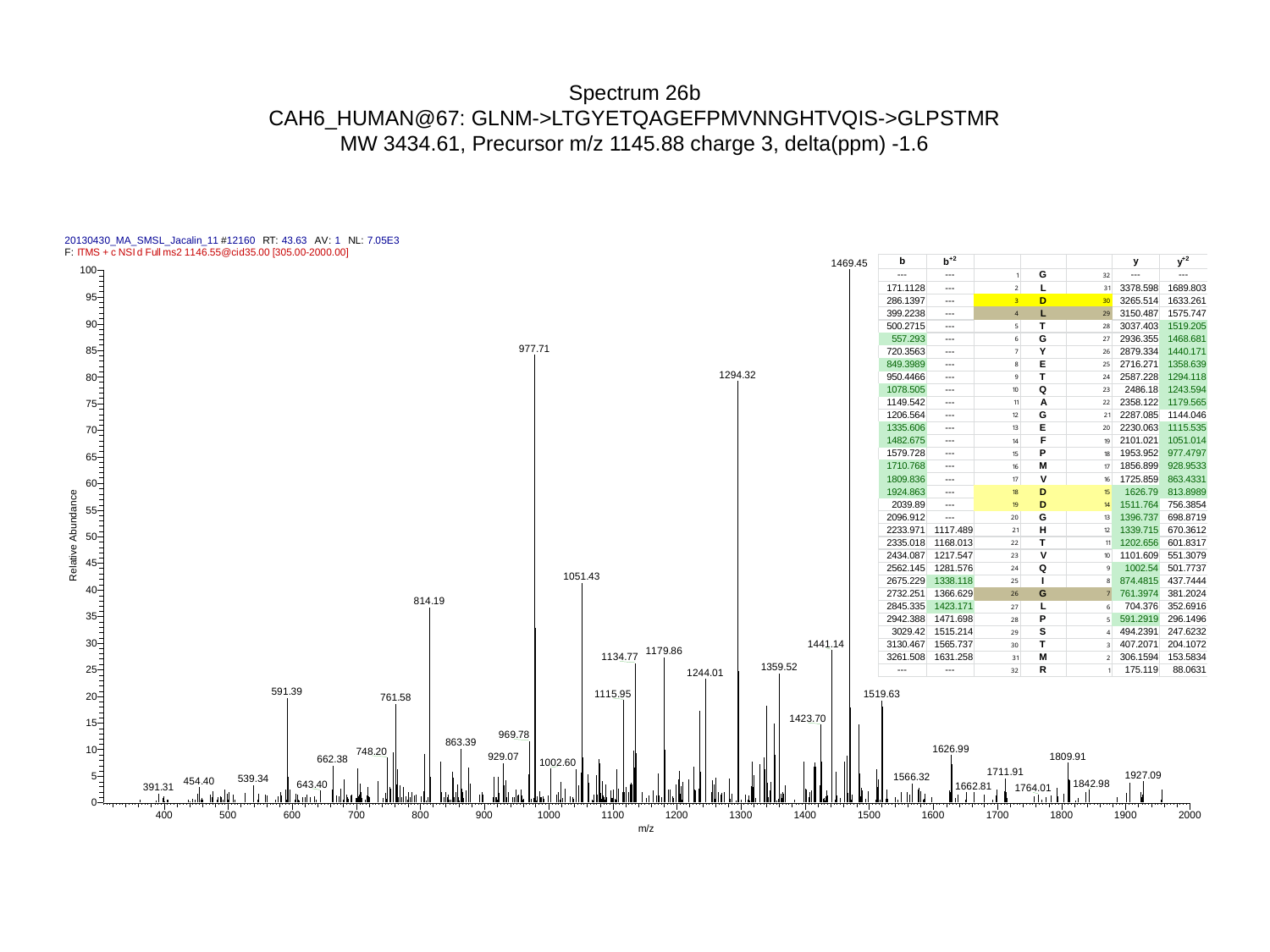

# Spectrum 26b CAH6_HUMAN@67: GLNM->LTGYETQAGEFPMVNNGHTVQIS->GLPSTMR MW 3434.61, Precursor m/z 1145.88 charge 3, delta(ppm) -1.6

## Slide 34
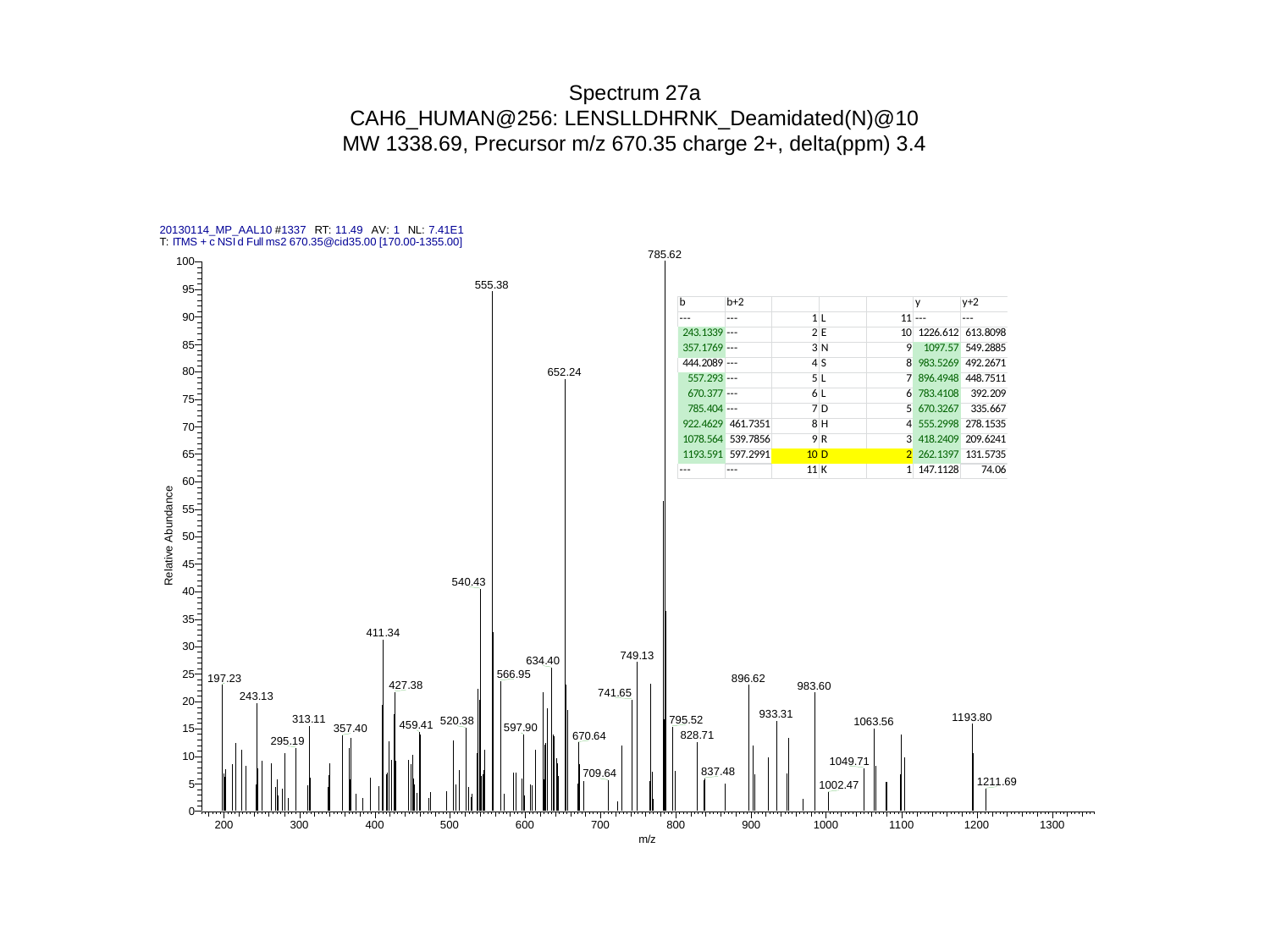

# Spectrum 27a CAH6_HUMAN@256: LENSLLDHRNK_Deamidated(N)@10 MW 1338.69, Precursor m/z 670.35 charge 2+, delta(ppm) 3.4

## Slide 35
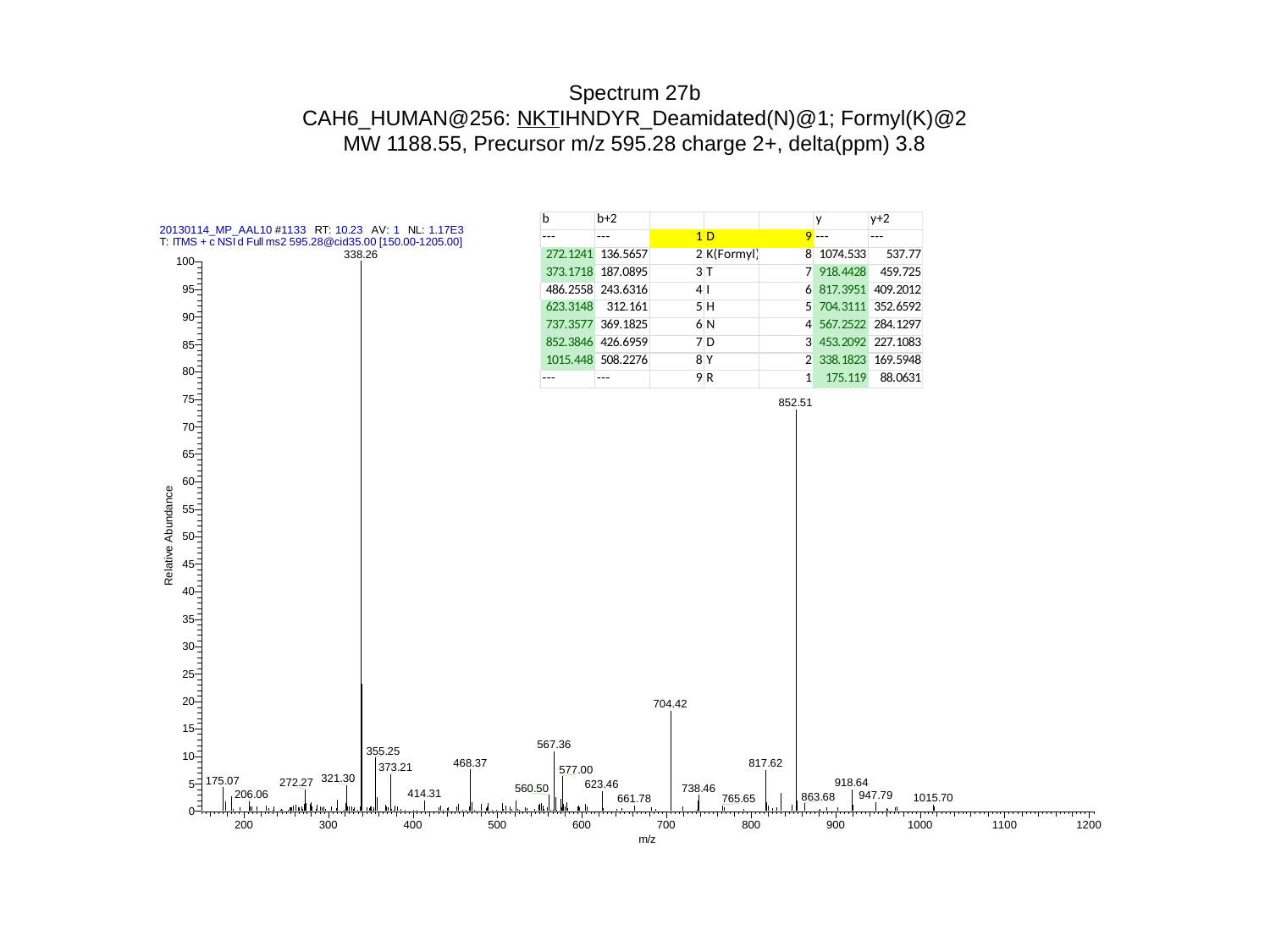

# Spectrum 27b CAH6_HUMAN@256: NKTIHNDYR_Deamidated(N)@1; Formyl(K)@2 MW 1188.55, Precursor m/z 595.28 charge 2+, delta(ppm) 3.8

## Slide 36
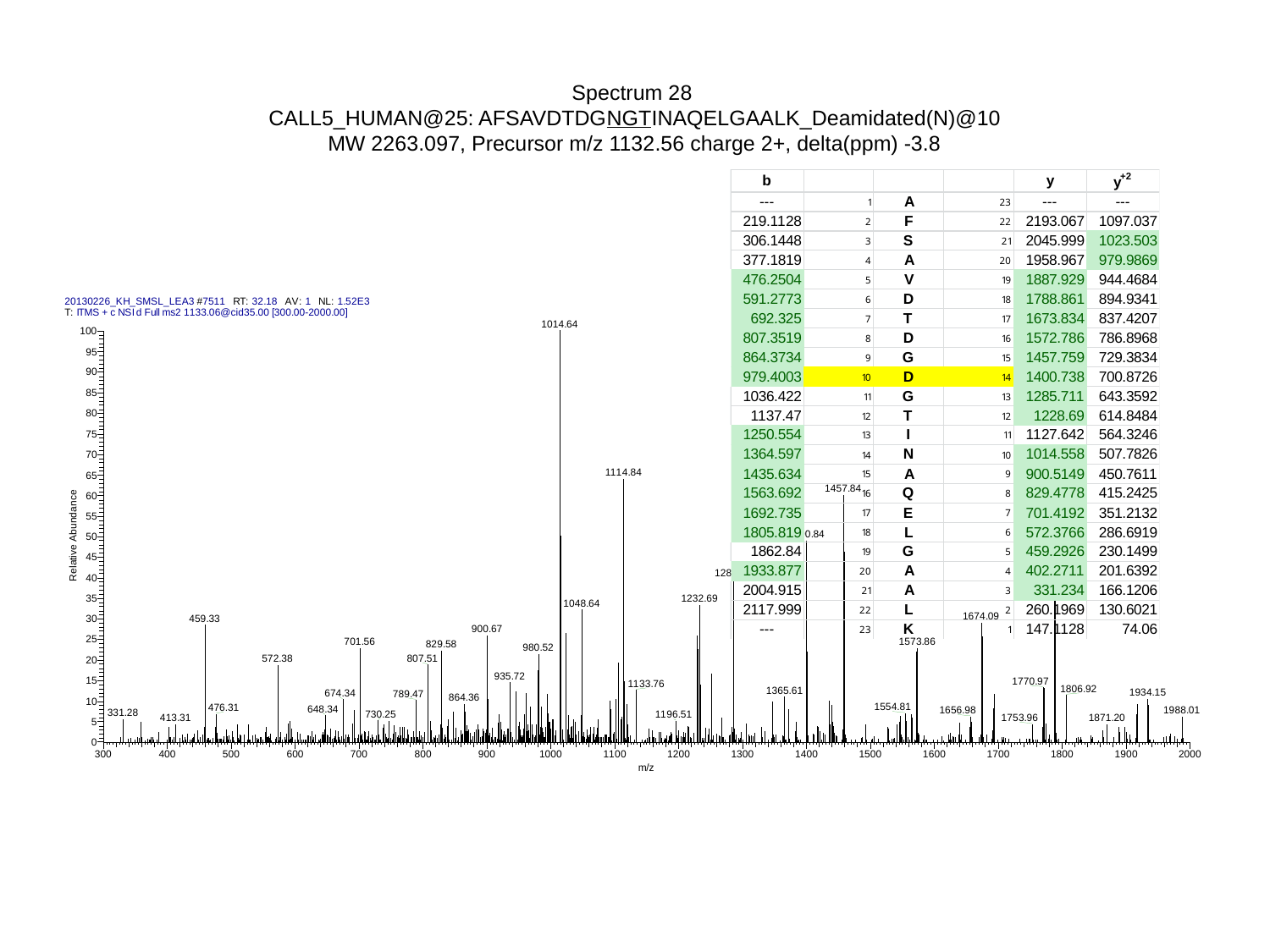

# Spectrum 28 CALL5_HUMAN@25: AFSAVDTDGNGTINAQELGAALK_Deamidated(N)@10 MW 2263.097, Precursor m/z 1132.56 charge 2+, delta(ppm) -3.8

## Slide 37
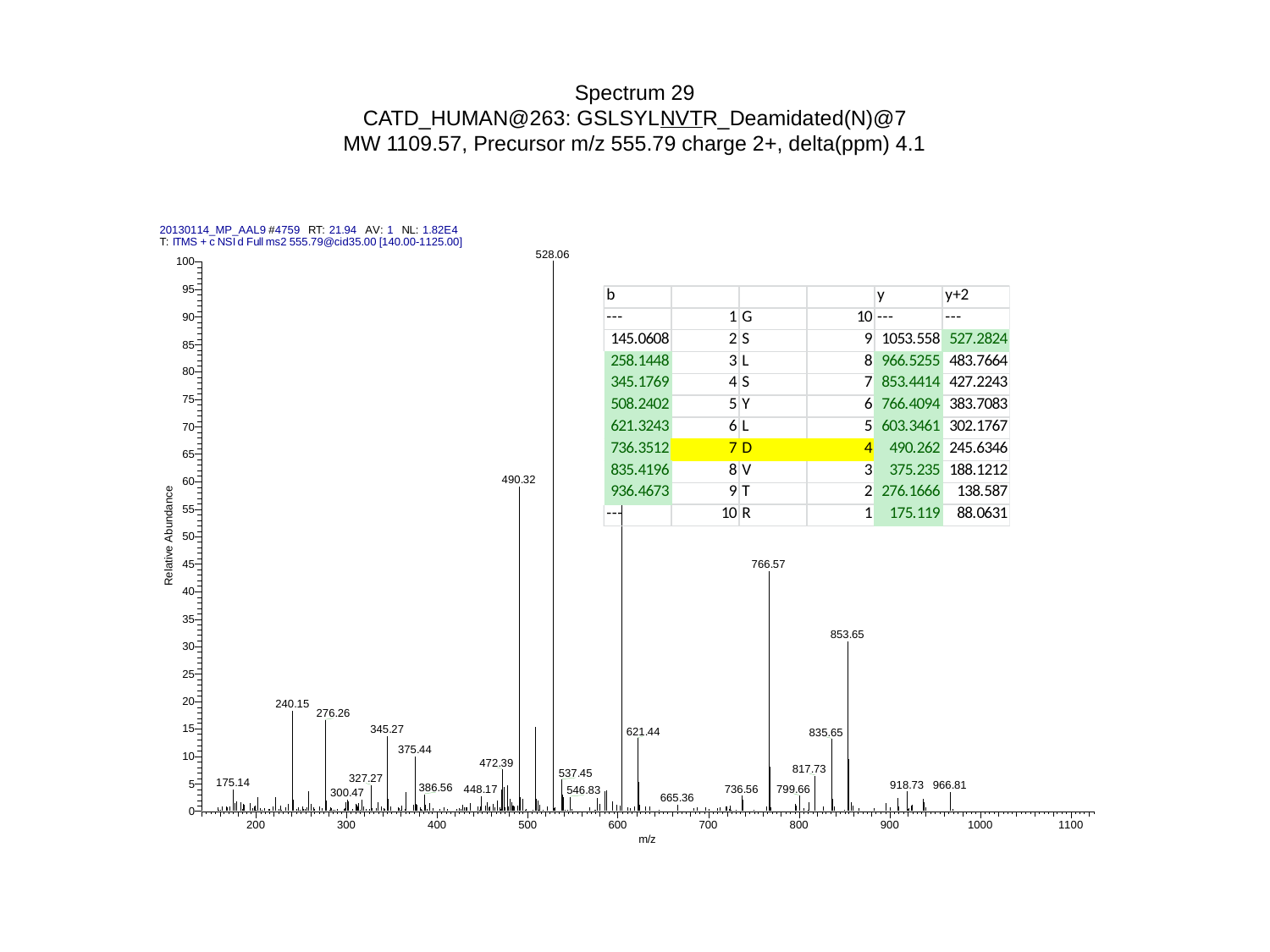

# Spectrum 29 CATD_HUMAN@263: GSLSYLNVTR_Deamidated(N)@7 MW 1109.57, Precursor m/z 555.79 charge 2+, delta(ppm) 4.1

## Slide 38
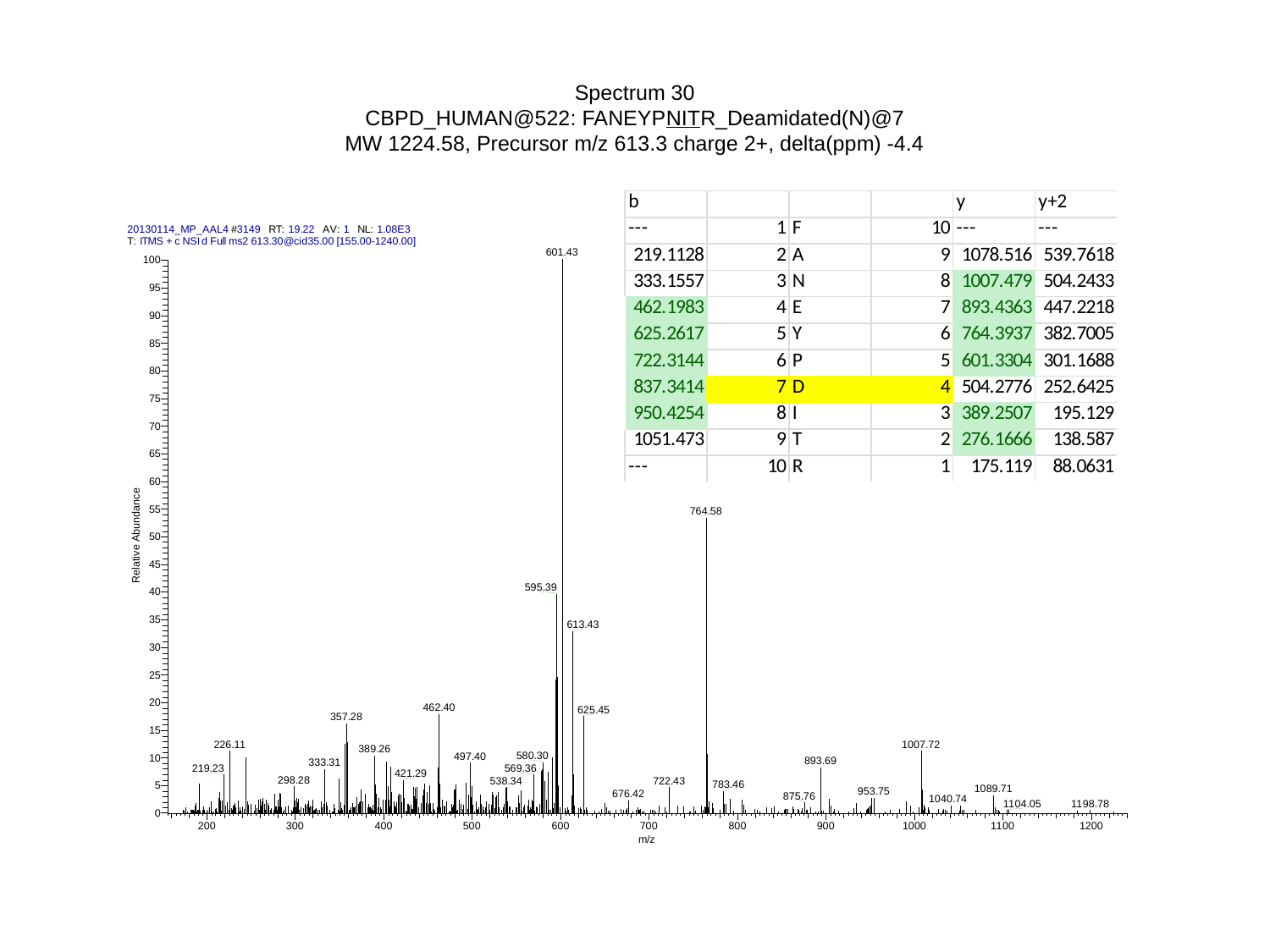

# Spectrum 30 CBPD_HUMAN@522: FANEYPNITR_Deamidated(N)@7 MW 1224.58, Precursor m/z 613.3 charge 2+, delta(ppm) -4.4

## Slide 39
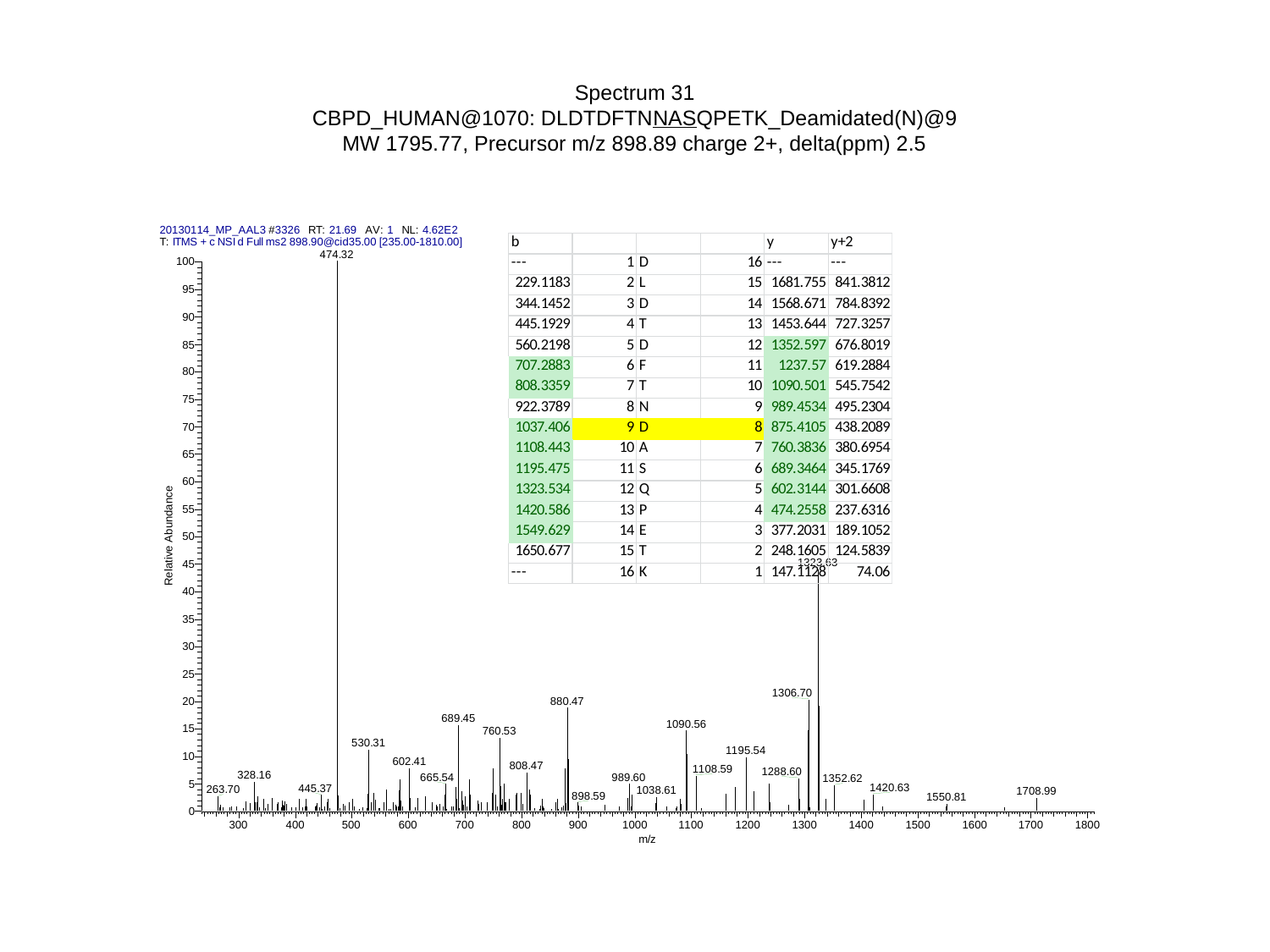

# Spectrum 31 CBPD_HUMAN@1070: DLDTDFTNNASQPETK_Deamidated(N)@9 MW 1795.77, Precursor m/z 898.89 charge 2+, delta(ppm) 2.5

## Slide 40
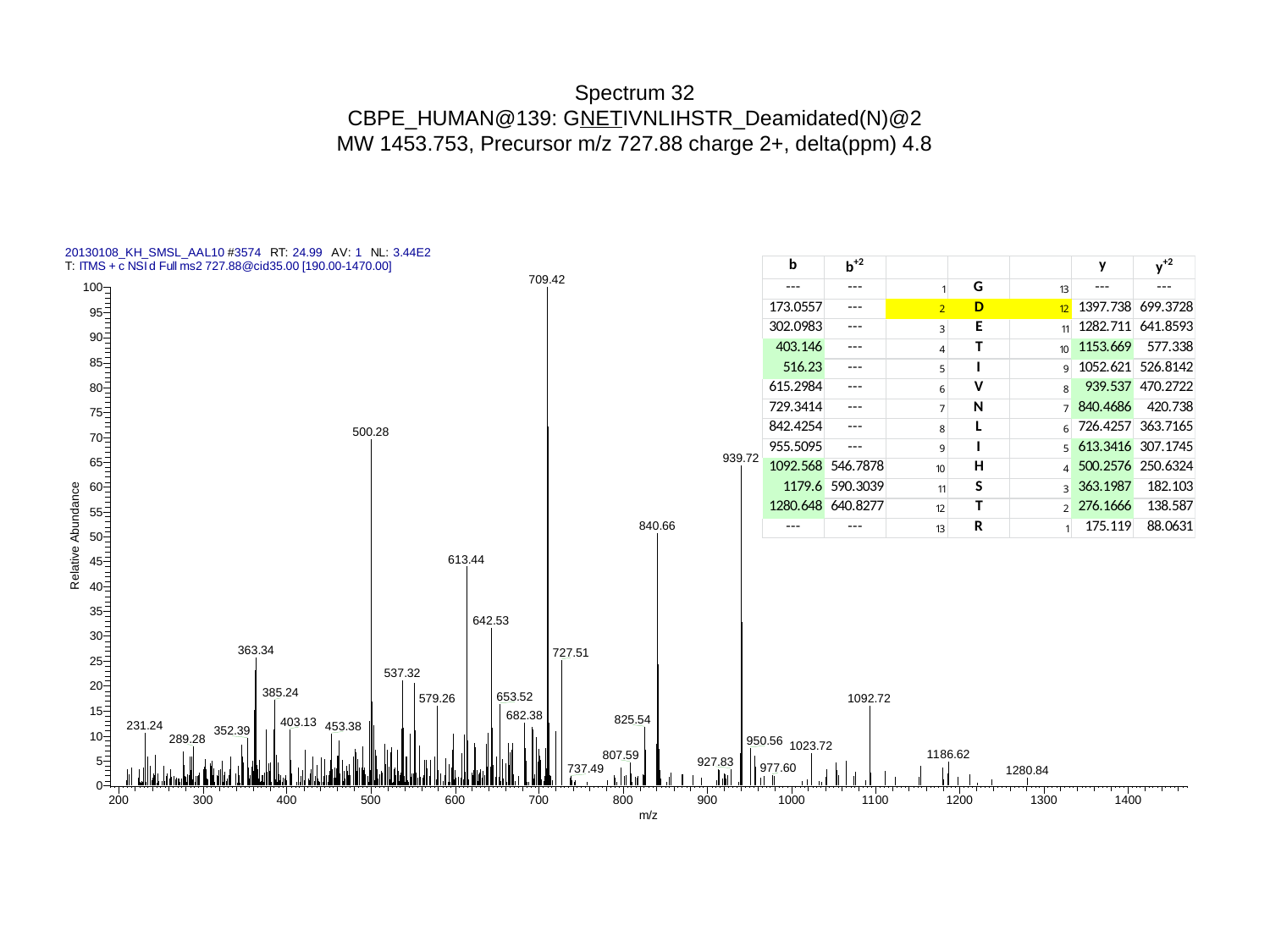

# Spectrum 32 CBPE_HUMAN@139: GNETIVNLIHSTR_Deamidated(N)@2 MW 1453.753, Precursor m/z 727.88 charge 2+, delta(ppm) 4.8

## Slide 41
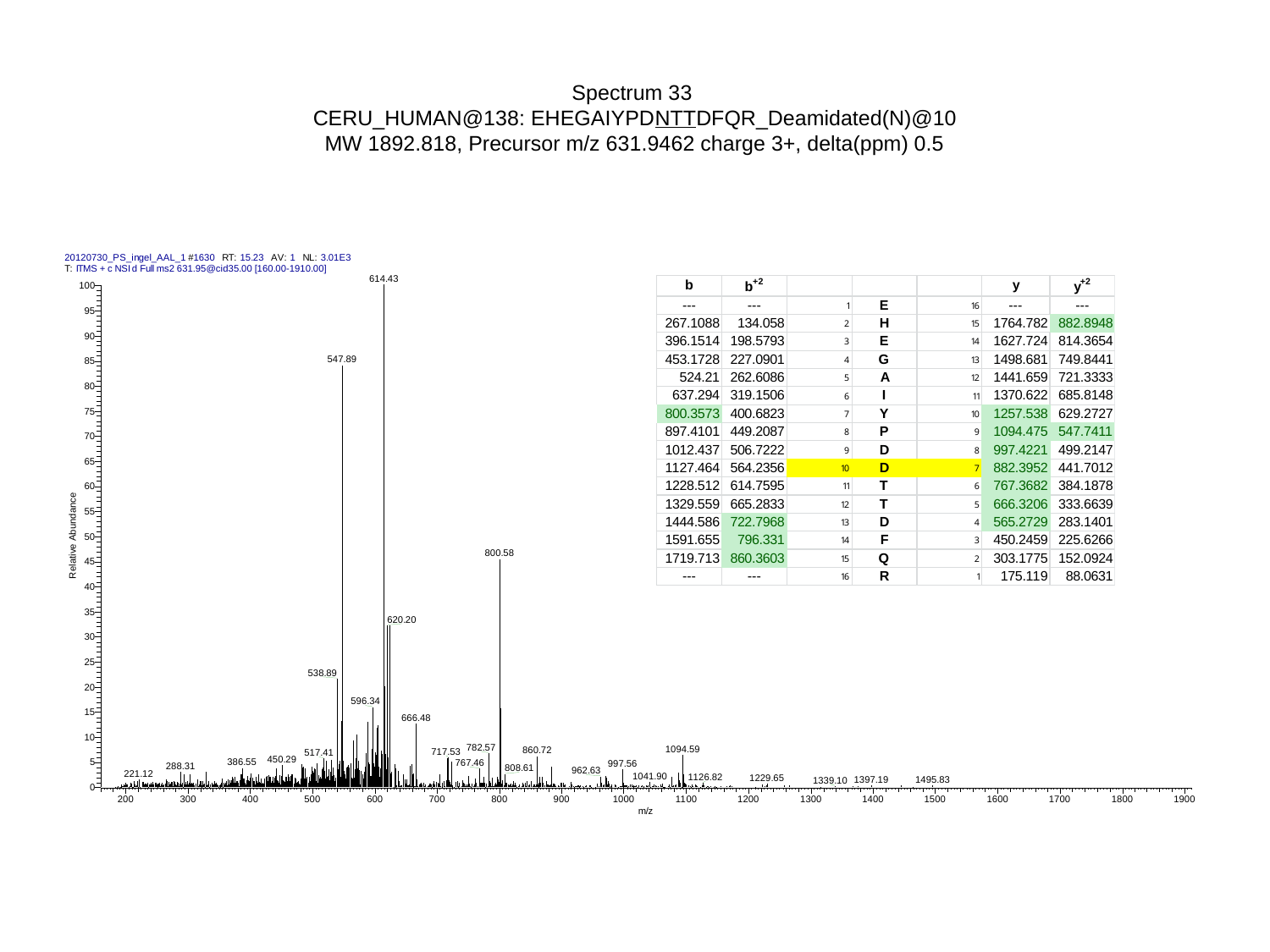

# Spectrum 33 CERU_HUMAN@138: EHEGAIYPDNTTDFQR_Deamidated(N)@10 MW 1892.818, Precursor m/z 631.9462 charge 3+, delta(ppm) 0.5

## Slide 42
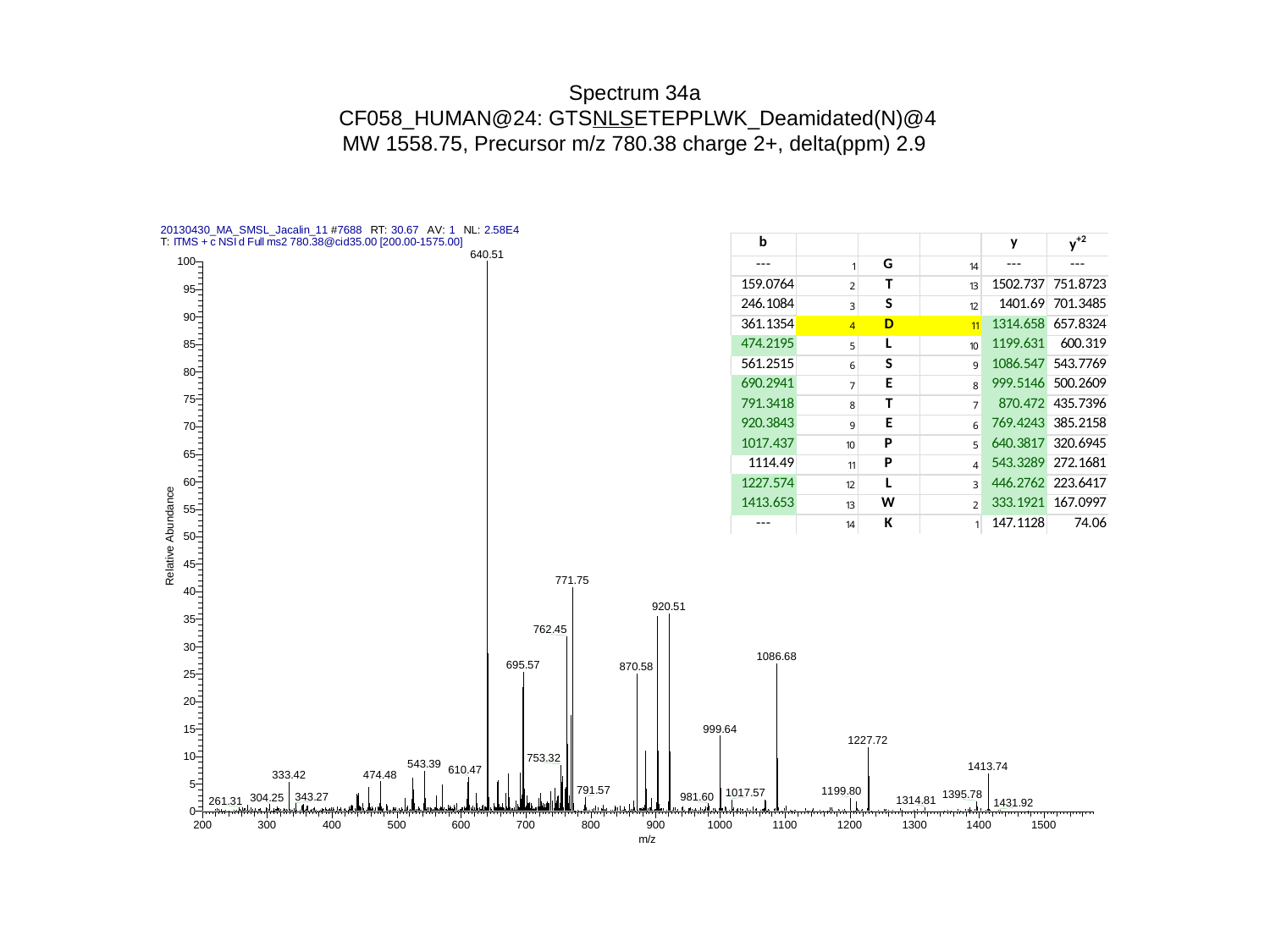

# Spectrum 34a CF058_HUMAN@24: GTSNLSETEPPLWK_Deamidated(N)@4 MW 1558.75, Precursor m/z 780.38 charge 2+, delta(ppm) 2.9

## Slide 43
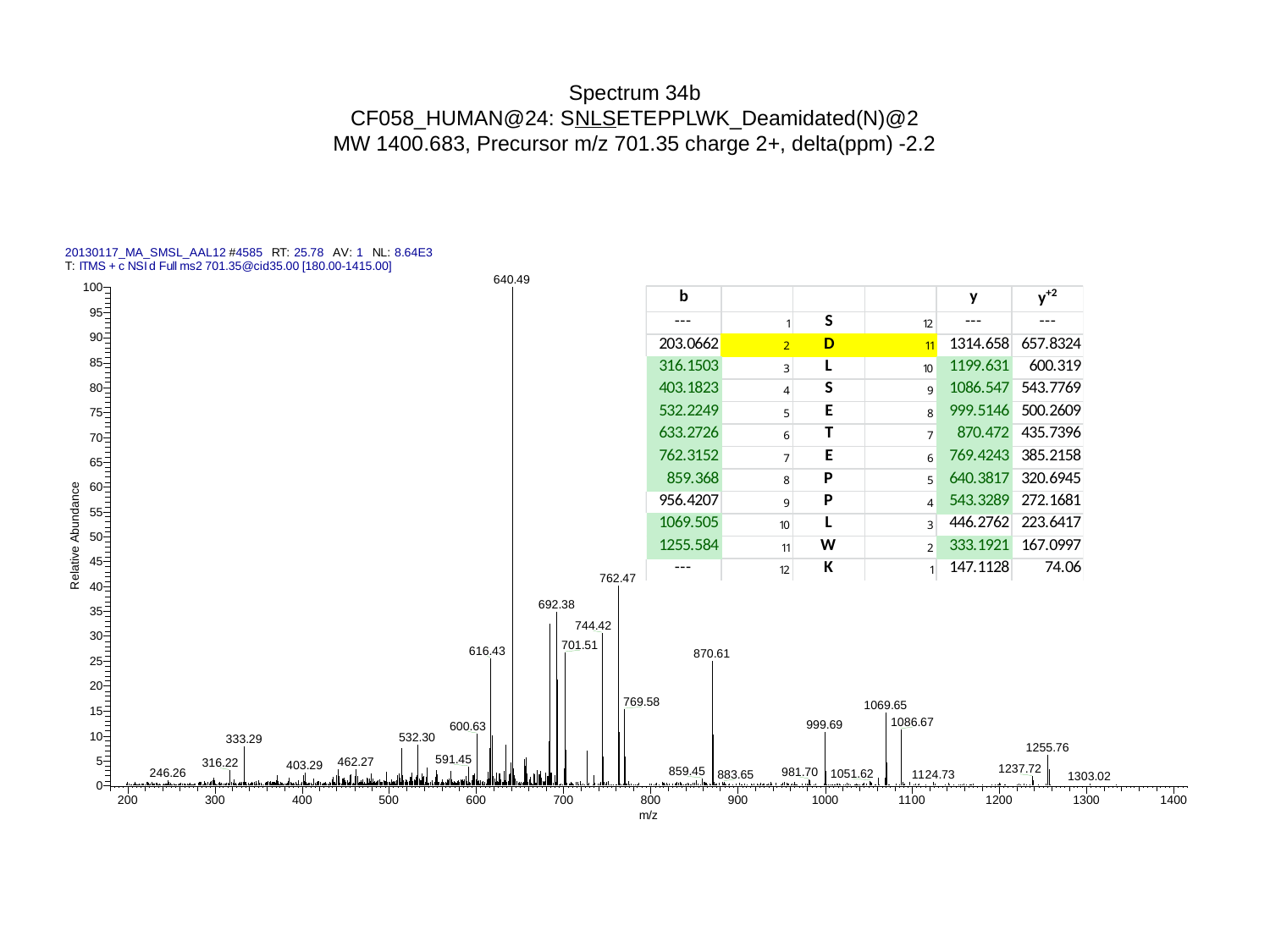

# Spectrum 34b CF058_HUMAN@24: SNLSETEPPLWK_Deamidated(N)@2 MW 1400.683, Precursor m/z 701.35 charge 2+, delta(ppm) -2.2

## Slide 44
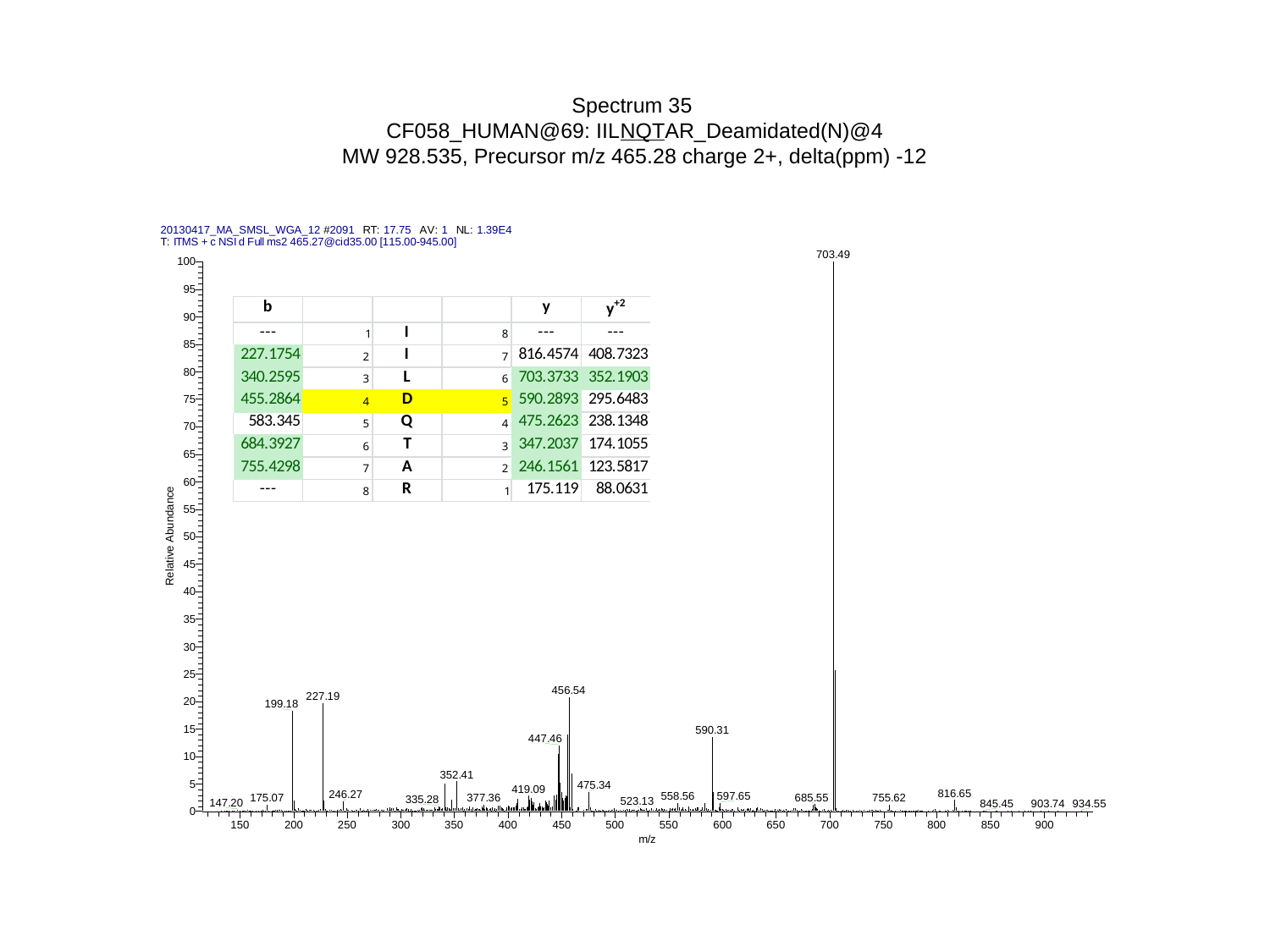

# Spectrum 35 CF058_HUMAN@69: IILNQTAR_Deamidated(N)@4 MW 928.535, Precursor m/z 465.28 charge 2+, delta(ppm) -12

## Slide 45
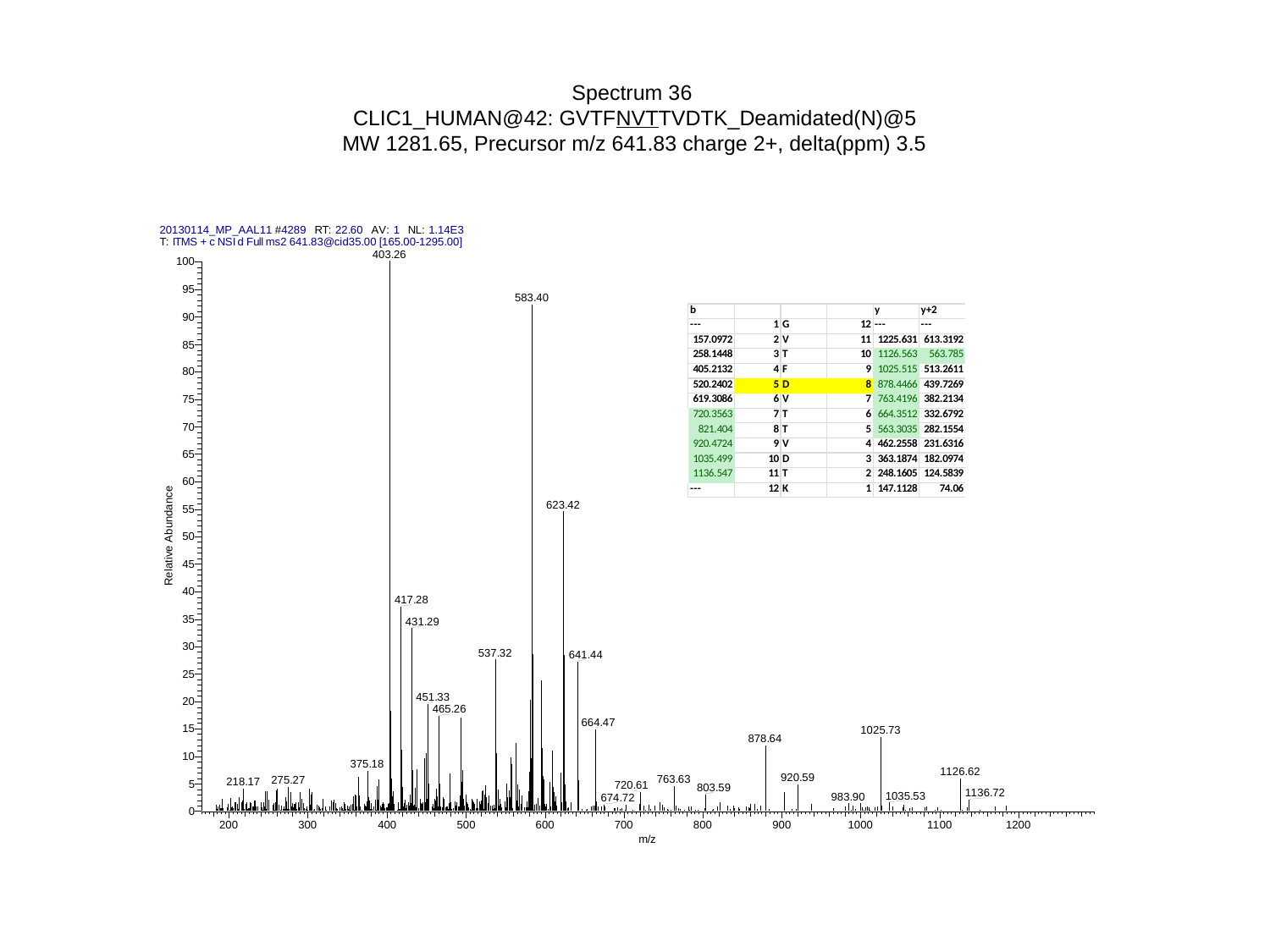

# Spectrum 36 CLIC1_HUMAN@42: GVTFNVTTVDTK_Deamidated(N)@5 MW 1281.65, Precursor m/z 641.83 charge 2+, delta(ppm) 3.5

## Slide 46
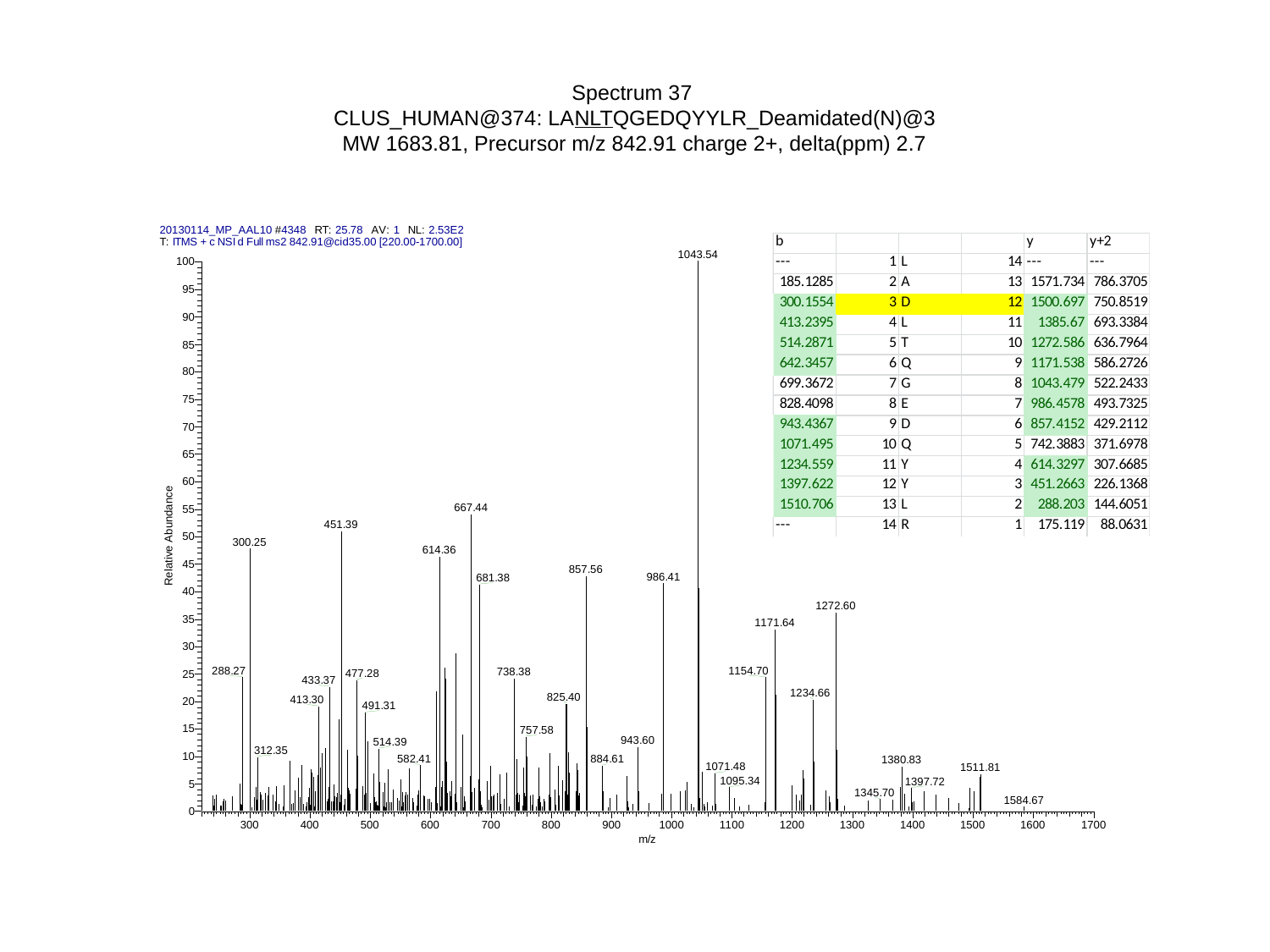

# Spectrum 37 CLUS_HUMAN@374: LANLTQGEDQYYLR_Deamidated(N)@3 MW 1683.81, Precursor m/z 842.91 charge 2+, delta(ppm) 2.7

## Slide 47
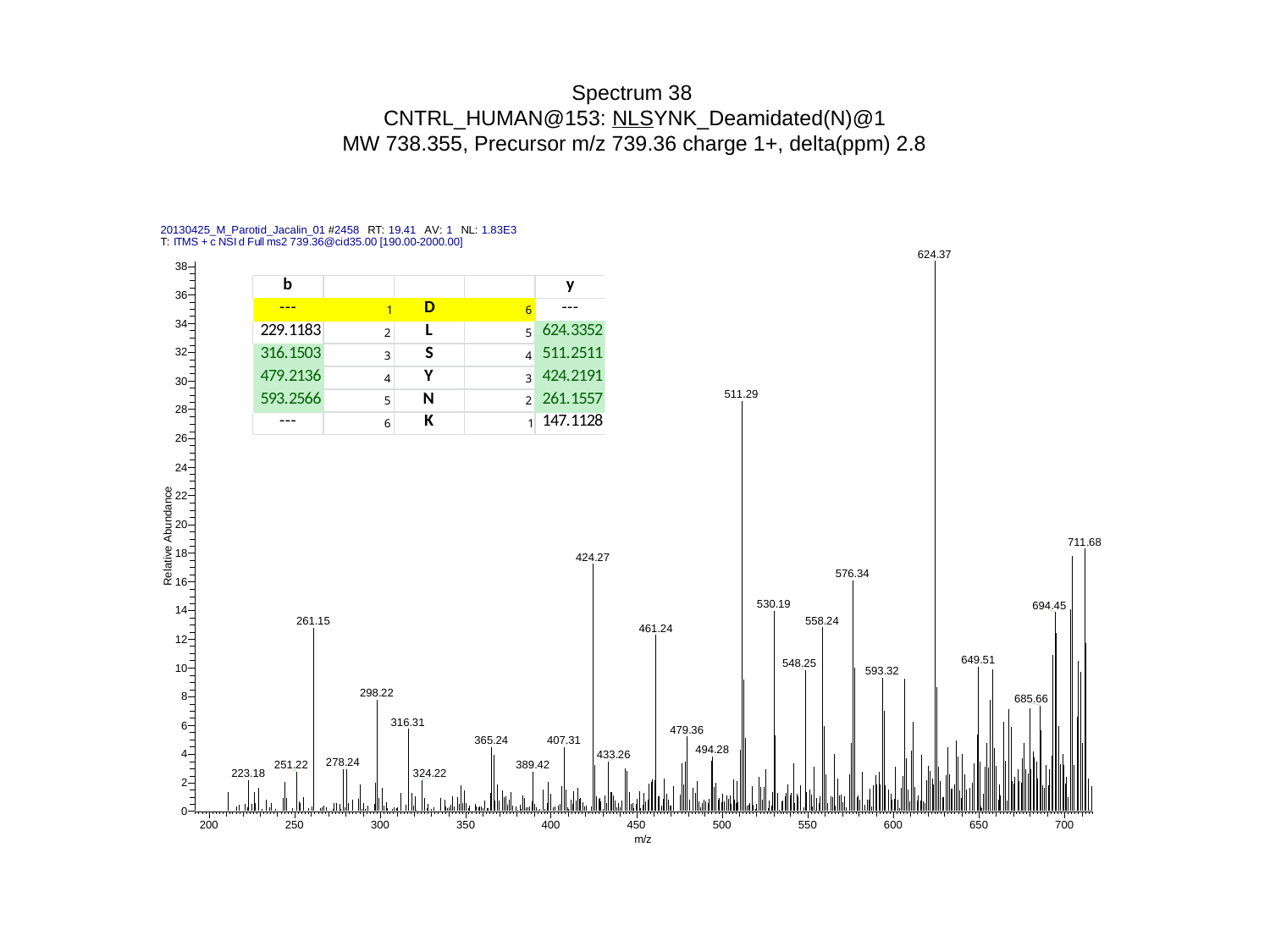

# Spectrum 38 CNTRL_HUMAN@153: NLSYNK_Deamidated(N)@1 MW 738.355, Precursor m/z 739.36 charge 1+, delta(ppm) 2.8

## Slide 48
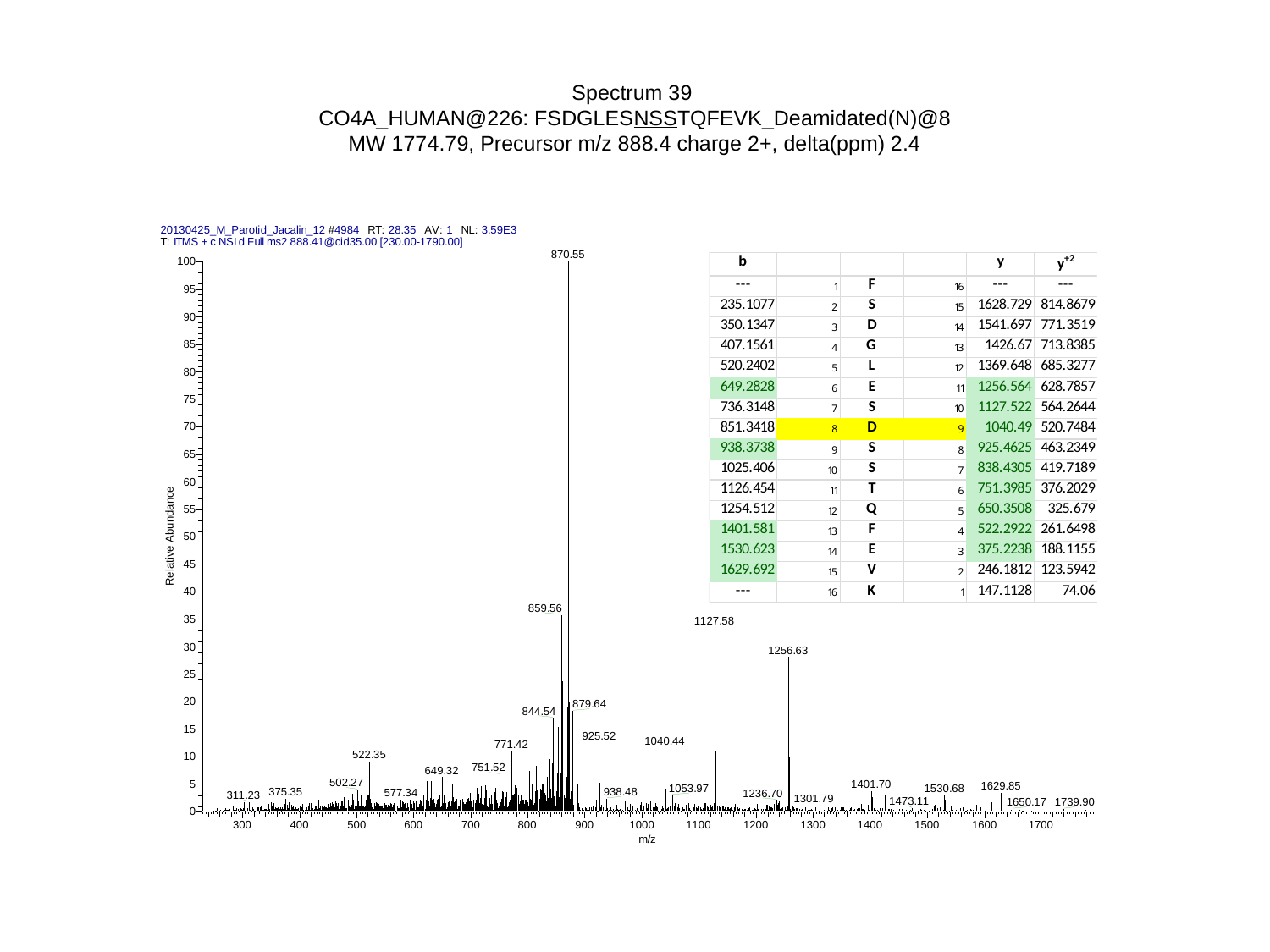

# Spectrum 39 CO4A_HUMAN@226: FSDGLESNSSTQFEVK_Deamidated(N)@8 MW 1774.79, Precursor m/z 888.4 charge 2+, delta(ppm) 2.4

## Slide 49
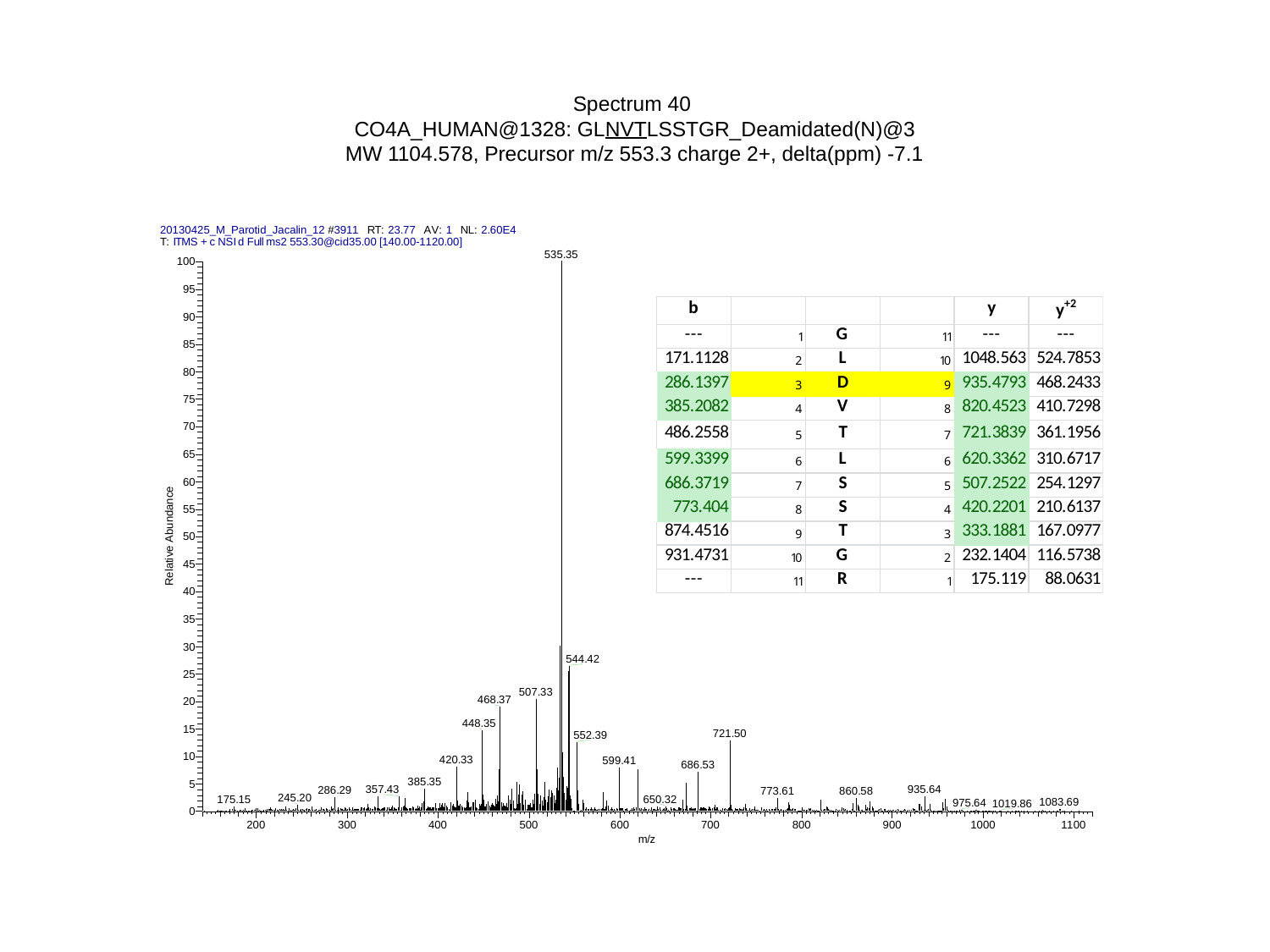

# Spectrum 40 CO4A_HUMAN@1328: GLNVTLSSTGR_Deamidated(N)@3 MW 1104.578, Precursor m/z 553.3 charge 2+, delta(ppm) -7.1

## Slide 50
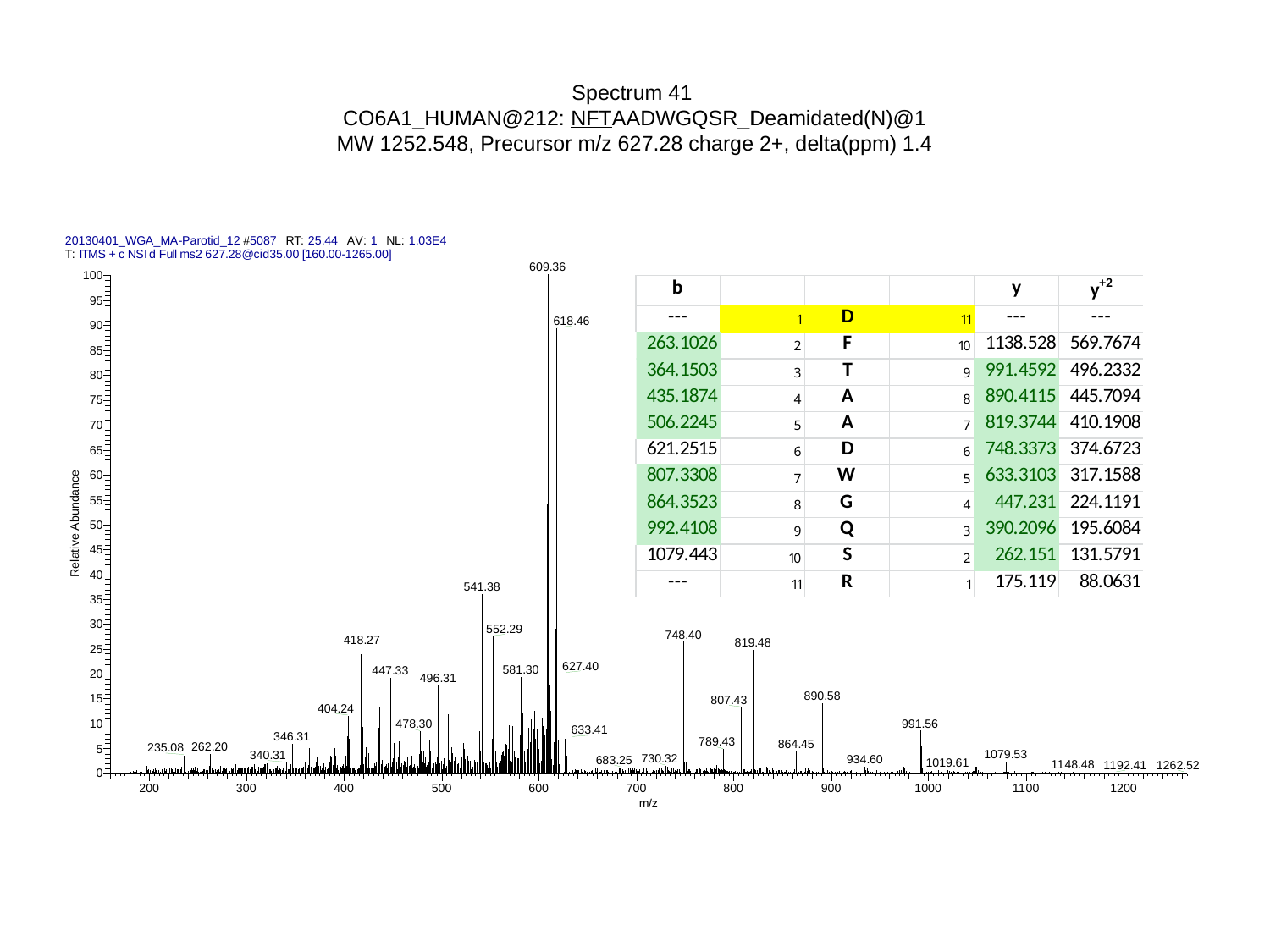

# Spectrum 41 CO6A1_HUMAN@212: NFTAADWGQSR_Deamidated(N)@1 MW 1252.548, Precursor m/z 627.28 charge 2+, delta(ppm) 1.4

## Slide 51
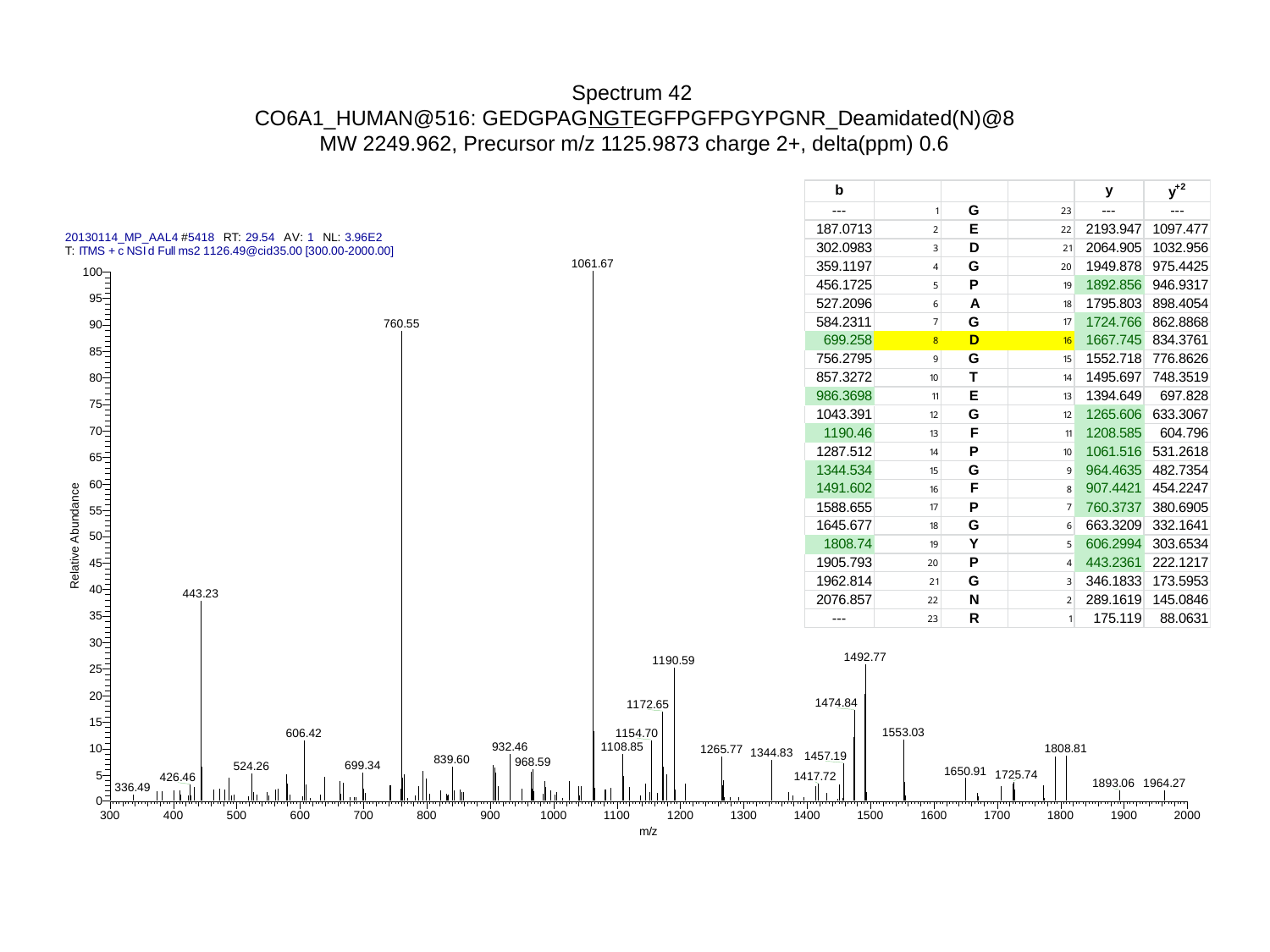

# Spectrum 42 CO6A1_HUMAN@516: GEDGPAGNGTEGFPGFPGYPGNR_Deamidated(N)@8 MW 2249.962, Precursor m/z 1125.9873 charge 2+, delta(ppm) 0.6

## Slide 52
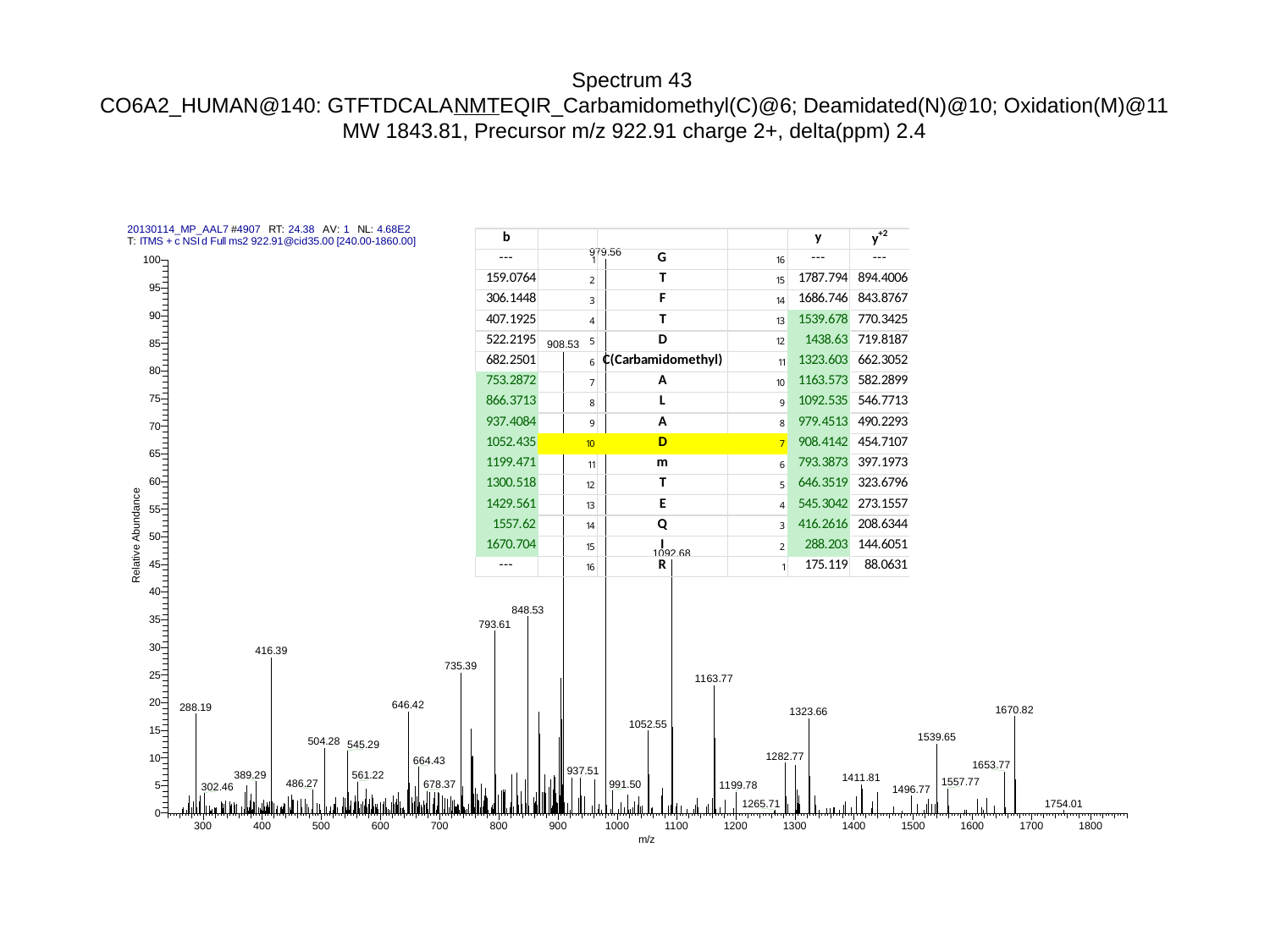

# Spectrum 43 CO6A2_HUMAN@140: GTFTDCALANMTEQIR_Carbamidomethyl(C)@6; Deamidated(N)@10; Oxidation(M)@11 MW 1843.81, Precursor m/z 922.91 charge 2+, delta(ppm) 2.4

## Slide 53
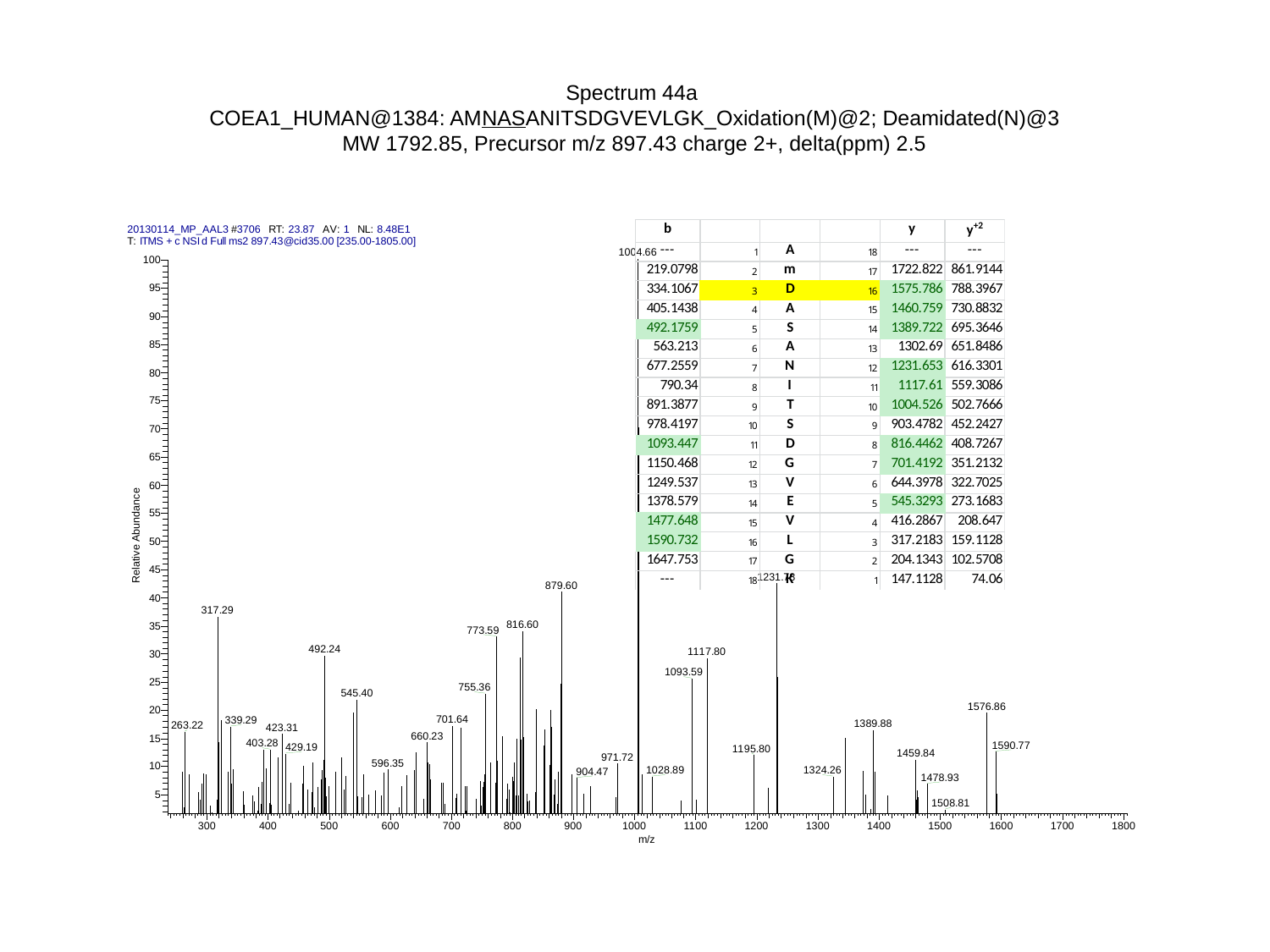

# Spectrum 44a COEA1_HUMAN@1384: AMNASANITSDGVEVLGK_Oxidation(M)@2; Deamidated(N)@3 MW 1792.85, Precursor m/z 897.43 charge 2+, delta(ppm) 2.5

## Slide 54
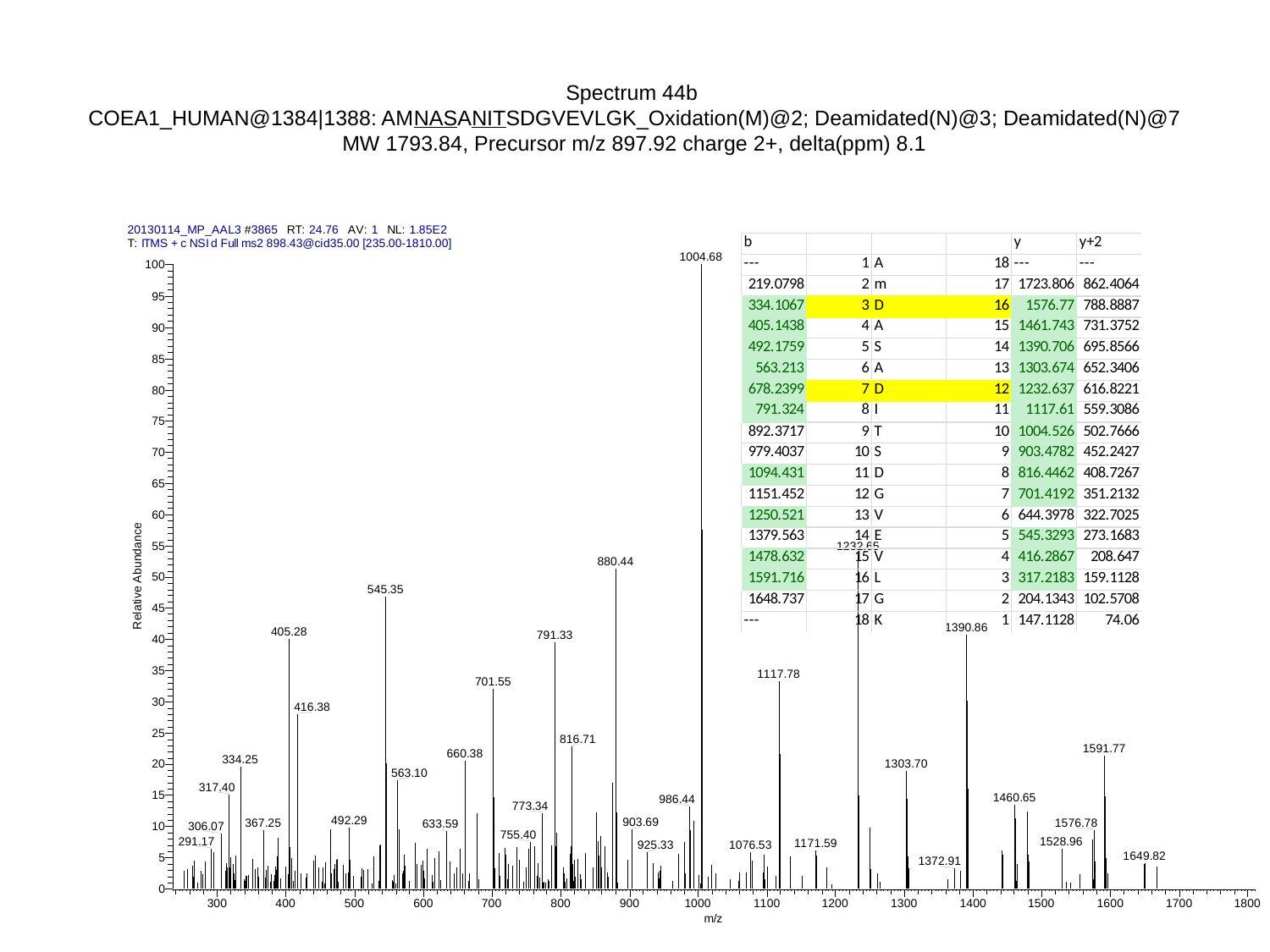

# Spectrum 44b COEA1_HUMAN@1384|1388: AMNASANITSDGVEVLGK_Oxidation(M)@2; Deamidated(N)@3; Deamidated(N)@7 MW 1793.84, Precursor m/z 897.92 charge 2+, delta(ppm) 8.1

## Slide 55
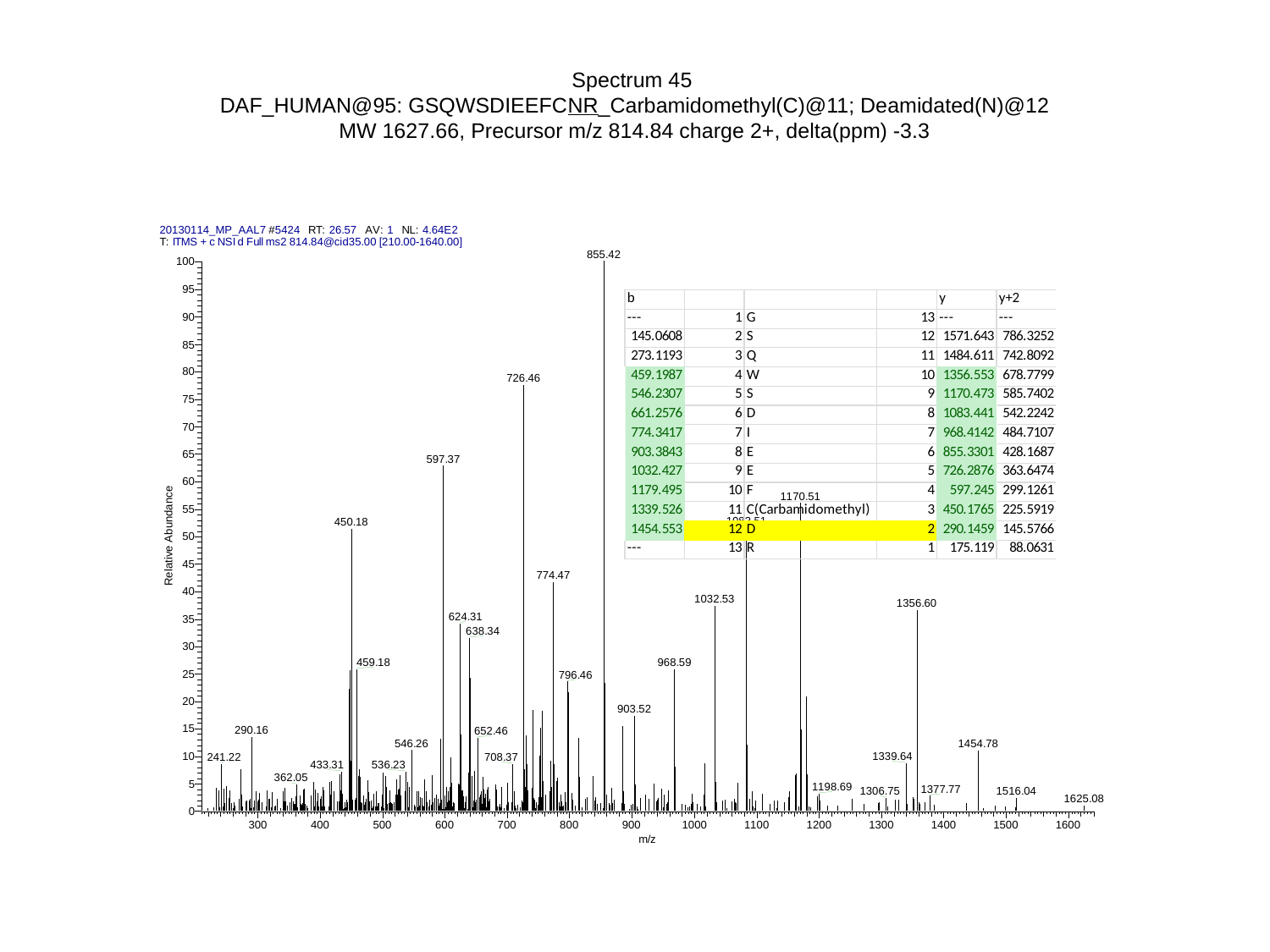

# Spectrum 45 DAF_HUMAN@95: GSQWSDIEEFCNR_Carbamidomethyl(C)@11; Deamidated(N)@12 MW 1627.66, Precursor m/z 814.84 charge 2+, delta(ppm) -3.3

## Slide 56
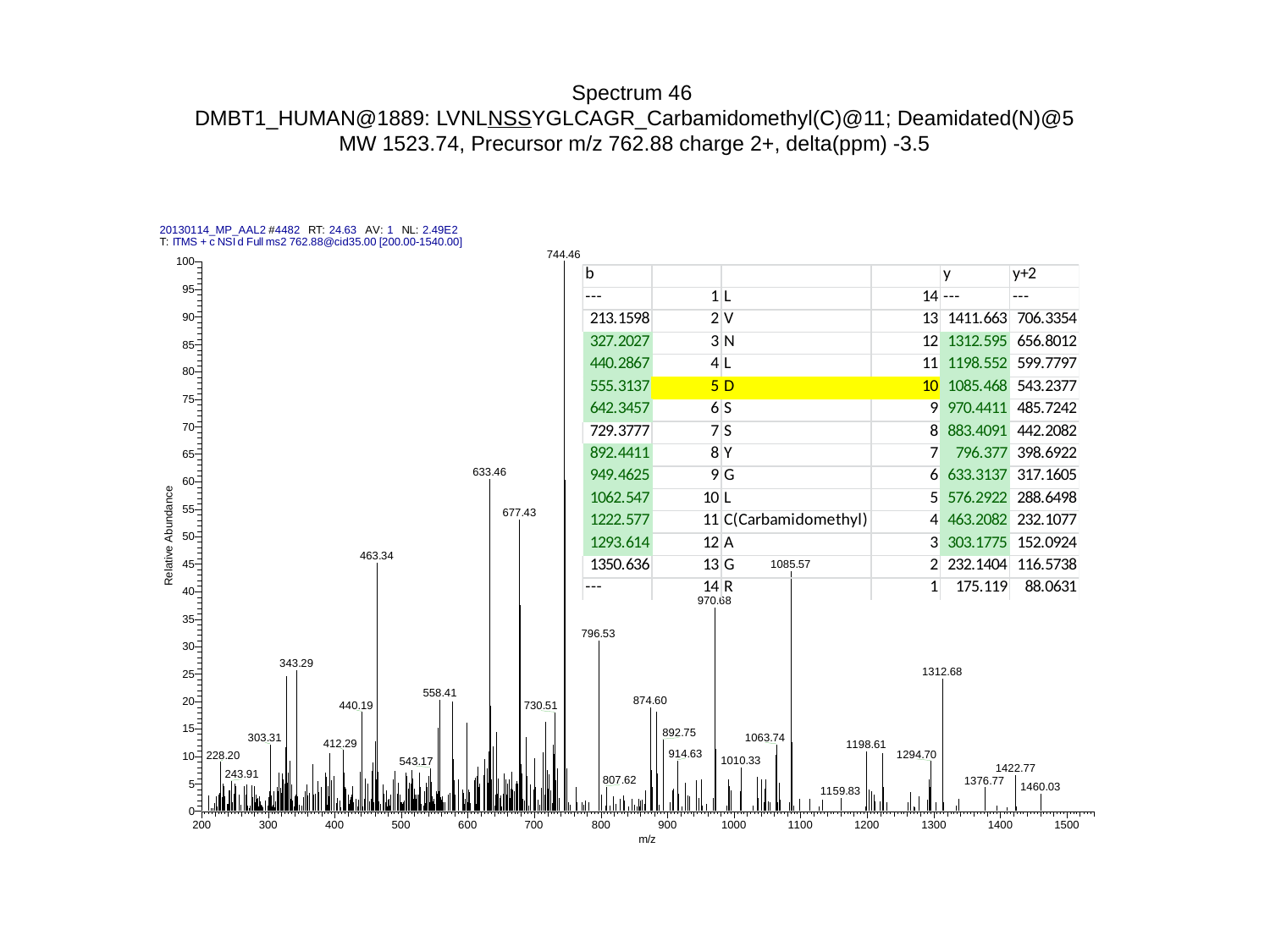

# Spectrum 46 DMBT1_HUMAN@1889: LVNLNSSYGLCAGR_Carbamidomethyl(C)@11; Deamidated(N)@5 MW 1523.74, Precursor m/z 762.88 charge 2+, delta(ppm) -3.5

## Slide 57
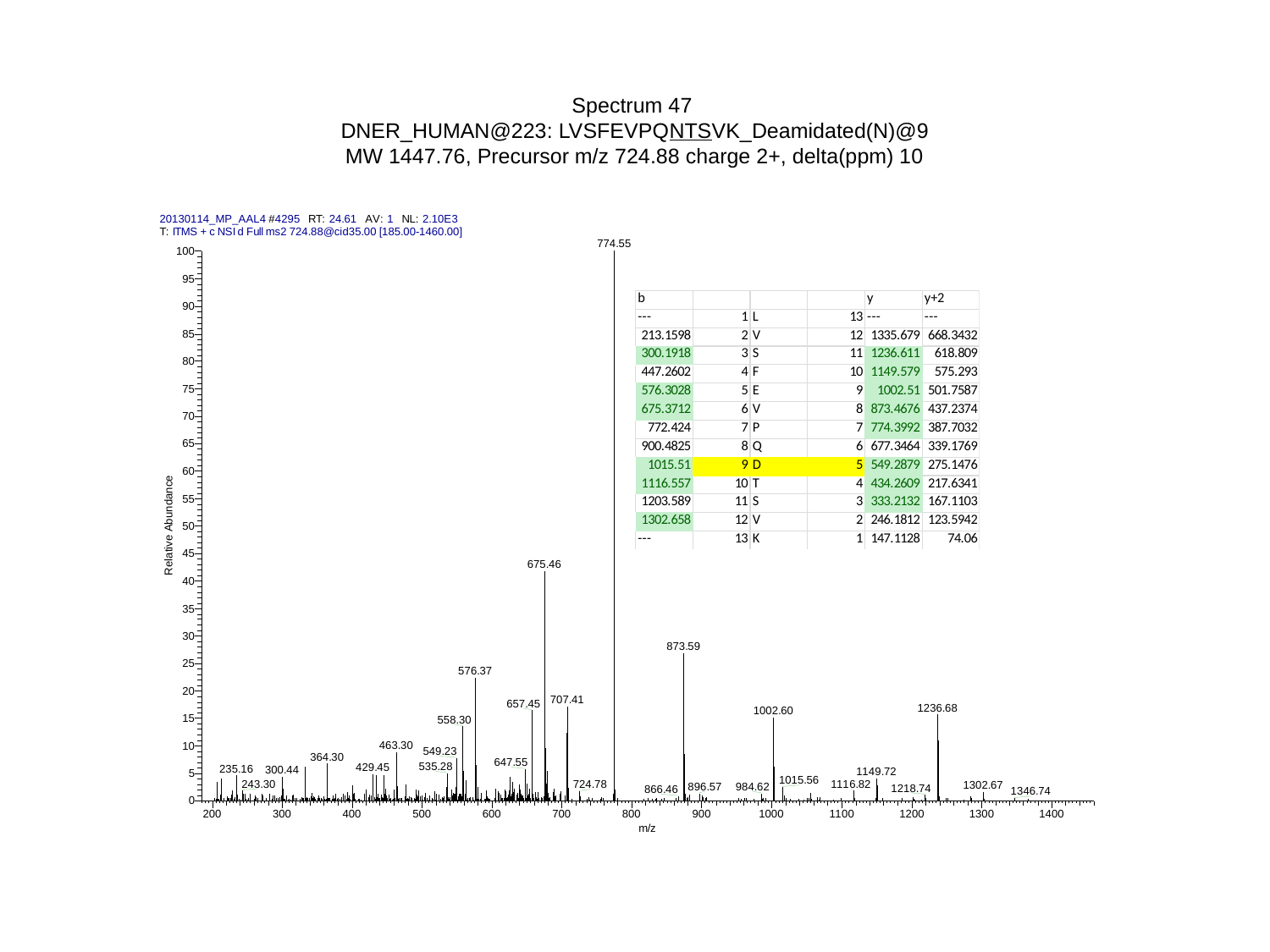

# Spectrum 47 DNER_HUMAN@223: LVSFEVPQNTSVK_Deamidated(N)@9 MW 1447.76, Precursor m/z 724.88 charge 2+, delta(ppm) 10

## Slide 58
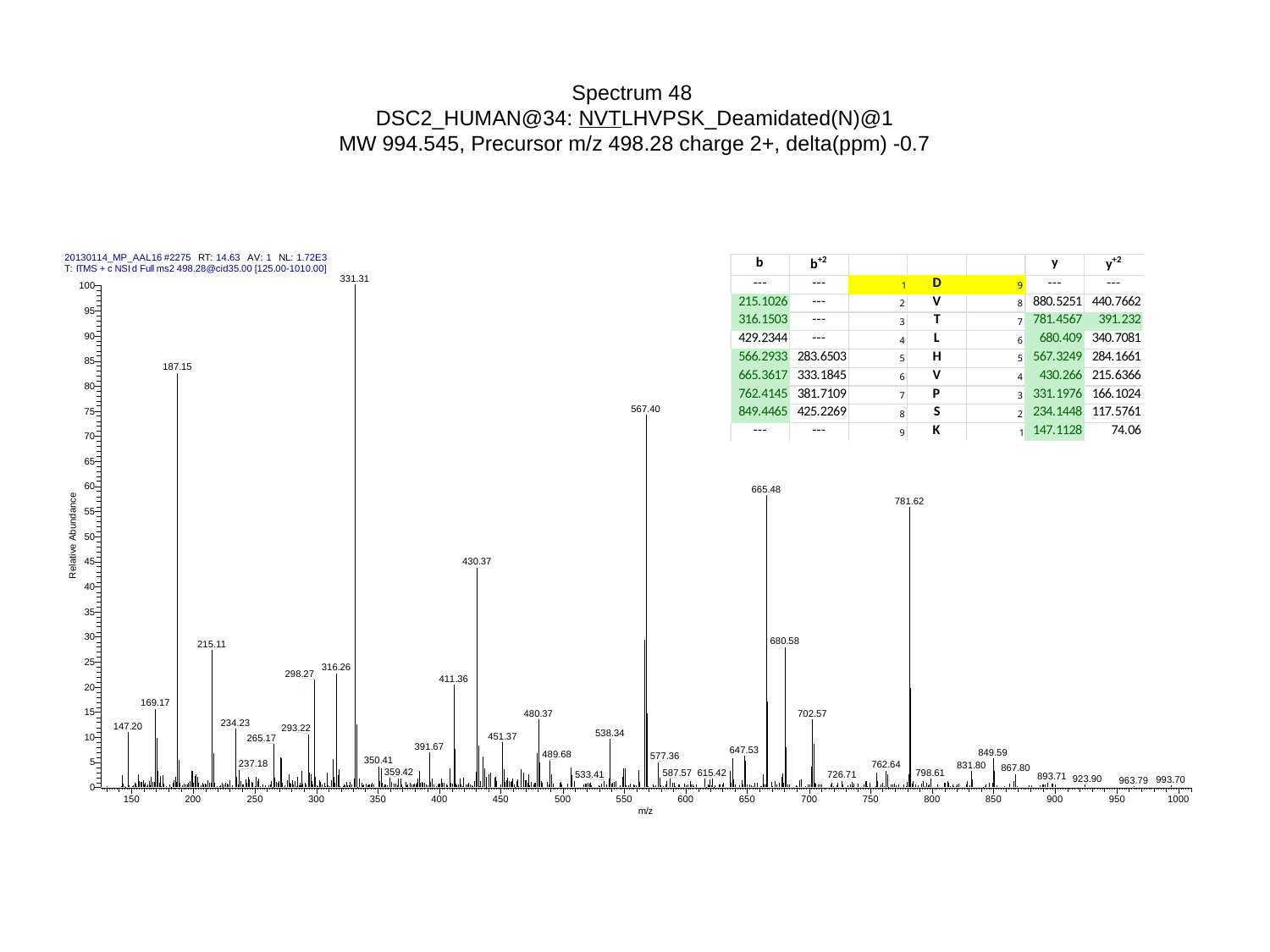

# Spectrum 48 DSC2_HUMAN@34: NVTLHVPSK_Deamidated(N)@1 MW 994.545, Precursor m/z 498.28 charge 2+, delta(ppm) -0.7

## Slide 59
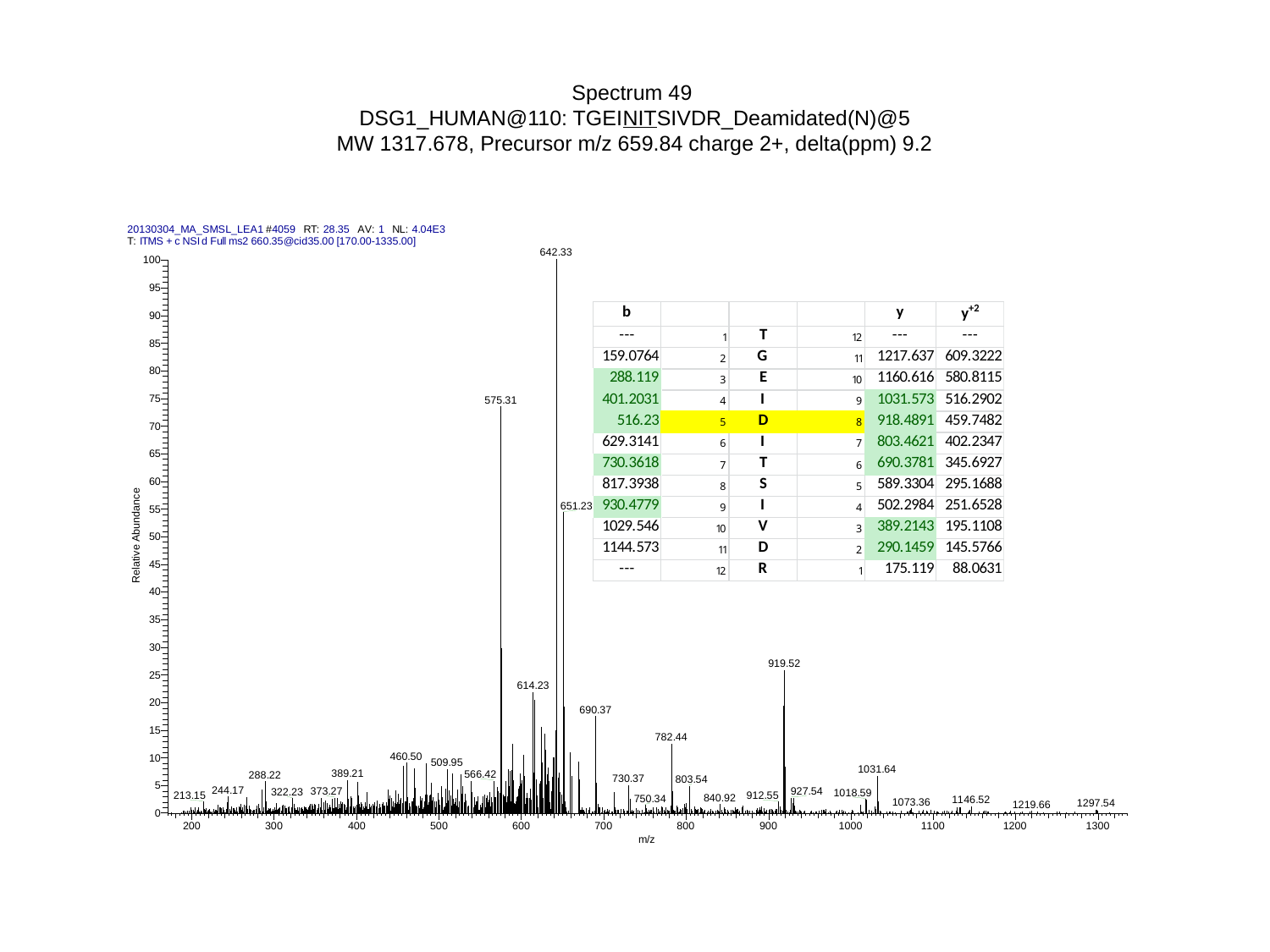

# Spectrum 49 DSG1_HUMAN@110: TGEINITSIVDR_Deamidated(N)@5 MW 1317.678, Precursor m/z 659.84 charge 2+, delta(ppm) 9.2

## Slide 60
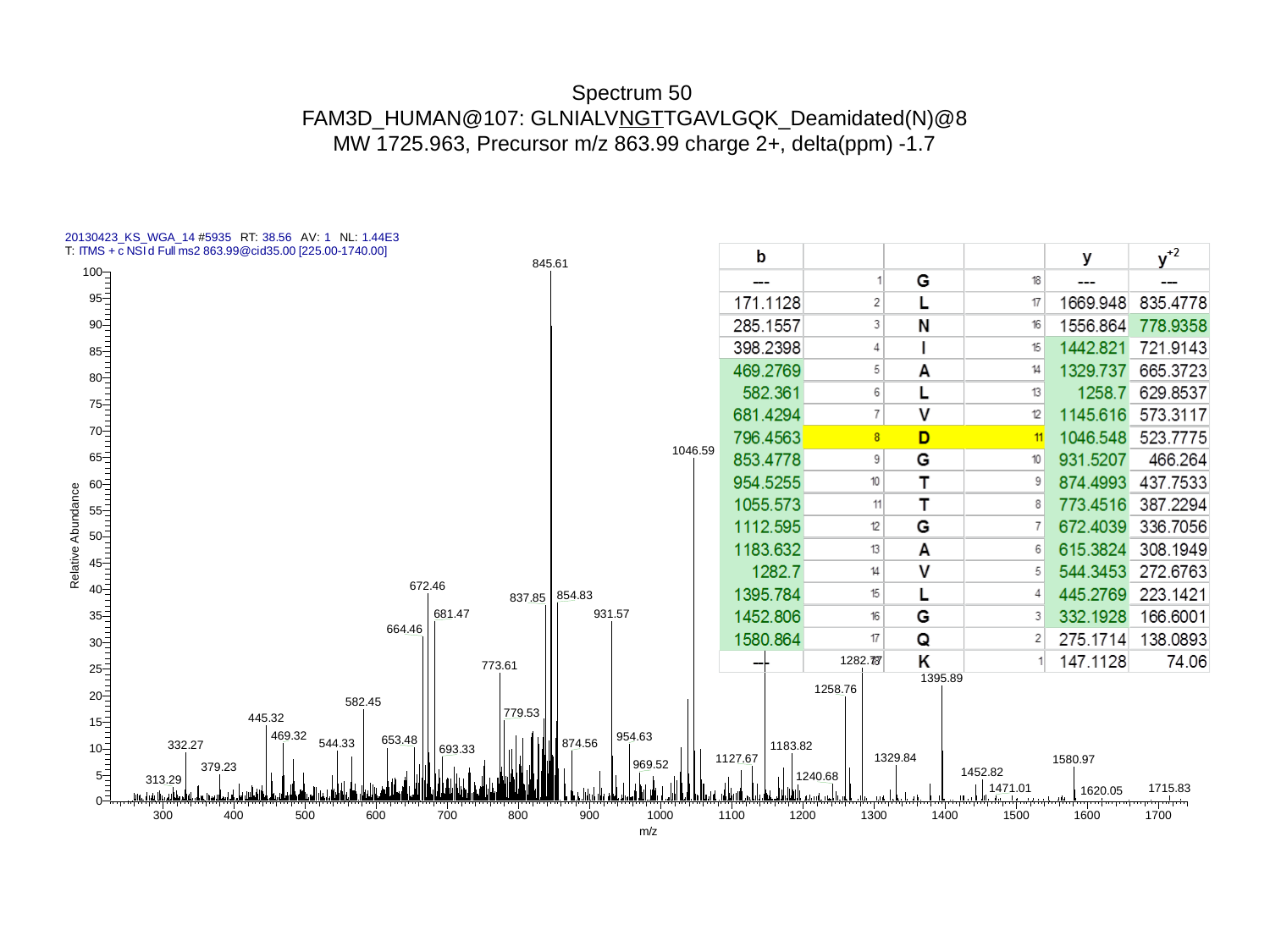

# Spectrum 50 FAM3D_HUMAN@107: GLNIALVNGTTGAVLGQK_Deamidated(N)@8 MW 1725.963, Precursor m/z 863.99 charge 2+, delta(ppm) -1.7

## Slide 61
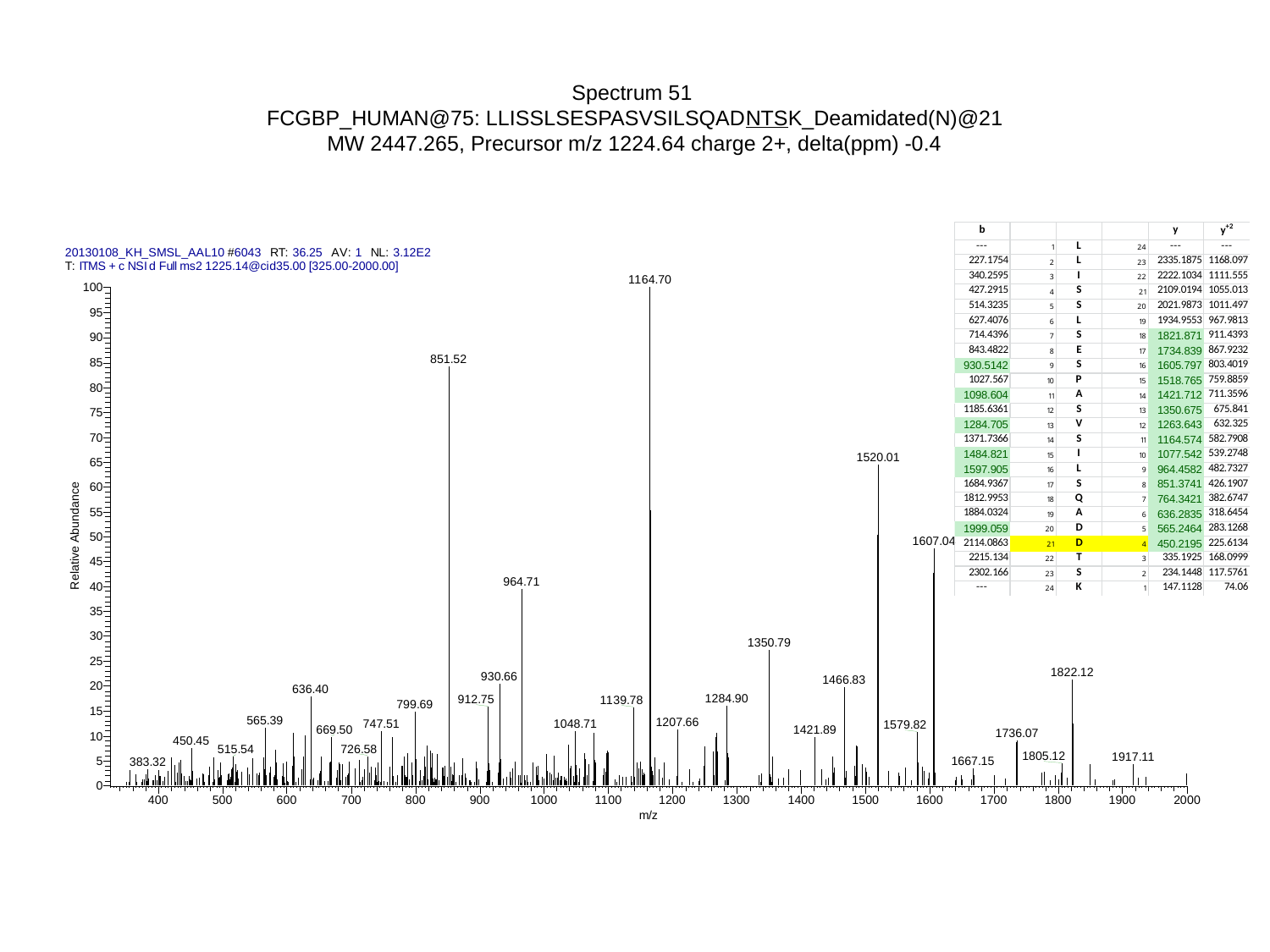

# Spectrum 51 FCGBP_HUMAN@75: LLISSLSESPASVSILSQADNTSK_Deamidated(N)@21 MW 2447.265, Precursor m/z 1224.64 charge 2+, delta(ppm) -0.4

## Slide 62
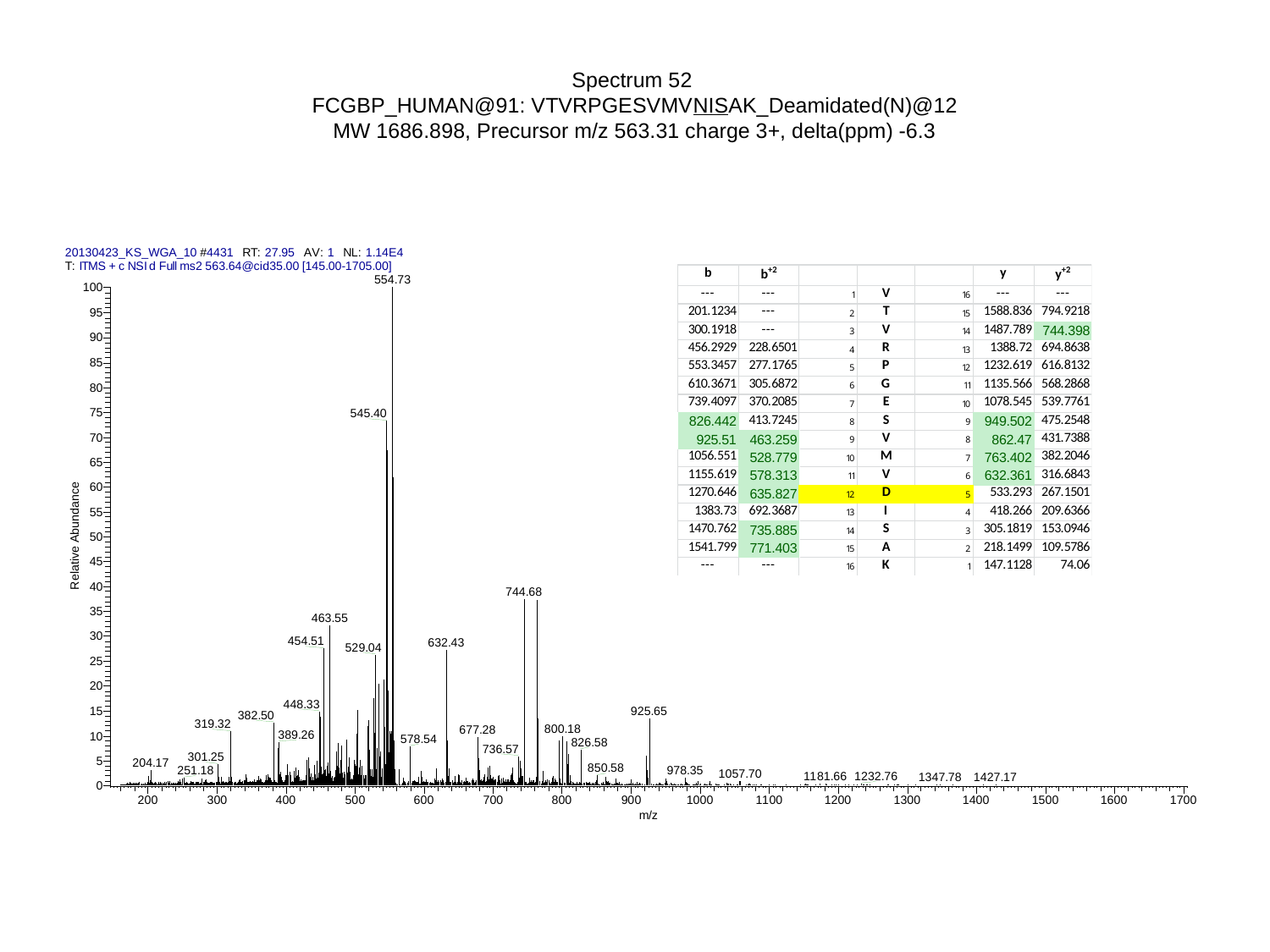

# Spectrum 52 FCGBP_HUMAN@91: VTVRPGESVMVNISAK_Deamidated(N)@12 MW 1686.898, Precursor m/z 563.31 charge 3+, delta(ppm) -6.3

## Slide 63
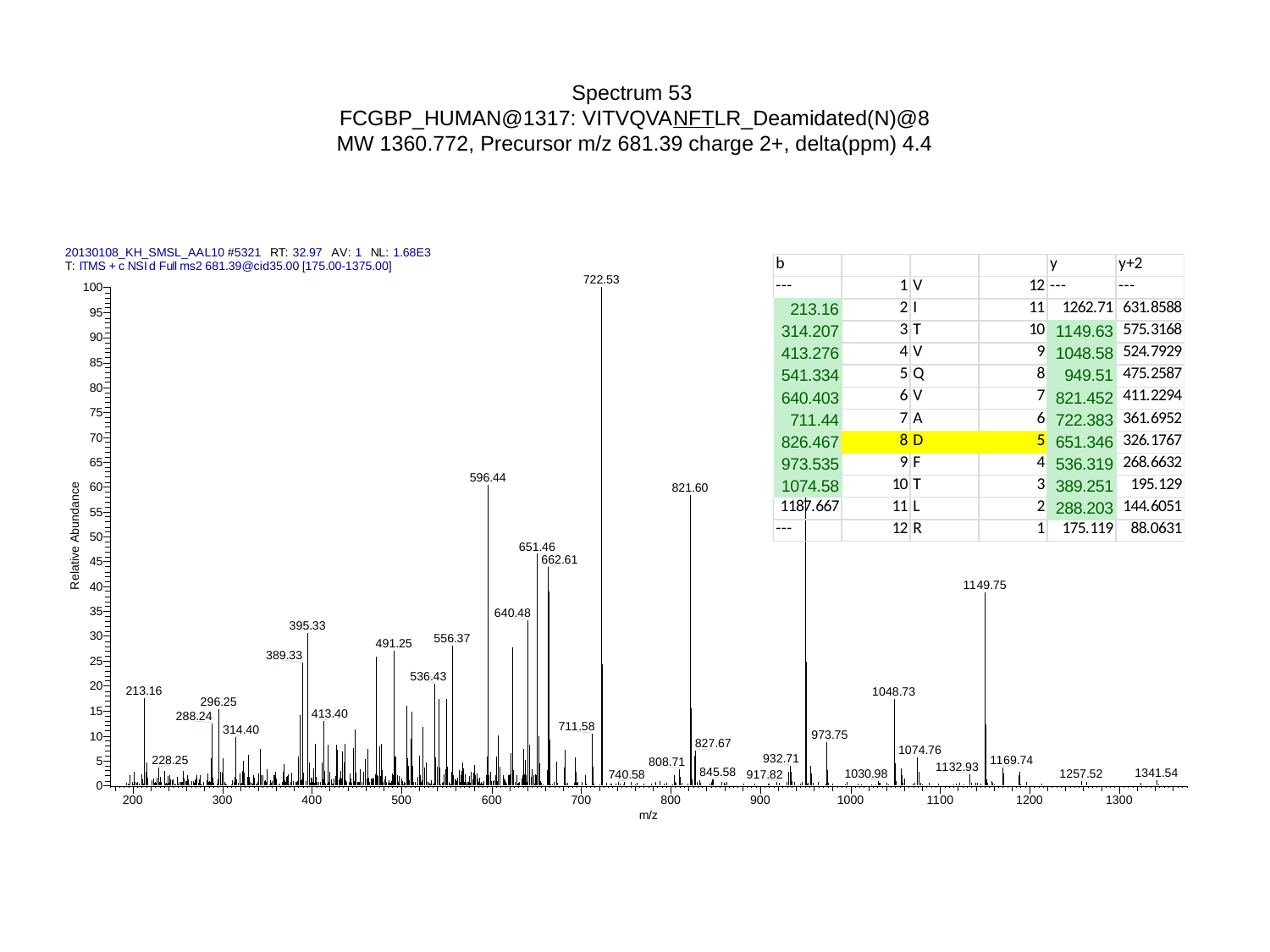

# Spectrum 53 FCGBP_HUMAN@1317: VITVQVANFTLR_Deamidated(N)@8 MW 1360.772, Precursor m/z 681.39 charge 2+, delta(ppm) 4.4

## Slide 64
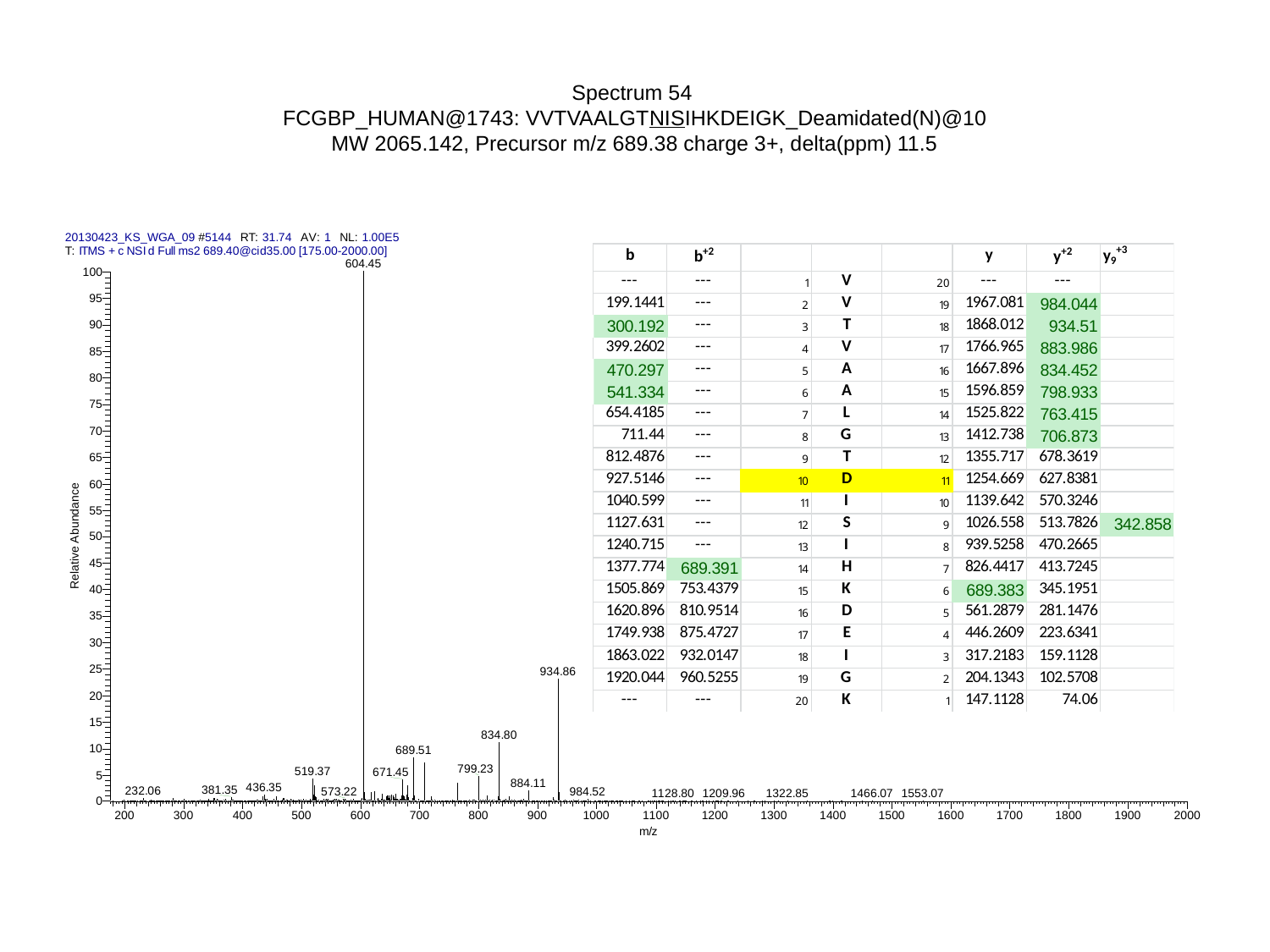

# Spectrum 54 FCGBP_HUMAN@1743: VVTVAALGTNISIHKDEIGK_Deamidated(N)@10 MW 2065.142, Precursor m/z 689.38 charge 3+, delta(ppm) 11.5

## Slide 65
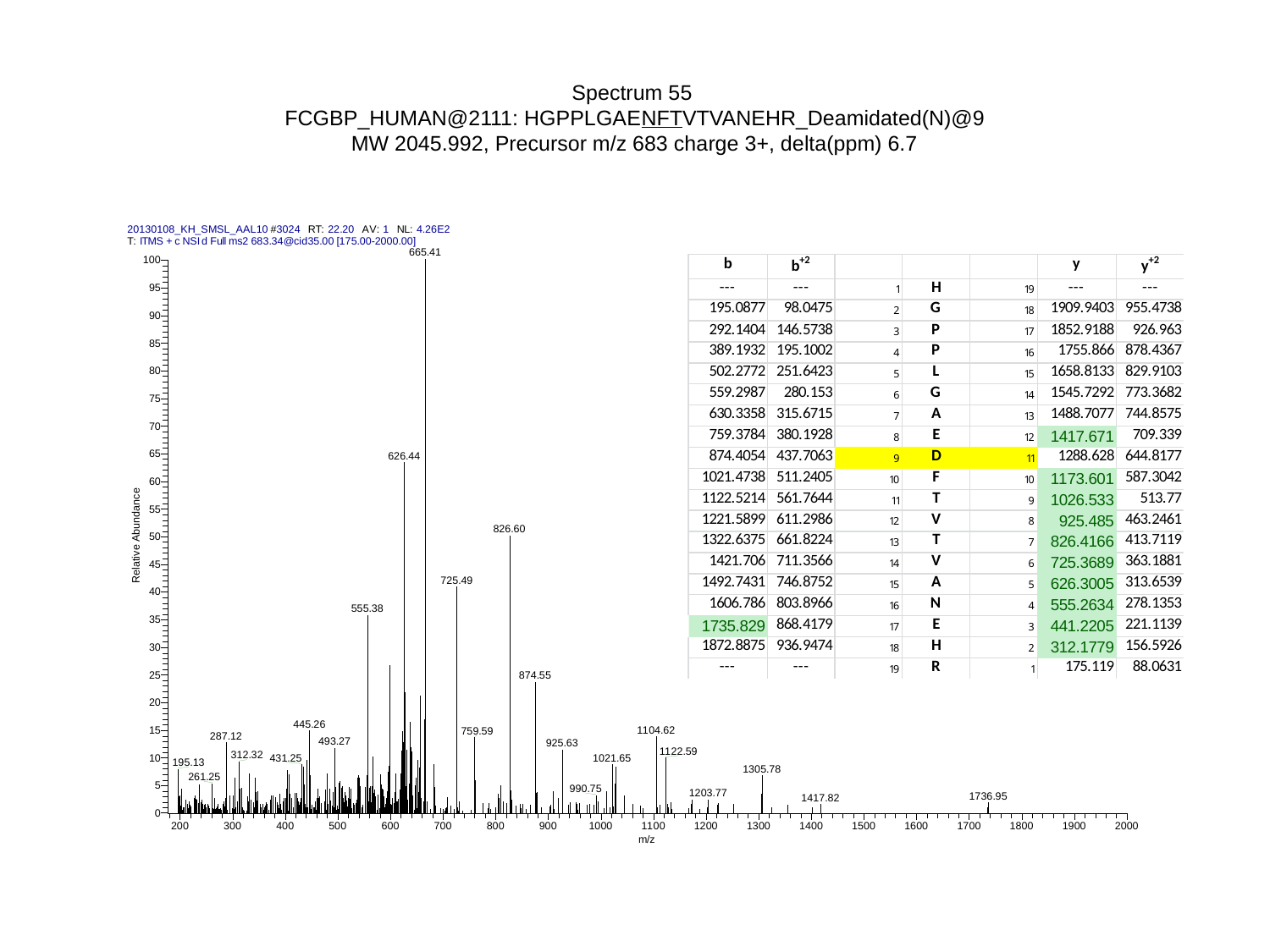

# Spectrum 55 FCGBP_HUMAN@2111: HGPPLGAENFTVTVANEHR_Deamidated(N)@9 MW 2045.992, Precursor m/z 683 charge 3+, delta(ppm) 6.7

## Slide 66
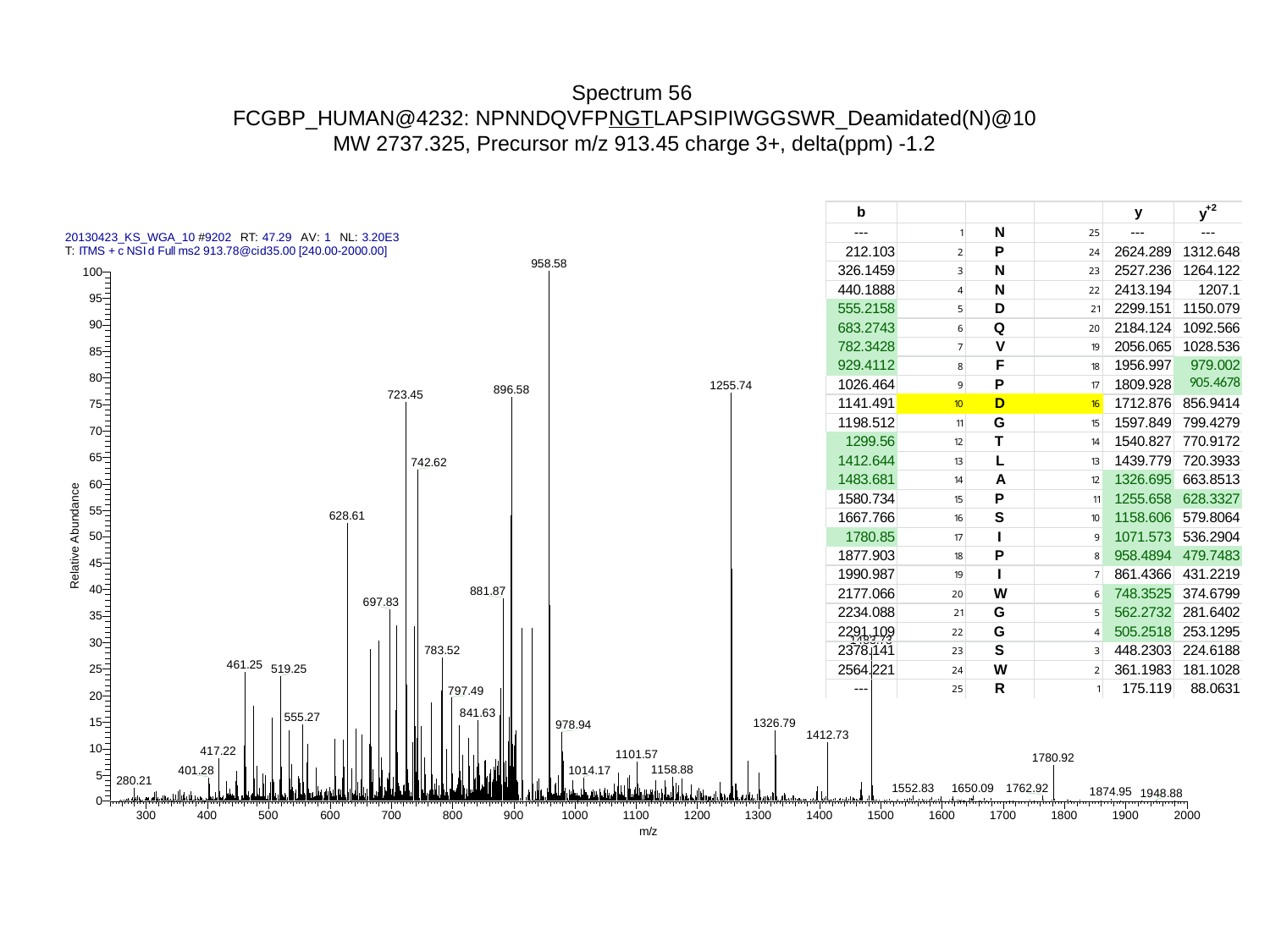

# Spectrum 56 FCGBP_HUMAN@4232: NPNNDQVFPNGTLAPSIPIWGGSWR_Deamidated(N)@10 MW 2737.325, Precursor m/z 913.45 charge 3+, delta(ppm) -1.2

## Slide 67
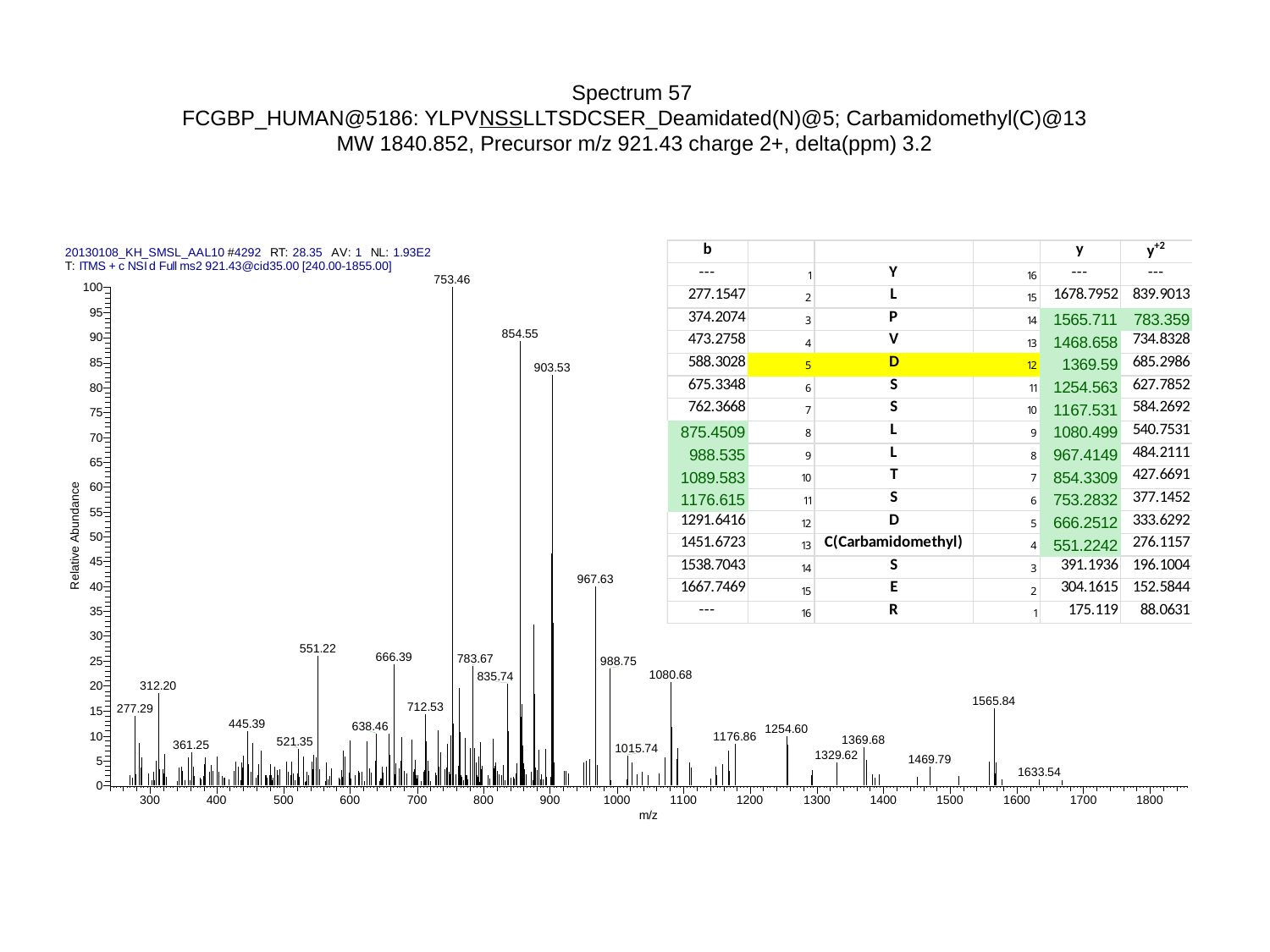

# Spectrum 57 FCGBP_HUMAN@5186: YLPVNSSLLTSDCSER_Deamidated(N)@5; Carbamidomethyl(C)@13 MW 1840.852, Precursor m/z 921.43 charge 2+, delta(ppm) 3.2

## Slide 68
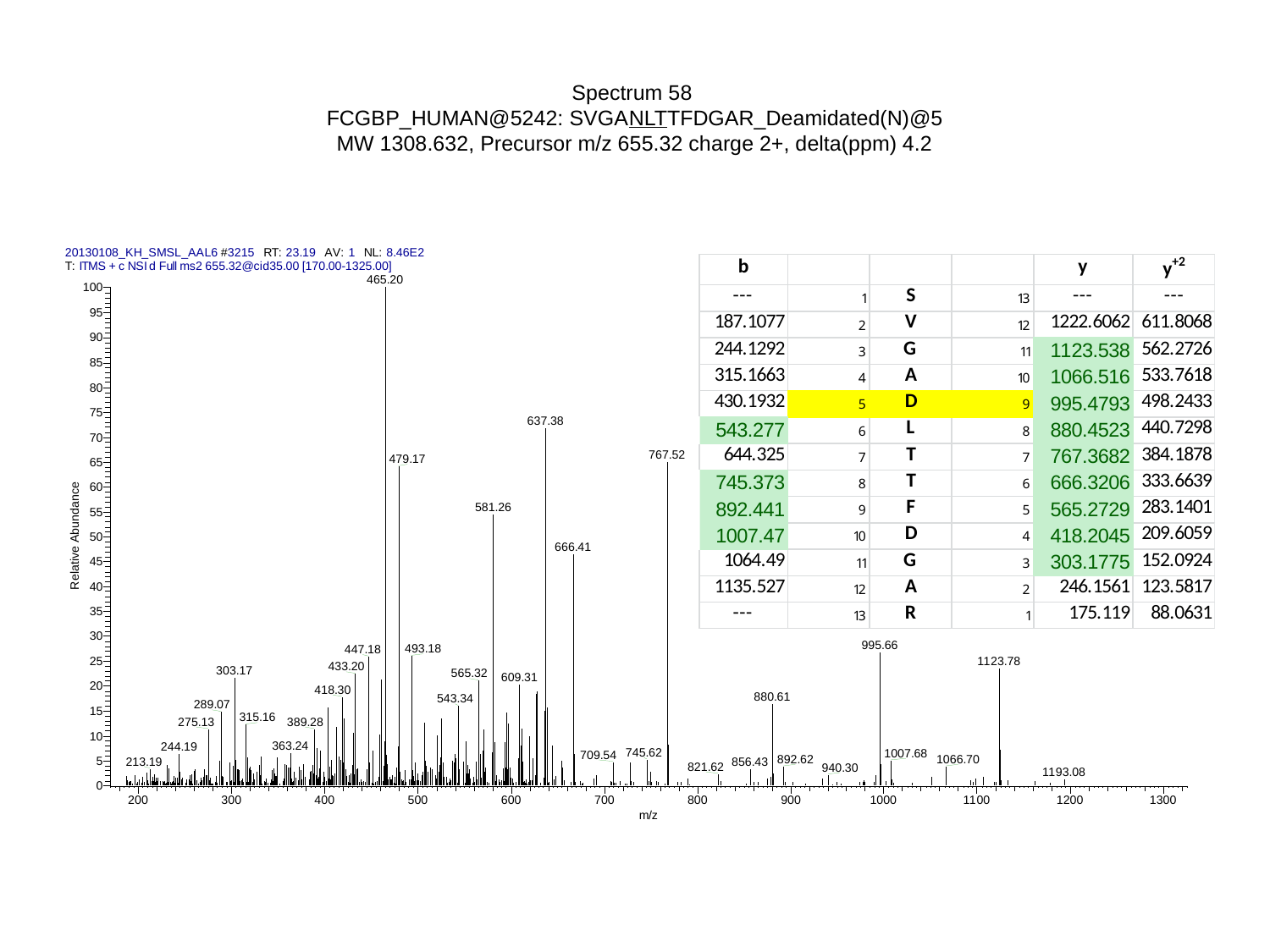

# Spectrum 58 FCGBP_HUMAN@5242: SVGANLTTFDGAR_Deamidated(N)@5 MW 1308.632, Precursor m/z 655.32 charge 2+, delta(ppm) 4.2

## Slide 69
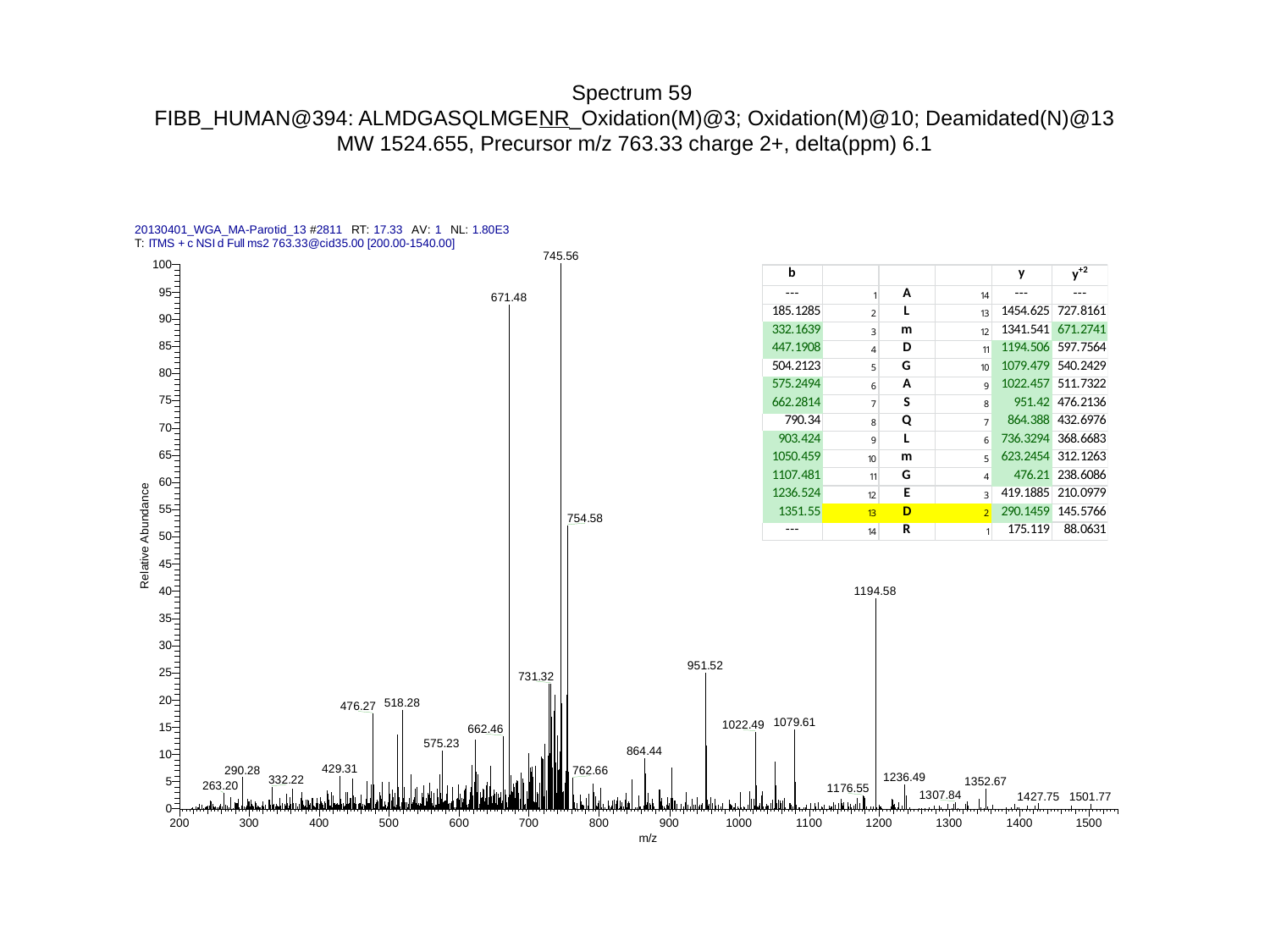

# Spectrum 59 FIBB_HUMAN@394: ALMDGASQLMGENR_Oxidation(M)@3; Oxidation(M)@10; Deamidated(N)@13 MW 1524.655, Precursor m/z 763.33 charge 2+, delta(ppm) 6.1

## Slide 70
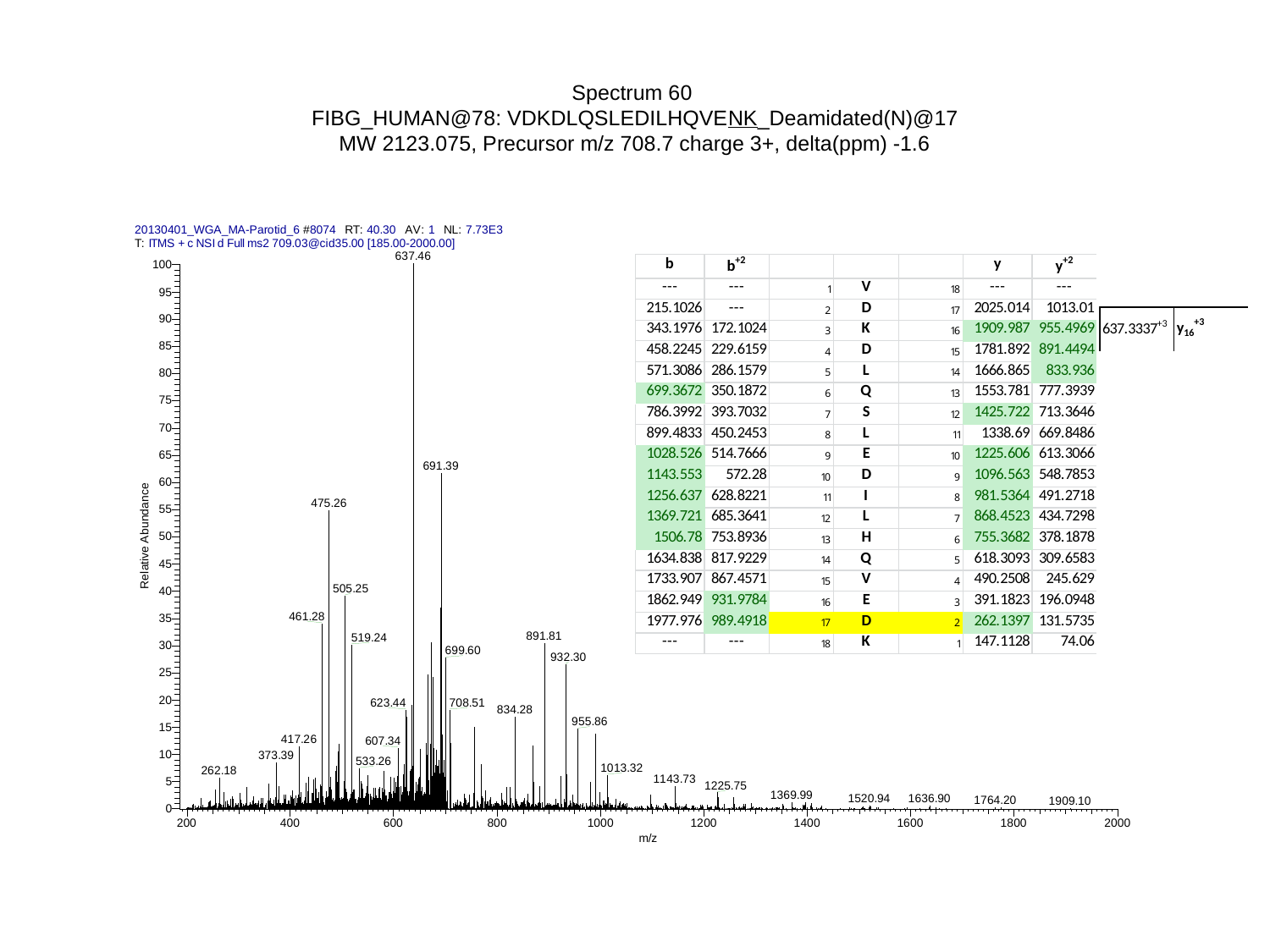

# Spectrum 60 FIBG_HUMAN@78: VDKDLQSLEDILHQVENK_Deamidated(N)@17 MW 2123.075, Precursor m/z 708.7 charge 3+, delta(ppm) -1.6

## Slide 71
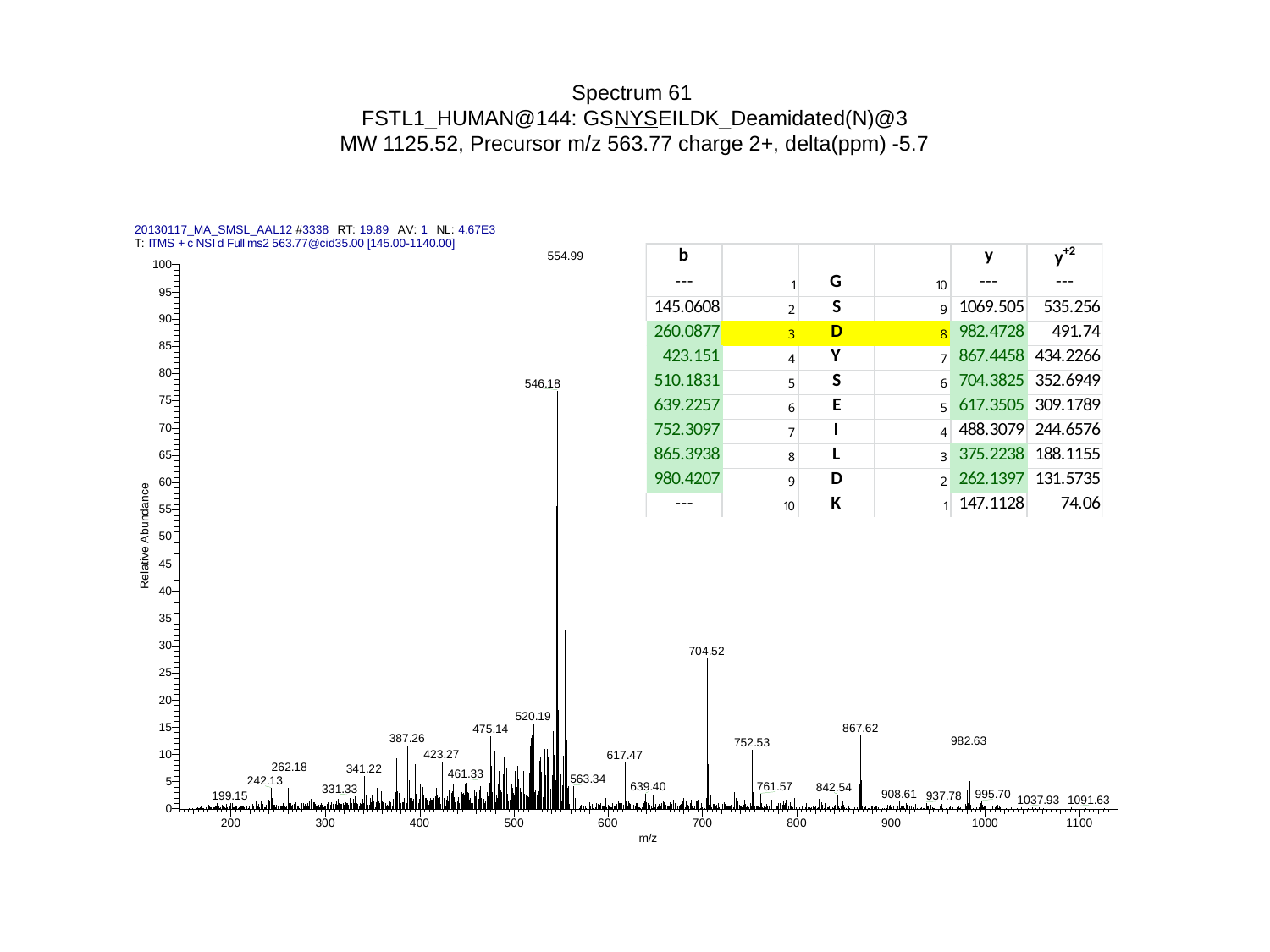

# Spectrum 61 FSTL1_HUMAN@144: GSNYSEILDK_Deamidated(N)@3 MW 1125.52, Precursor m/z 563.77 charge 2+, delta(ppm) -5.7

## Slide 72
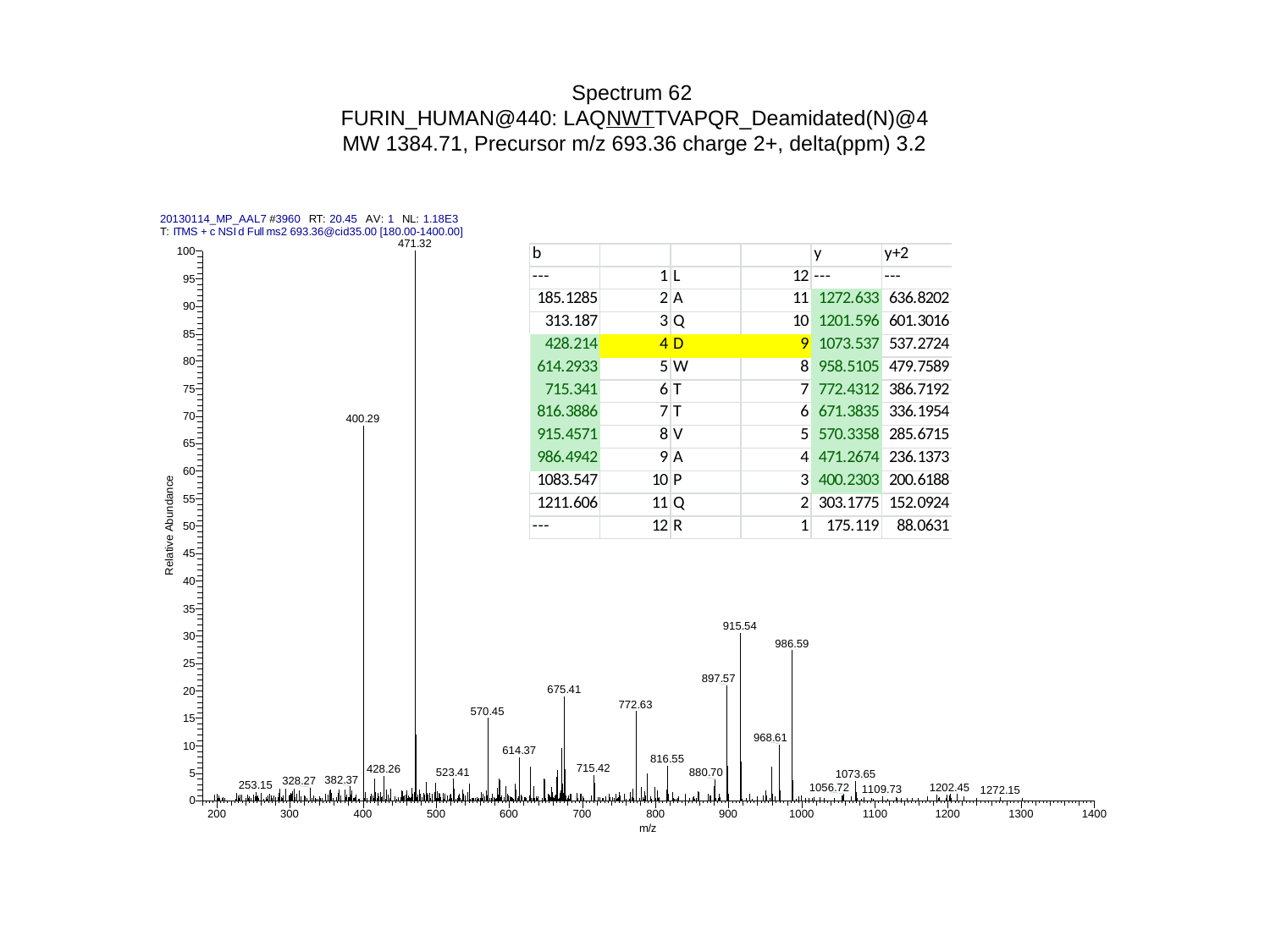

# Spectrum 62 FURIN_HUMAN@440: LAQNWTTVAPQR_Deamidated(N)@4 MW 1384.71, Precursor m/z 693.36 charge 2+, delta(ppm) 3.2

## Slide 73
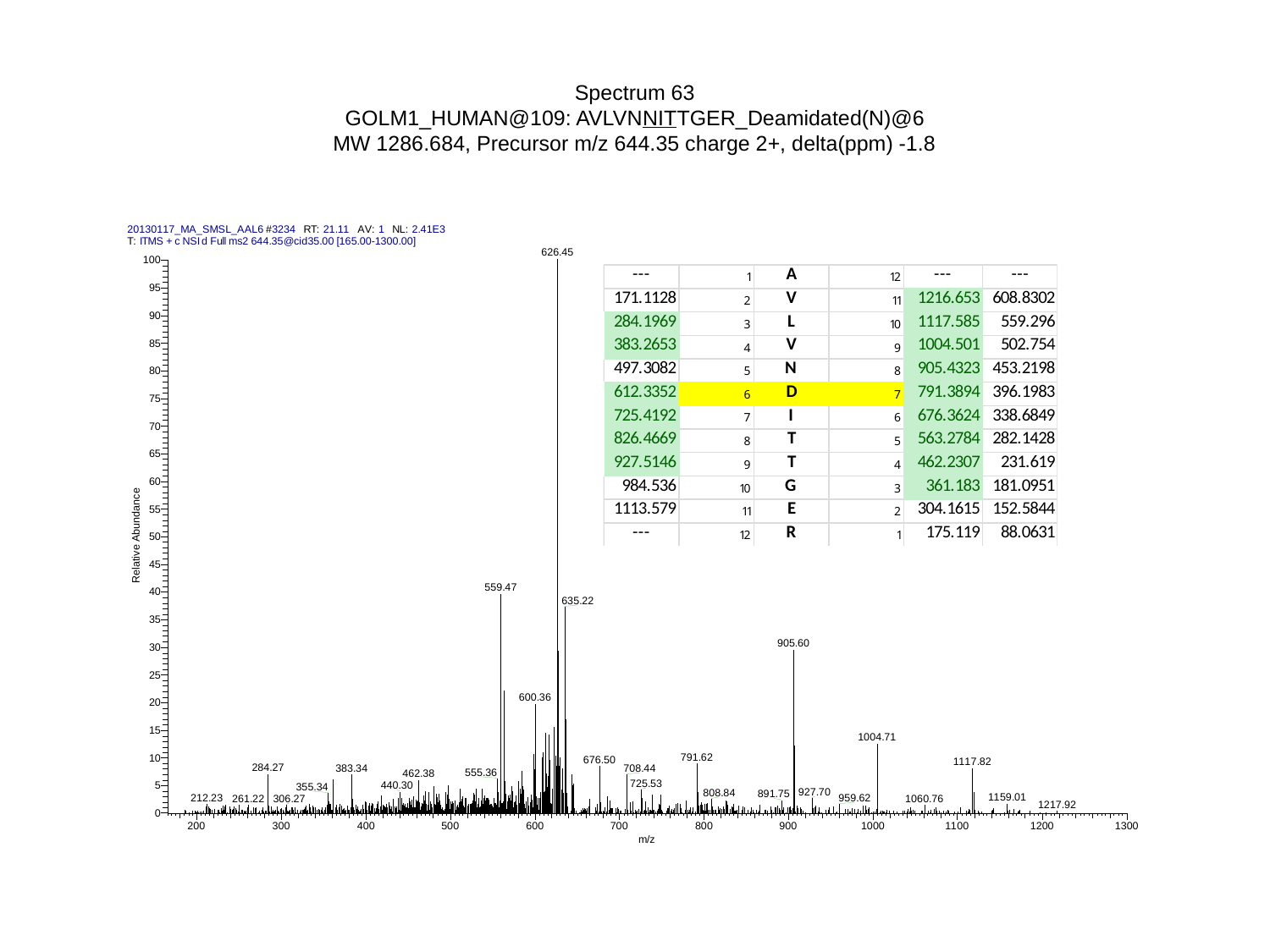

# Spectrum 63 GOLM1_HUMAN@109: AVLVNNITTGER_Deamidated(N)@6 MW 1286.684, Precursor m/z 644.35 charge 2+, delta(ppm) -1.8

## Slide 74
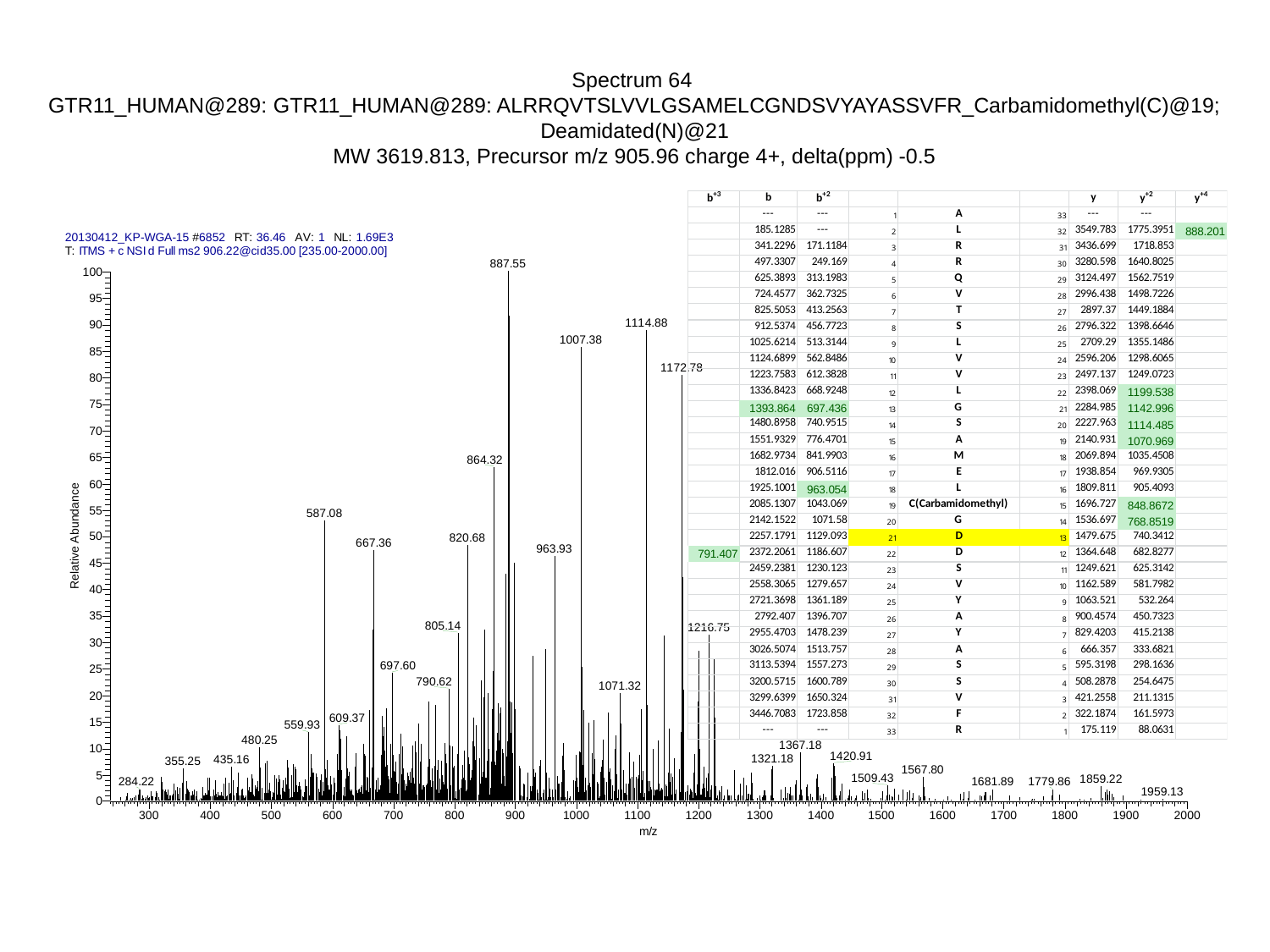

# Spectrum 64 GTR11_HUMAN@289: GTR11_HUMAN@289: ALRRQVTSLVVLGSAMELCGNDSVYAYASSVFR_Carbamidomethyl(C)@19; Deamidated(N)@21 MW 3619.813, Precursor m/z 905.96 charge 4+, delta(ppm) -0.5

## Slide 75
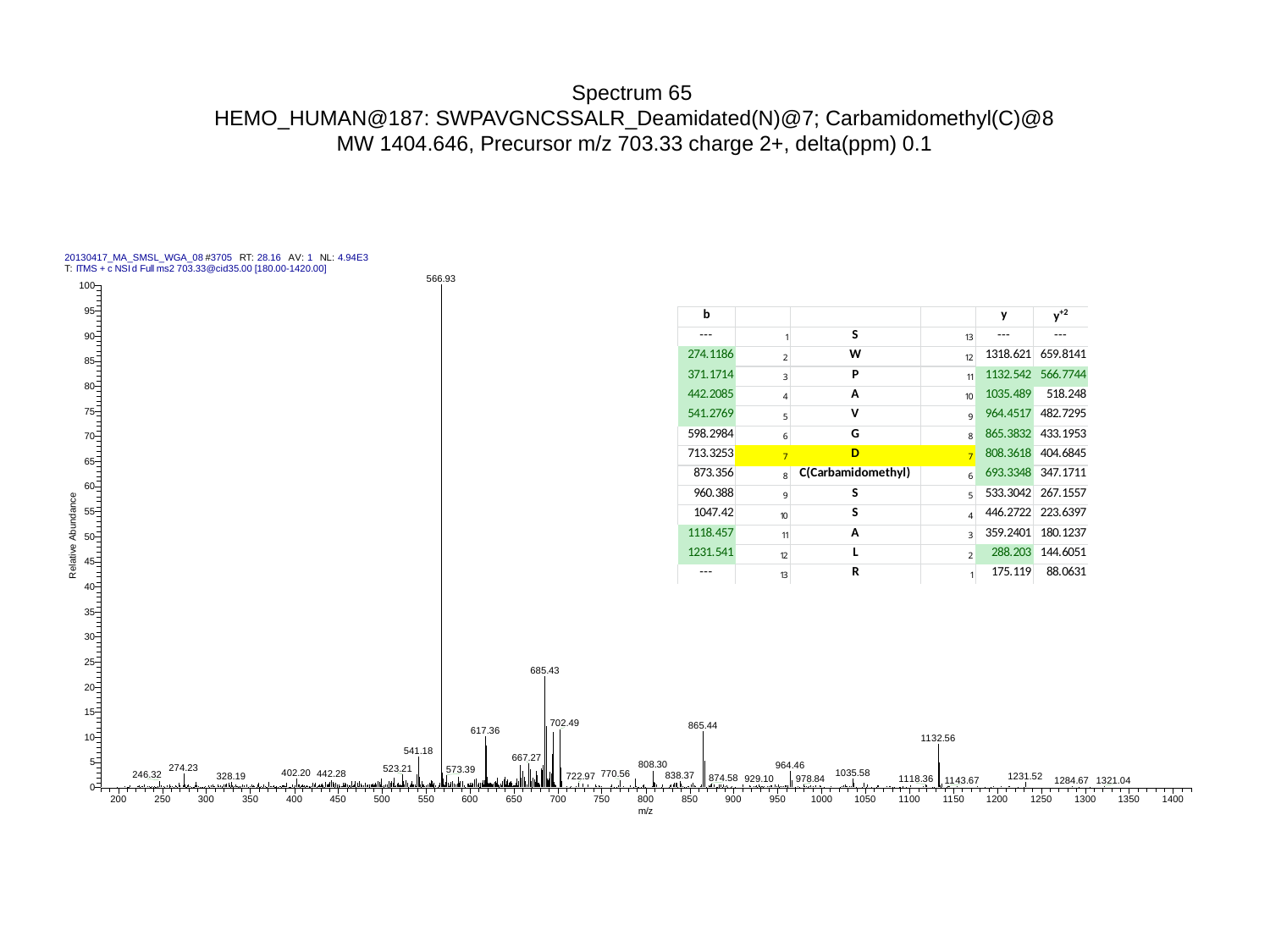

# Spectrum 65 HEMO_HUMAN@187: SWPAVGNCSSALR_Deamidated(N)@7; Carbamidomethyl(C)@8 MW 1404.646, Precursor m/z 703.33 charge 2+, delta(ppm) 0.1

## Slide 76
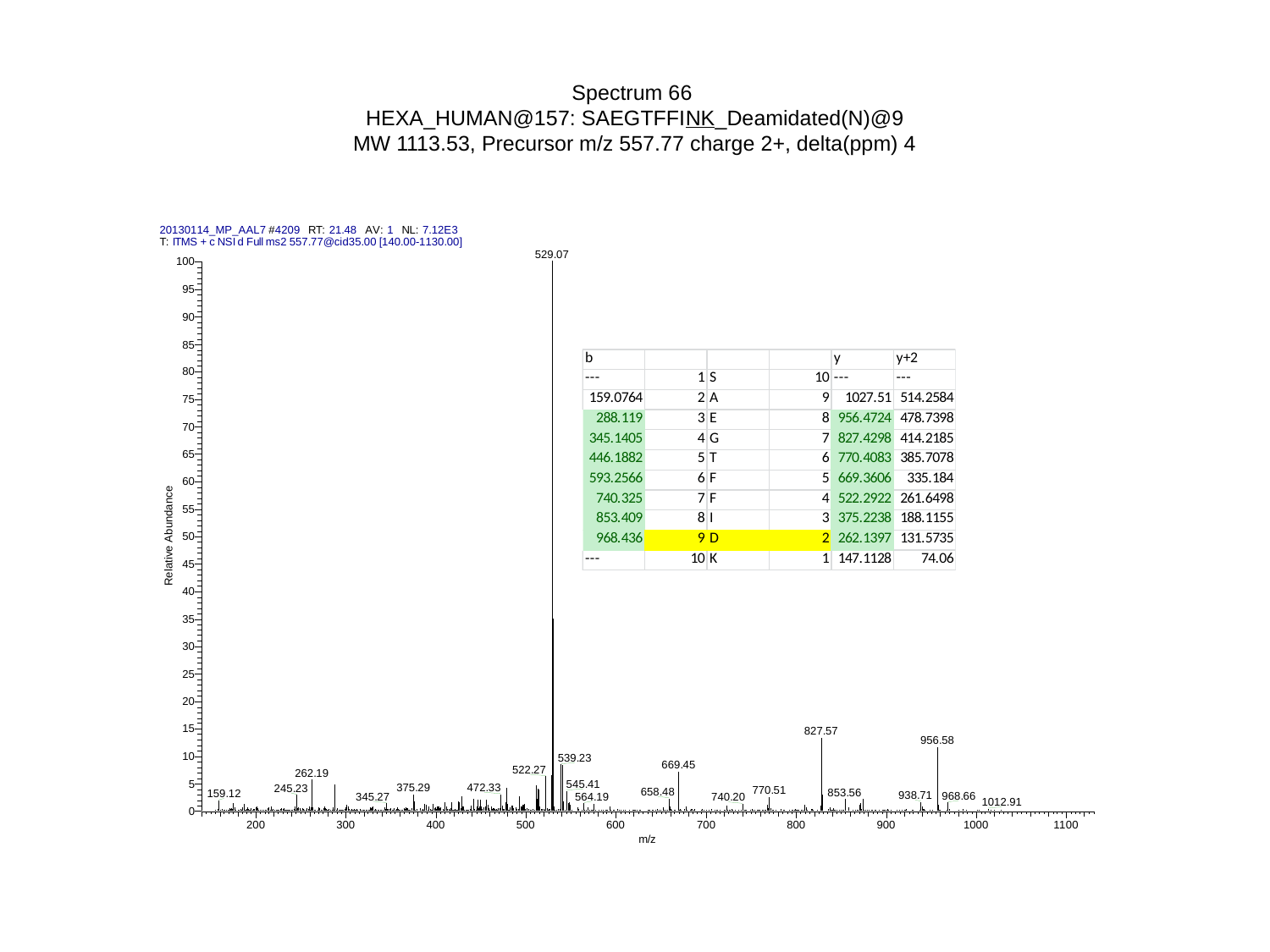

# Spectrum 66 HEXA_HUMAN@157: SAEGTFFINK_Deamidated(N)@9 MW 1113.53, Precursor m/z 557.77 charge 2+, delta(ppm) 4

## Slide 77
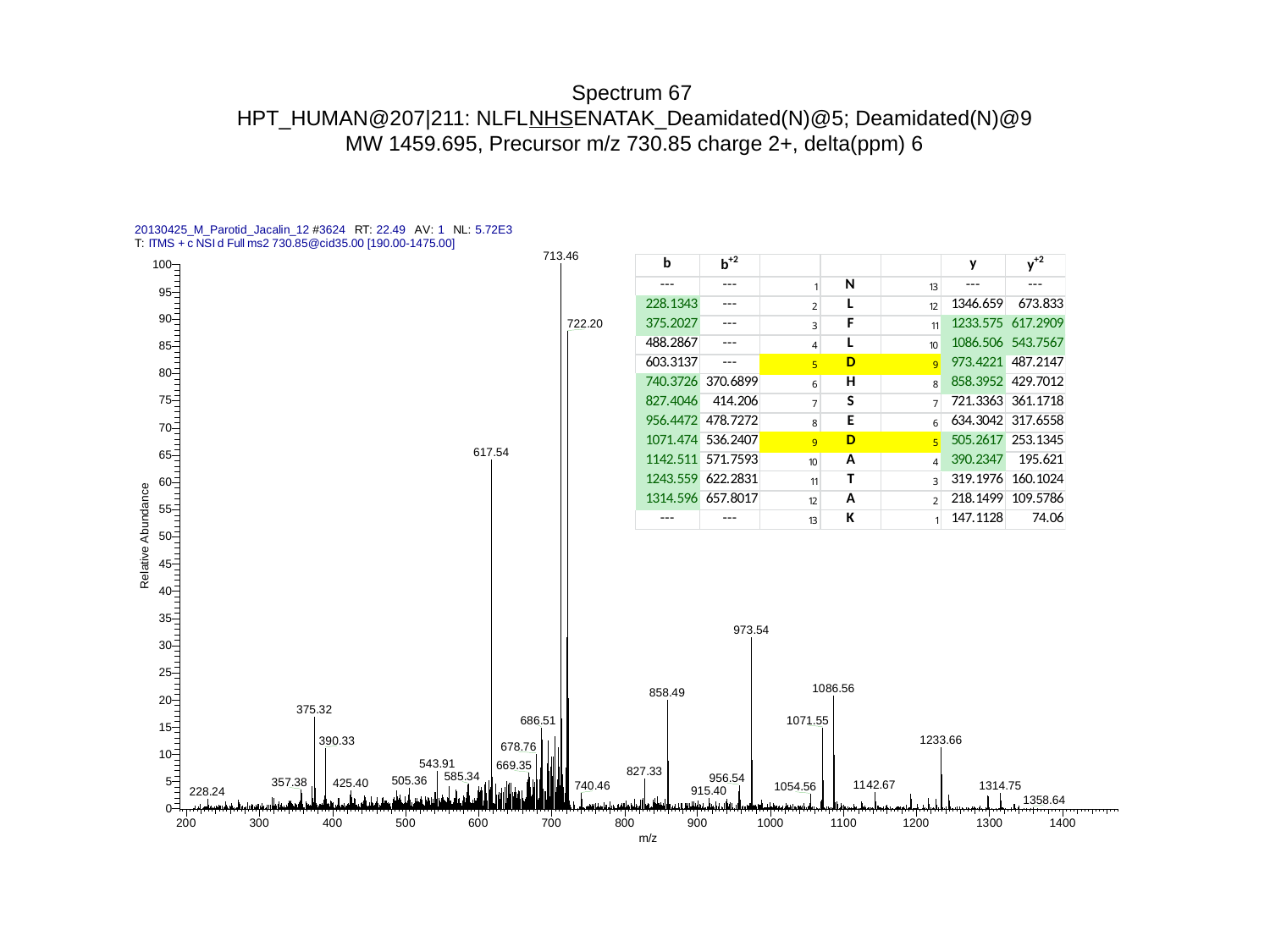

# Spectrum 67 HPT_HUMAN@207|211: NLFLNHSENATAK_Deamidated(N)@5; Deamidated(N)@9 MW 1459.695, Precursor m/z 730.85 charge 2+, delta(ppm) 6

## Slide 78
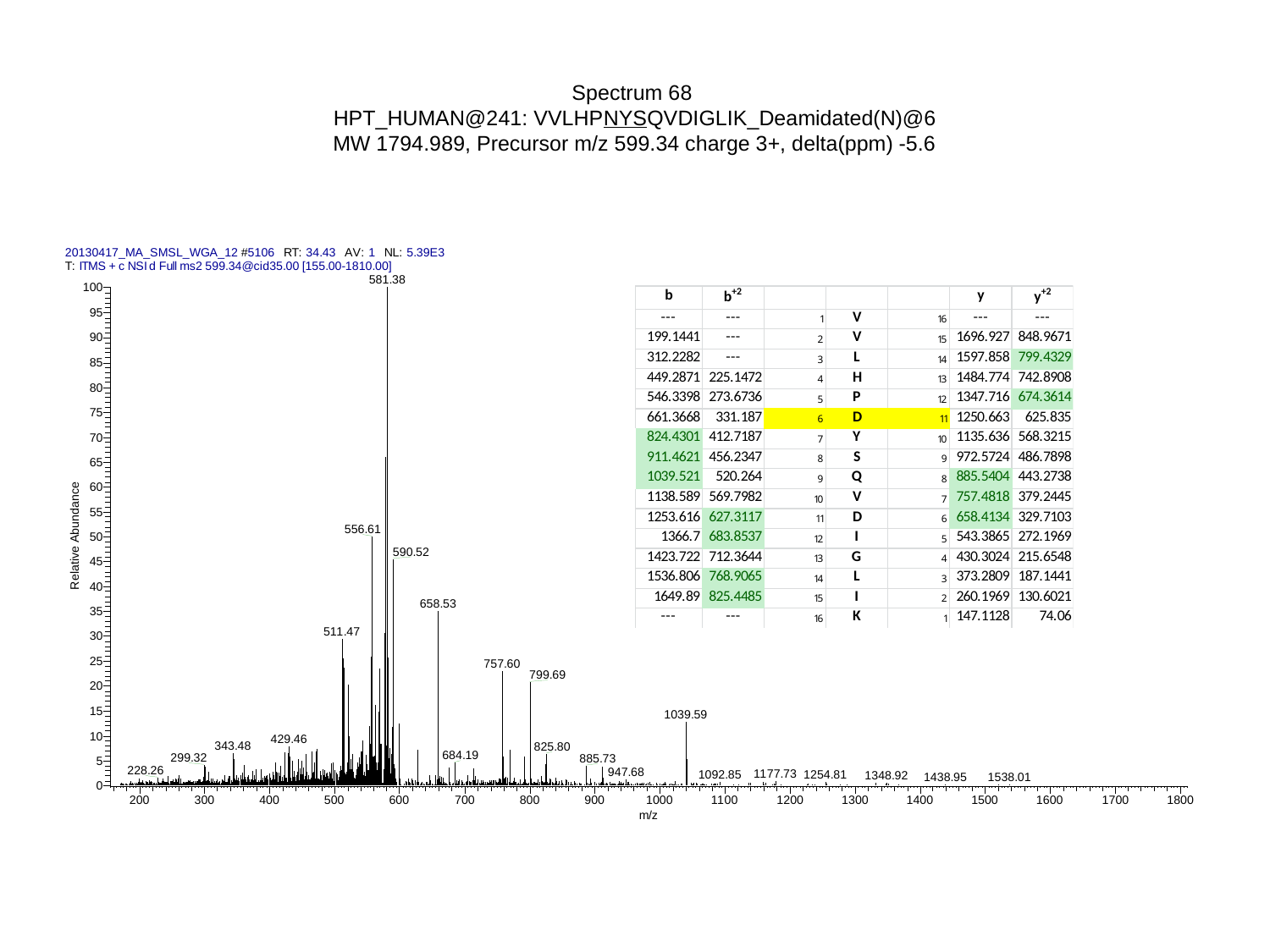

# Spectrum 68 HPT_HUMAN@241: VVLHPNYSQVDIGLIK_Deamidated(N)@6 MW 1794.989, Precursor m/z 599.34 charge 3+, delta(ppm) -5.6

## Slide 79
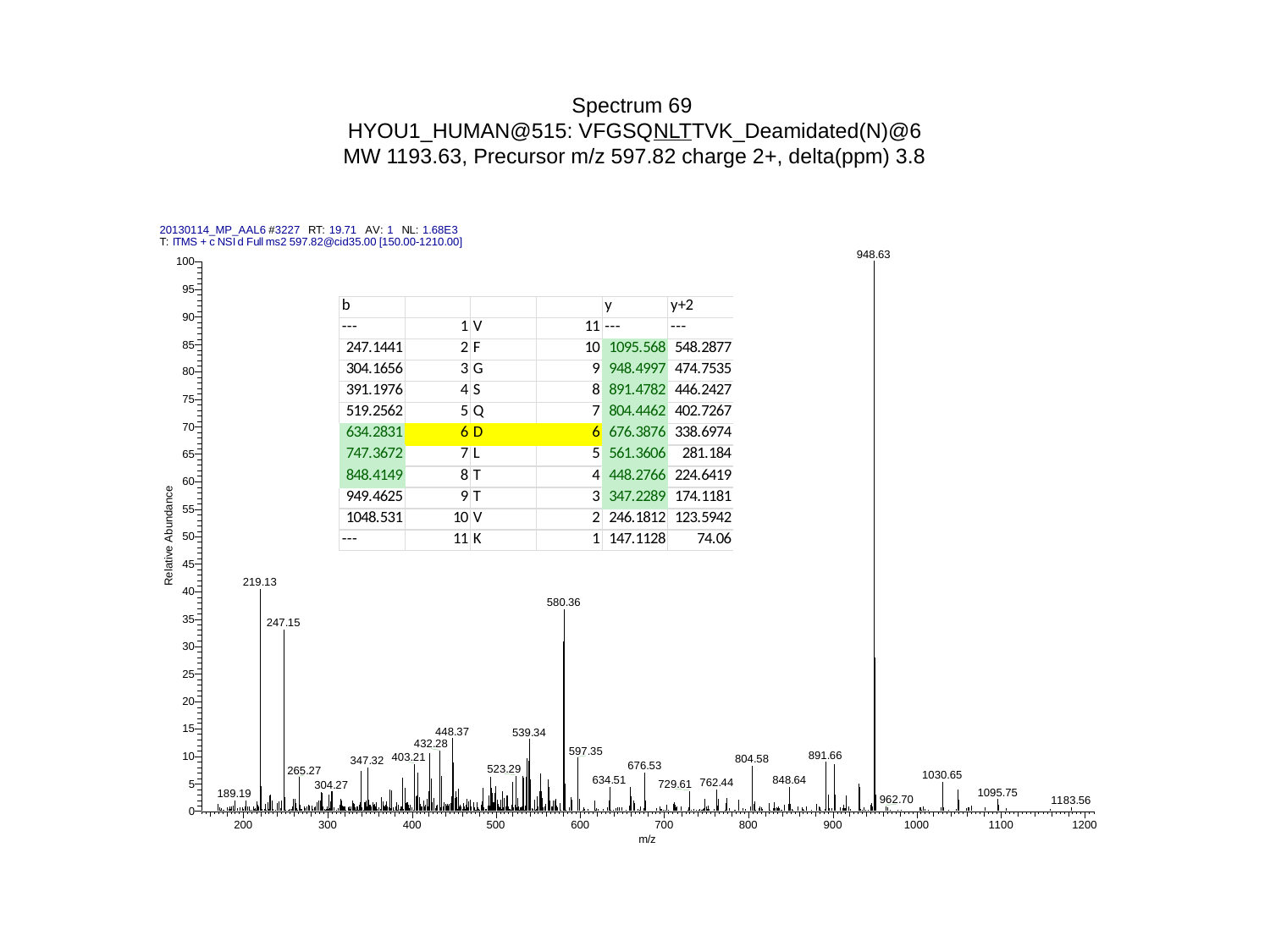

# Spectrum 69 HYOU1_HUMAN@515: VFGSQNLTTVK_Deamidated(N)@6 MW 1193.63, Precursor m/z 597.82 charge 2+, delta(ppm) 3.8

## Slide 80
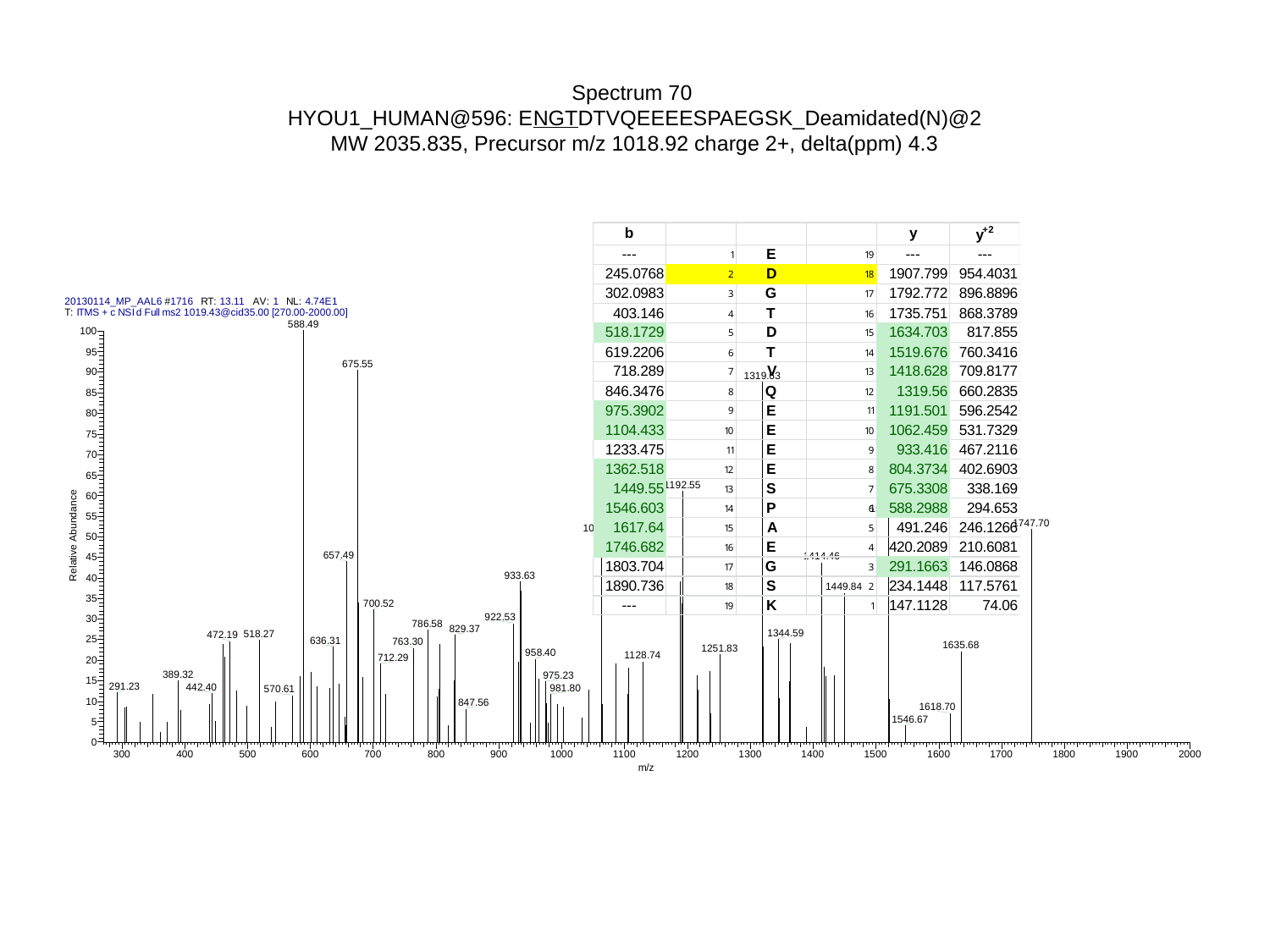

# Spectrum 70 HYOU1_HUMAN@596: ENGTDTVQEEEESPAEGSK_Deamidated(N)@2 MW 2035.835, Precursor m/z 1018.92 charge 2+, delta(ppm) 4.3

## Slide 81
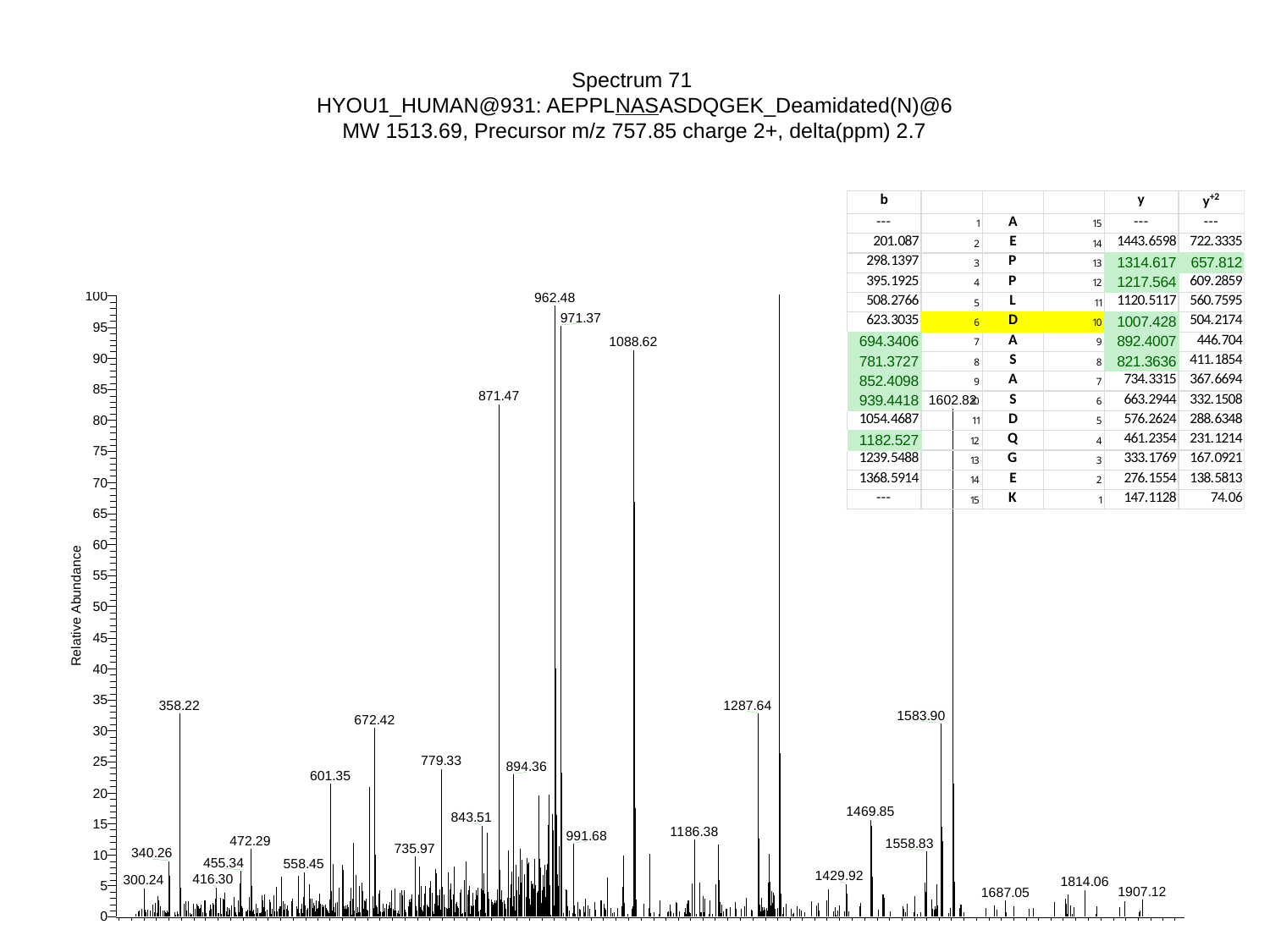

# Spectrum 71 HYOU1_HUMAN@931: AEPPLNASASDQGEK_Deamidated(N)@6 MW 1513.69, Precursor m/z 757.85 charge 2+, delta(ppm) 2.7

## Slide 82
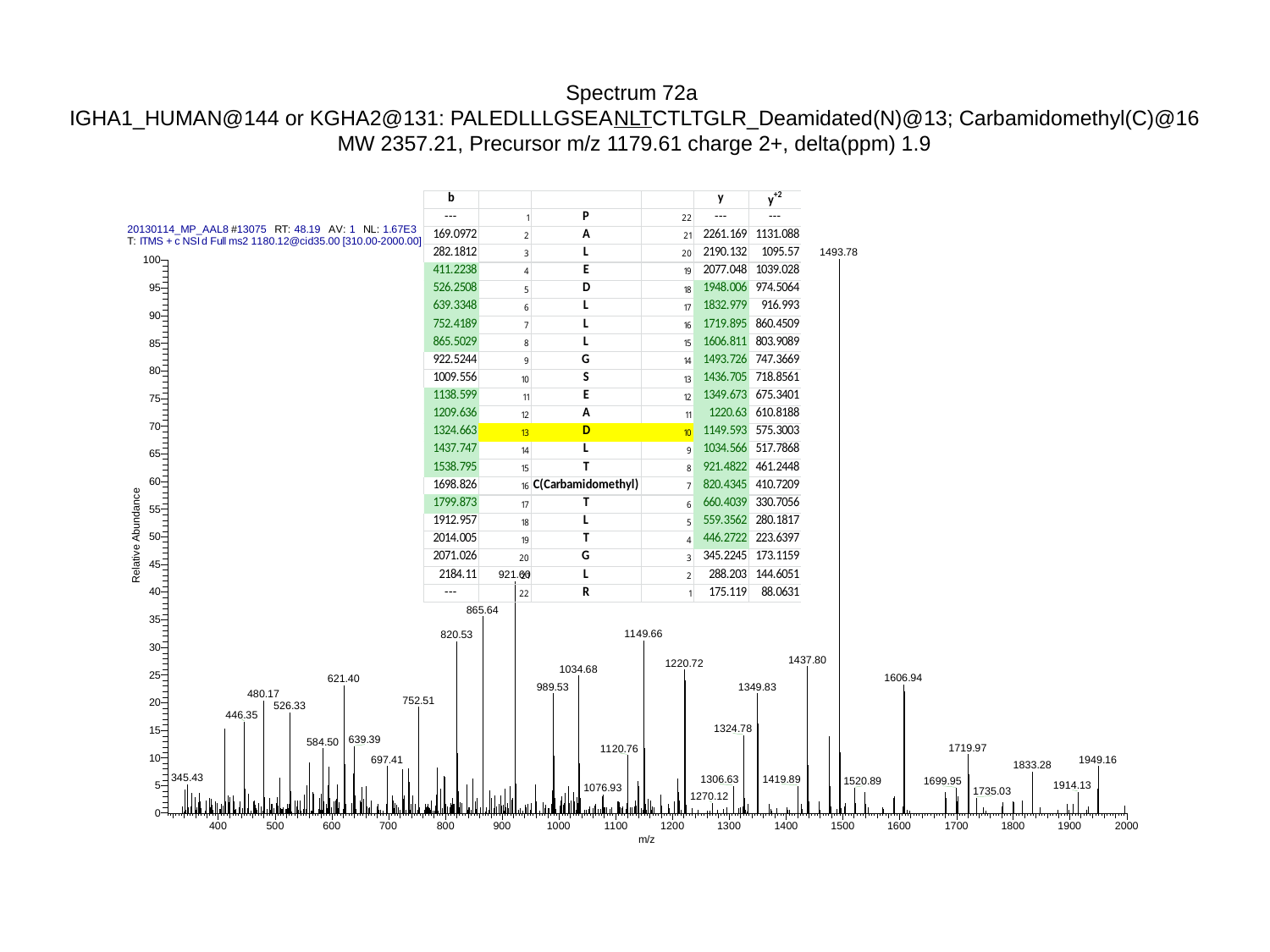

# Spectrum 72a IGHA1_HUMAN@144 or KGHA2@131: PALEDLLLGSEANLTCTLTGLR_Deamidated(N)@13; Carbamidomethyl(C)@16 MW 2357.21, Precursor m/z 1179.61 charge 2+, delta(ppm) 1.9

## Slide 83
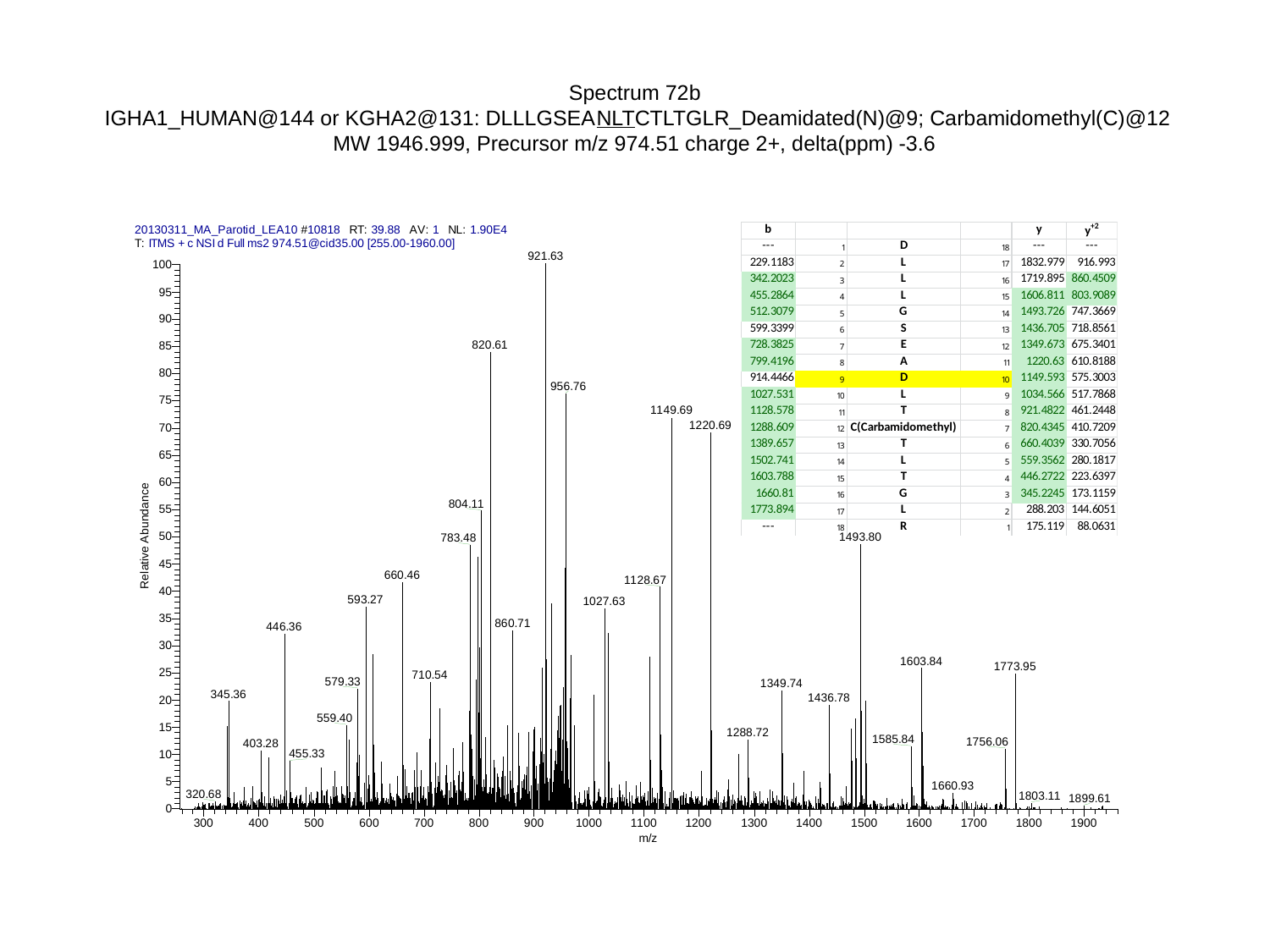

# Spectrum 72b IGHA1_HUMAN@144 or KGHA2@131: DLLLGSEANLTCTLTGLR_Deamidated(N)@9; Carbamidomethyl(C)@12 MW 1946.999, Precursor m/z 974.51 charge 2+, delta(ppm) -3.6

## Slide 84
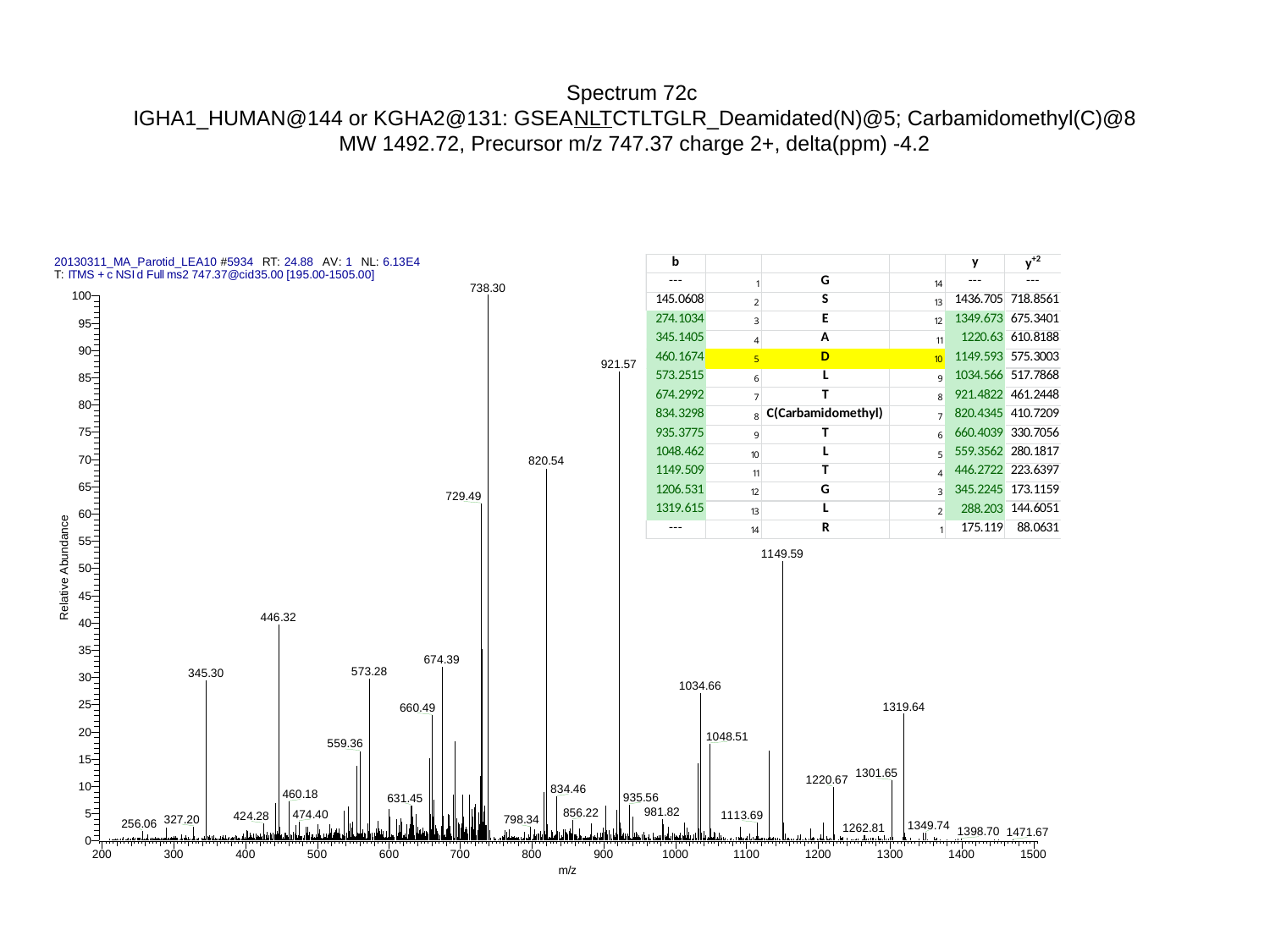

# Spectrum 72c IGHA1_HUMAN@144 or KGHA2@131: GSEANLTCTLTGLR_Deamidated(N)@5; Carbamidomethyl(C)@8 MW 1492.72, Precursor m/z 747.37 charge 2+, delta(ppm) -4.2

## Slide 85
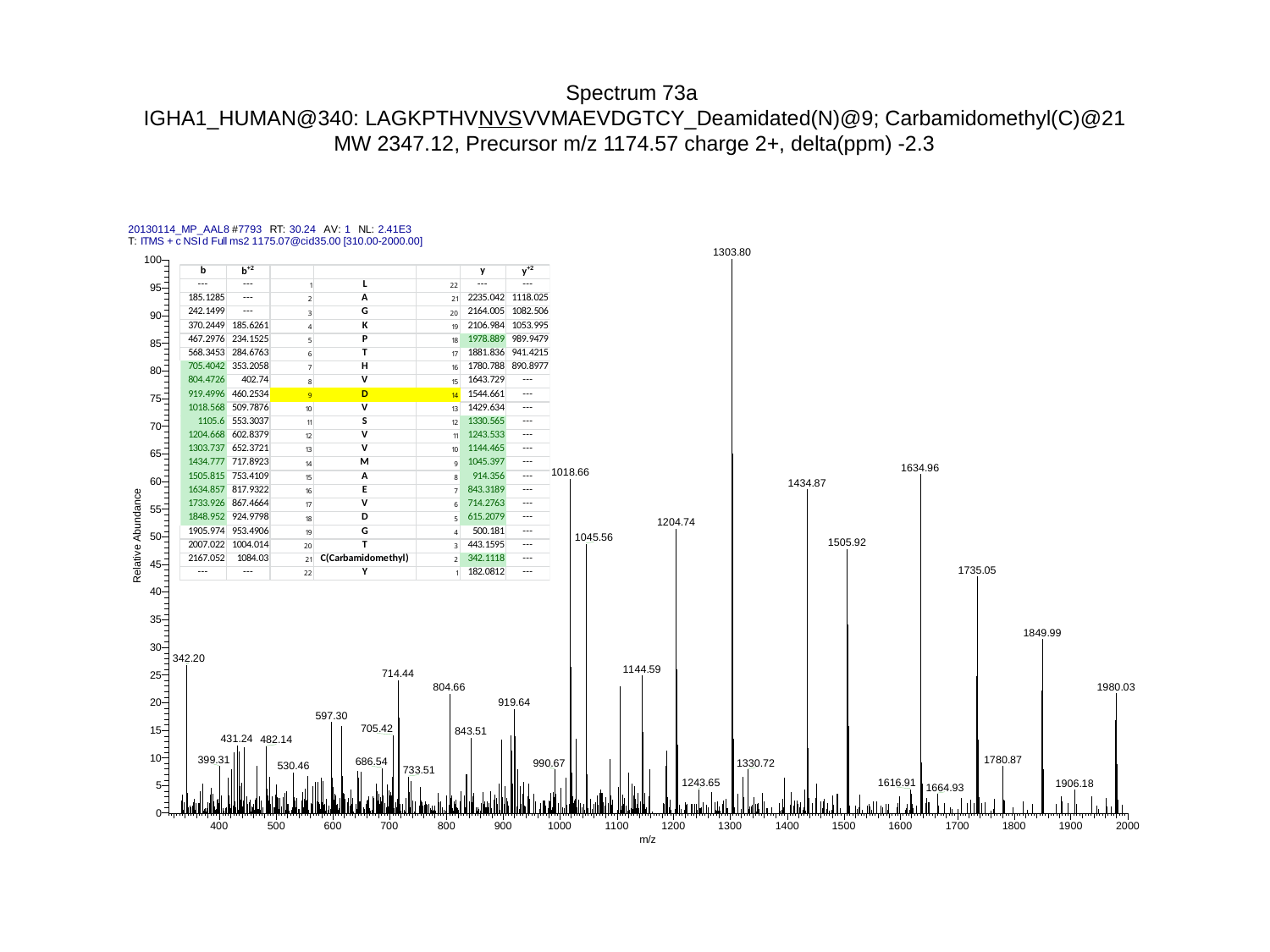

# Spectrum 73a IGHA1_HUMAN@340: LAGKPTHVNVSVVMAEVDGTCY_Deamidated(N)@9; Carbamidomethyl(C)@21 MW 2347.12, Precursor m/z 1174.57 charge 2+, delta(ppm) -2.3

## Slide 86
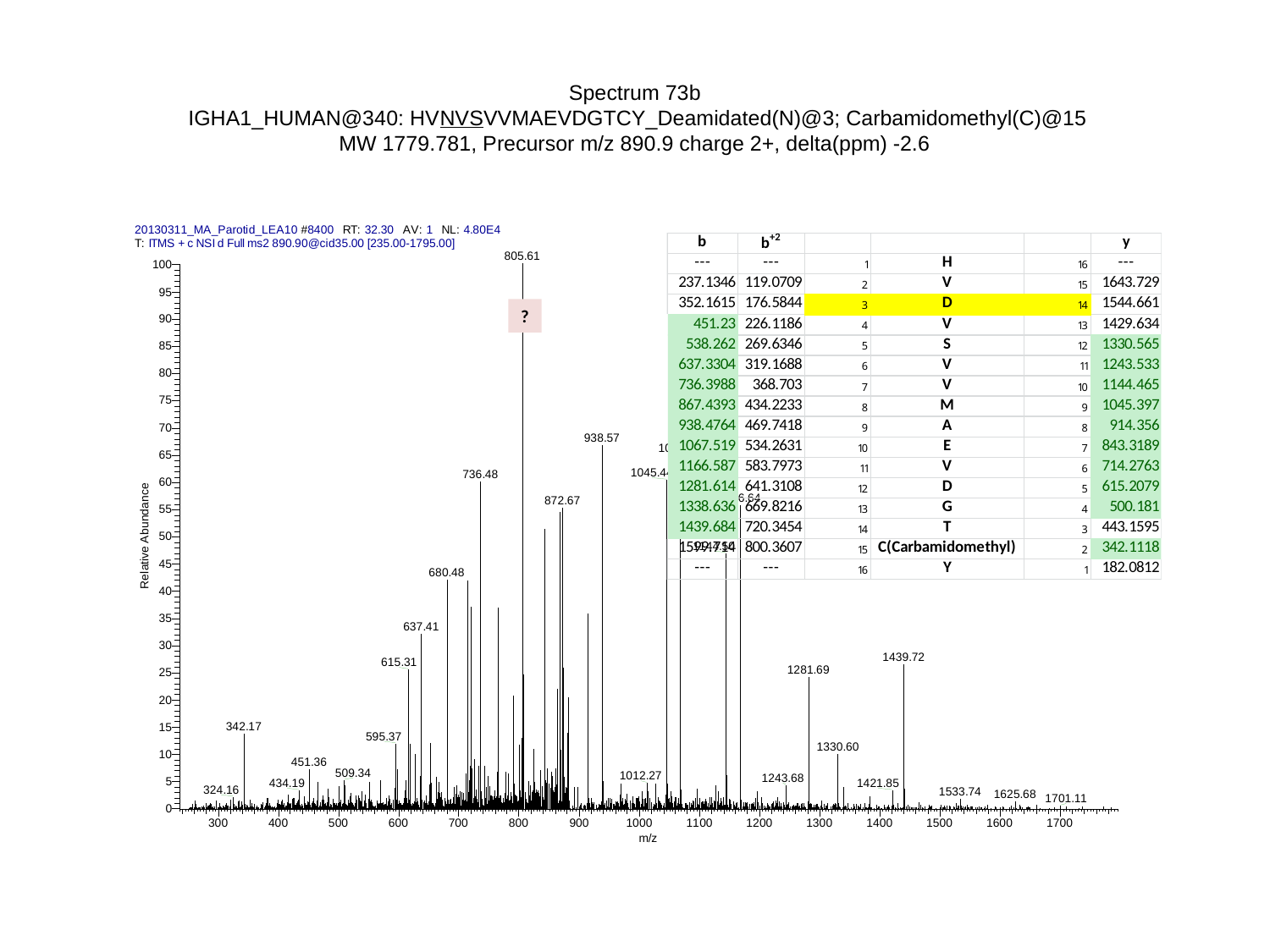

# Spectrum 73b IGHA1_HUMAN@340: HVNVSVVMAEVDGTCY_Deamidated(N)@3; Carbamidomethyl(C)@15 MW 1779.781, Precursor m/z 890.9 charge 2+, delta(ppm) -2.6
?

## Slide 87
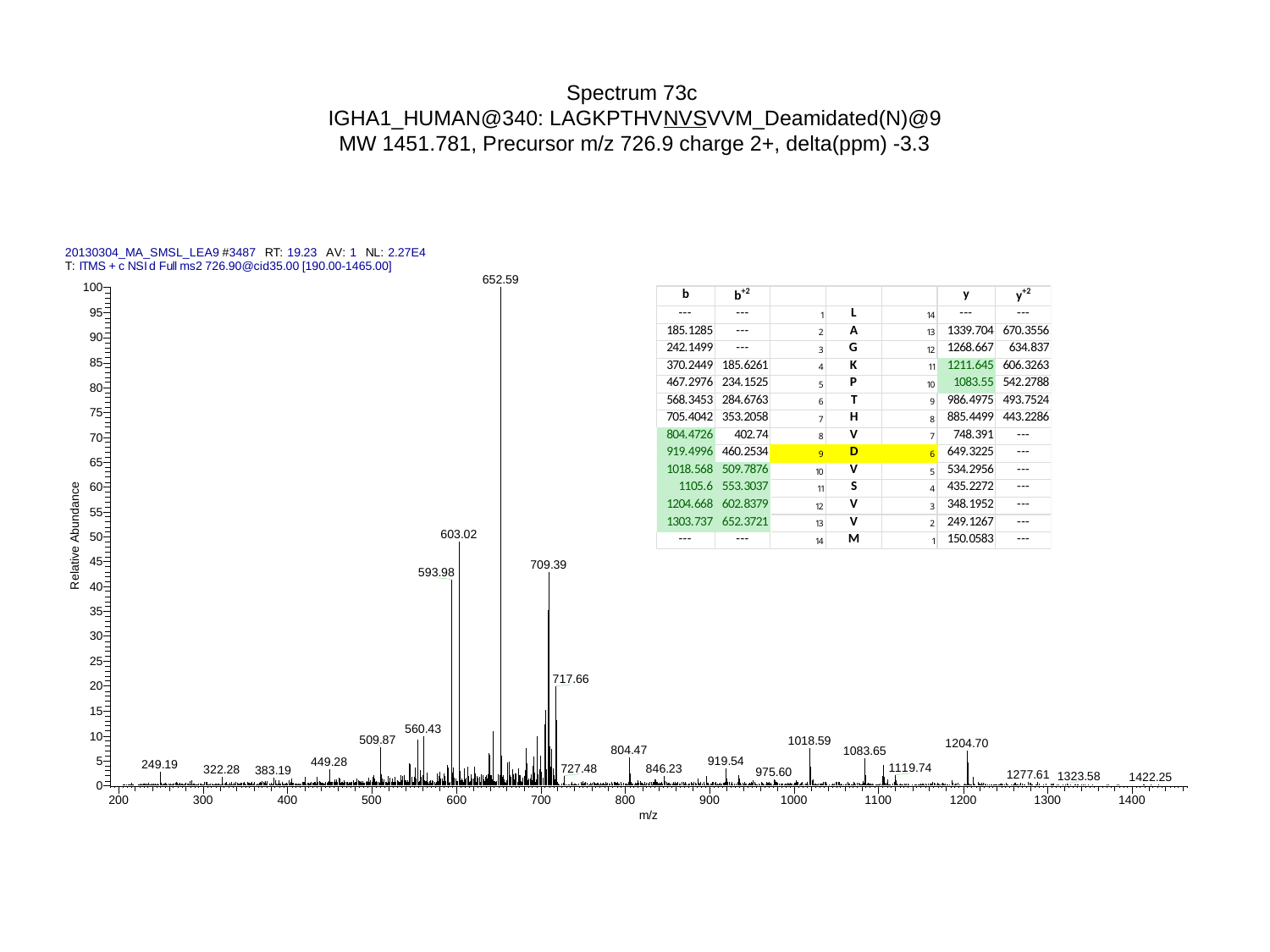

# Spectrum 73c IGHA1_HUMAN@340: LAGKPTHVNVSVVM_Deamidated(N)@9 MW 1451.781, Precursor m/z 726.9 charge 2+, delta(ppm) -3.3

## Slide 88
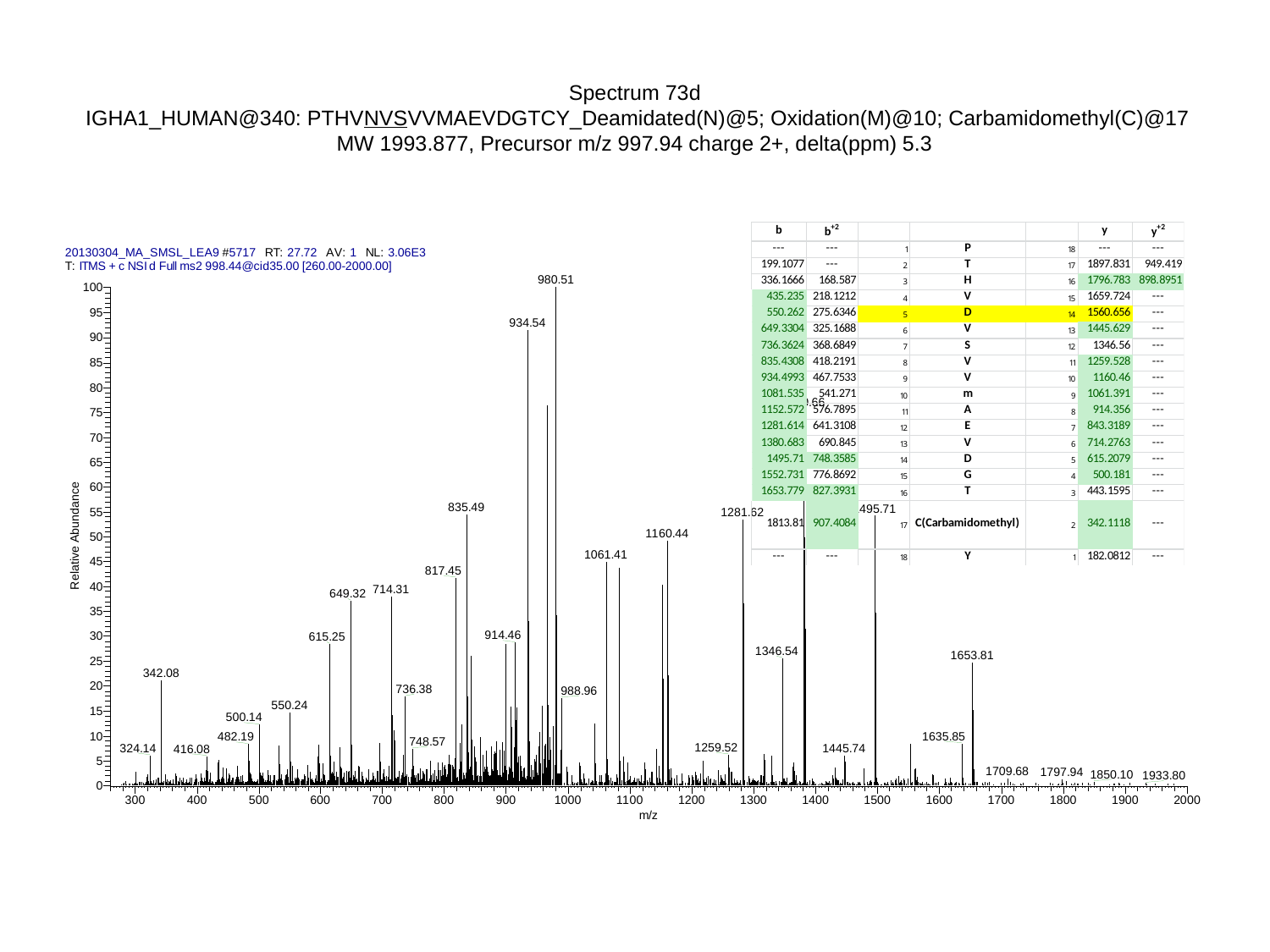

# Spectrum 73d IGHA1_HUMAN@340: PTHVNVSVVMAEVDGTCY_Deamidated(N)@5; Oxidation(M)@10; Carbamidomethyl(C)@17 MW 1993.877, Precursor m/z 997.94 charge 2+, delta(ppm) 5.3

## Slide 89
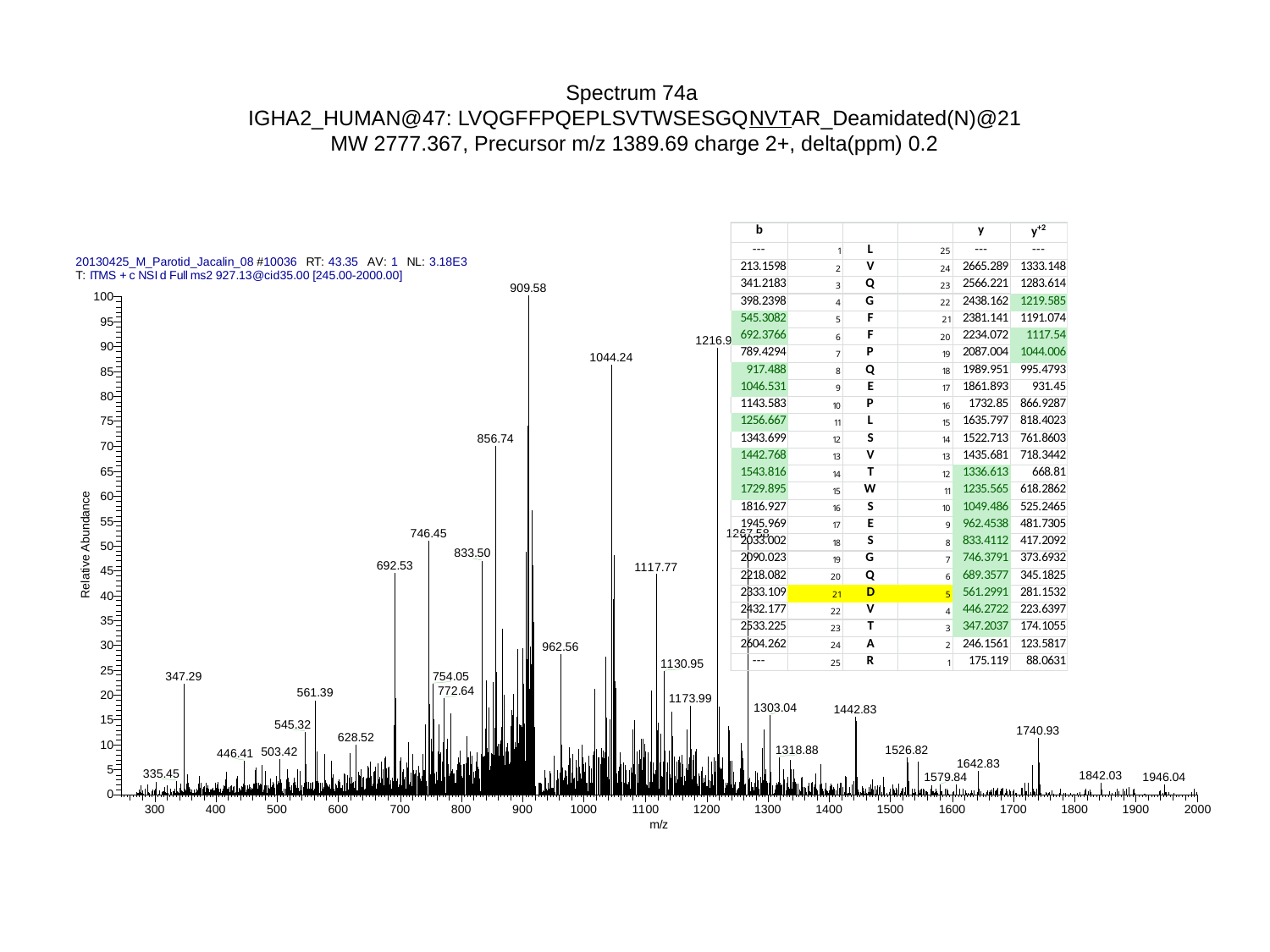

# Spectrum 74a IGHA2_HUMAN@47: LVQGFFPQEPLSVTWSESGQNVTAR_Deamidated(N)@21 MW 2777.367, Precursor m/z 1389.69 charge 2+, delta(ppm) 0.2

## Slide 90
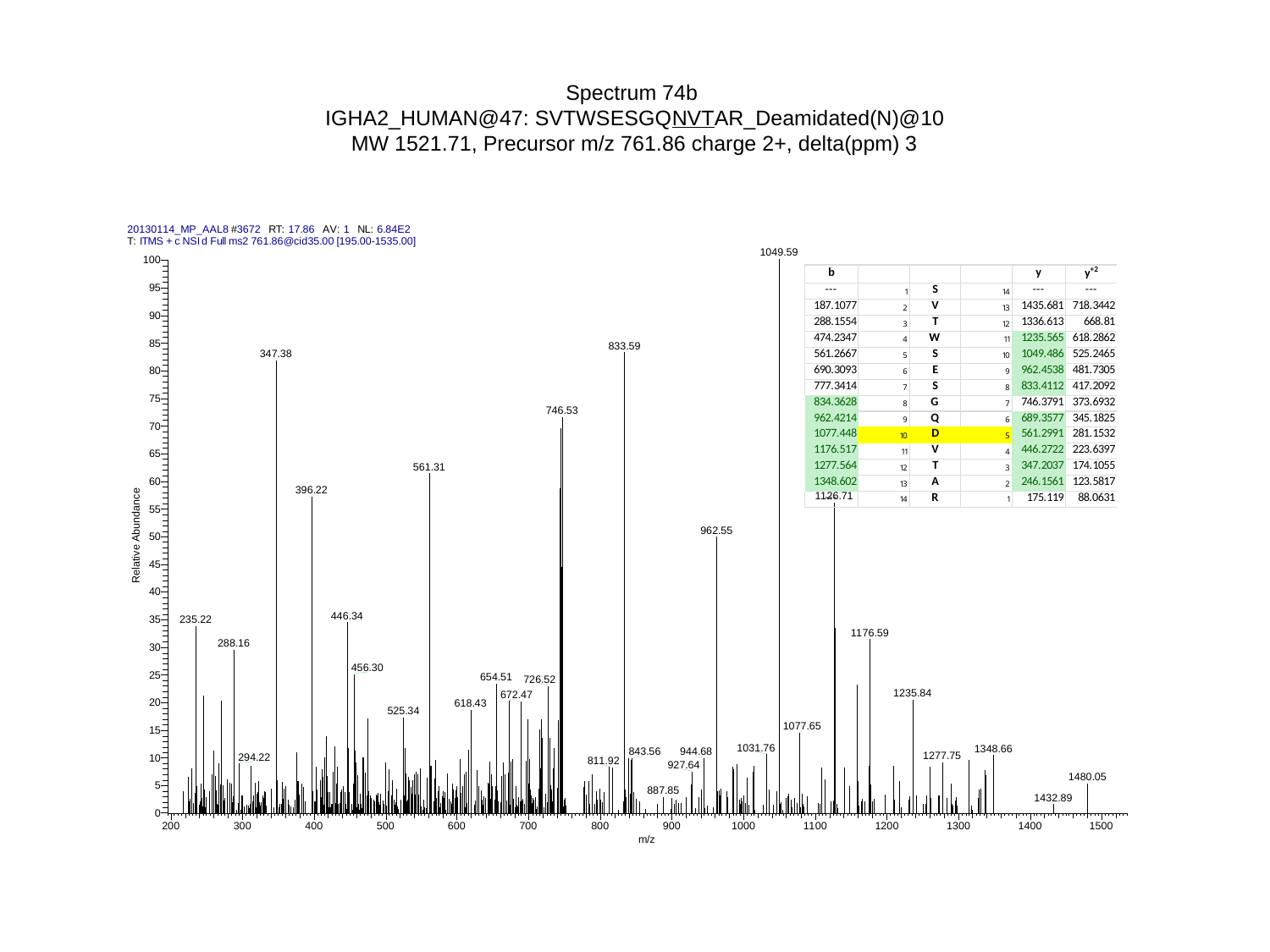

# Spectrum 74b IGHA2_HUMAN@47: SVTWSESGQNVTAR_Deamidated(N)@10 MW 1521.71, Precursor m/z 761.86 charge 2+, delta(ppm) 3

## Slide 91
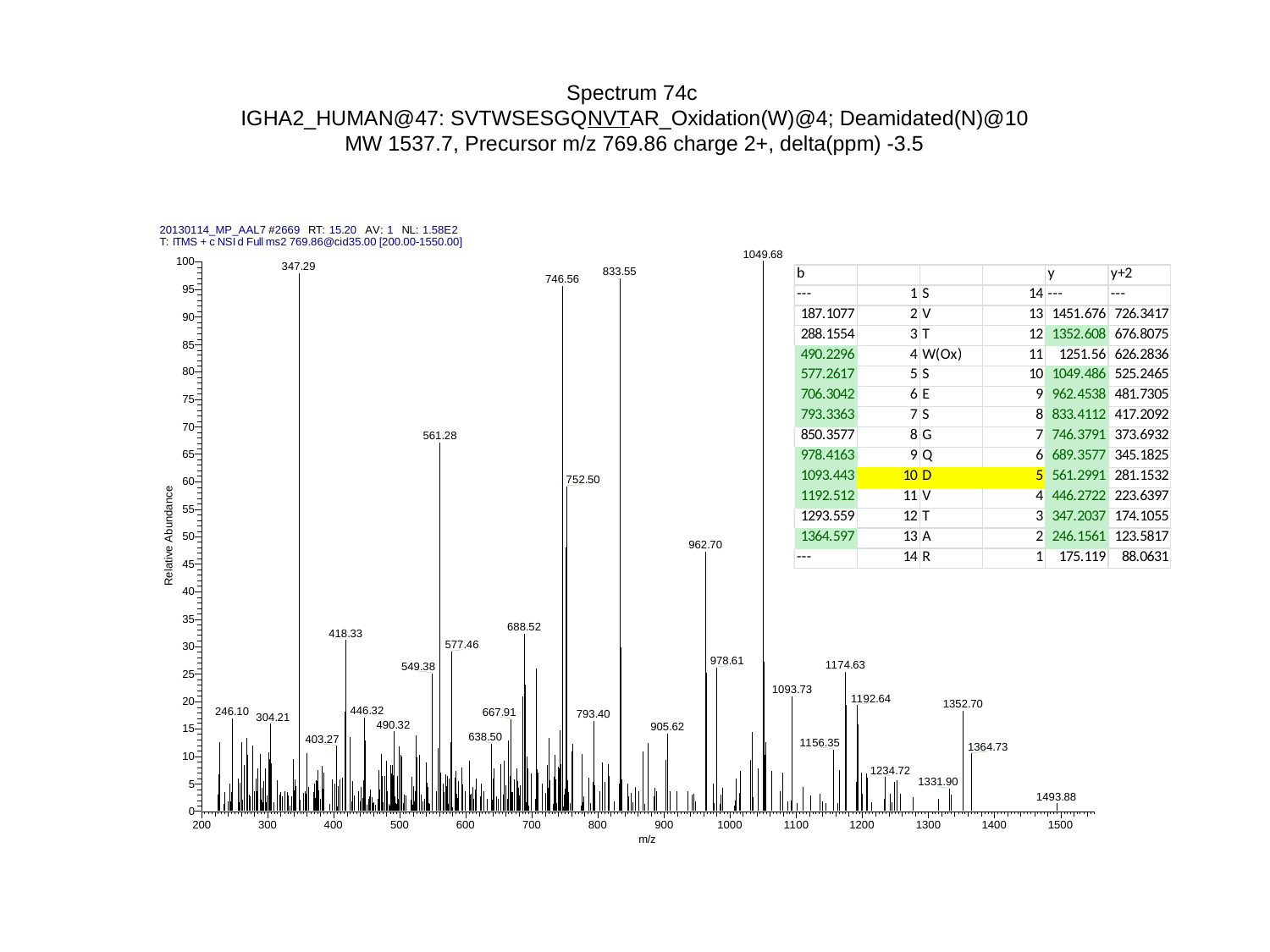

# Spectrum 74c IGHA2_HUMAN@47: SVTWSESGQNVTAR_Oxidation(W)@4; Deamidated(N)@10 MW 1537.7, Precursor m/z 769.86 charge 2+, delta(ppm) -3.5

## Slide 92
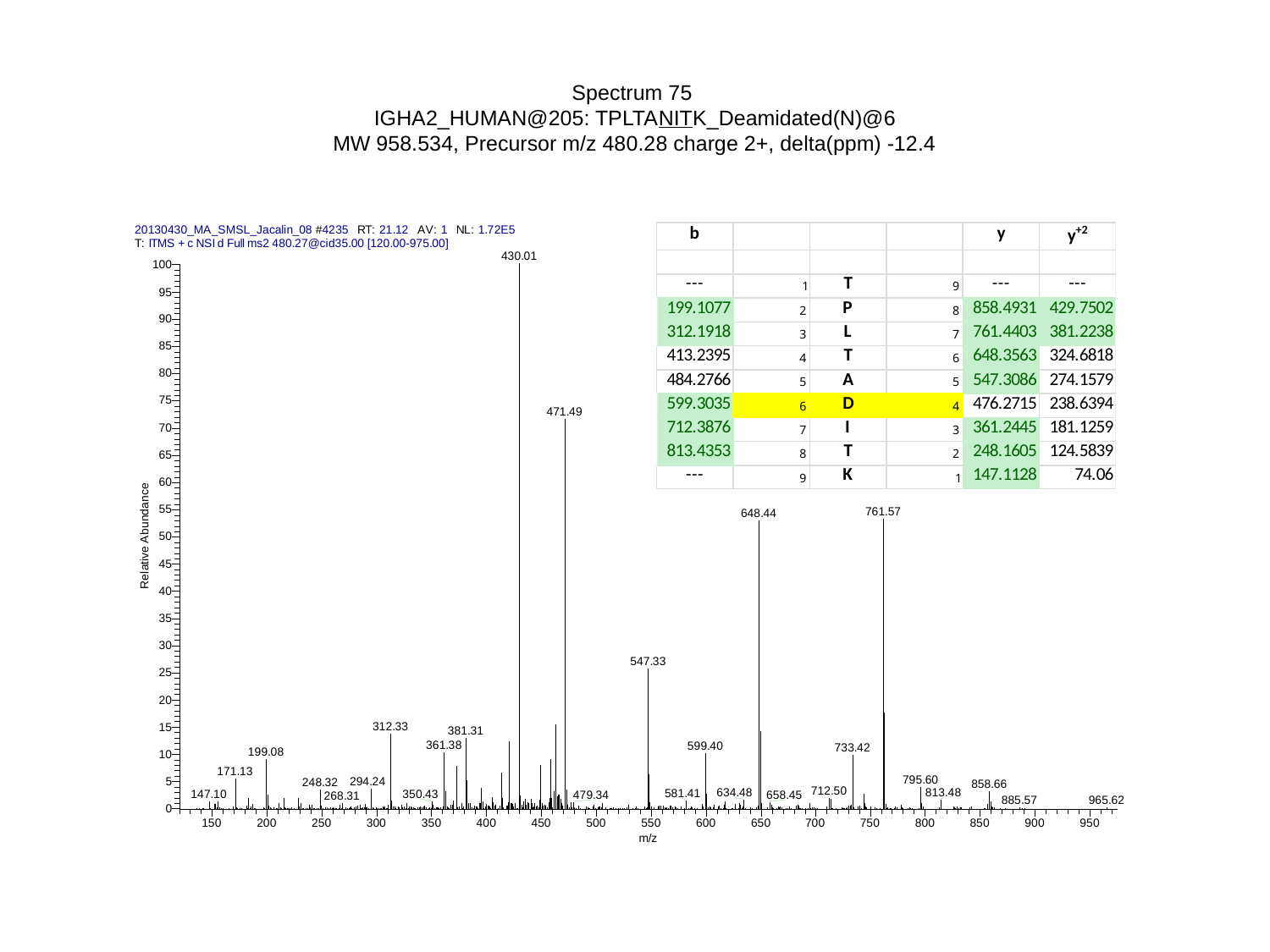

# Spectrum 75 IGHA2_HUMAN@205: TPLTANITK_Deamidated(N)@6 MW 958.534, Precursor m/z 480.28 charge 2+, delta(ppm) -12.4

## Slide 93
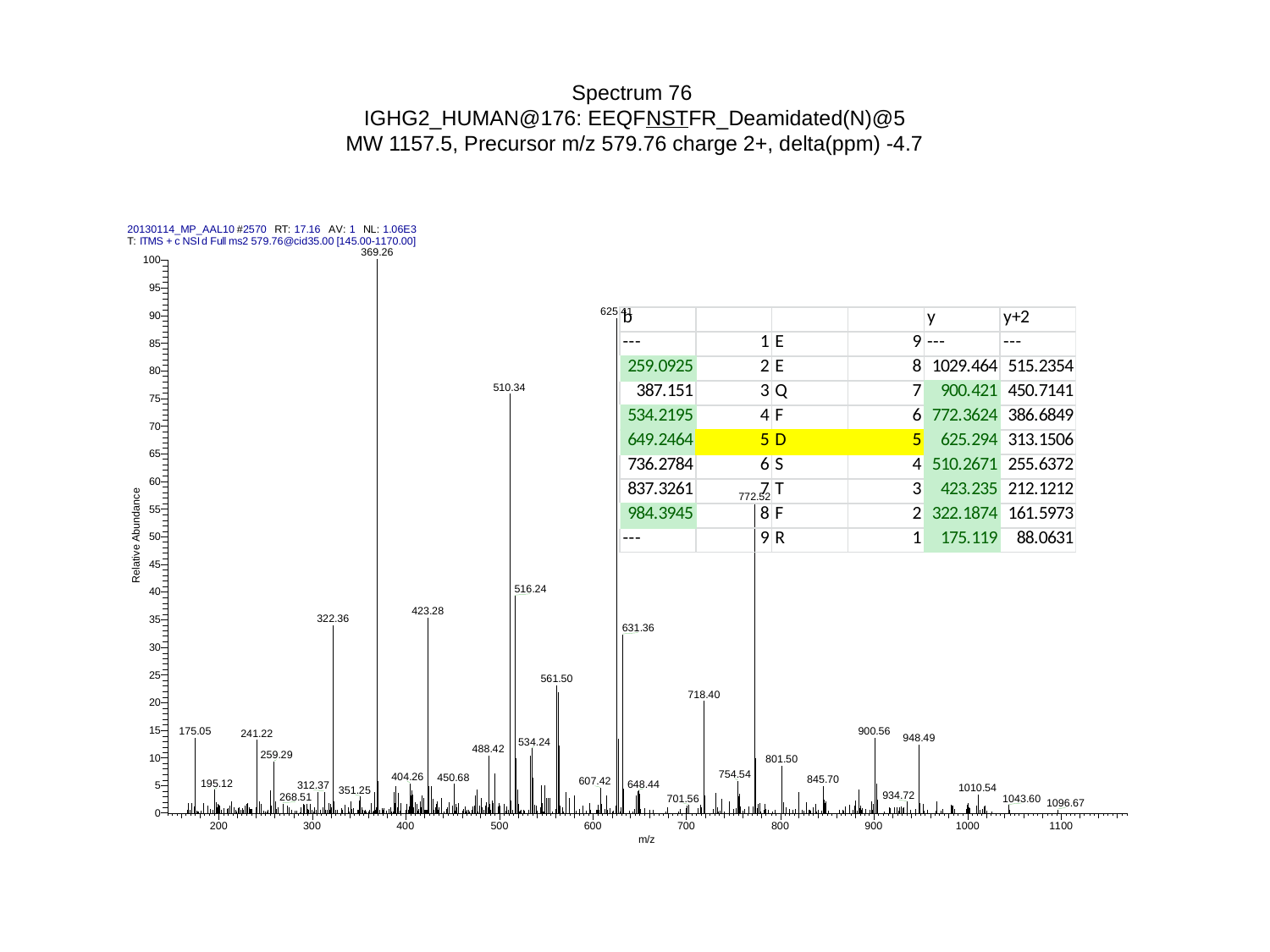

# Spectrum 76 IGHG2_HUMAN@176: EEQFNSTFR_Deamidated(N)@5 MW 1157.5, Precursor m/z 579.76 charge 2+, delta(ppm) -4.7

## Slide 94
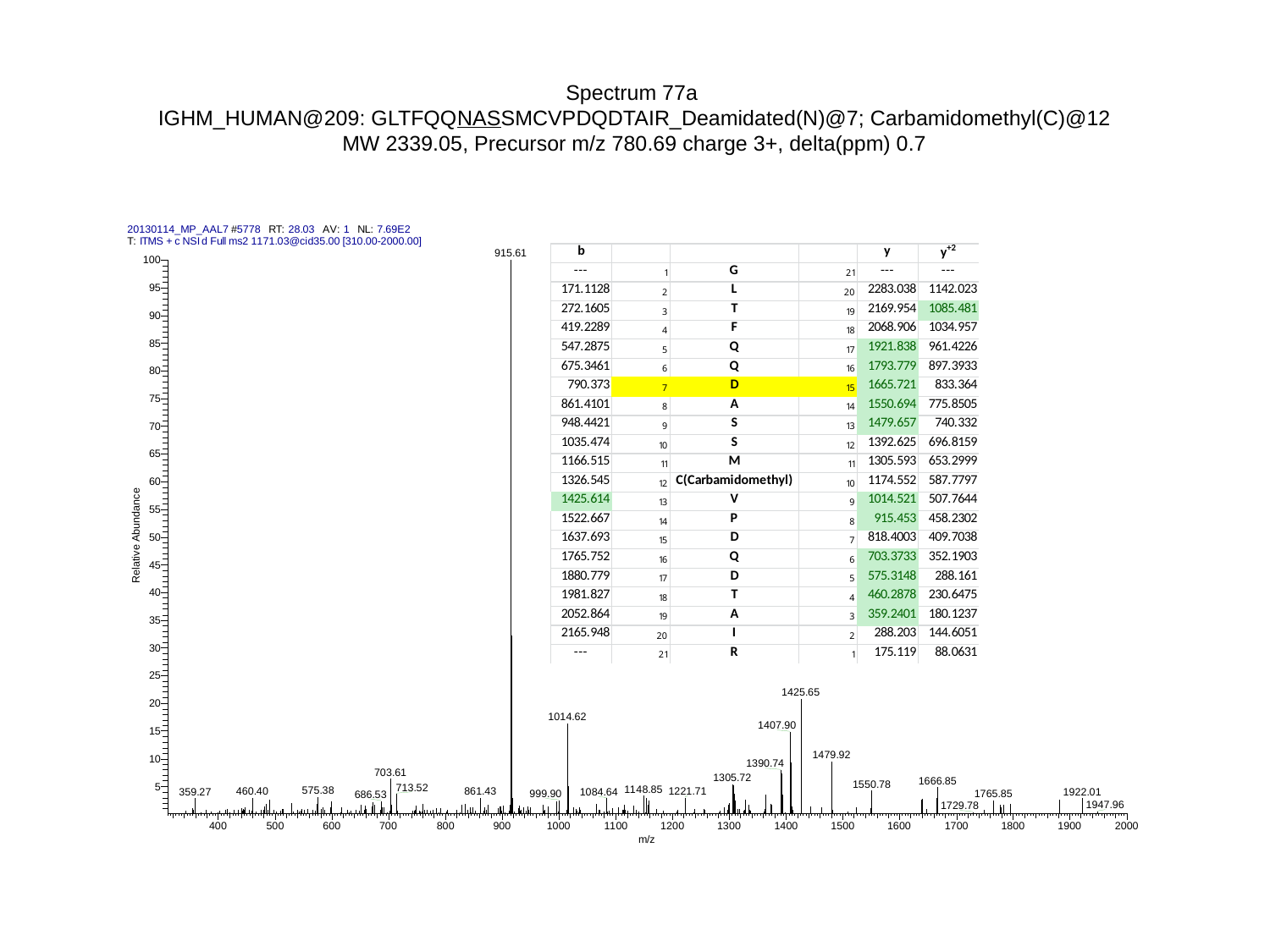

# Spectrum 77a IGHM_HUMAN@209: GLTFQQNASSMCVPDQDTAIR_Deamidated(N)@7; Carbamidomethyl(C)@12 MW 2339.05, Precursor m/z 780.69 charge 3+, delta(ppm) 0.7

## Slide 95
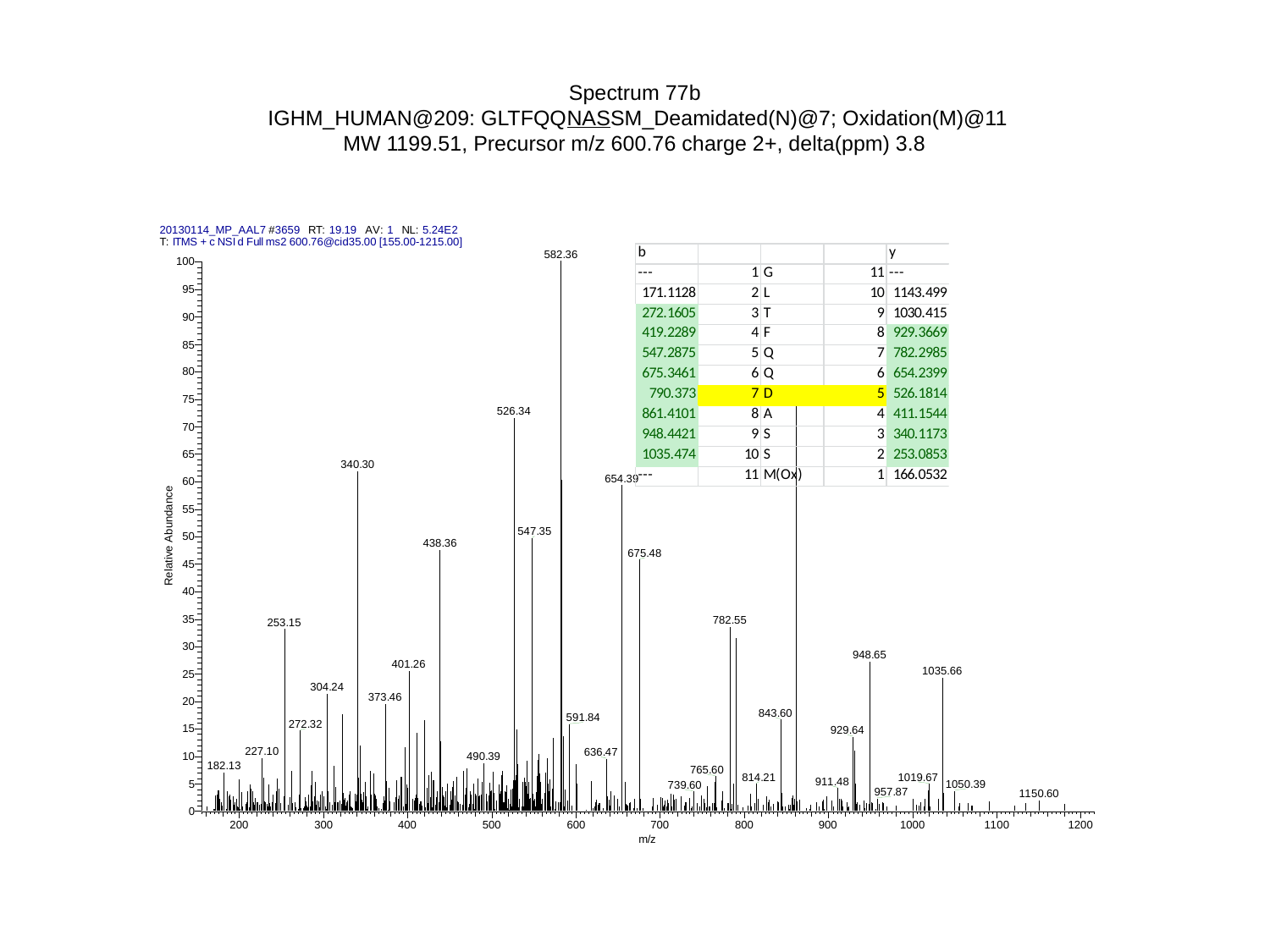

# Spectrum 77b IGHM_HUMAN@209: GLTFQQNASSM_Deamidated(N)@7; Oxidation(M)@11 MW 1199.51, Precursor m/z 600.76 charge 2+, delta(ppm) 3.8

## Slide 96
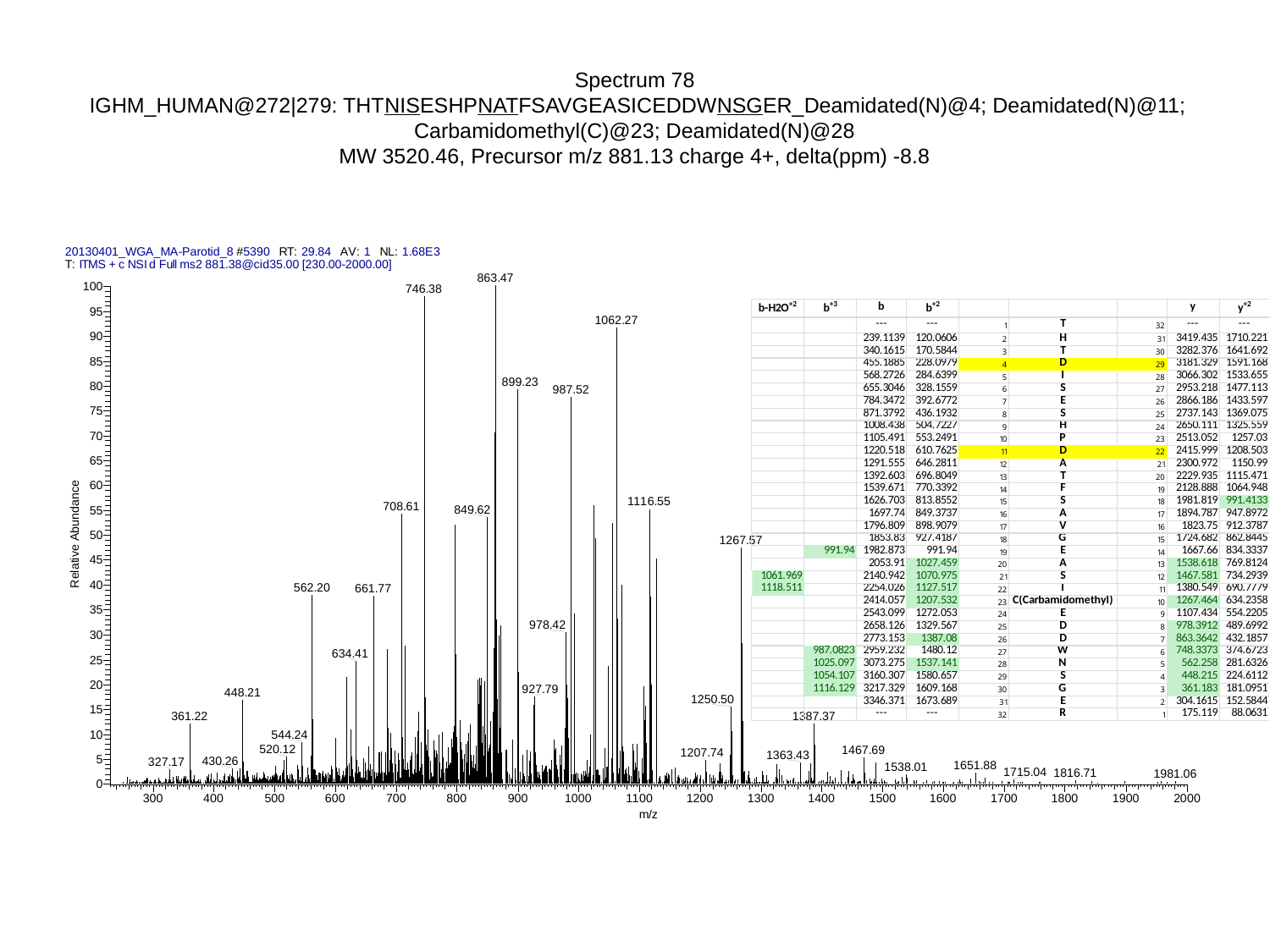

# Spectrum 78 IGHM_HUMAN@272|279: THTNISESHPNATFSAVGEASICEDDWNSGER_Deamidated(N)@4; Deamidated(N)@11; Carbamidomethyl(C)@23; Deamidated(N)@28 MW 3520.46, Precursor m/z 881.13 charge 4+, delta(ppm) -8.8

## Slide 97
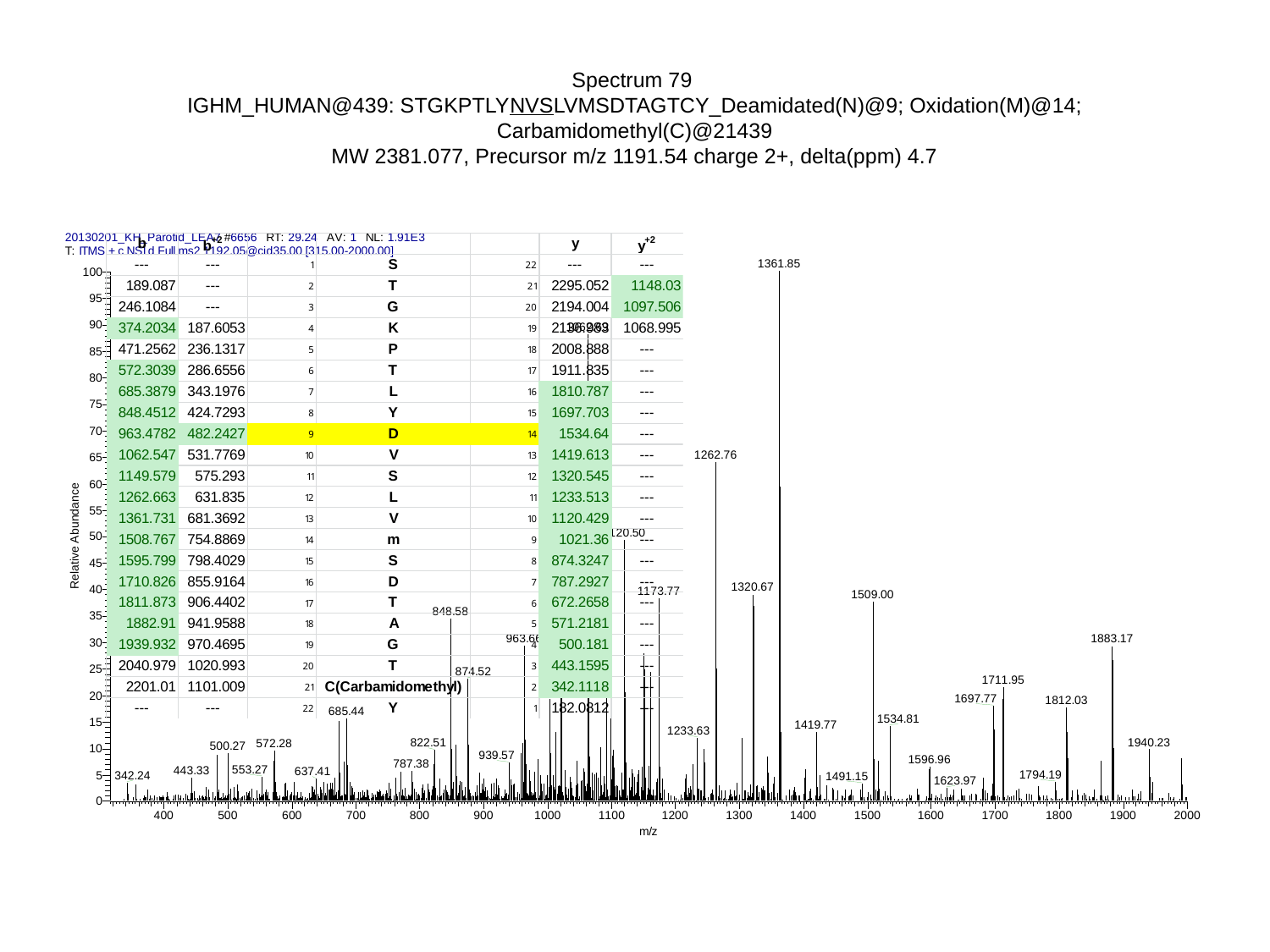

# Spectrum 79 IGHM_HUMAN@439: STGKPTLYNVSLVMSDTAGTCY_Deamidated(N)@9; Oxidation(M)@14; Carbamidomethyl(C)@21439 MW 2381.077, Precursor m/z 1191.54 charge 2+, delta(ppm) 4.7

## Slide 98
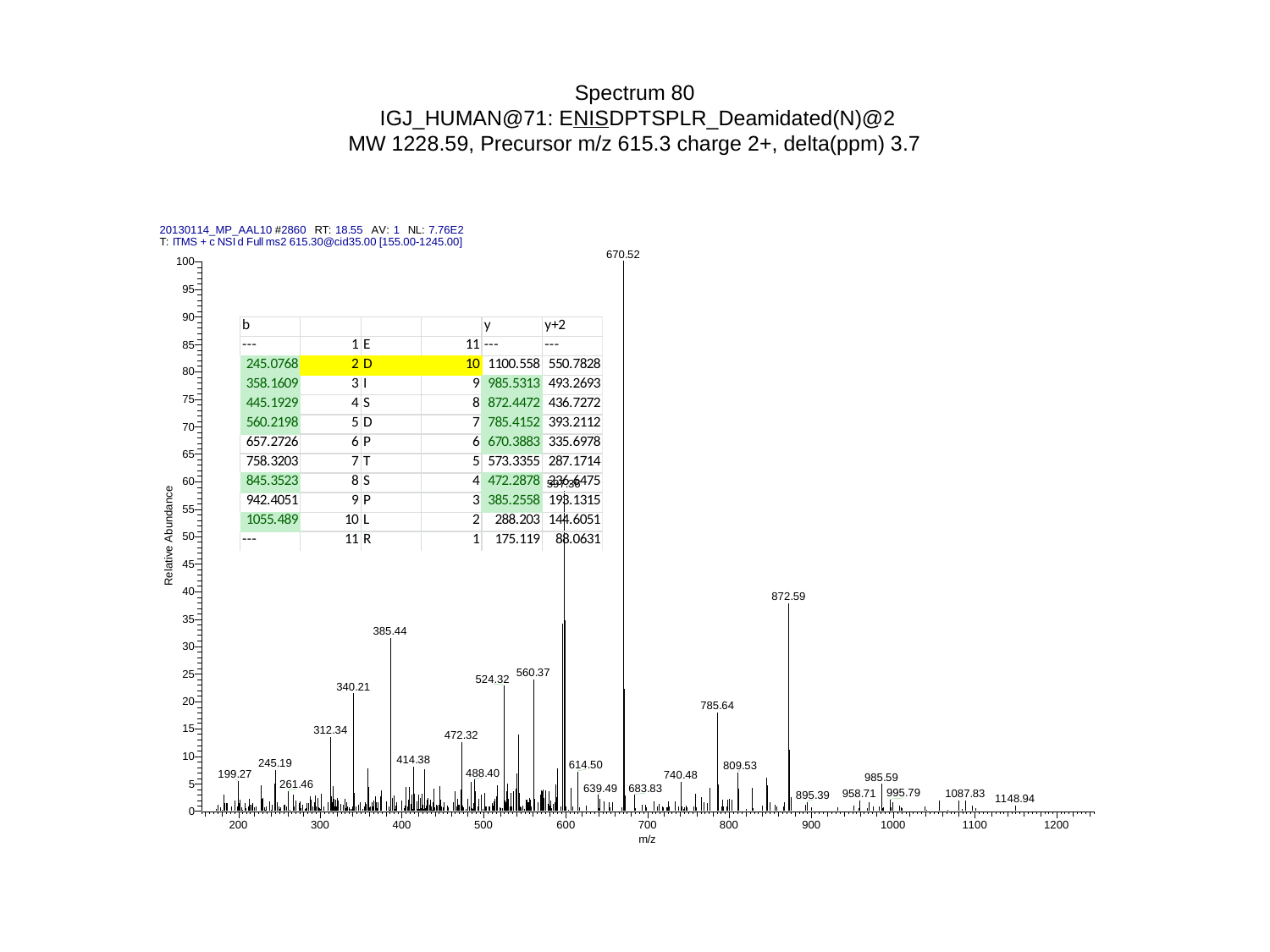

# Spectrum 80 IGJ_HUMAN@71: ENISDPTSPLR_Deamidated(N)@2 MW 1228.59, Precursor m/z 615.3 charge 2+, delta(ppm) 3.7

## Slide 99
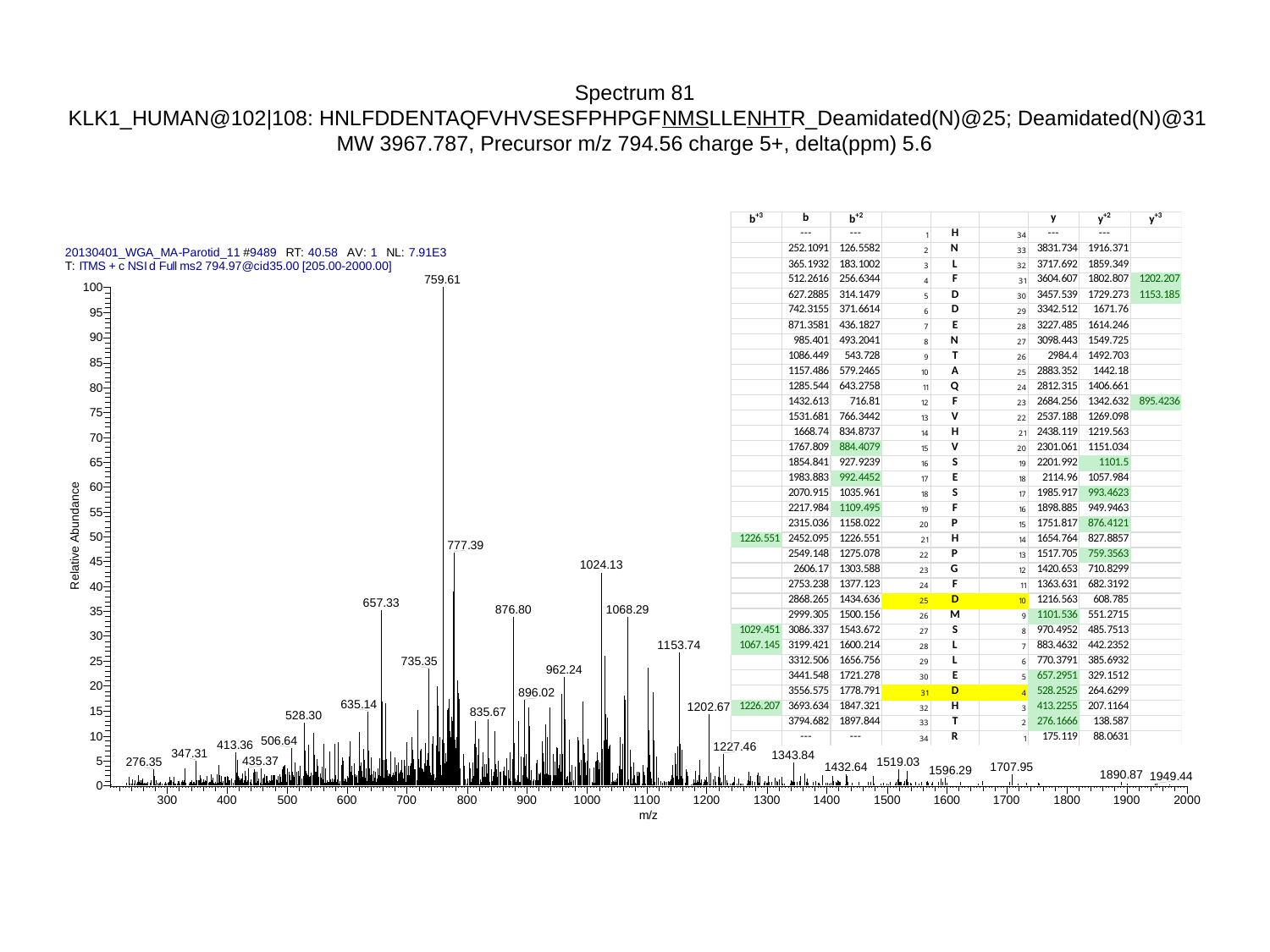

# Spectrum 81 KLK1_HUMAN@102|108: HNLFDDENTAQFVHVSESFPHPGFNMSLLENHTR_Deamidated(N)@25; Deamidated(N)@31 MW 3967.787, Precursor m/z 794.56 charge 5+, delta(ppm) 5.6

## Slide 100
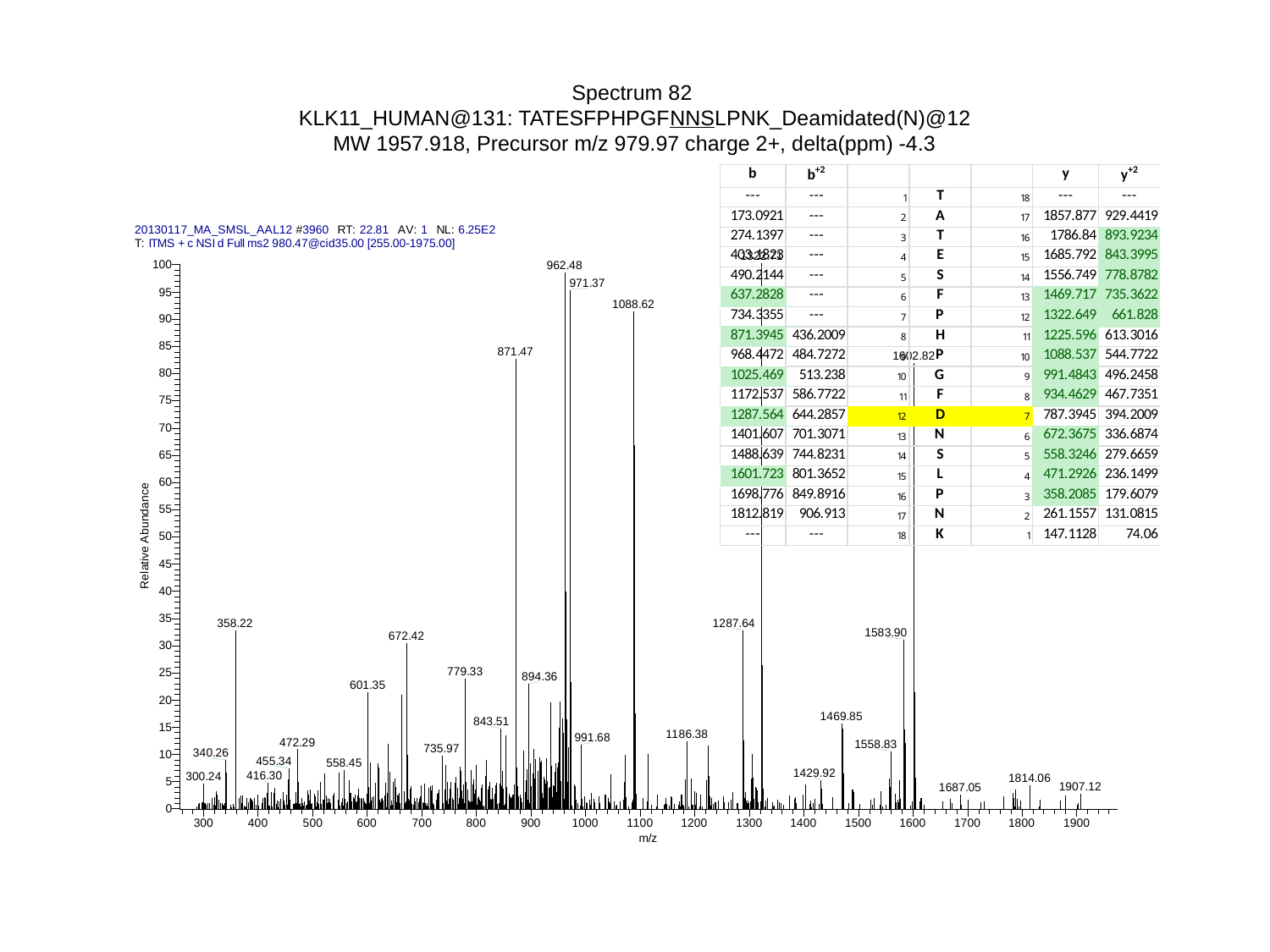

# Spectrum 82 KLK11_HUMAN@131: TATESFPHPGFNNSLPNK_Deamidated(N)@12 MW 1957.918, Precursor m/z 979.97 charge 2+, delta(ppm) -4.3

## Slide 101
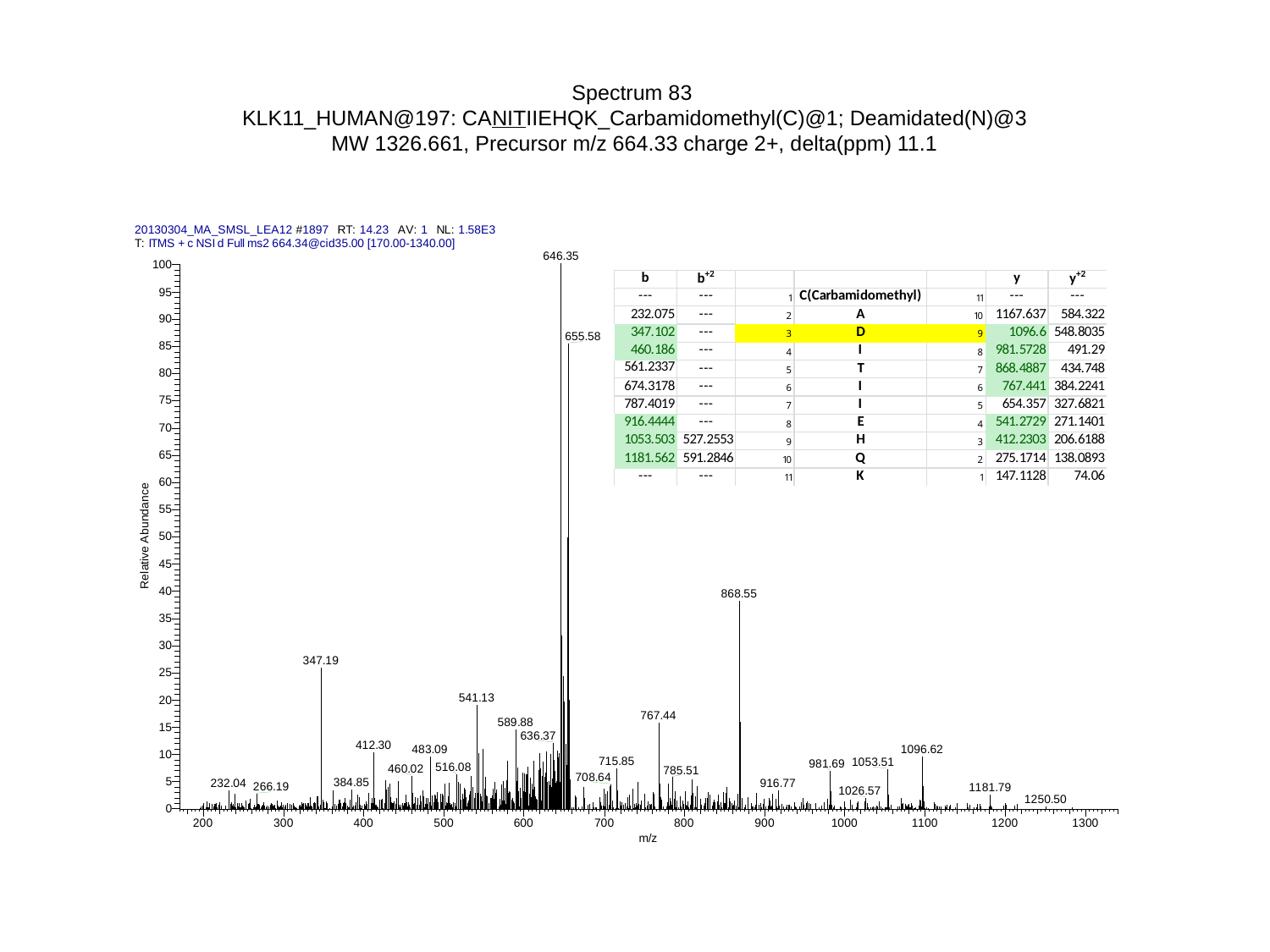

# Spectrum 83 KLK11_HUMAN@197: CANITIIEHQK_Carbamidomethyl(C)@1; Deamidated(N)@3 MW 1326.661, Precursor m/z 664.33 charge 2+, delta(ppm) 11.1

## Slide 102
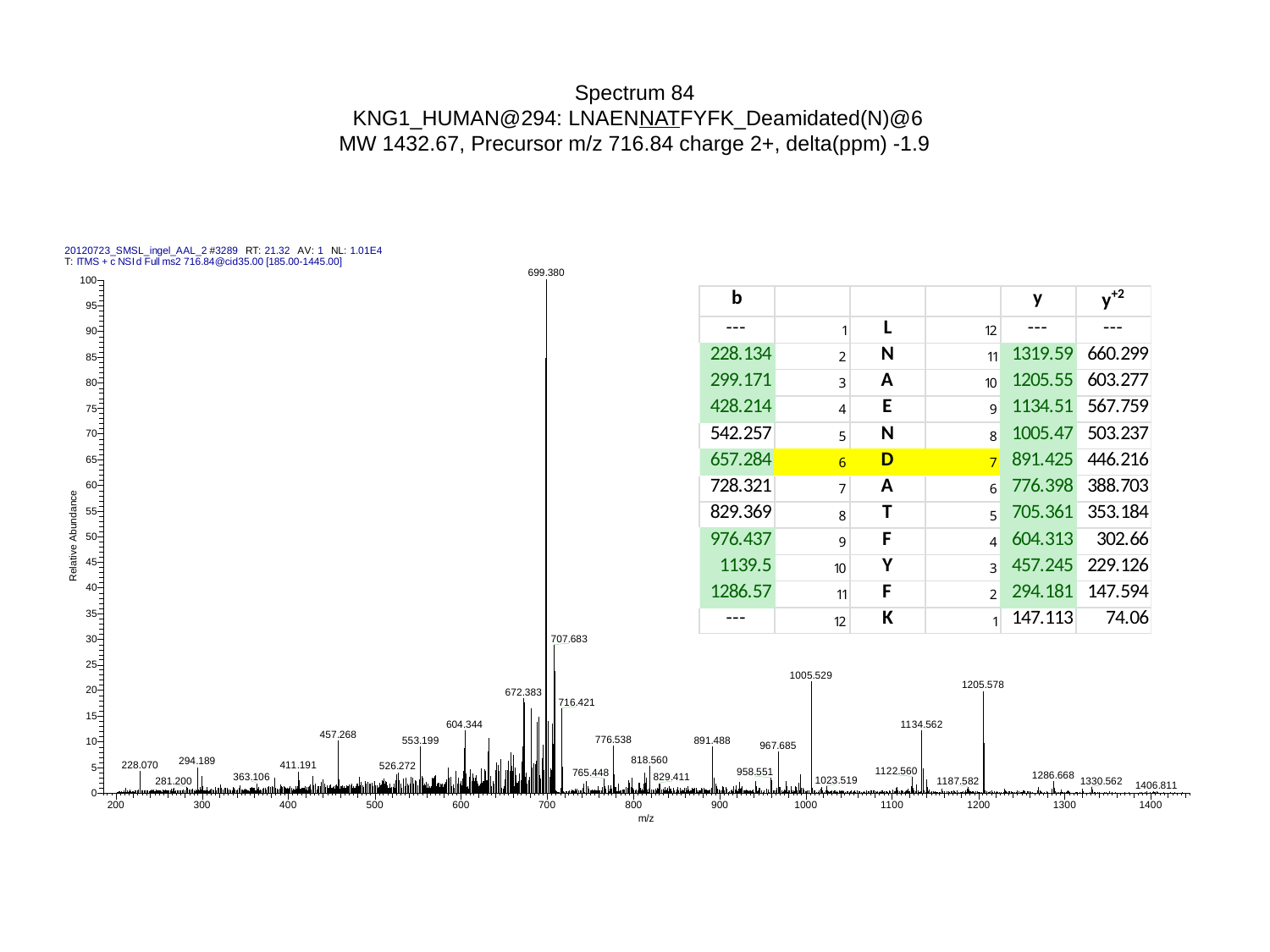

# Spectrum 84 KNG1_HUMAN@294: LNAENNATFYFK_Deamidated(N)@6 MW 1432.67, Precursor m/z 716.84 charge 2+, delta(ppm) -1.9

## Slide 103
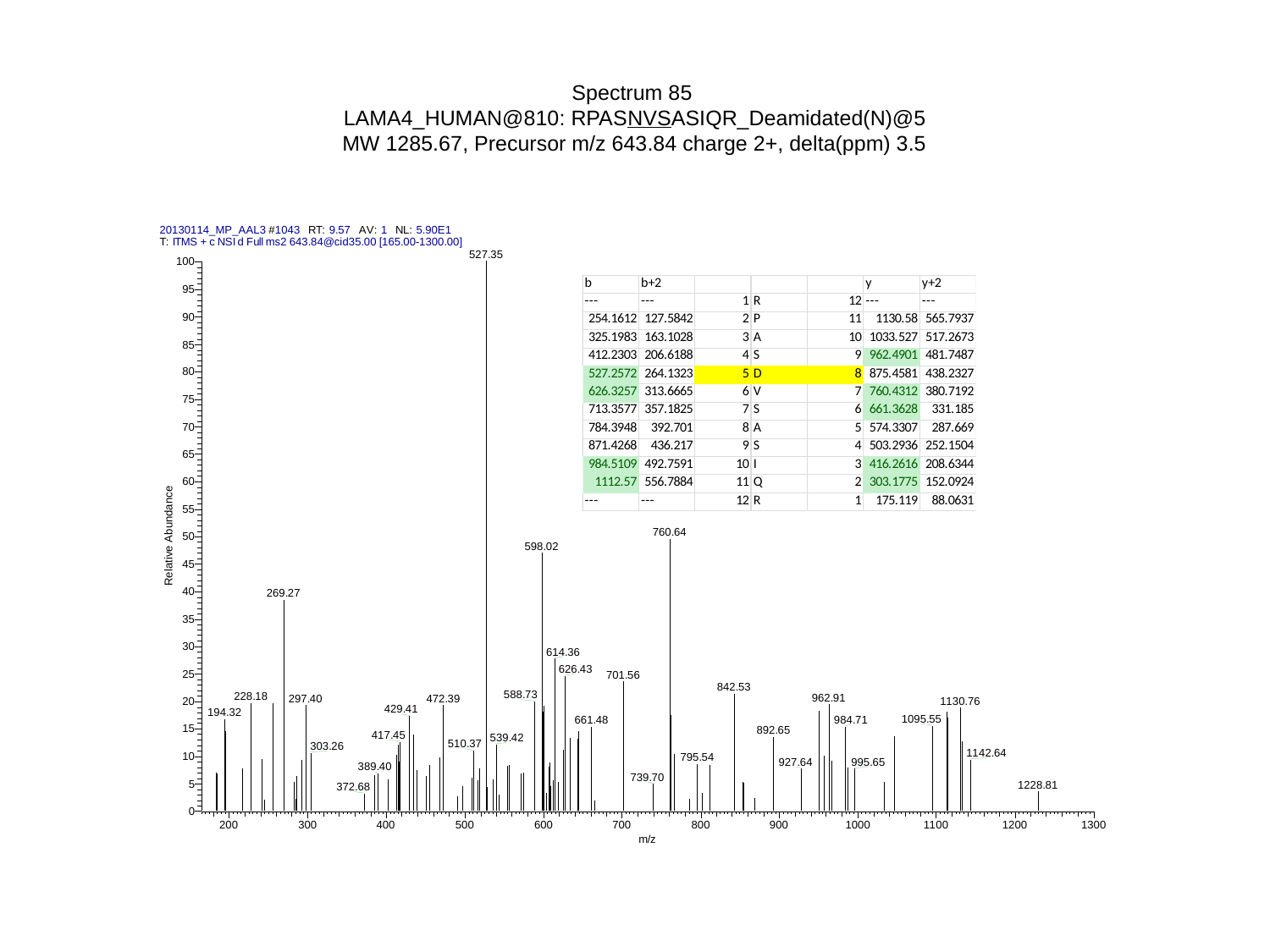

# Spectrum 85 LAMA4_HUMAN@810: RPASNVSASIQR_Deamidated(N)@5 MW 1285.67, Precursor m/z 643.84 charge 2+, delta(ppm) 3.5

## Slide 104
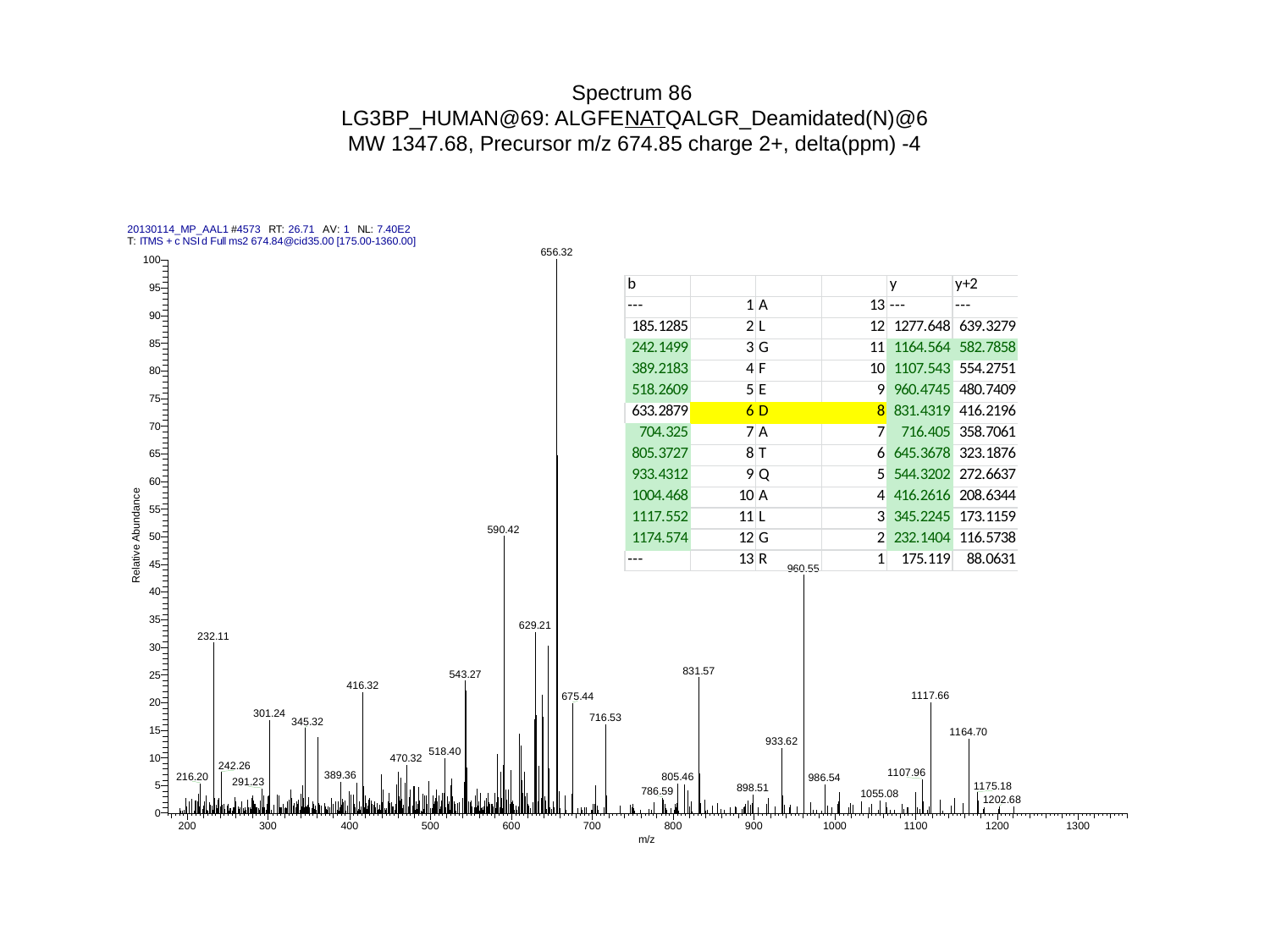

# Spectrum 86 LG3BP_HUMAN@69: ALGFENATQALGR_Deamidated(N)@6 MW 1347.68, Precursor m/z 674.85 charge 2+, delta(ppm) -4

## Slide 105
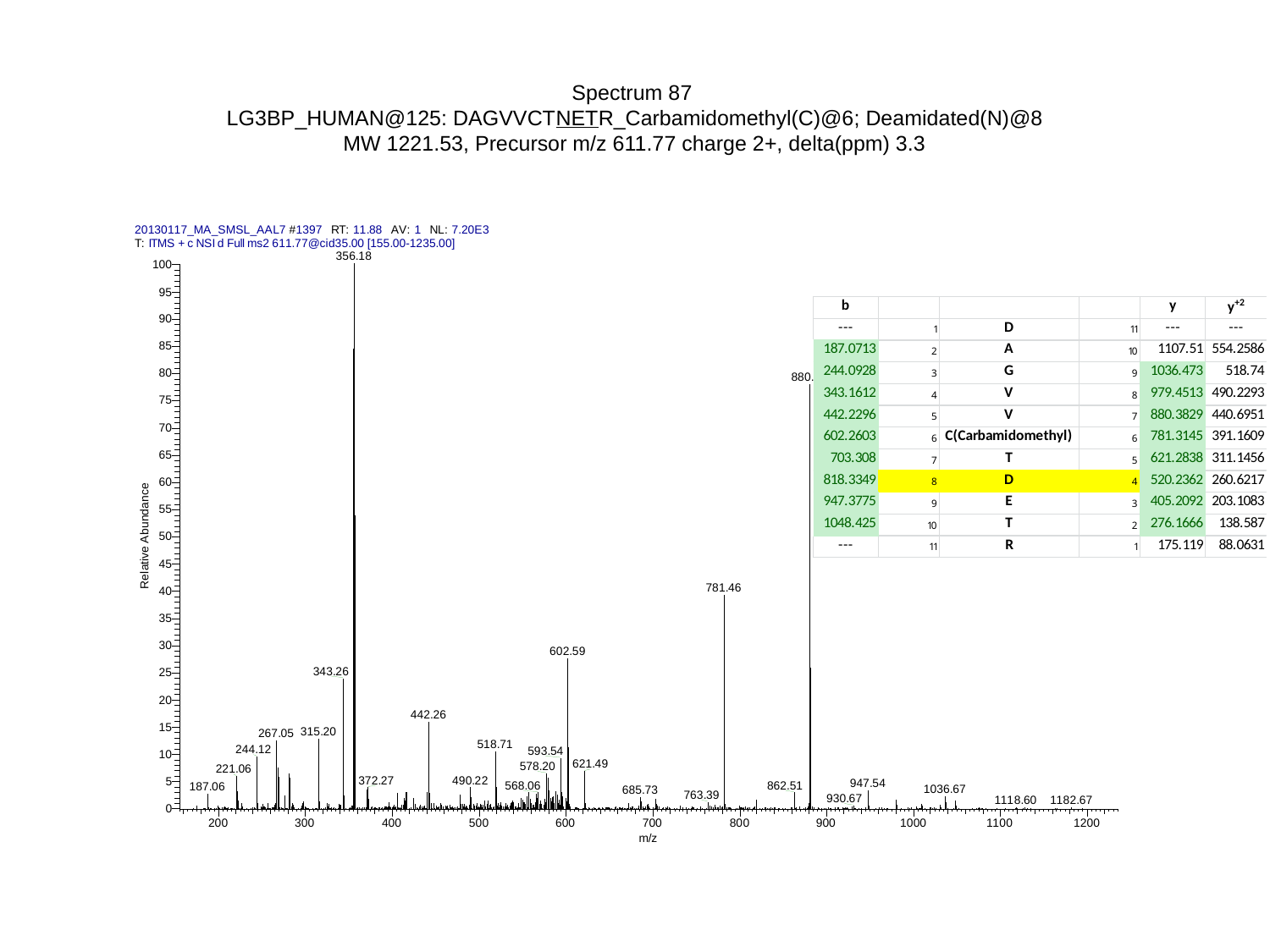

# Spectrum 87 LG3BP_HUMAN@125: DAGVVCTNETR_Carbamidomethyl(C)@6; Deamidated(N)@8 MW 1221.53, Precursor m/z 611.77 charge 2+, delta(ppm) 3.3

## Slide 106
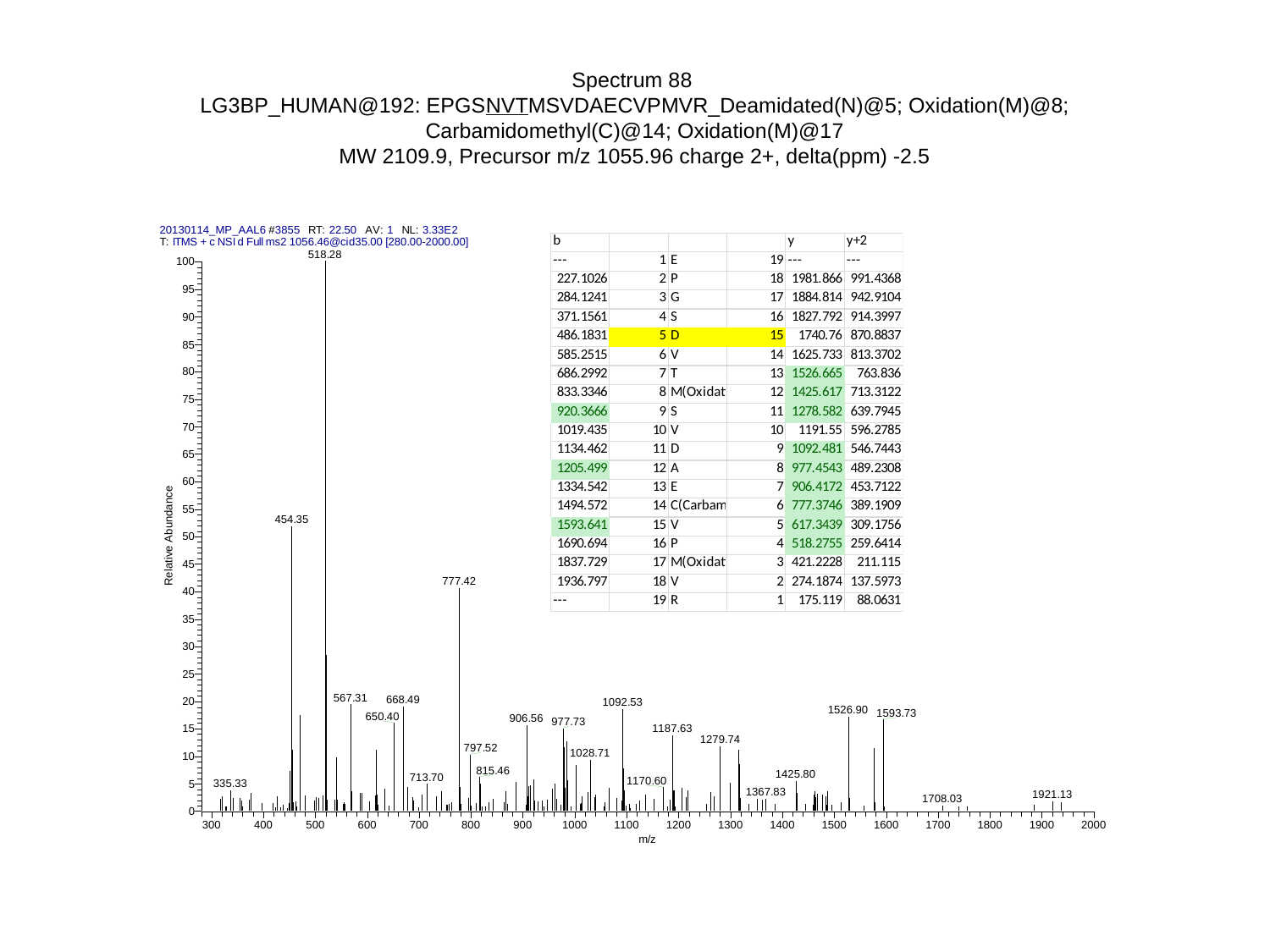

# Spectrum 88 LG3BP_HUMAN@192: EPGSNVTMSVDAECVPMVR_Deamidated(N)@5; Oxidation(M)@8; Carbamidomethyl(C)@14; Oxidation(M)@17 MW 2109.9, Precursor m/z 1055.96 charge 2+, delta(ppm) -2.5

## Slide 107
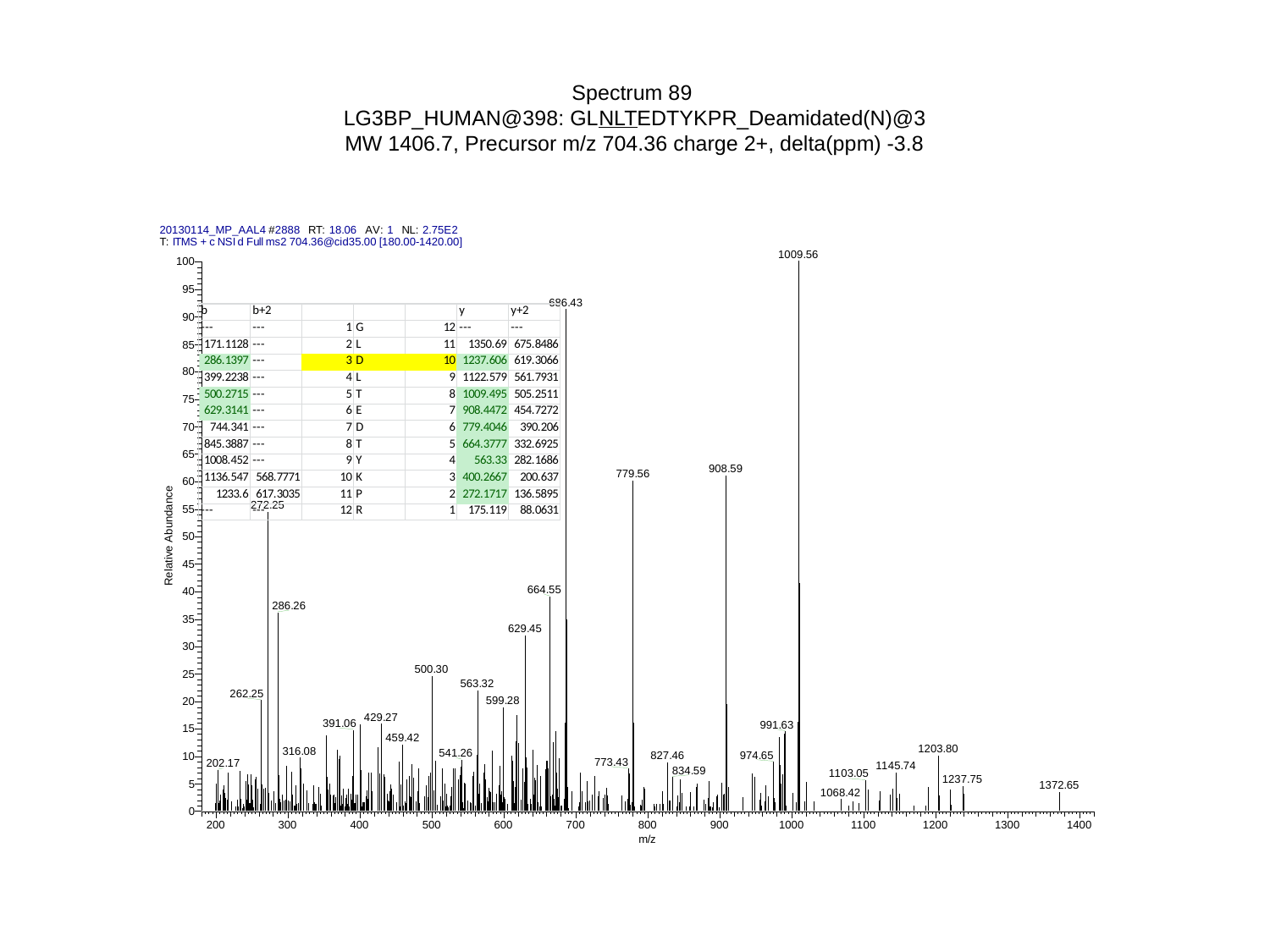

# Spectrum 89 LG3BP_HUMAN@398: GLNLTEDTYKPR_Deamidated(N)@3 MW 1406.7, Precursor m/z 704.36 charge 2+, delta(ppm) -3.8

## Slide 108
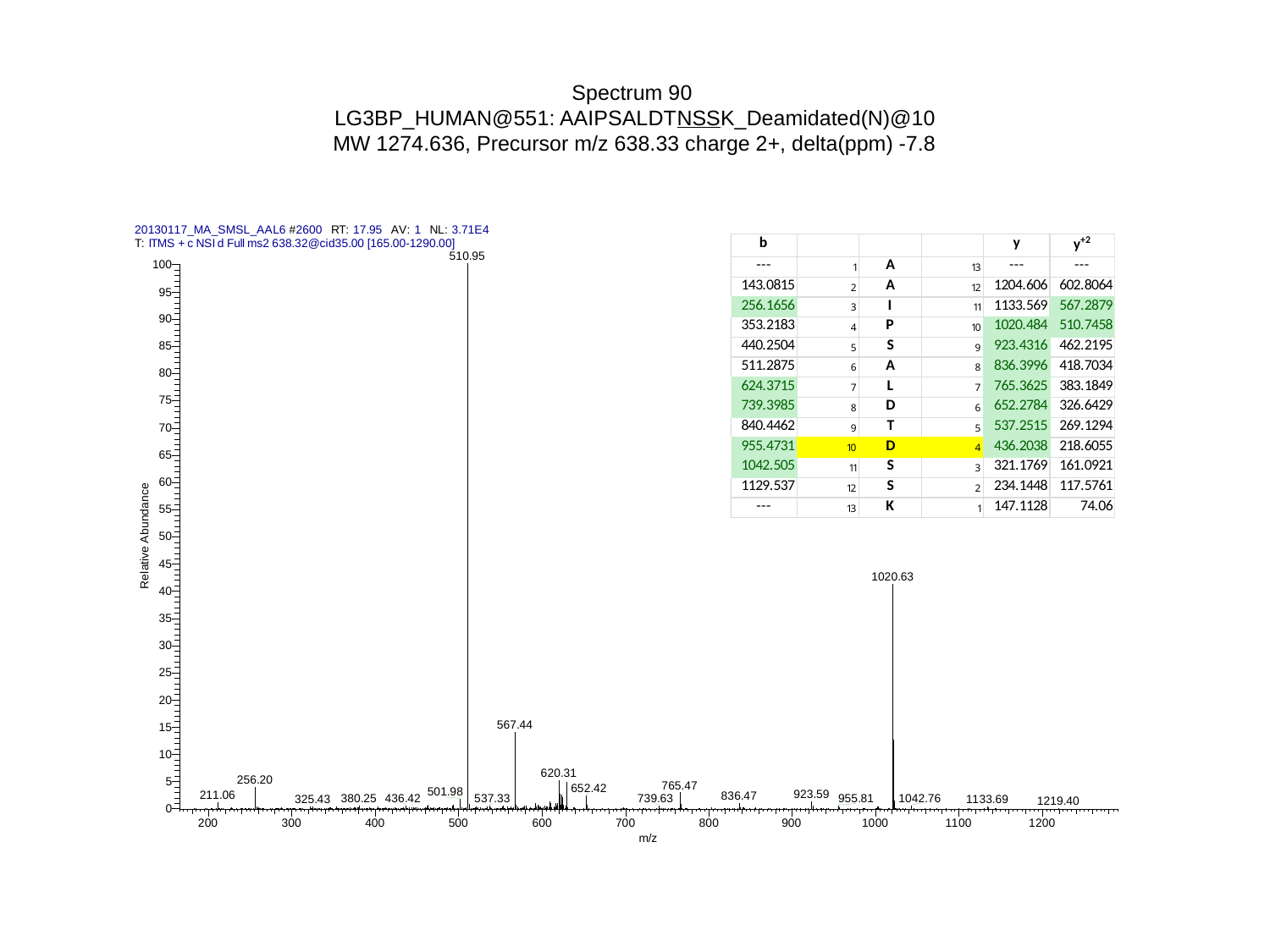

# Spectrum 90 LG3BP_HUMAN@551: AAIPSALDTNSSK_Deamidated(N)@10 MW 1274.636, Precursor m/z 638.33 charge 2+, delta(ppm) -7.8

## Slide 109
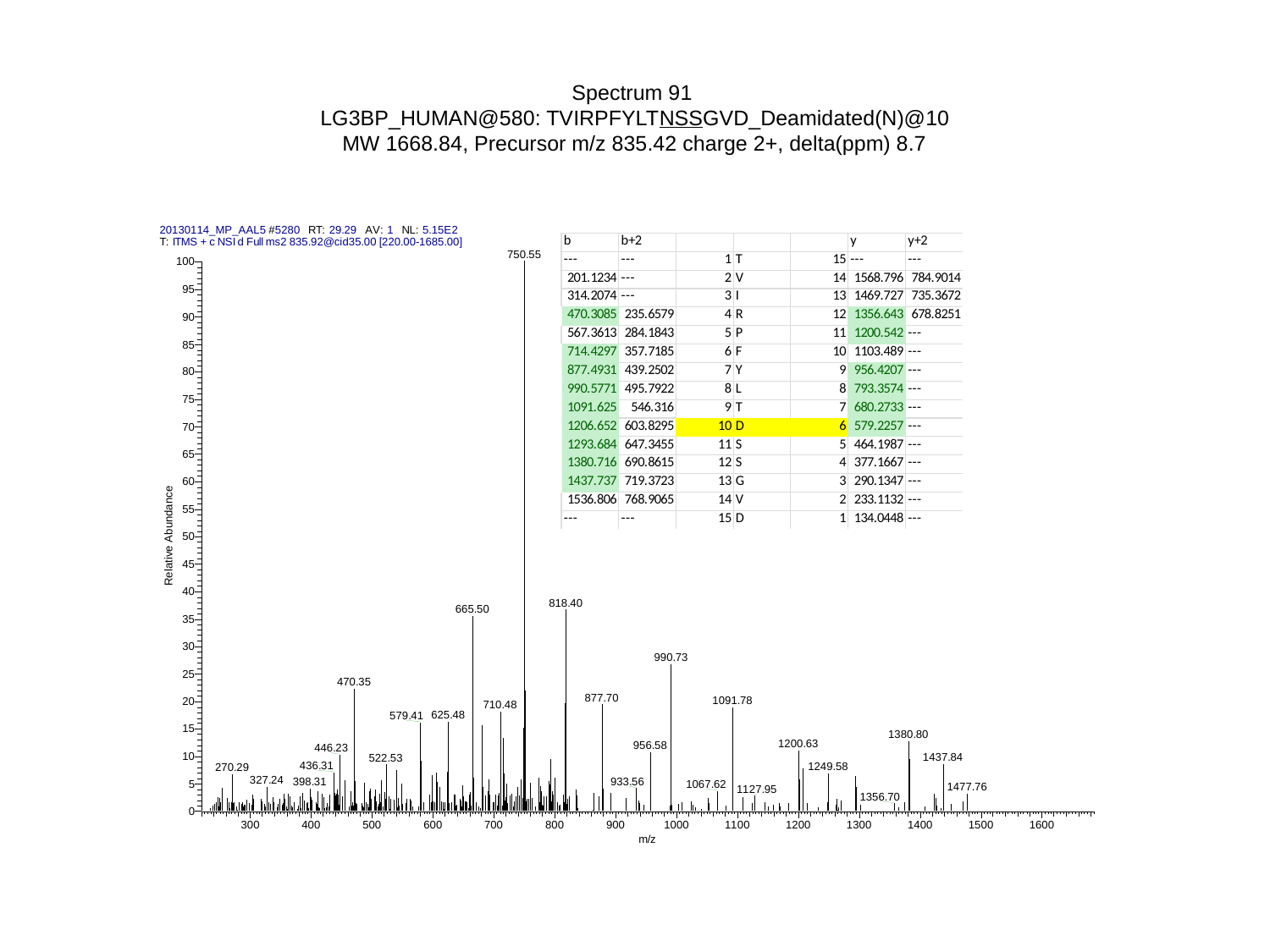

# Spectrum 91 LG3BP_HUMAN@580: TVIRPFYLTNSSGVD_Deamidated(N)@10 MW 1668.84, Precursor m/z 835.42 charge 2+, delta(ppm) 8.7

## Slide 110
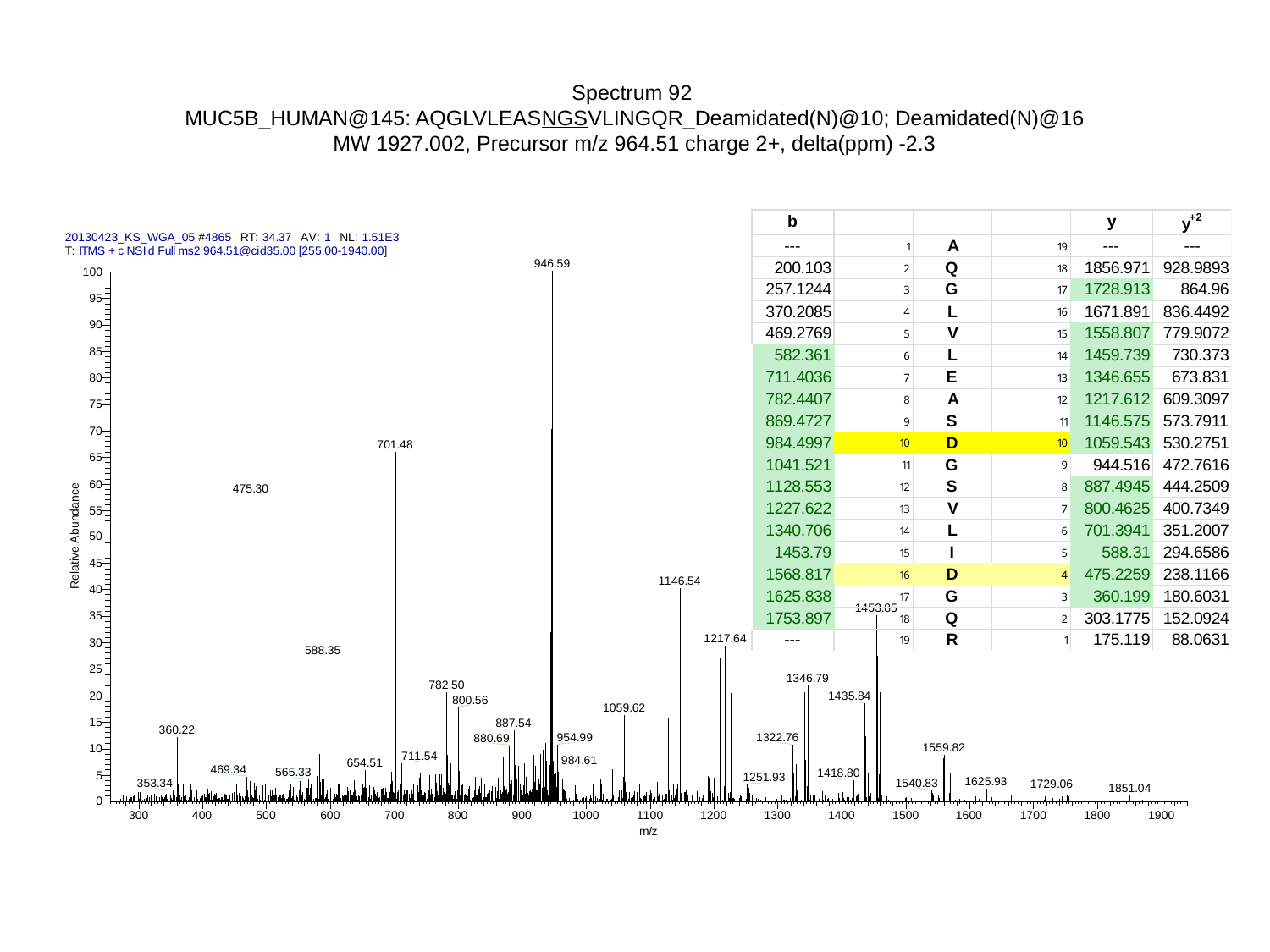

# Spectrum 92 MUC5B_HUMAN@145: AQGLVLEASNGSVLINGQR_Deamidated(N)@10; Deamidated(N)@16 MW 1927.002, Precursor m/z 964.51 charge 2+, delta(ppm) -2.3

## Slide 111
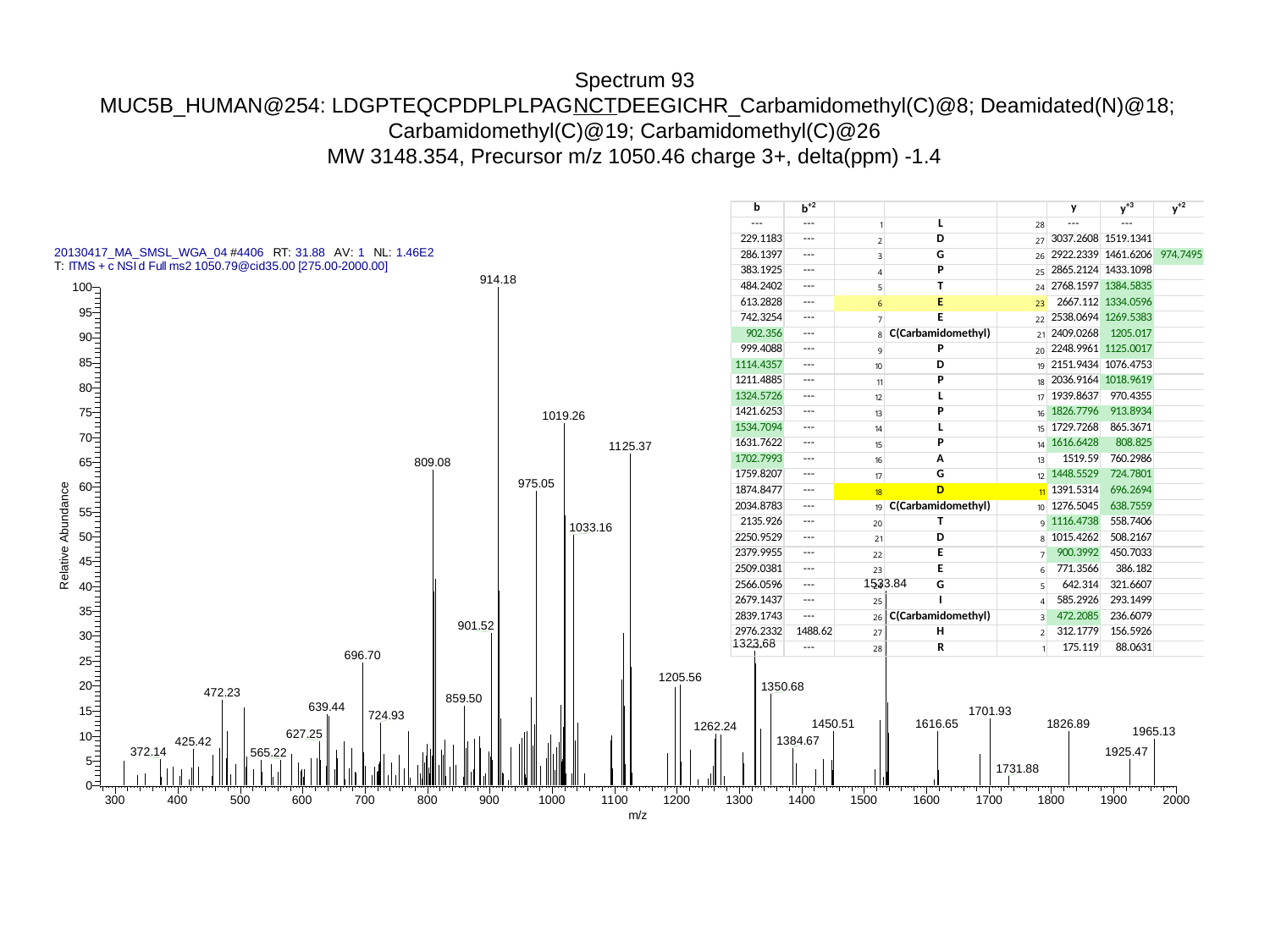

# Spectrum 93 MUC5B_HUMAN@254: LDGPTEQCPDPLPLPAGNCTDEEGICHR_Carbamidomethyl(C)@8; Deamidated(N)@18; Carbamidomethyl(C)@19; Carbamidomethyl(C)@26 MW 3148.354, Precursor m/z 1050.46 charge 3+, delta(ppm) -1.4

## Slide 112
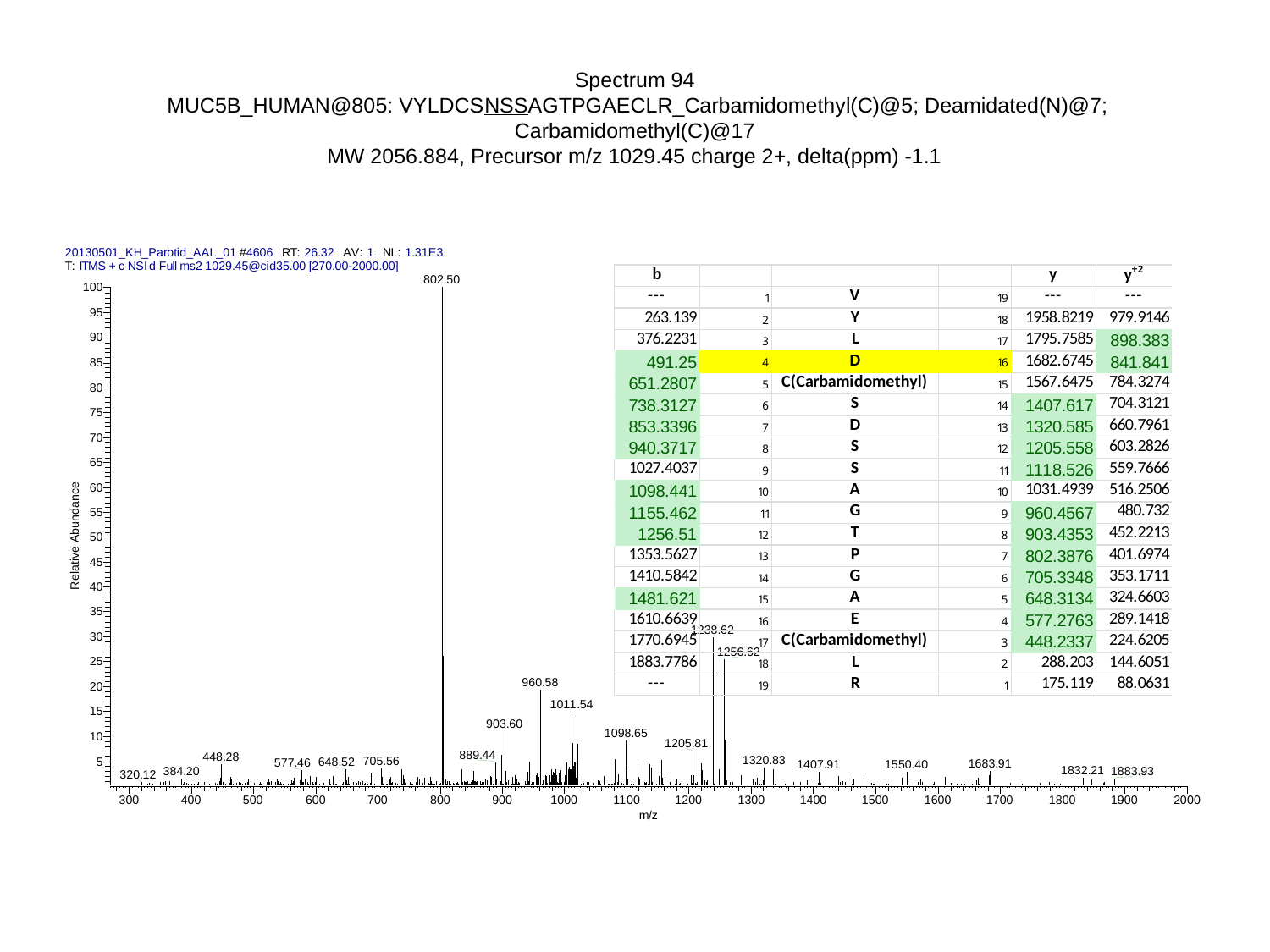

# Spectrum 94 MUC5B_HUMAN@805: VYLDCSNSSAGTPGAECLR_Carbamidomethyl(C)@5; Deamidated(N)@7; Carbamidomethyl(C)@17 MW 2056.884, Precursor m/z 1029.45 charge 2+, delta(ppm) -1.1

## Slide 113
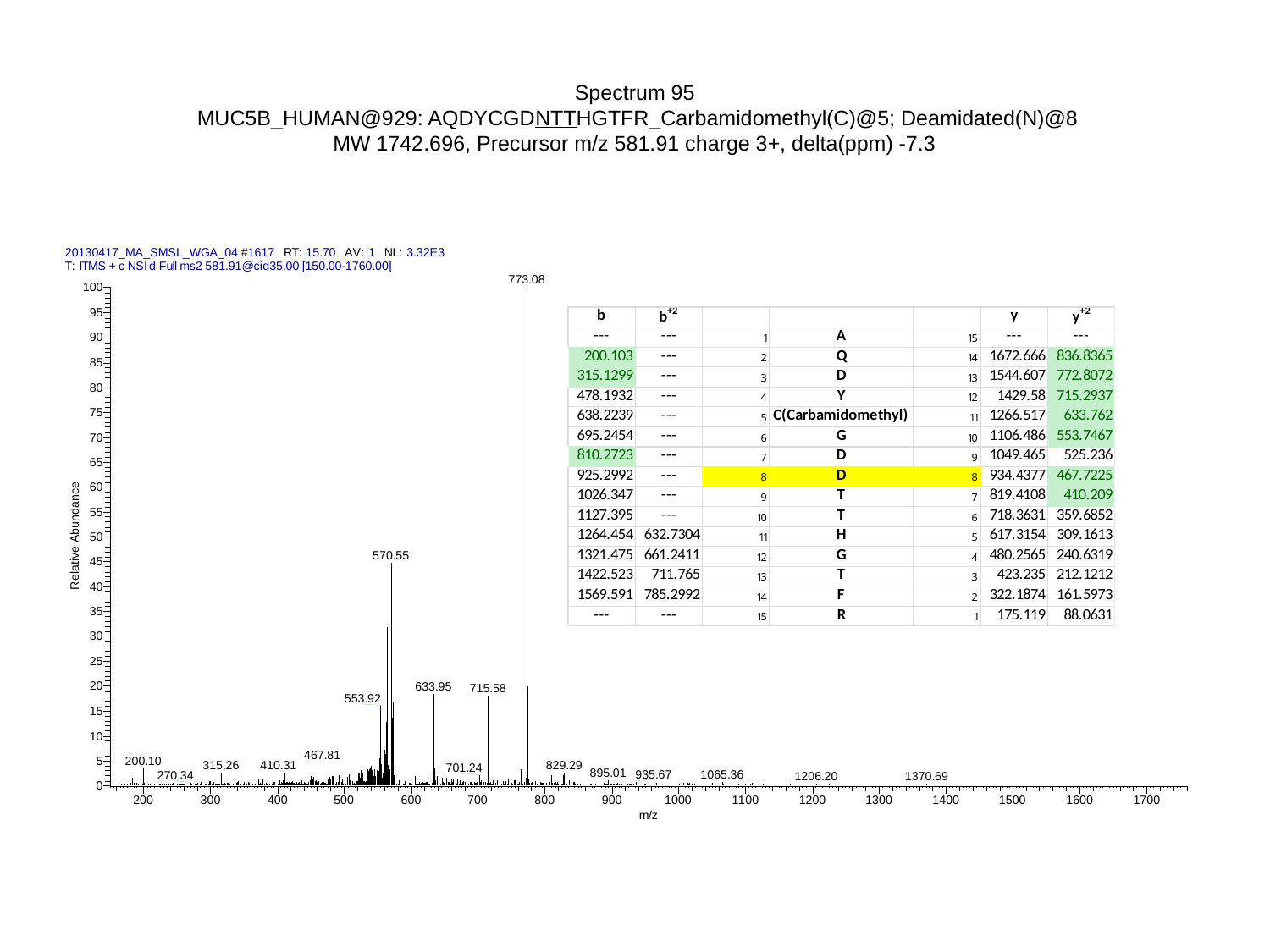

# Spectrum 95 MUC5B_HUMAN@929: AQDYCGDNTTHGTFR_Carbamidomethyl(C)@5; Deamidated(N)@8 MW 1742.696, Precursor m/z 581.91 charge 3+, delta(ppm) -7.3

## Slide 114
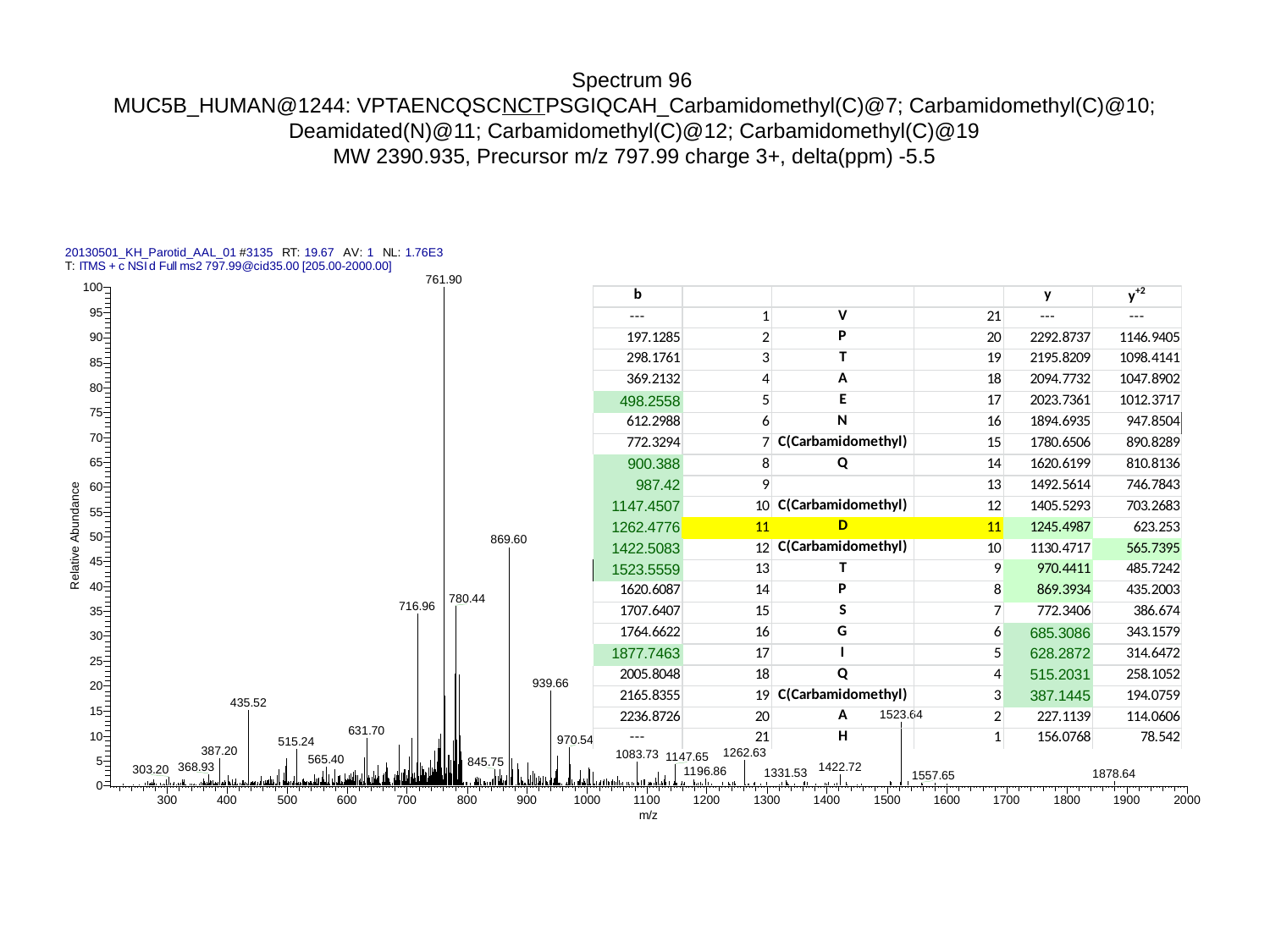

# Spectrum 96 MUC5B_HUMAN@1244: VPTAENCQSCNCTPSGIQCAH_Carbamidomethyl(C)@7; Carbamidomethyl(C)@10; Deamidated(N)@11; Carbamidomethyl(C)@12; Carbamidomethyl(C)@19 MW 2390.935, Precursor m/z 797.99 charge 3+, delta(ppm) -5.5

## Slide 115
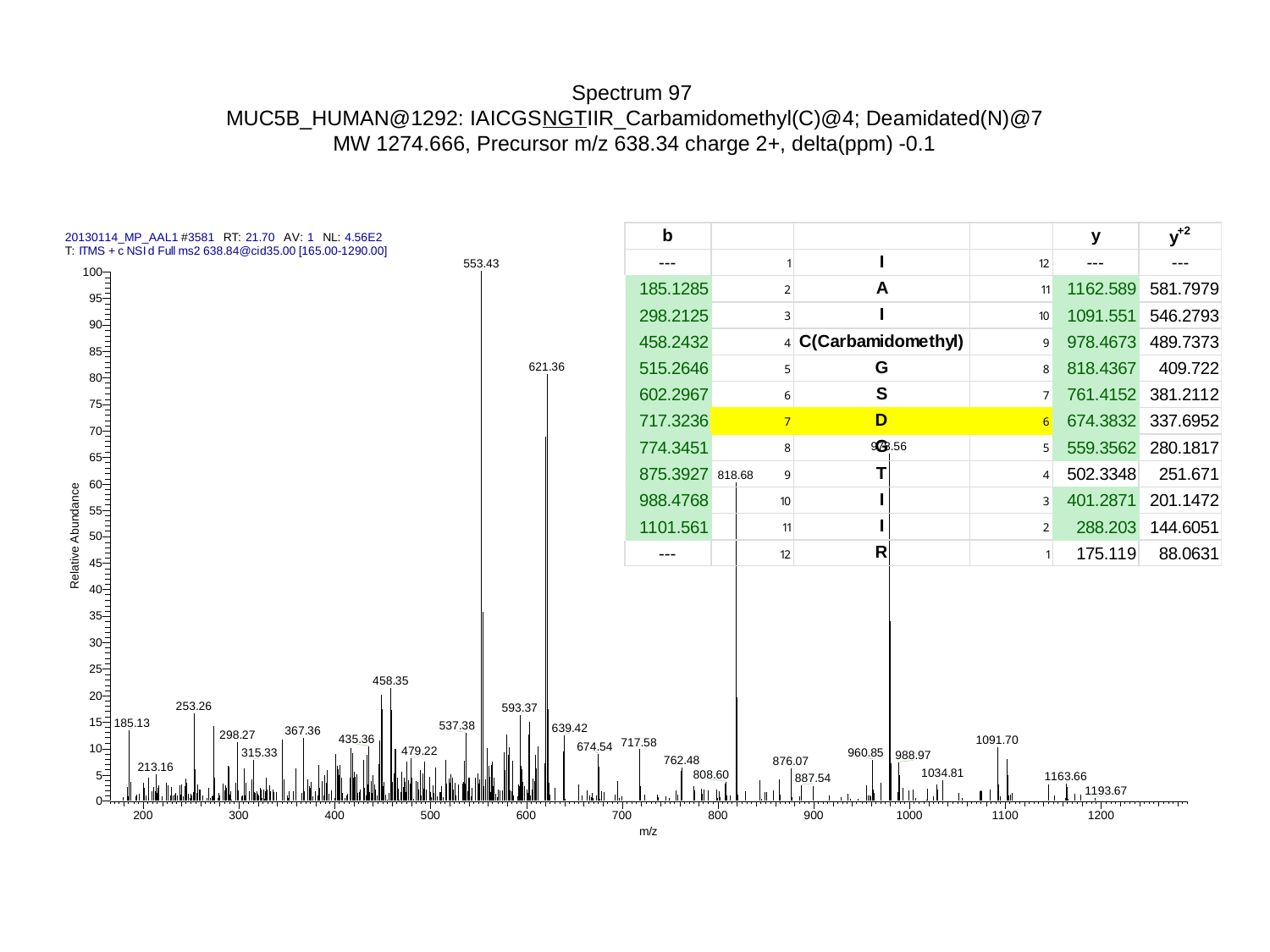

# Spectrum 97 MUC5B_HUMAN@1292: IAICGSNGTIIR_Carbamidomethyl(C)@4; Deamidated(N)@7 MW 1274.666, Precursor m/z 638.34 charge 2+, delta(ppm) -0.1

## Slide 116
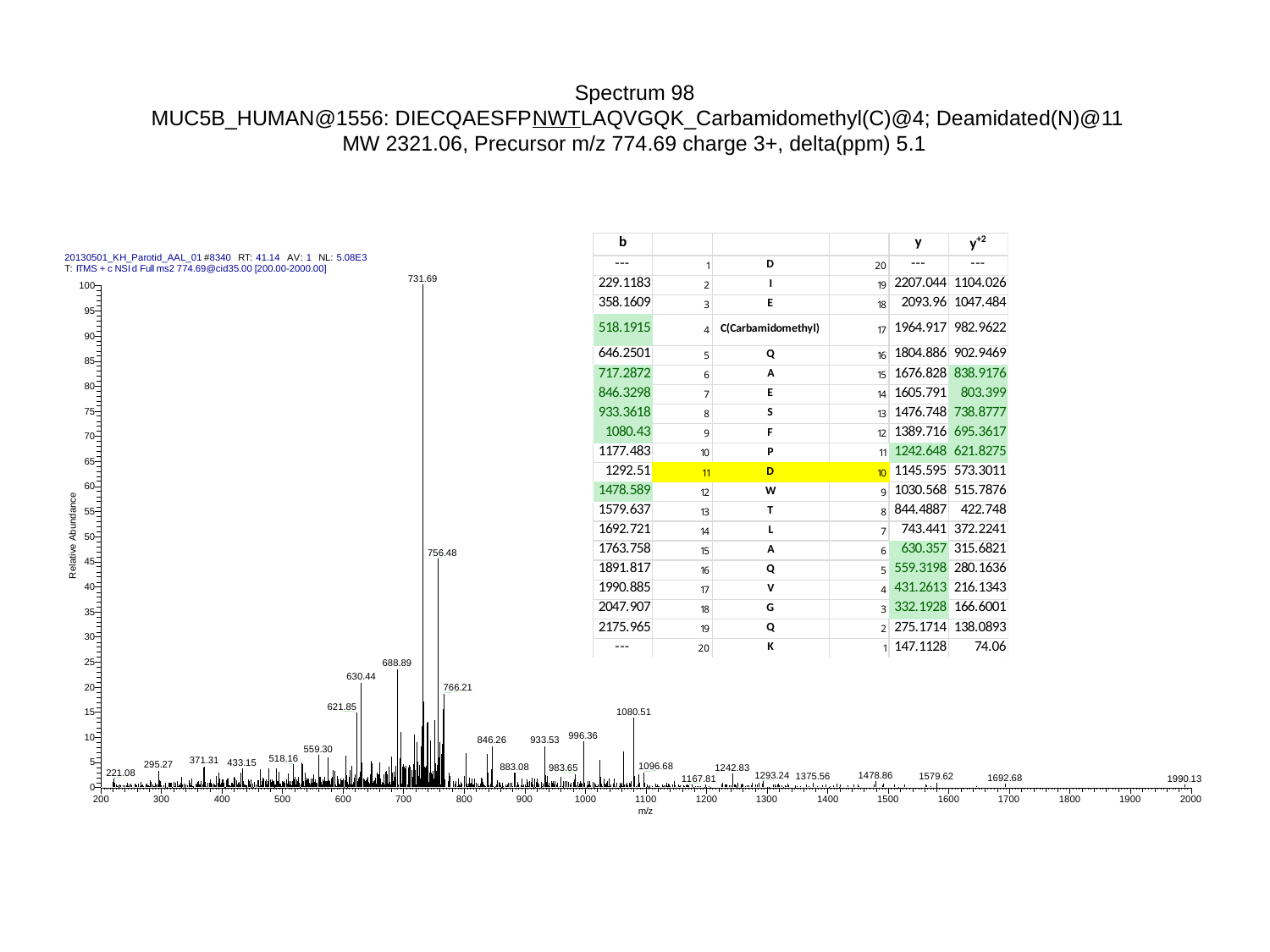

# Spectrum 98 MUC5B_HUMAN@1556: DIECQAESFPNWTLAQVGQK_Carbamidomethyl(C)@4; Deamidated(N)@11 MW 2321.06, Precursor m/z 774.69 charge 3+, delta(ppm) 5.1

## Slide 117
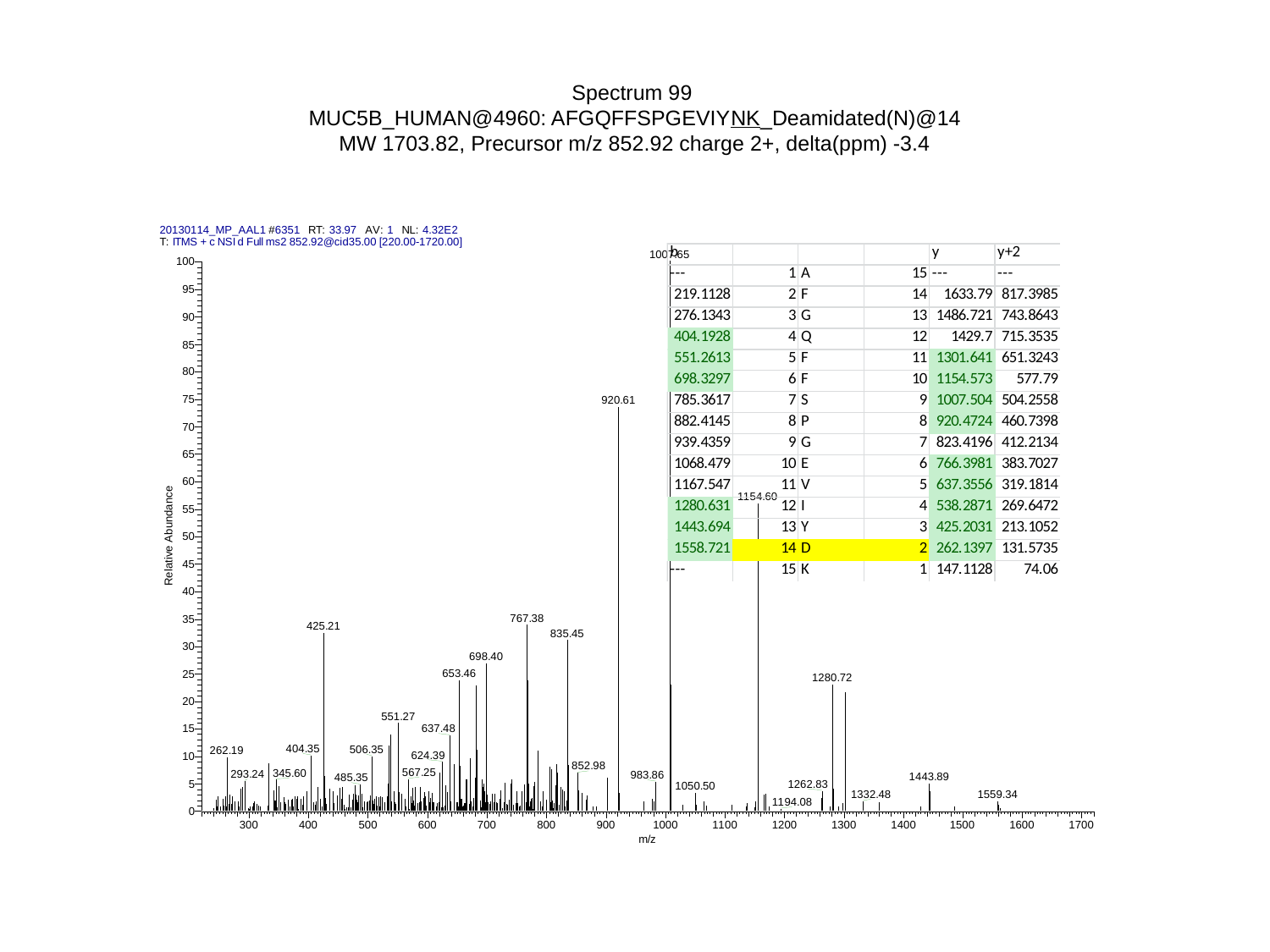

# Spectrum 99 MUC5B_HUMAN@4960: AFGQFFSPGEVIYNK_Deamidated(N)@14 MW 1703.82, Precursor m/z 852.92 charge 2+, delta(ppm) -3.4

## Slide 118
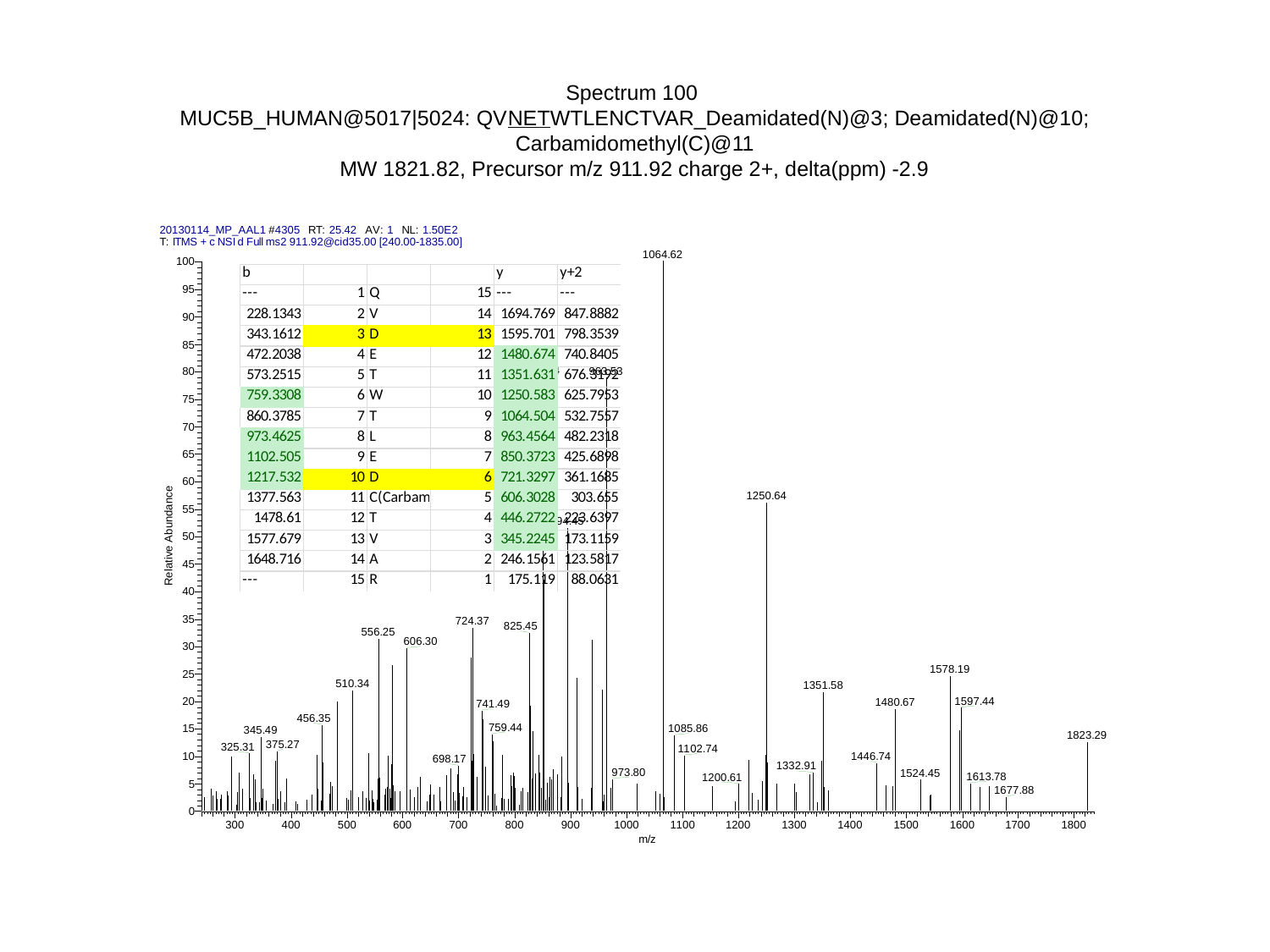

# Spectrum 100 MUC5B_HUMAN@5017|5024: QVNETWTLENCTVAR_Deamidated(N)@3; Deamidated(N)@10; Carbamidomethyl(C)@11 MW 1821.82, Precursor m/z 911.92 charge 2+, delta(ppm) -2.9

## Slide 119
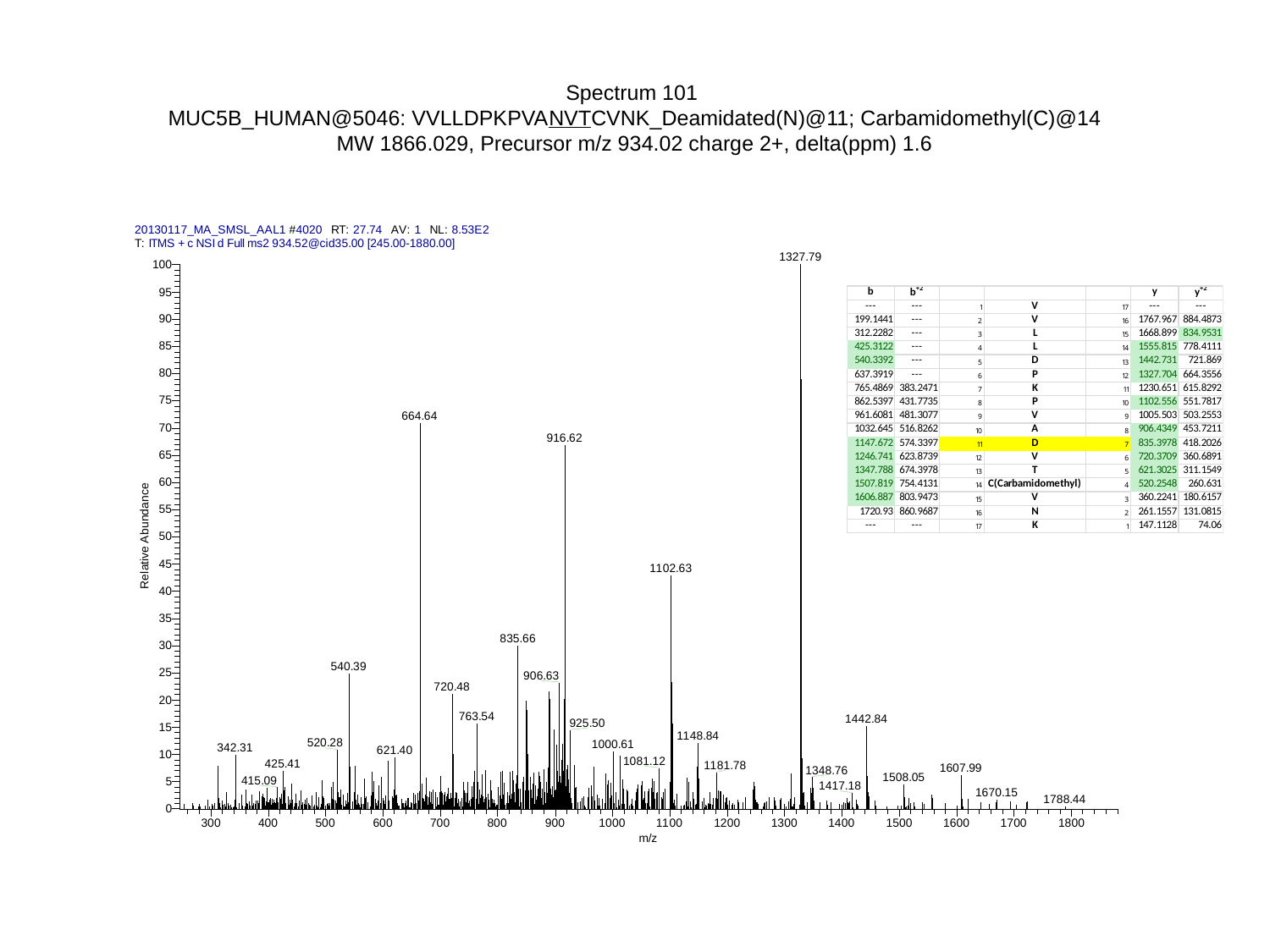

# Spectrum 101 MUC5B_HUMAN@5046: VVLLDPKPVANVTCVNK_Deamidated(N)@11; Carbamidomethyl(C)@14 MW 1866.029, Precursor m/z 934.02 charge 2+, delta(ppm) 1.6

## Slide 120
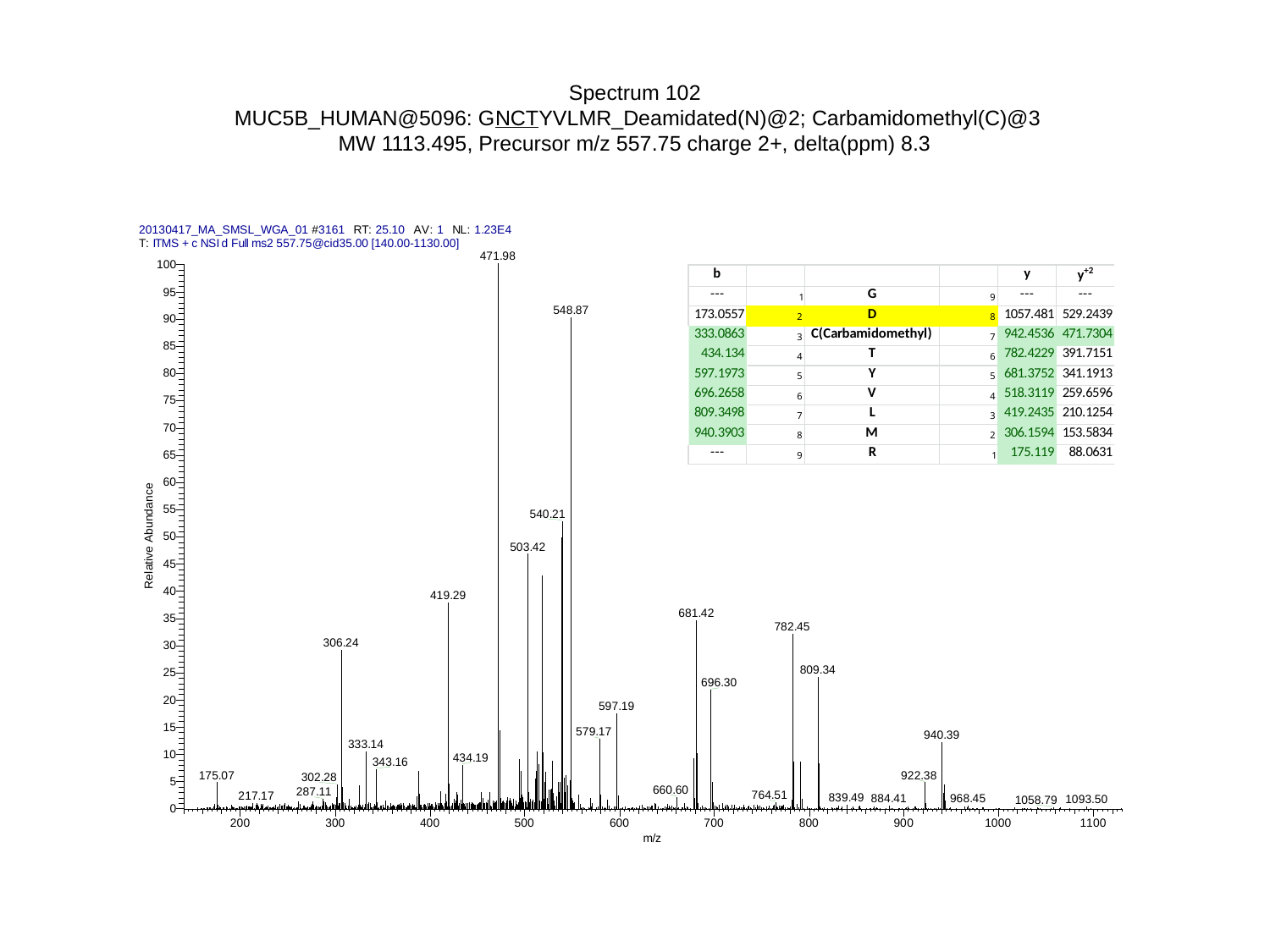

# Spectrum 102 MUC5B_HUMAN@5096: GNCTYVLMR_Deamidated(N)@2; Carbamidomethyl(C)@3 MW 1113.495, Precursor m/z 557.75 charge 2+, delta(ppm) 8.3

## Slide 121
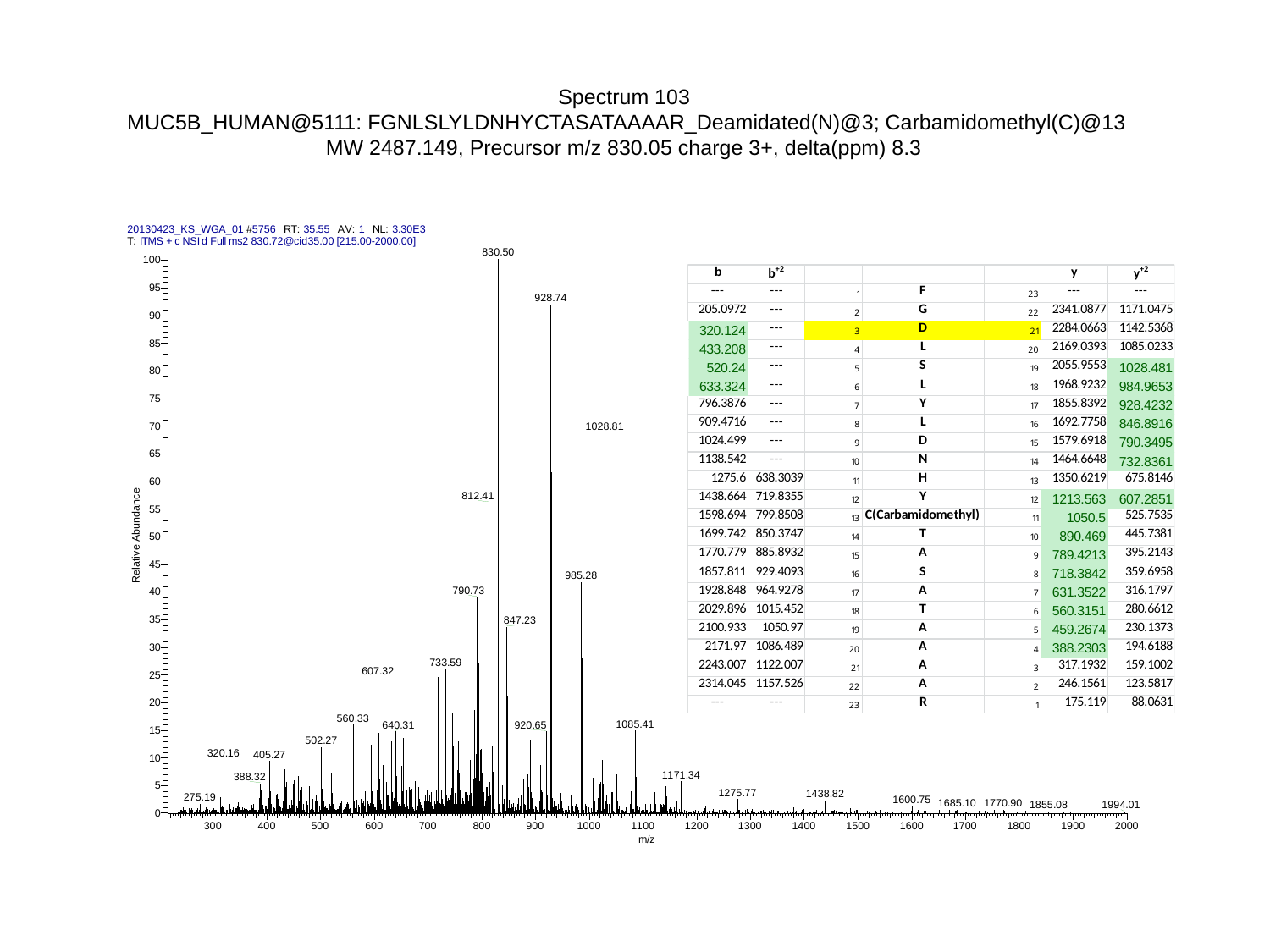

# Spectrum 103 MUC5B_HUMAN@5111: FGNLSLYLDNHYCTASATAAAAR_Deamidated(N)@3; Carbamidomethyl(C)@13 MW 2487.149, Precursor m/z 830.05 charge 3+, delta(ppm) 8.3

## Slide 122
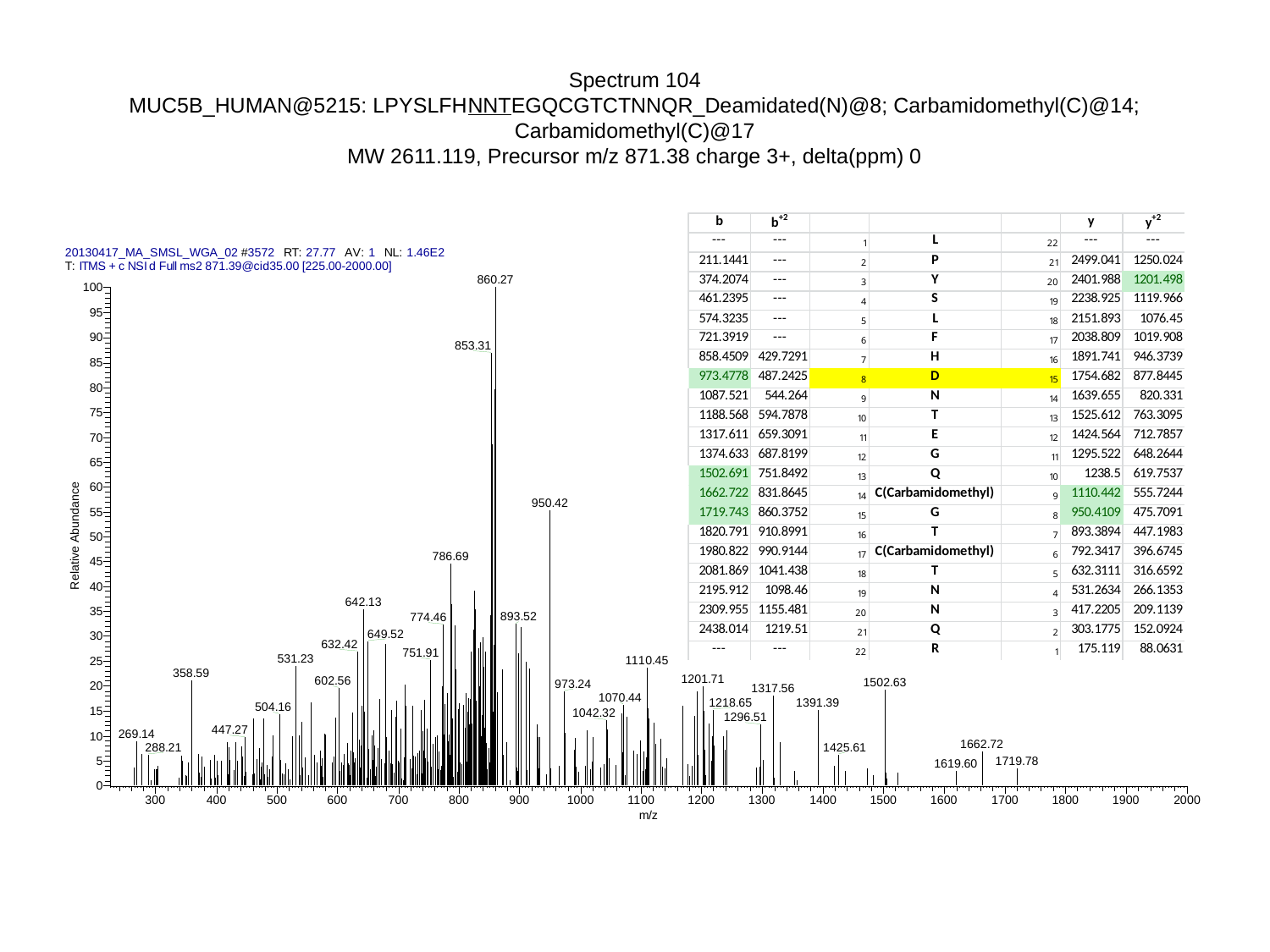

# Spectrum 104 MUC5B_HUMAN@5215: LPYSLFHNNTEGQCGTCTNNQR_Deamidated(N)@8; Carbamidomethyl(C)@14; Carbamidomethyl(C)@17 MW 2611.119, Precursor m/z 871.38 charge 3+, delta(ppm) 0

## Slide 123
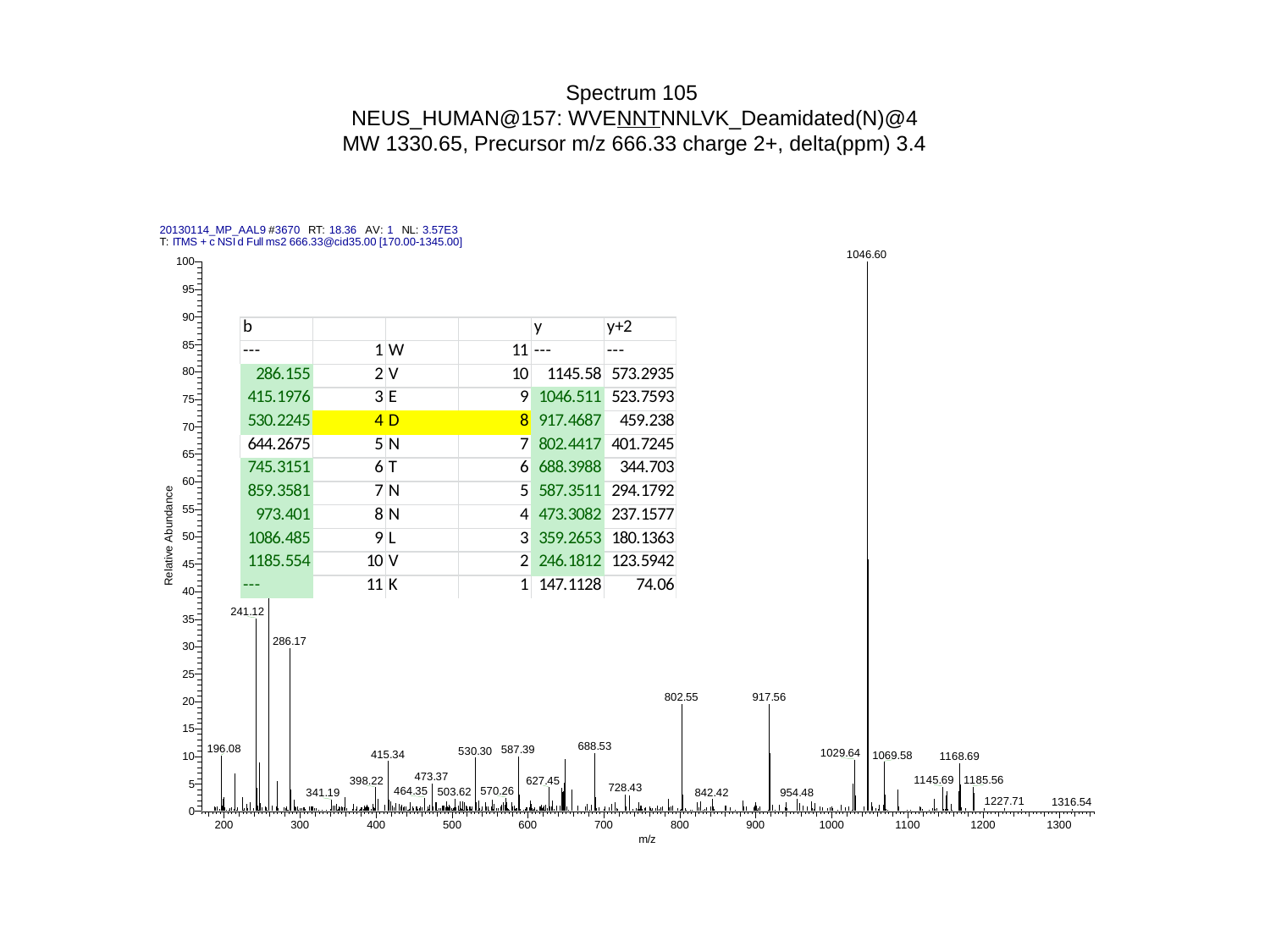

# Spectrum 105 NEUS_HUMAN@157: WVENNTNNLVK_Deamidated(N)@4 MW 1330.65, Precursor m/z 666.33 charge 2+, delta(ppm) 3.4

## Slide 124
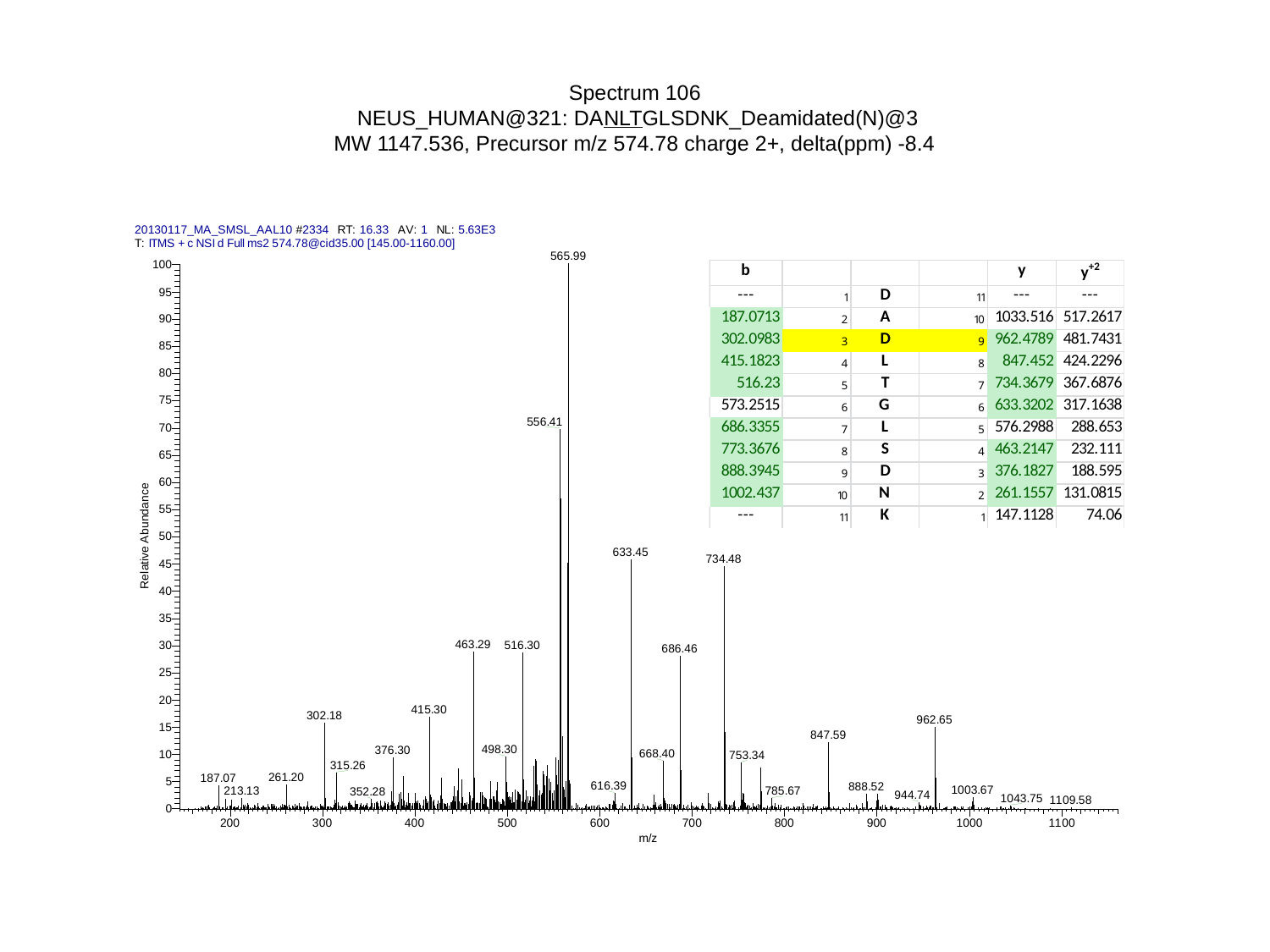

# Spectrum 106 NEUS_HUMAN@321: DANLTGLSDNK_Deamidated(N)@3 MW 1147.536, Precursor m/z 574.78 charge 2+, delta(ppm) -8.4

## Slide 125
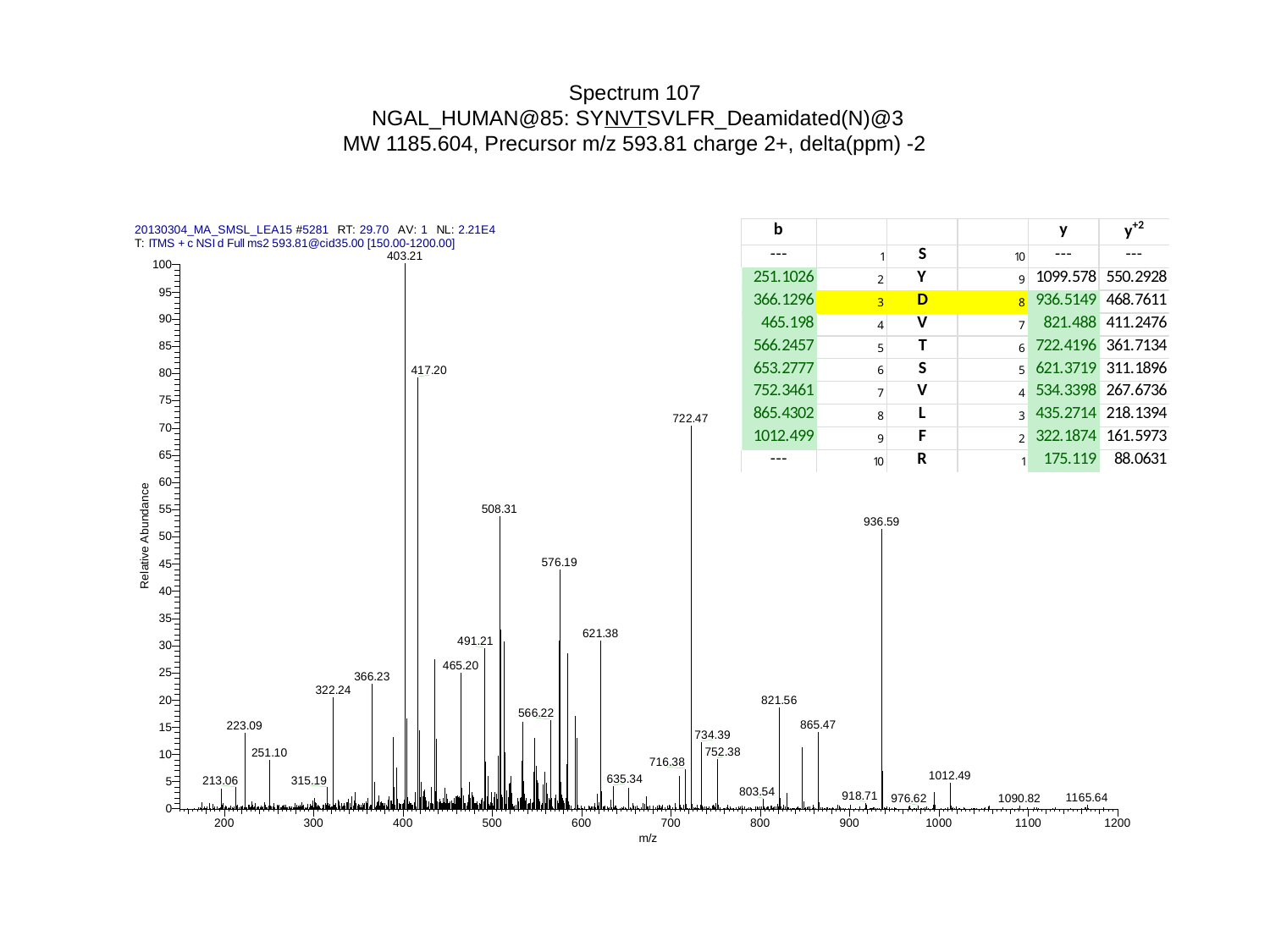

# Spectrum 107 NGAL_HUMAN@85: SYNVTSVLFR_Deamidated(N)@3 MW 1185.604, Precursor m/z 593.81 charge 2+, delta(ppm) -2

## Slide 126
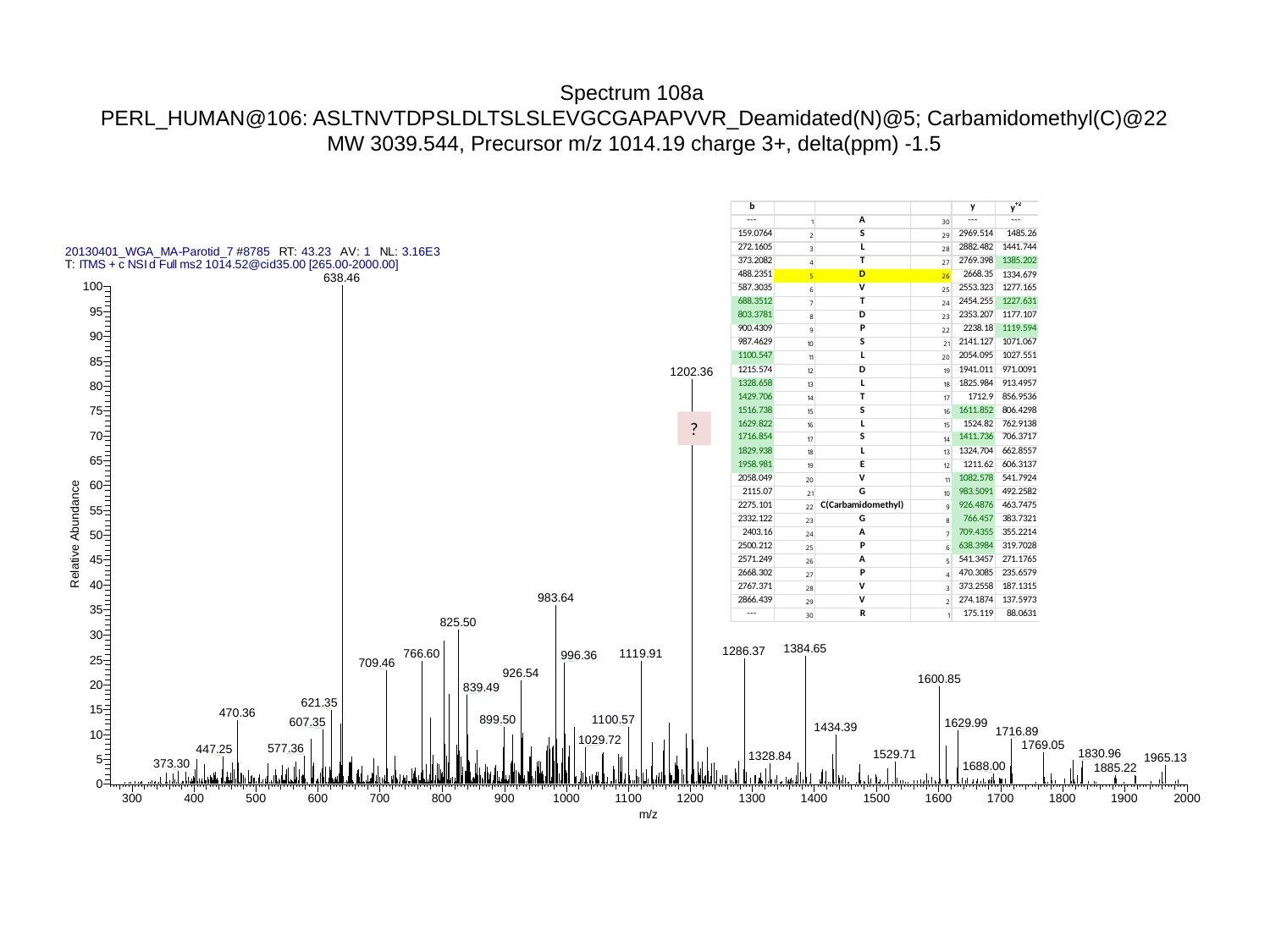

# Spectrum 108a PERL_HUMAN@106: ASLTNVTDPSLDLTSLSLEVGCGAPAPVVR_Deamidated(N)@5; Carbamidomethyl(C)@22 MW 3039.544, Precursor m/z 1014.19 charge 3+, delta(ppm) -1.5
?

## Slide 127
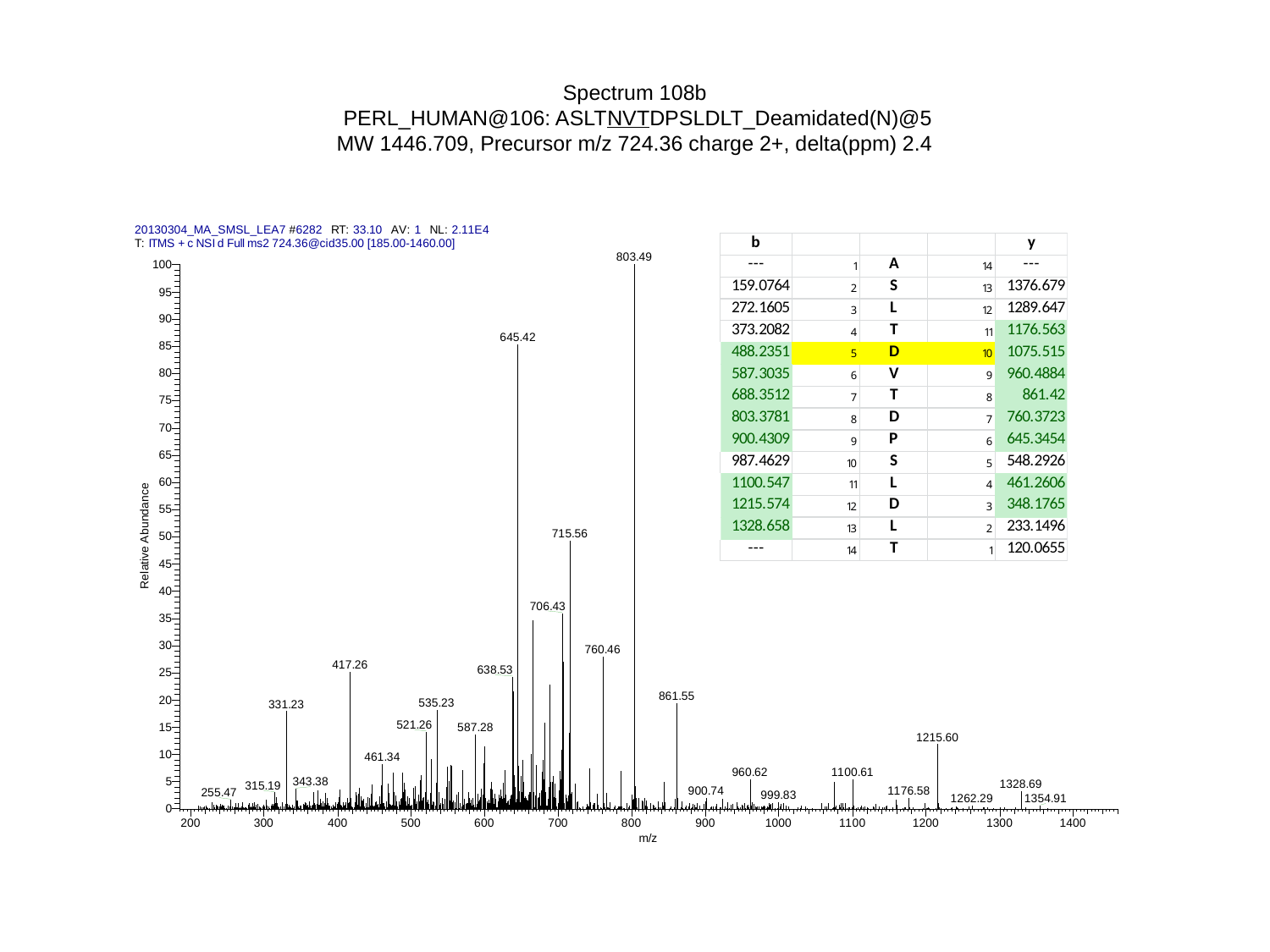

# Spectrum 108b PERL_HUMAN@106: ASLTNVTDPSLDLT_Deamidated(N)@5 MW 1446.709, Precursor m/z 724.36 charge 2+, delta(ppm) 2.4

## Slide 128
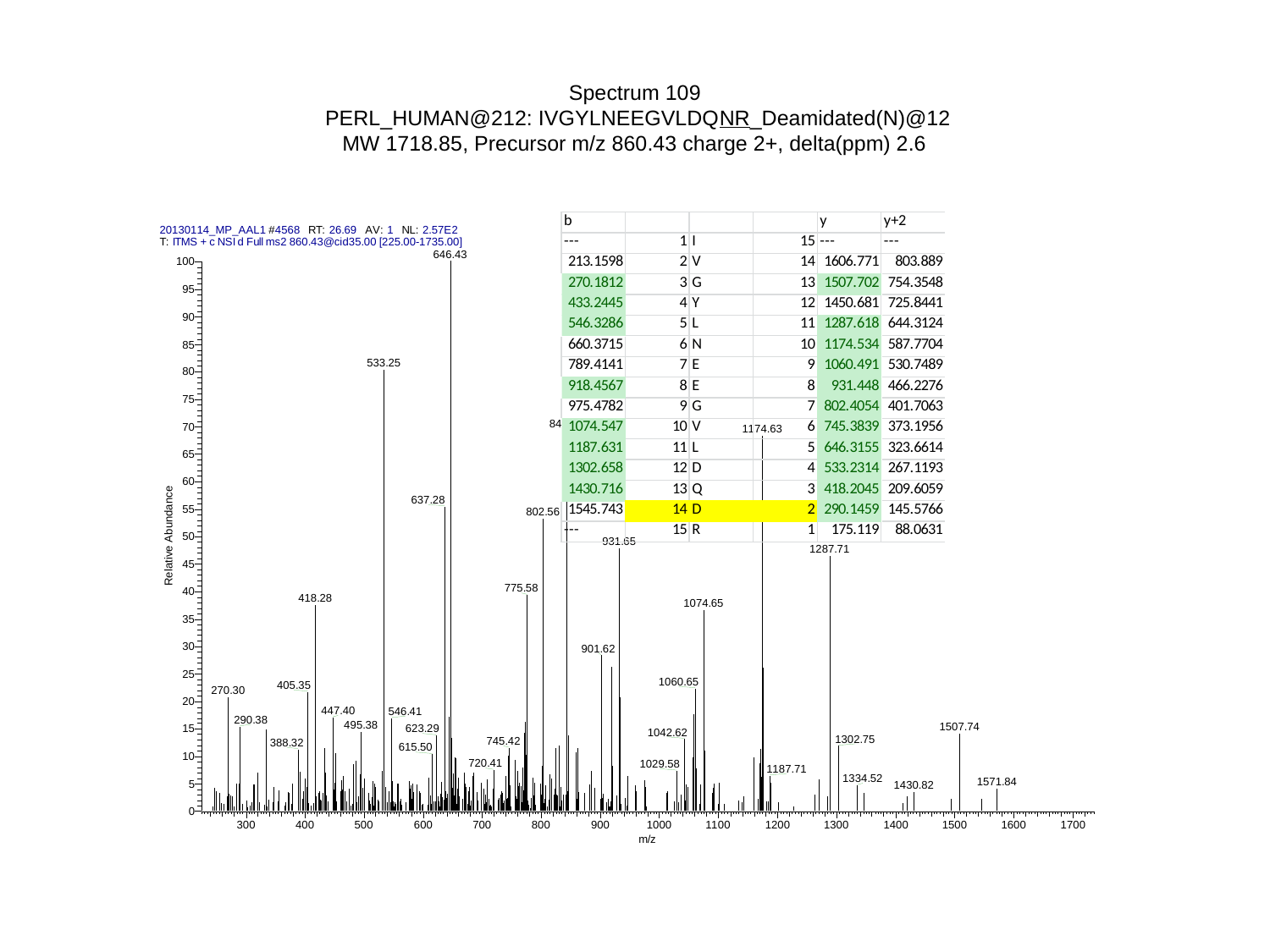

# Spectrum 109 PERL_HUMAN@212: IVGYLNEEGVLDQNR_Deamidated(N)@12 MW 1718.85, Precursor m/z 860.43 charge 2+, delta(ppm) 2.6

## Slide 129
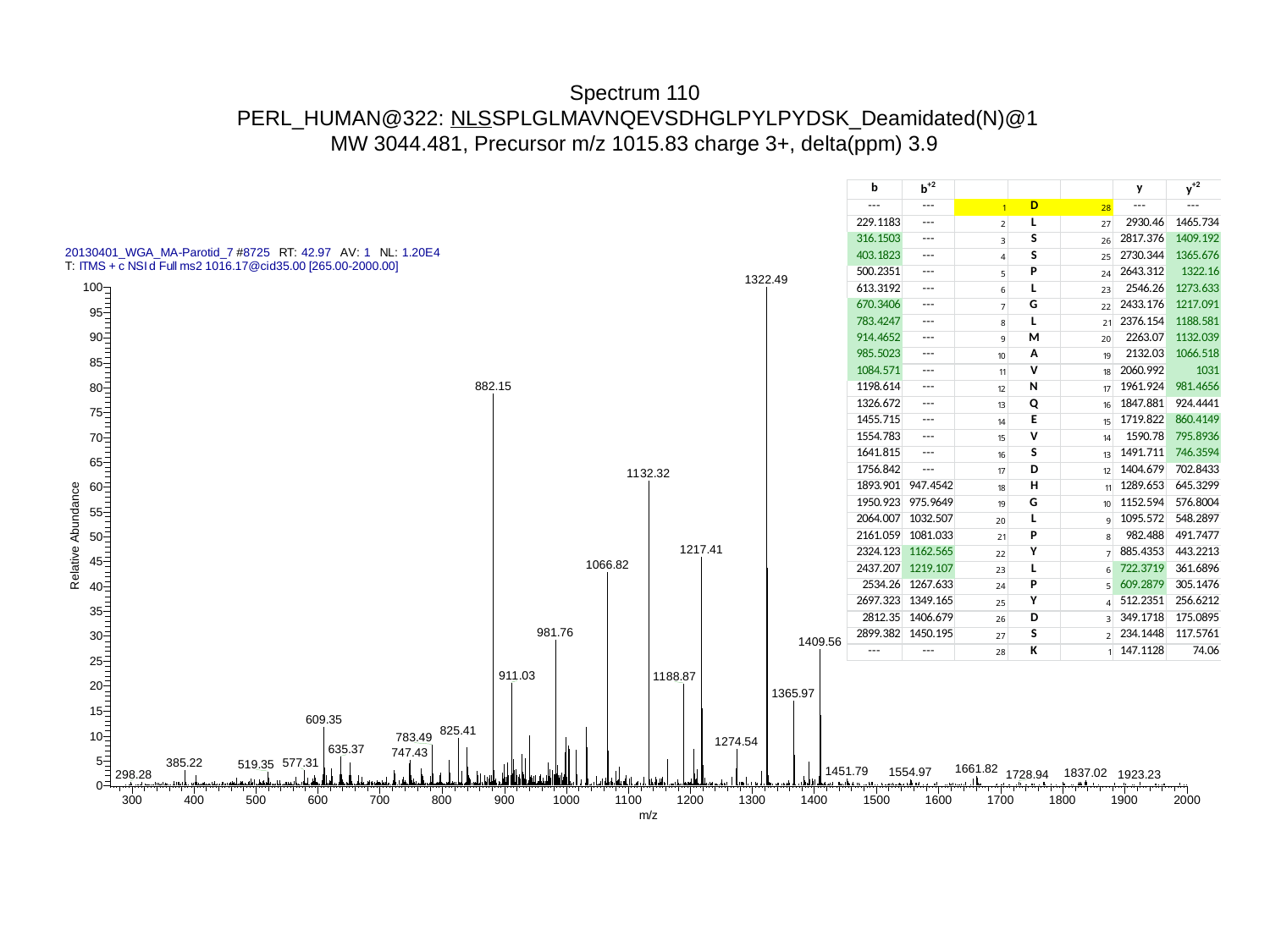

# Spectrum 110 PERL_HUMAN@322: NLSSPLGLMAVNQEVSDHGLPYLPYDSK_Deamidated(N)@1 MW 3044.481, Precursor m/z 1015.83 charge 3+, delta(ppm) 3.9

## Slide 130
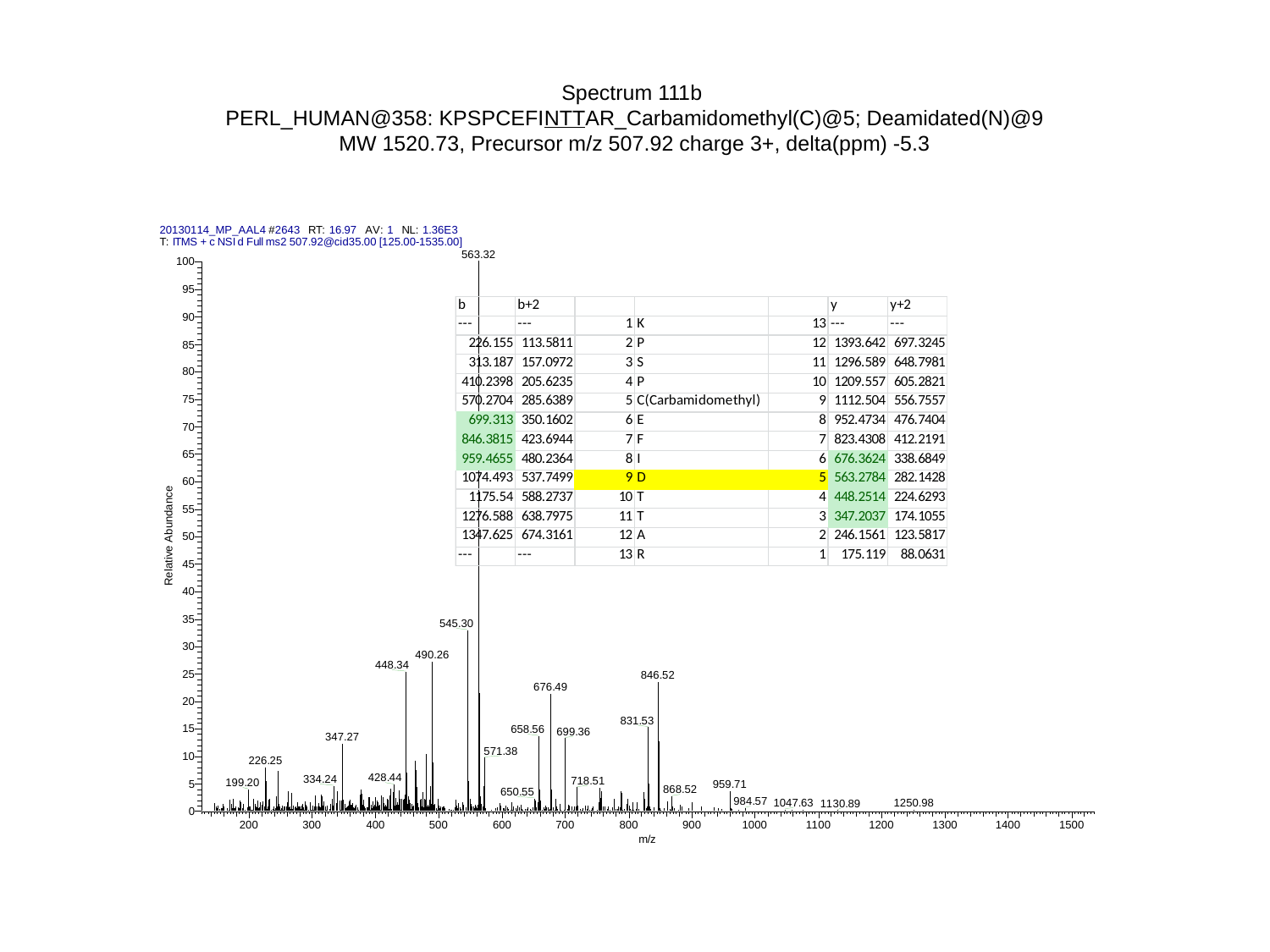

# Spectrum 111b PERL_HUMAN@358: KPSPCEFINTTAR_Carbamidomethyl(C)@5; Deamidated(N)@9 MW 1520.73, Precursor m/z 507.92 charge 3+, delta(ppm) -5.3

## Slide 131
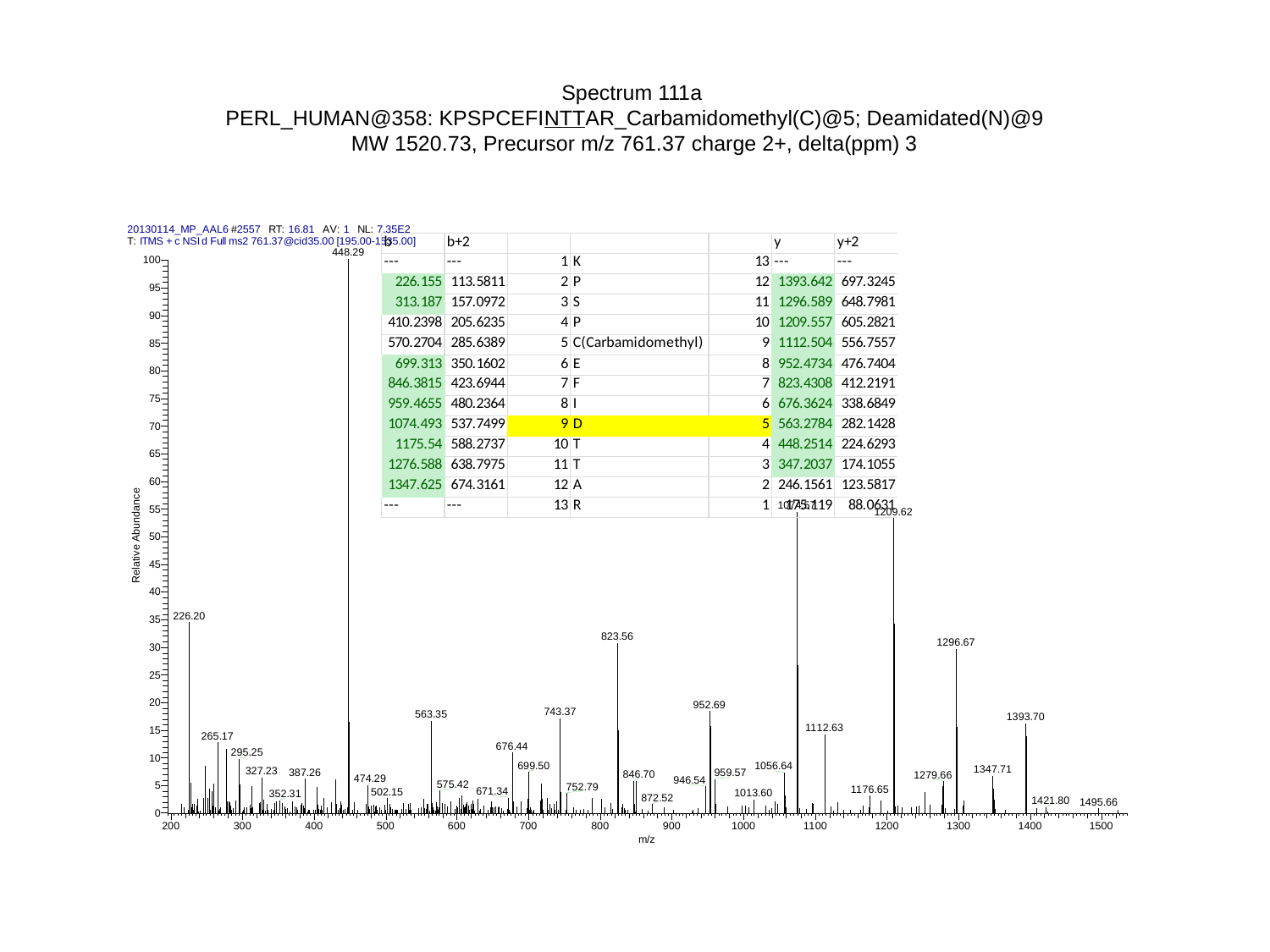

# Spectrum 111a PERL_HUMAN@358: KPSPCEFINTTAR_Carbamidomethyl(C)@5; Deamidated(N)@9 MW 1520.73, Precursor m/z 761.37 charge 2+, delta(ppm) 3

## Slide 132
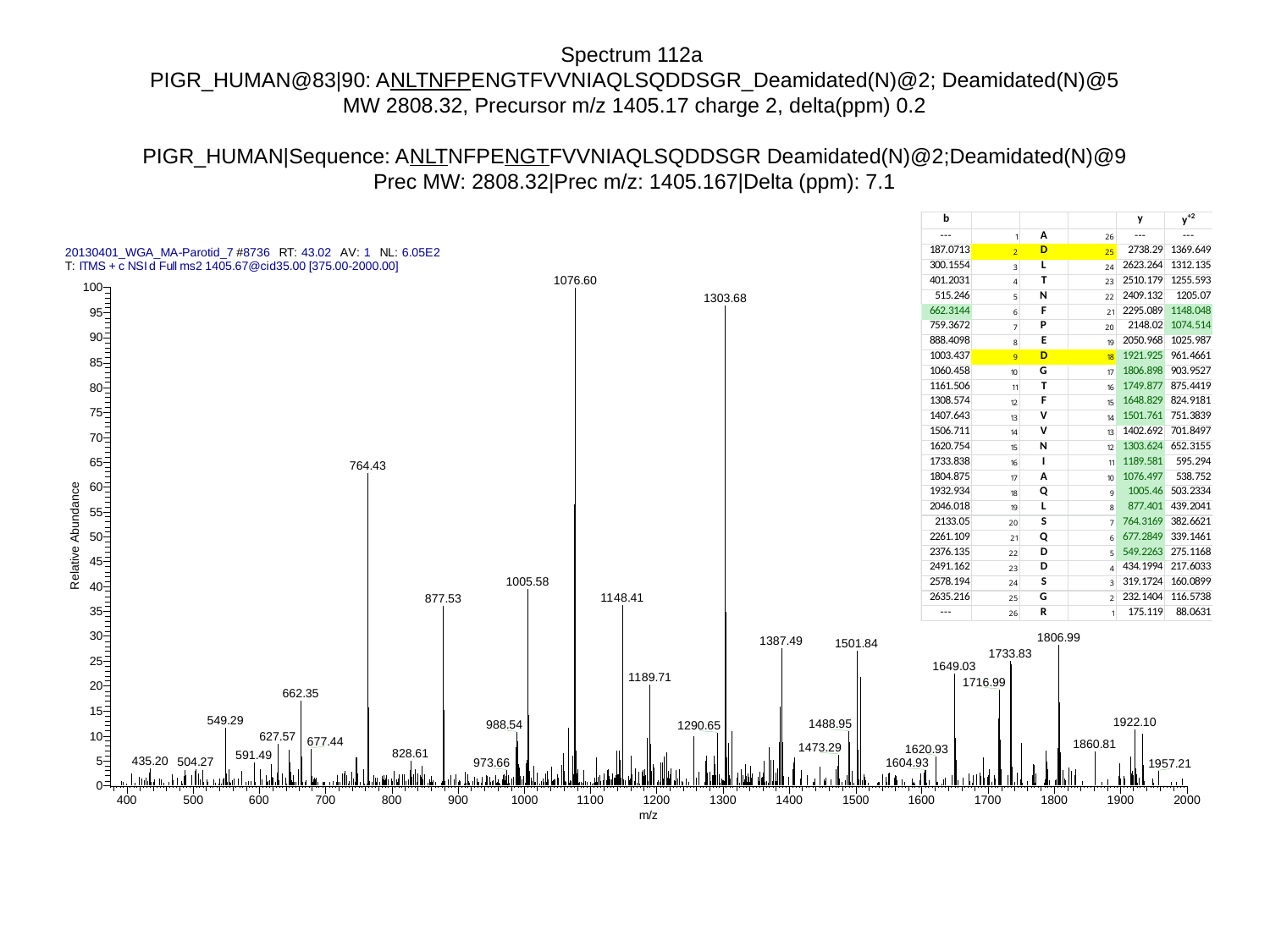

# Spectrum 112a PIGR_HUMAN@83|90: ANLTNFPENGTFVVNIAQLSQDDSGR_Deamidated(N)@2; Deamidated(N)@5 MW 2808.32, Precursor m/z 1405.17 charge 2, delta(ppm) 0.2 PIGR_HUMAN|Sequence: ANLTNFPENGTFVVNIAQLSQDDSGR Deamidated(N)@2;Deamidated(N)@9 Prec MW: 2808.32|Prec m/z: 1405.167|Delta (ppm): 7.1

## Slide 133
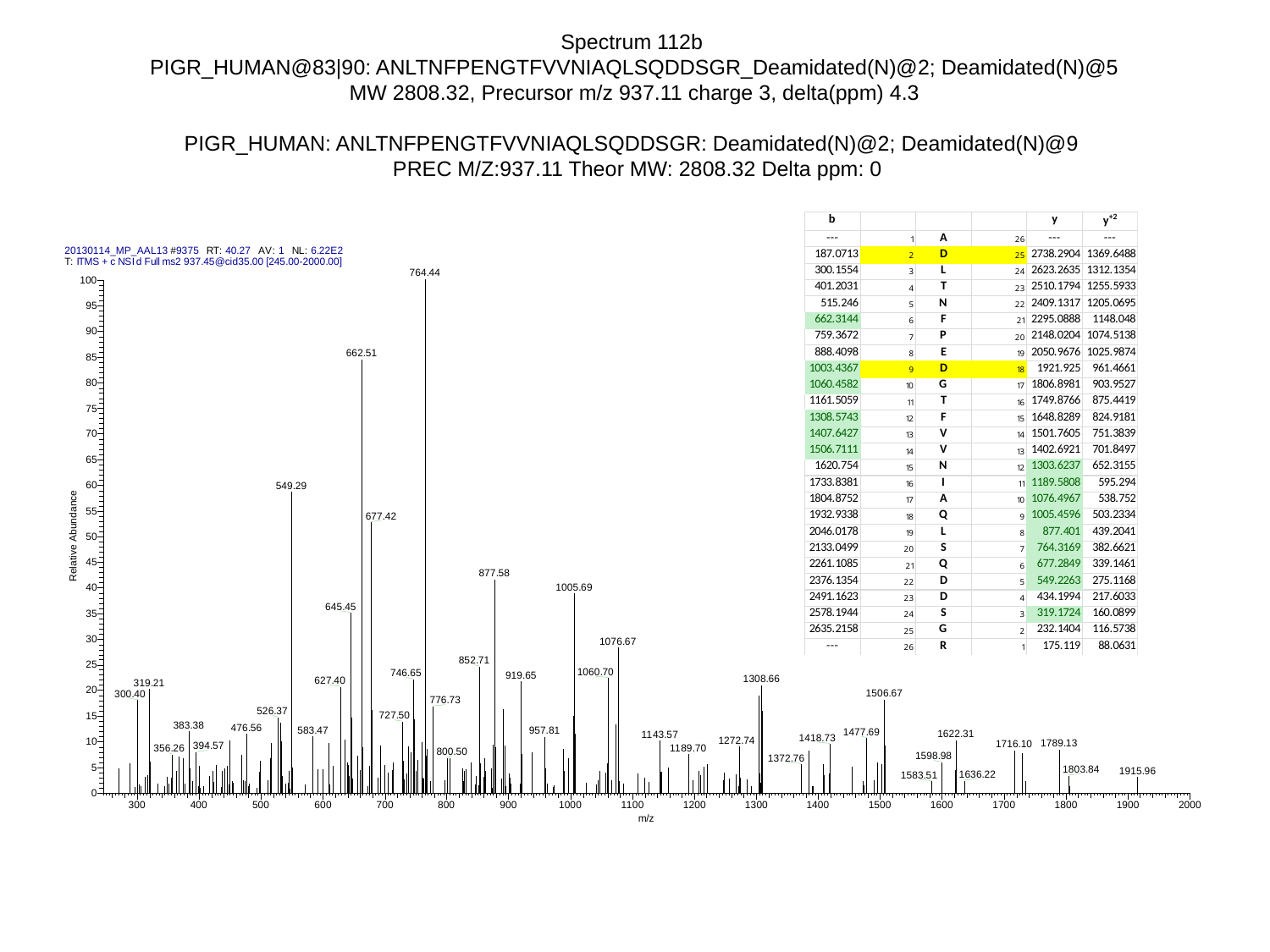

# Spectrum 112b PIGR_HUMAN@83|90: ANLTNFPENGTFVVNIAQLSQDDSGR_Deamidated(N)@2; Deamidated(N)@5 MW 2808.32, Precursor m/z 937.11 charge 3, delta(ppm) 4.3 PIGR_HUMAN: ANLTNFPENGTFVVNIAQLSQDDSGR: Deamidated(N)@2; Deamidated(N)@9 PREC M/Z:937.11 Theor MW: 2808.32 Delta ppm: 0

## Slide 134
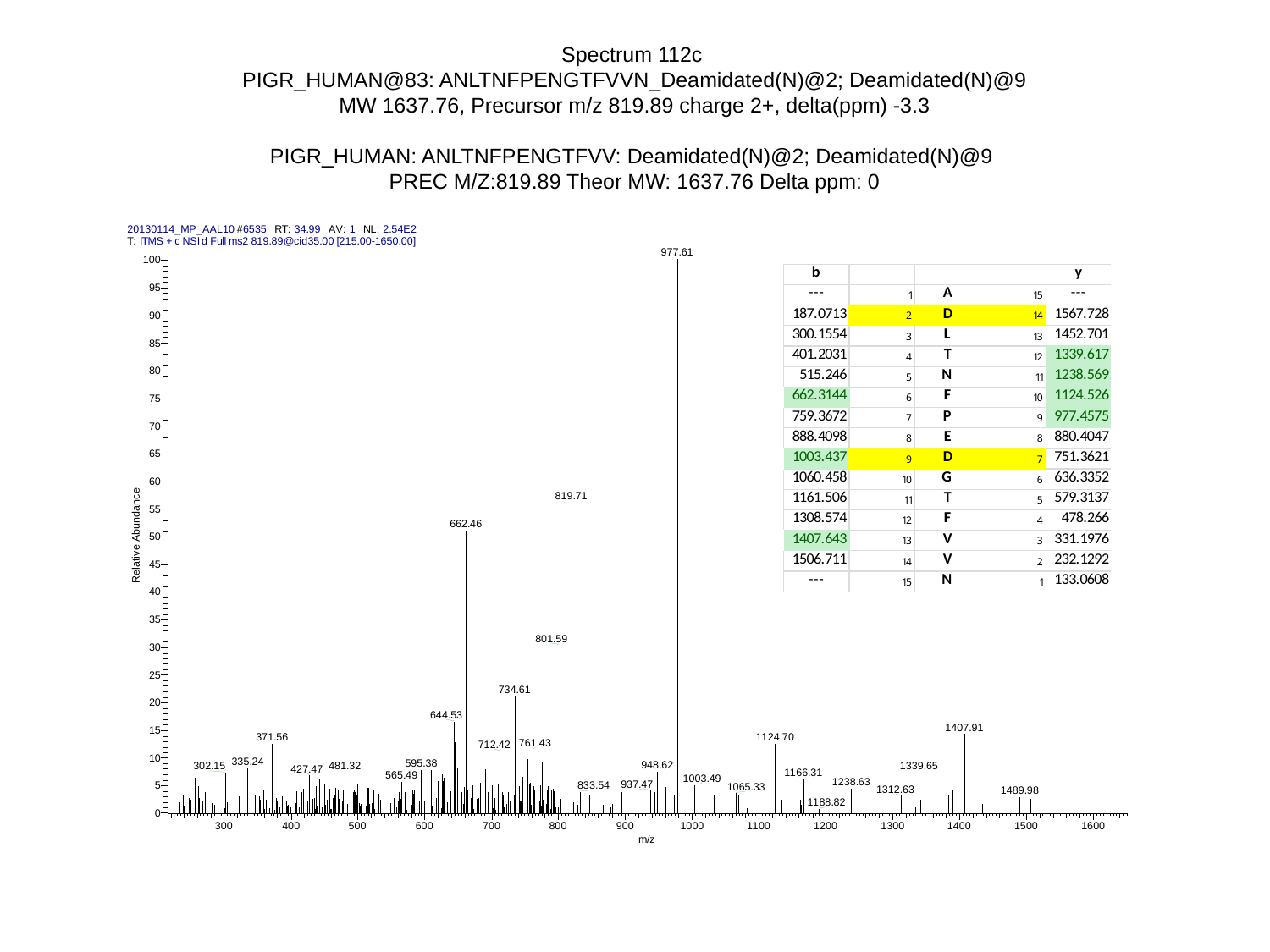

# Spectrum 112c PIGR_HUMAN@83: ANLTNFPENGTFVVN_Deamidated(N)@2; Deamidated(N)@9 MW 1637.76, Precursor m/z 819.89 charge 2+, delta(ppm) -3.3 PIGR_HUMAN: ANLTNFPENGTFVV: Deamidated(N)@2; Deamidated(N)@9 PREC M/Z:819.89 Theor MW: 1637.76 Delta ppm: 0

## Slide 135
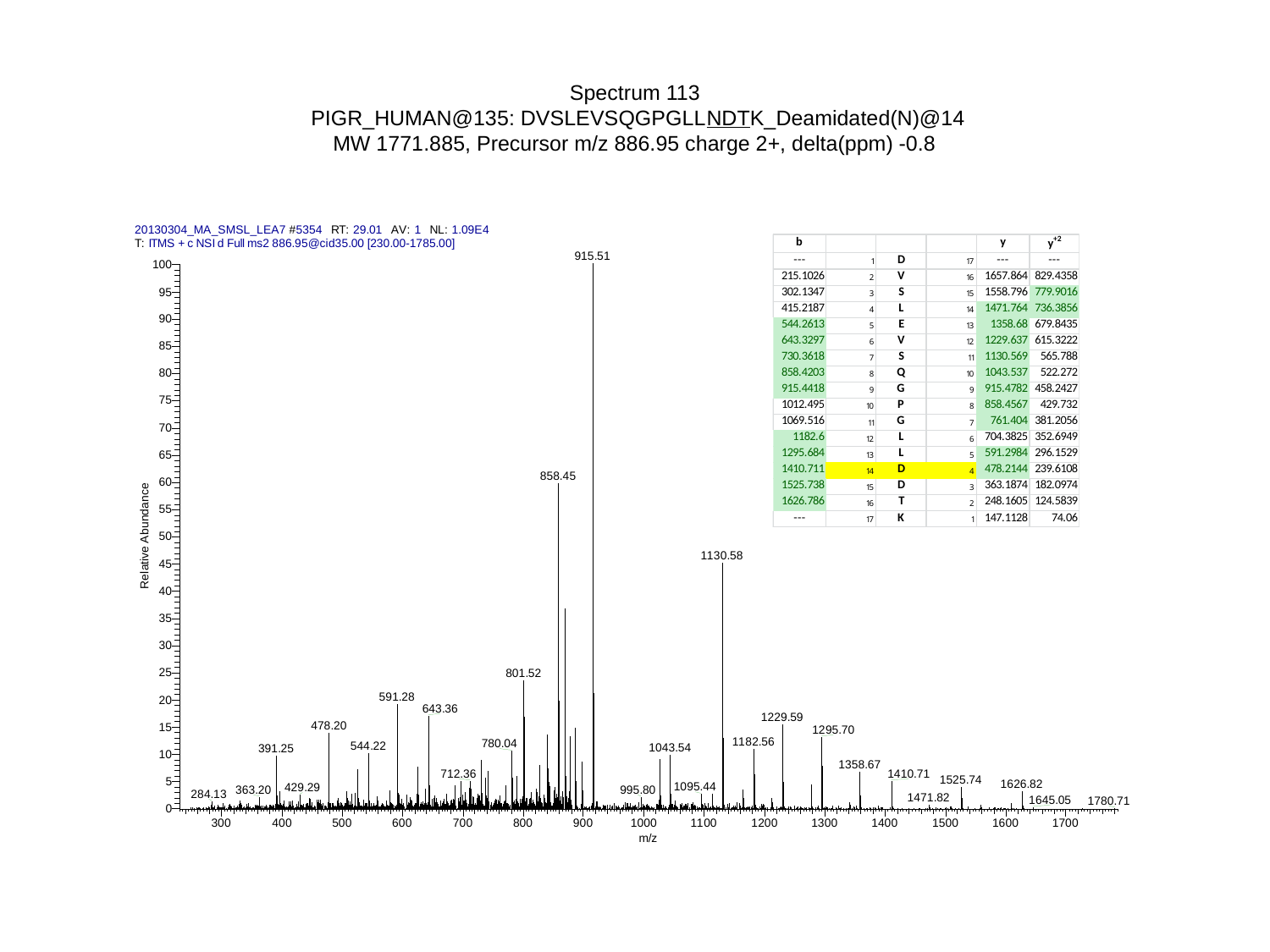

# Spectrum 113 PIGR_HUMAN@135: DVSLEVSQGPGLLNDTK_Deamidated(N)@14 MW 1771.885, Precursor m/z 886.95 charge 2+, delta(ppm) -0.8

## Slide 136
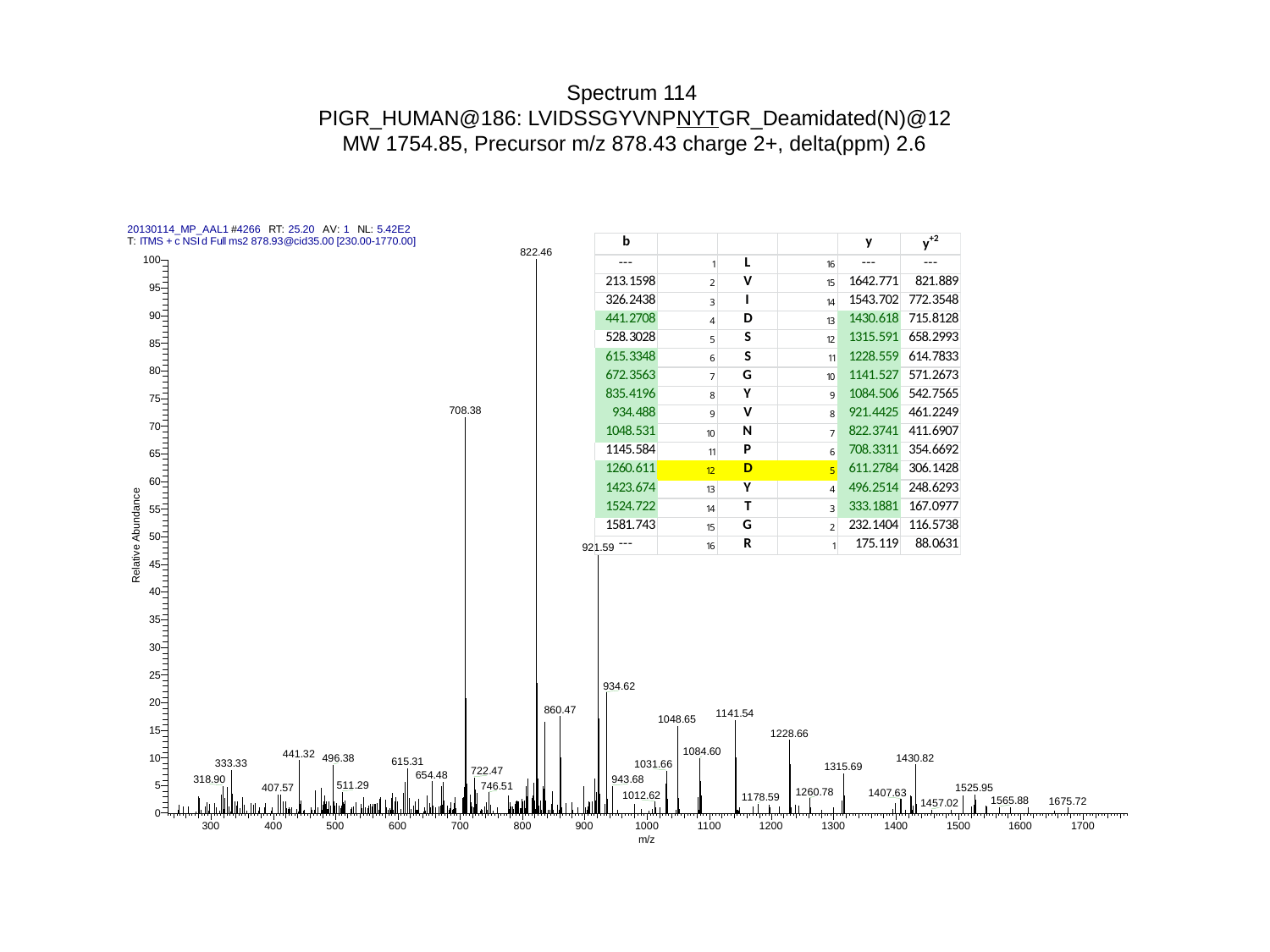

# Spectrum 114 PIGR_HUMAN@186: LVIDSSGYVNPNYTGR_Deamidated(N)@12 MW 1754.85, Precursor m/z 878.43 charge 2+, delta(ppm) 2.6

## Slide 137
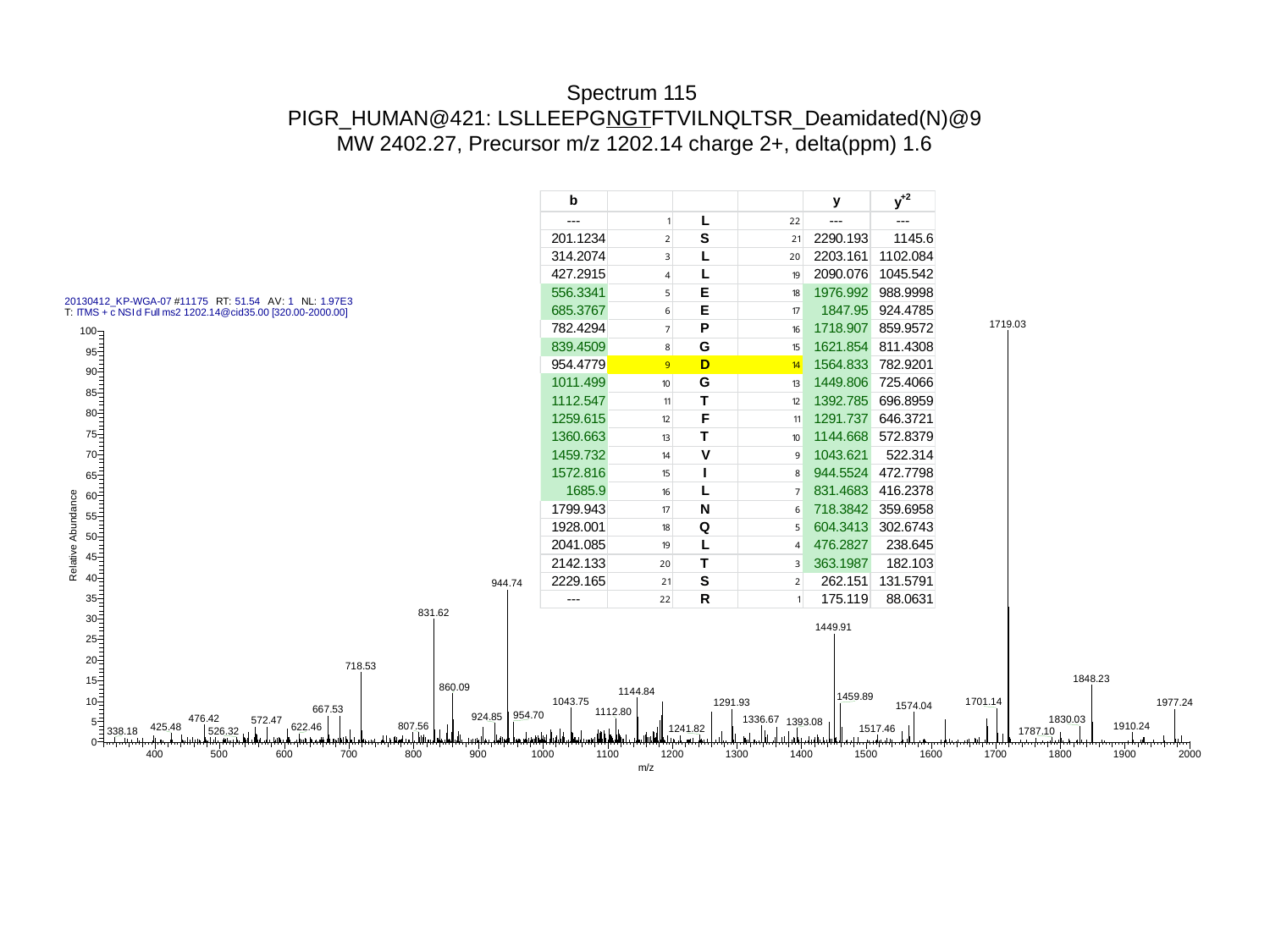

# Spectrum 115 PIGR_HUMAN@421: LSLLEEPGNGTFTVILNQLTSR_Deamidated(N)@9 MW 2402.27, Precursor m/z 1202.14 charge 2+, delta(ppm) 1.6

## Slide 138
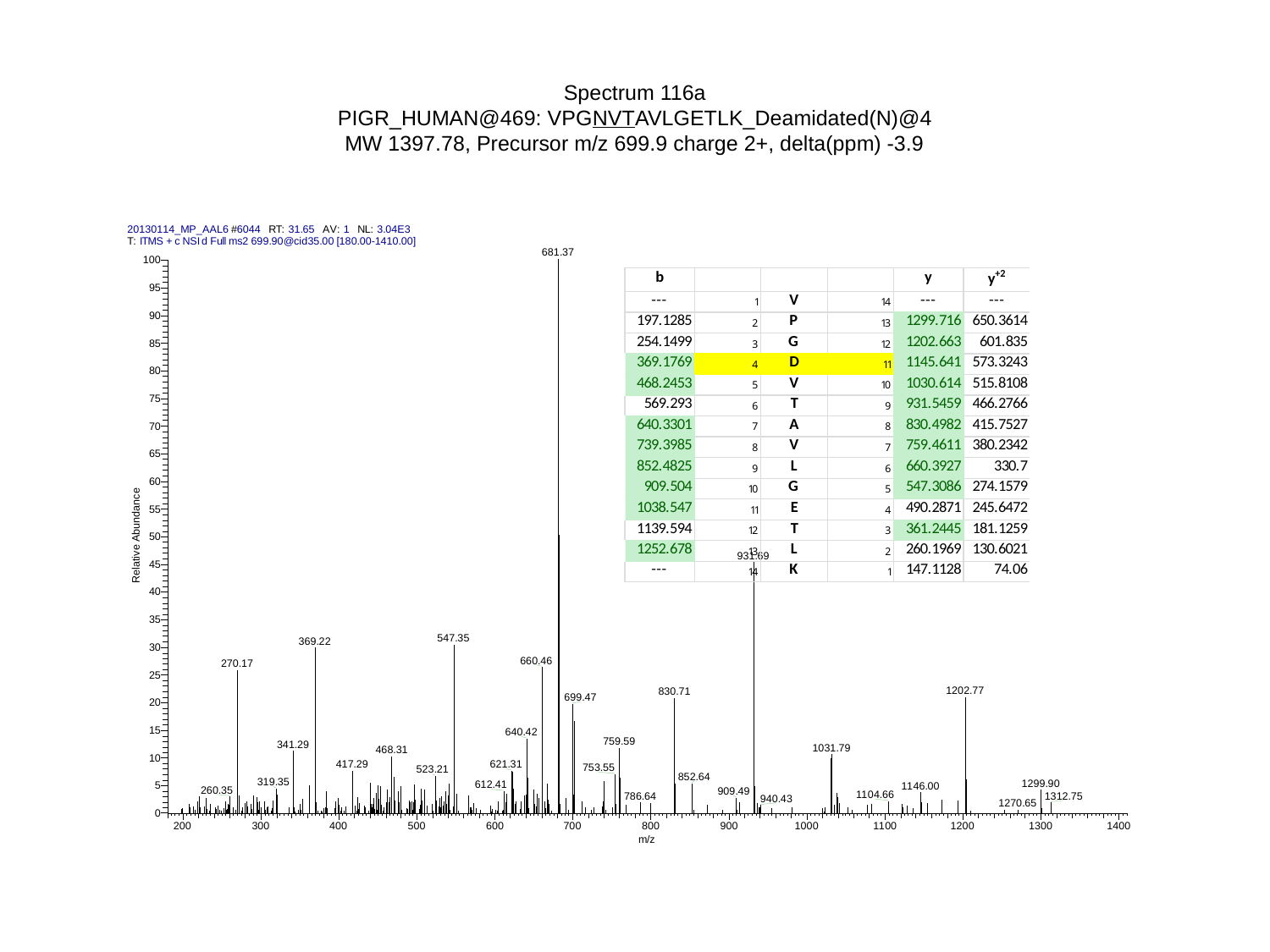

# Spectrum 116a PIGR_HUMAN@469: VPGNVTAVLGETLK_Deamidated(N)@4 MW 1397.78, Precursor m/z 699.9 charge 2+, delta(ppm) -3.9

## Slide 139
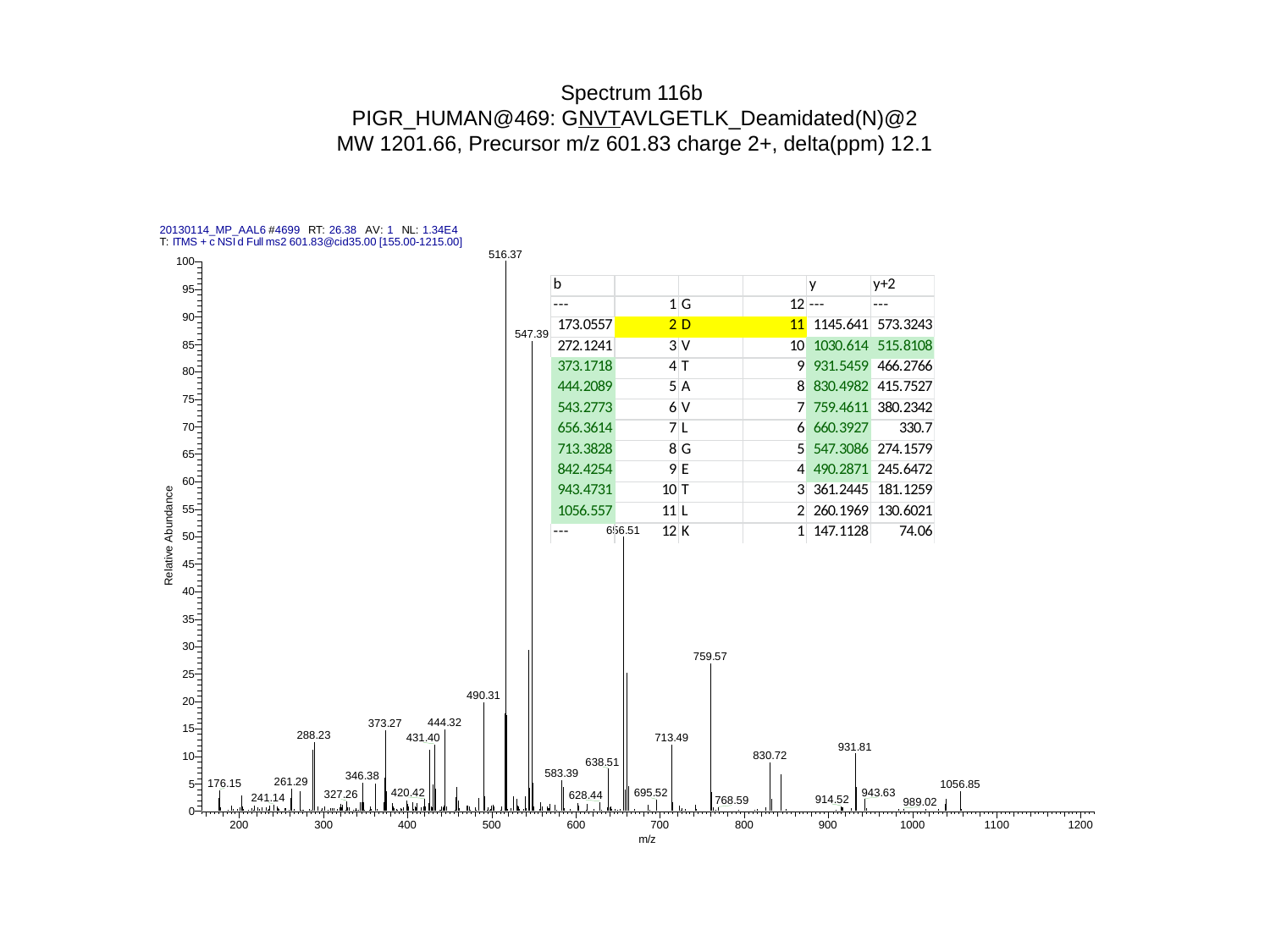

# Spectrum 116b PIGR_HUMAN@469: GNVTAVLGETLK_Deamidated(N)@2 MW 1201.66, Precursor m/z 601.83 charge 2+, delta(ppm) 12.1

## Slide 140
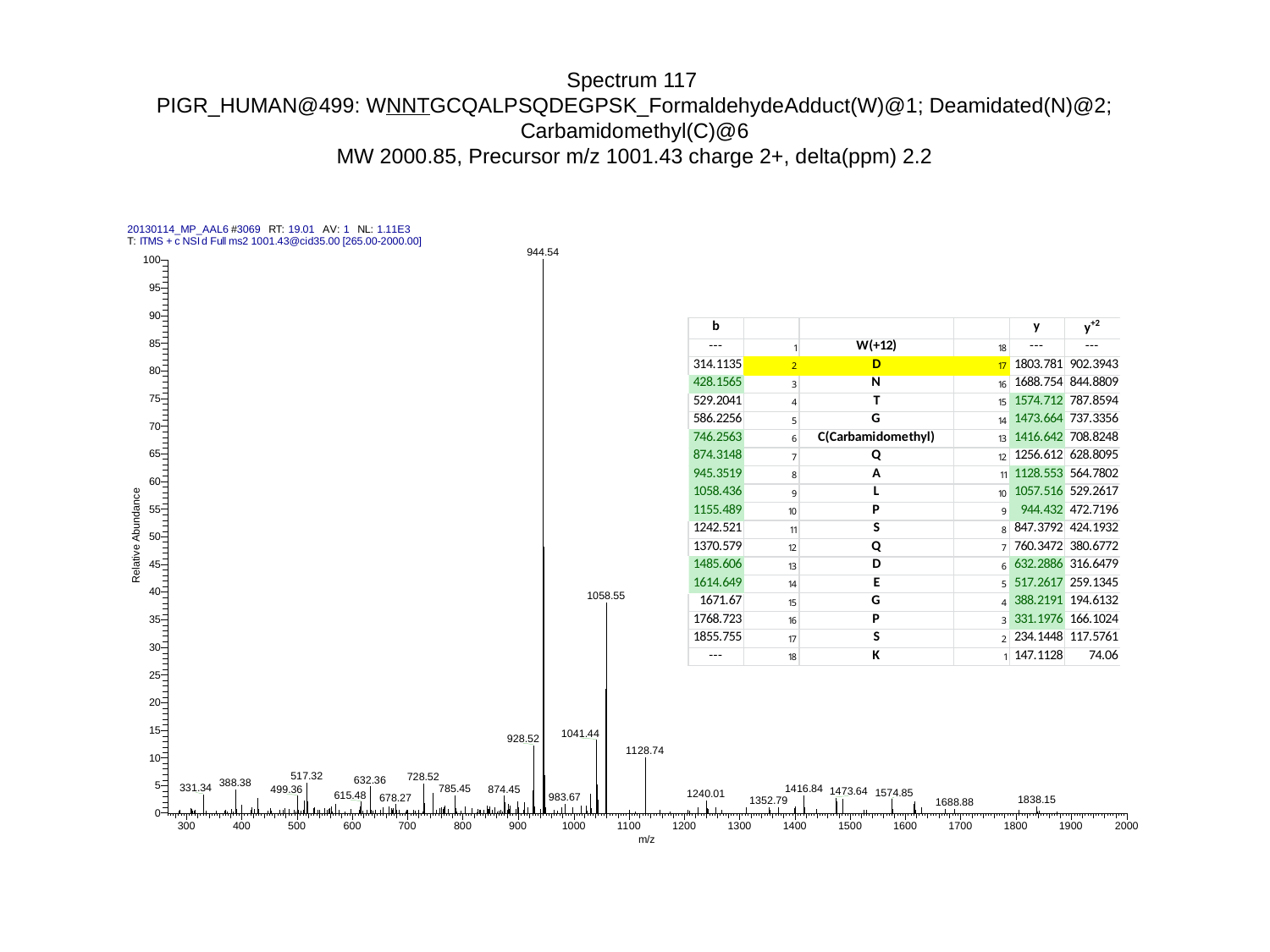

# Spectrum 117 PIGR_HUMAN@499: WNNTGCQALPSQDEGPSK_FormaldehydeAdduct(W)@1; Deamidated(N)@2; Carbamidomethyl(C)@6 MW 2000.85, Precursor m/z 1001.43 charge 2+, delta(ppm) 2.2

## Slide 141
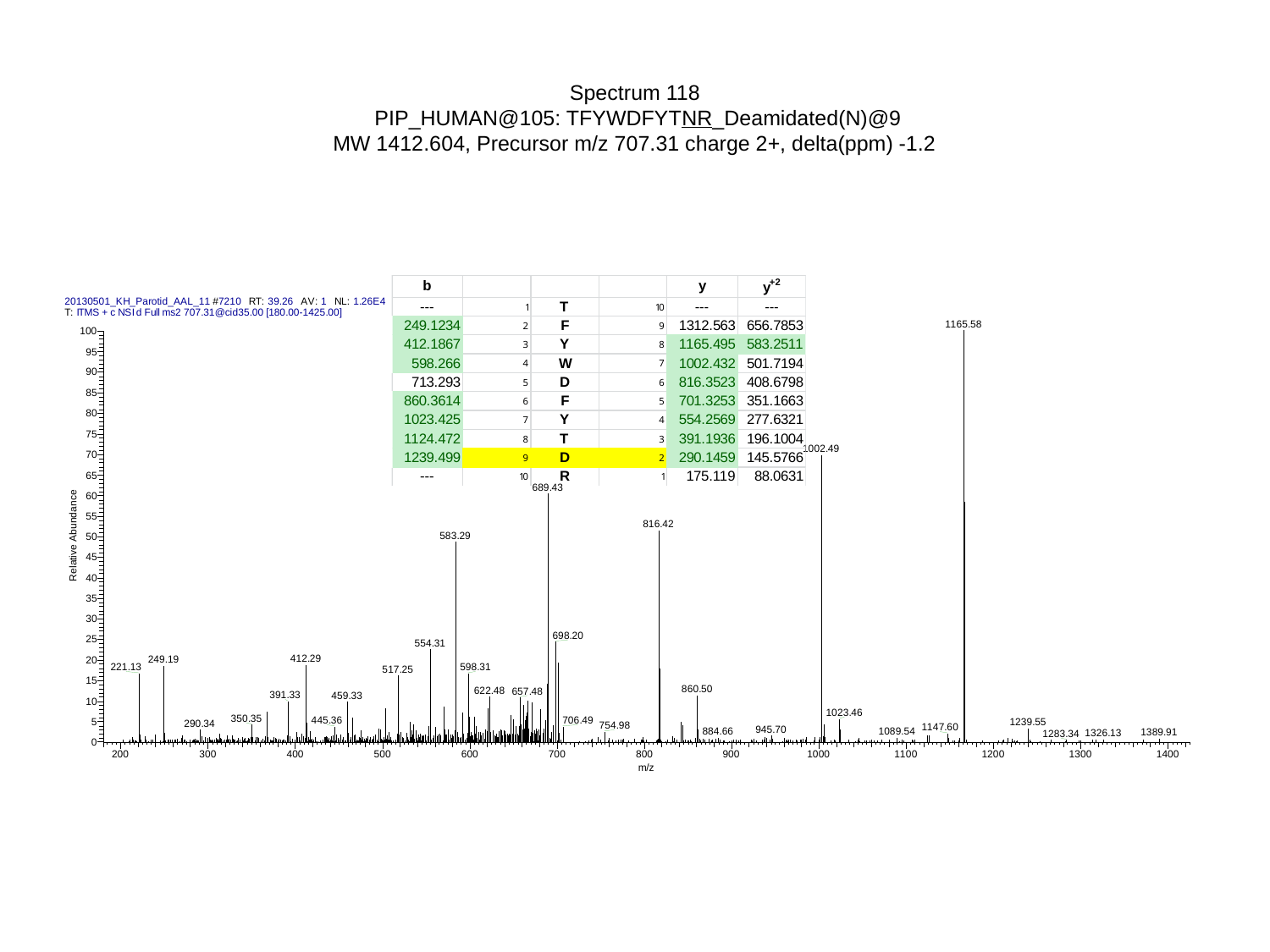

# Spectrum 118 PIP_HUMAN@105: TFYWDFYTNR_Deamidated(N)@9 MW 1412.604, Precursor m/z 707.31 charge 2+, delta(ppm) -1.2

## Slide 142
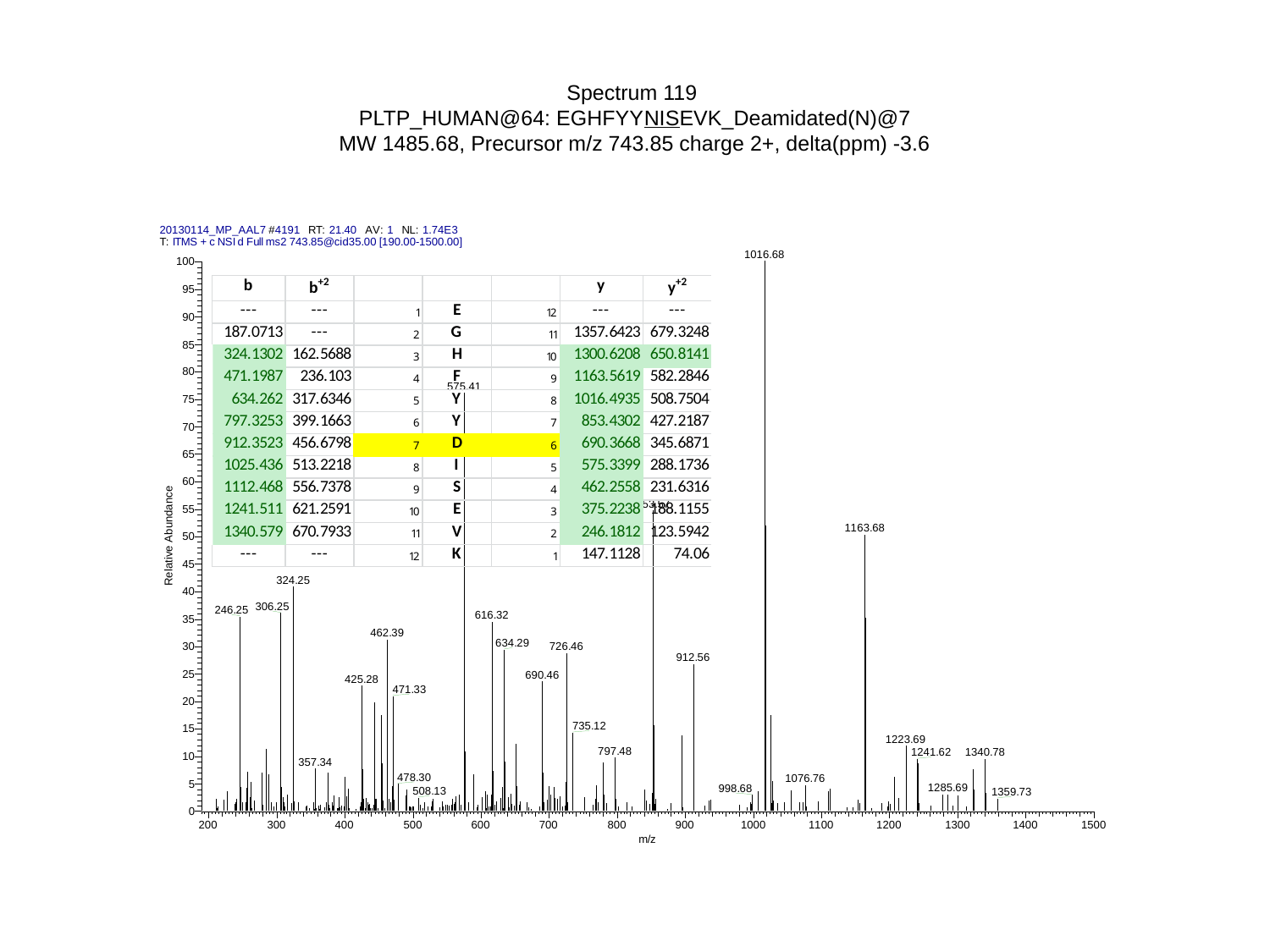

# Spectrum 119 PLTP_HUMAN@64: EGHFYYNISEVK_Deamidated(N)@7 MW 1485.68, Precursor m/z 743.85 charge 2+, delta(ppm) -3.6

## Slide 143
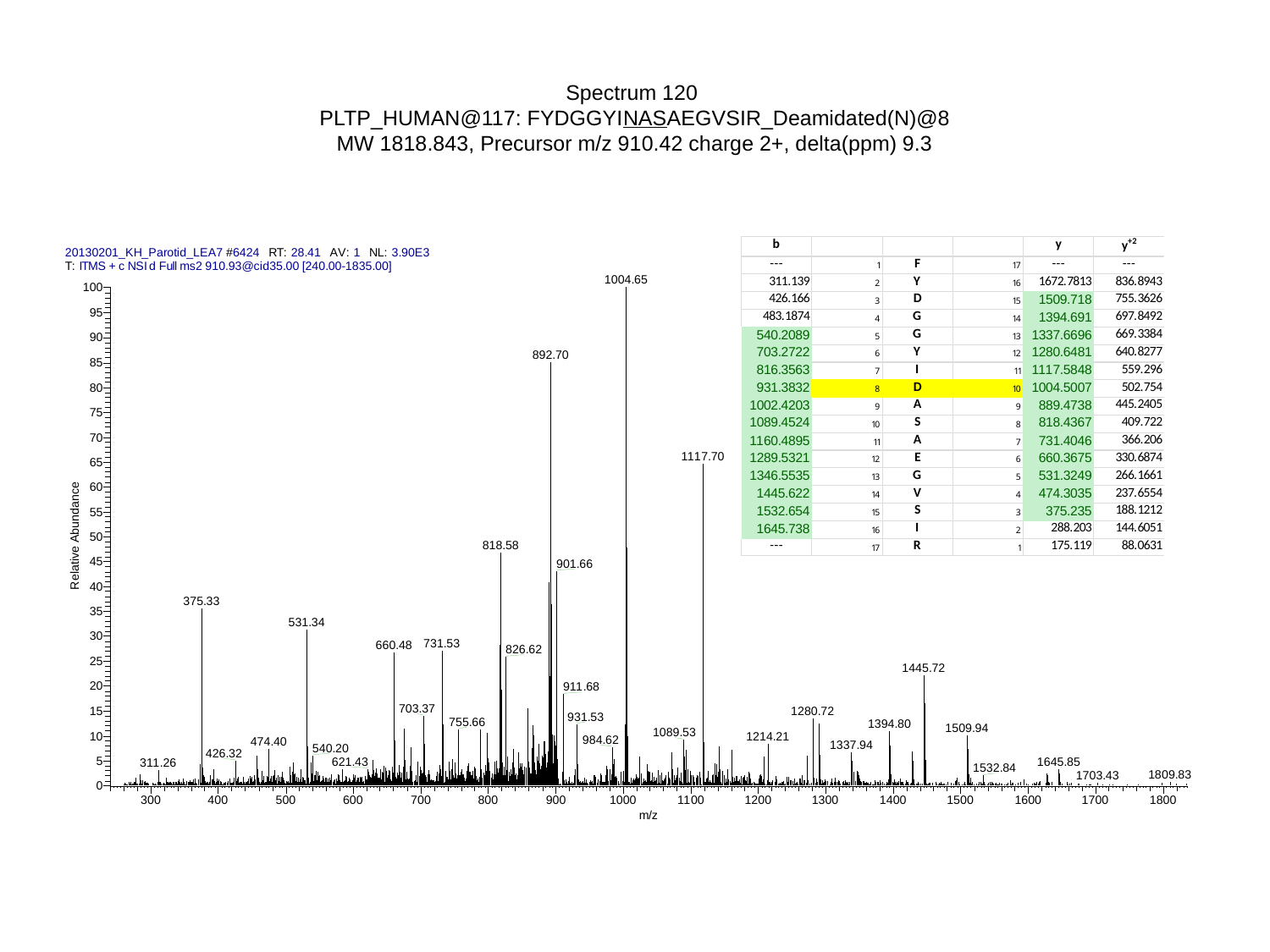

# Spectrum 120 PLTP_HUMAN@117: FYDGGYINASAEGVSIR_Deamidated(N)@8 MW 1818.843, Precursor m/z 910.42 charge 2+, delta(ppm) 9.3

## Slide 144
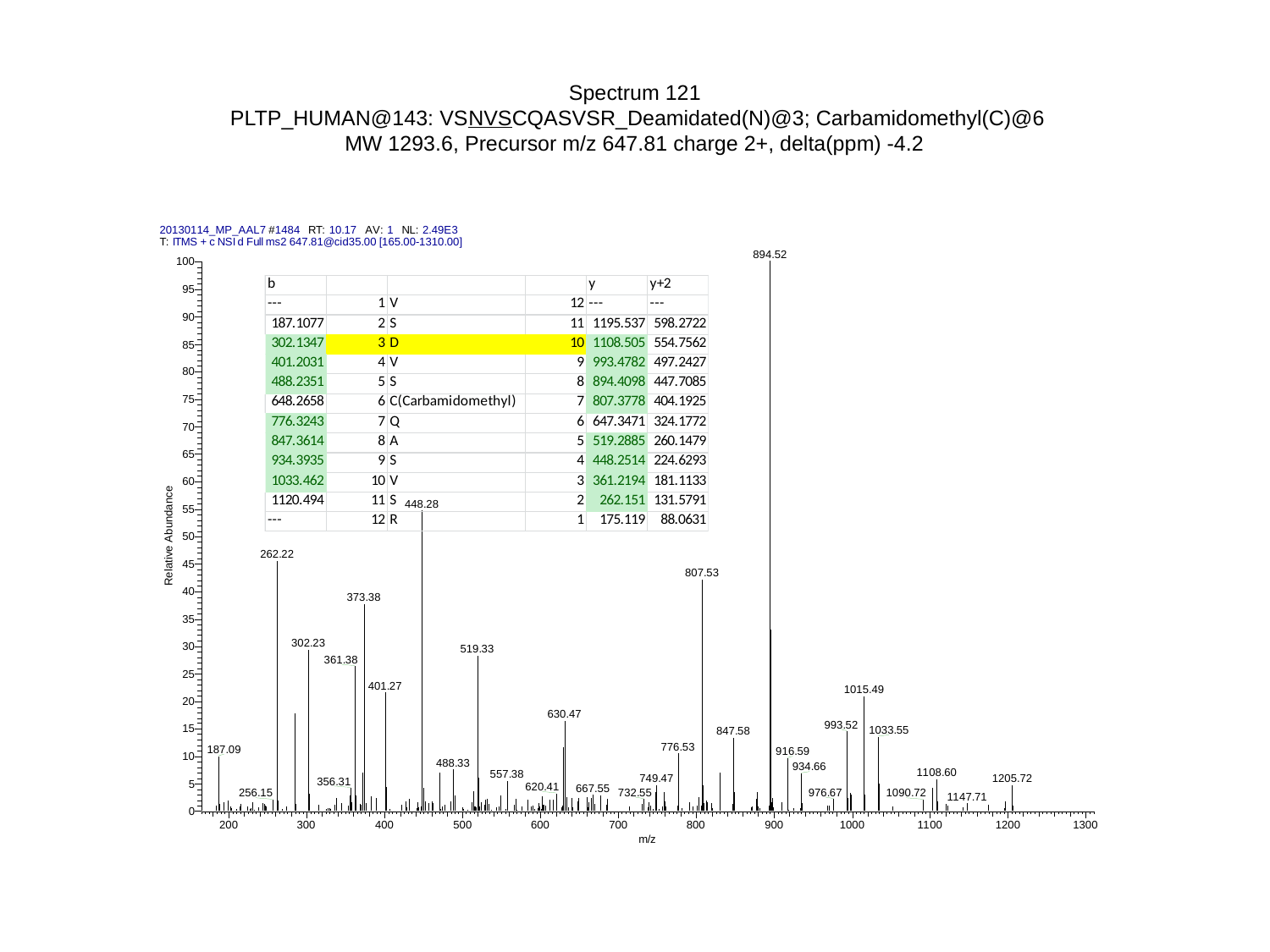

# Spectrum 121 PLTP_HUMAN@143: VSNVSCQASVSR_Deamidated(N)@3; Carbamidomethyl(C)@6 MW 1293.6, Precursor m/z 647.81 charge 2+, delta(ppm) -4.2

## Slide 145
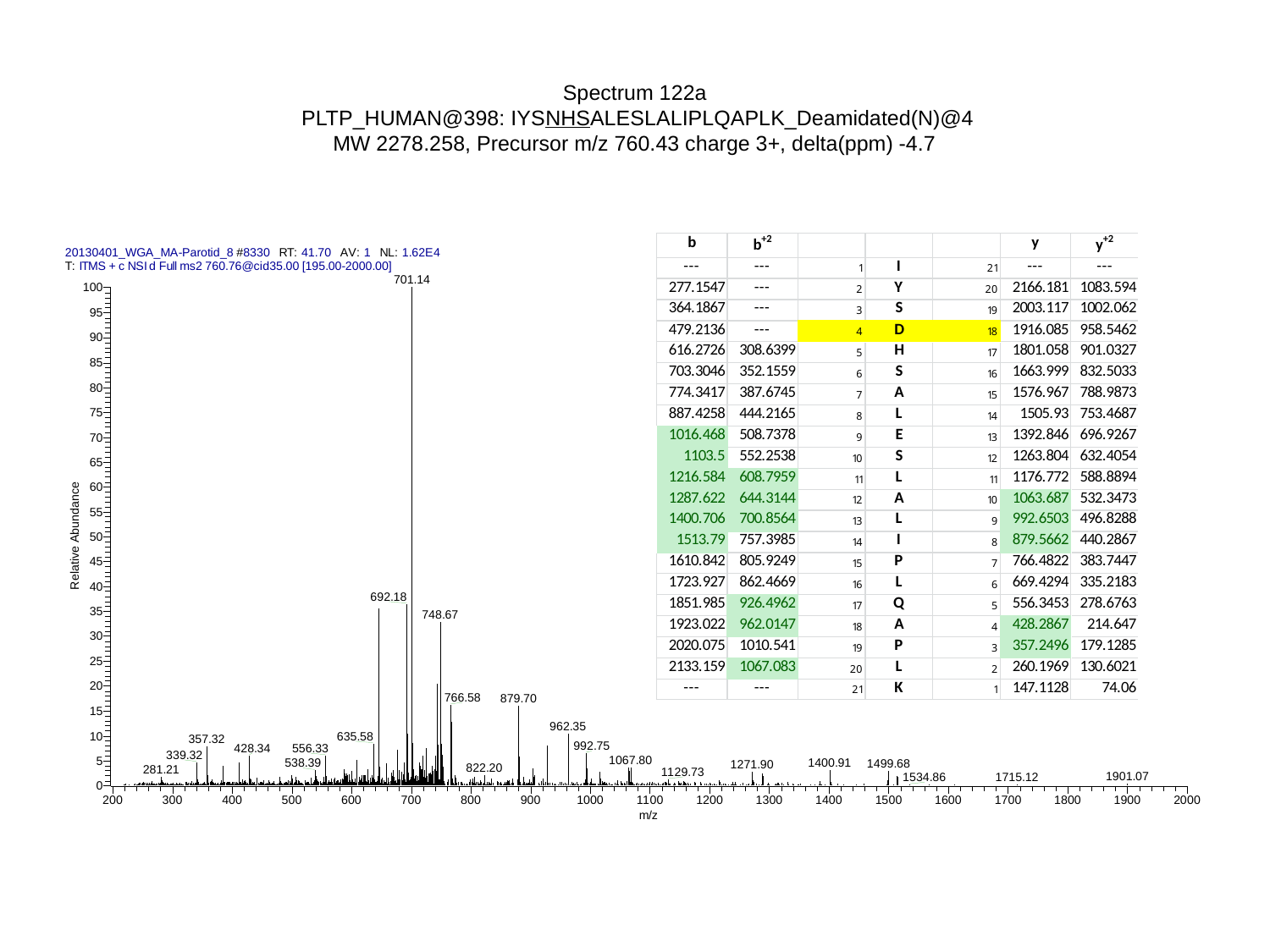

# Spectrum 122a PLTP_HUMAN@398: IYSNHSALESLALIPLQAPLK_Deamidated(N)@4 MW 2278.258, Precursor m/z 760.43 charge 3+, delta(ppm) -4.7

## Slide 146
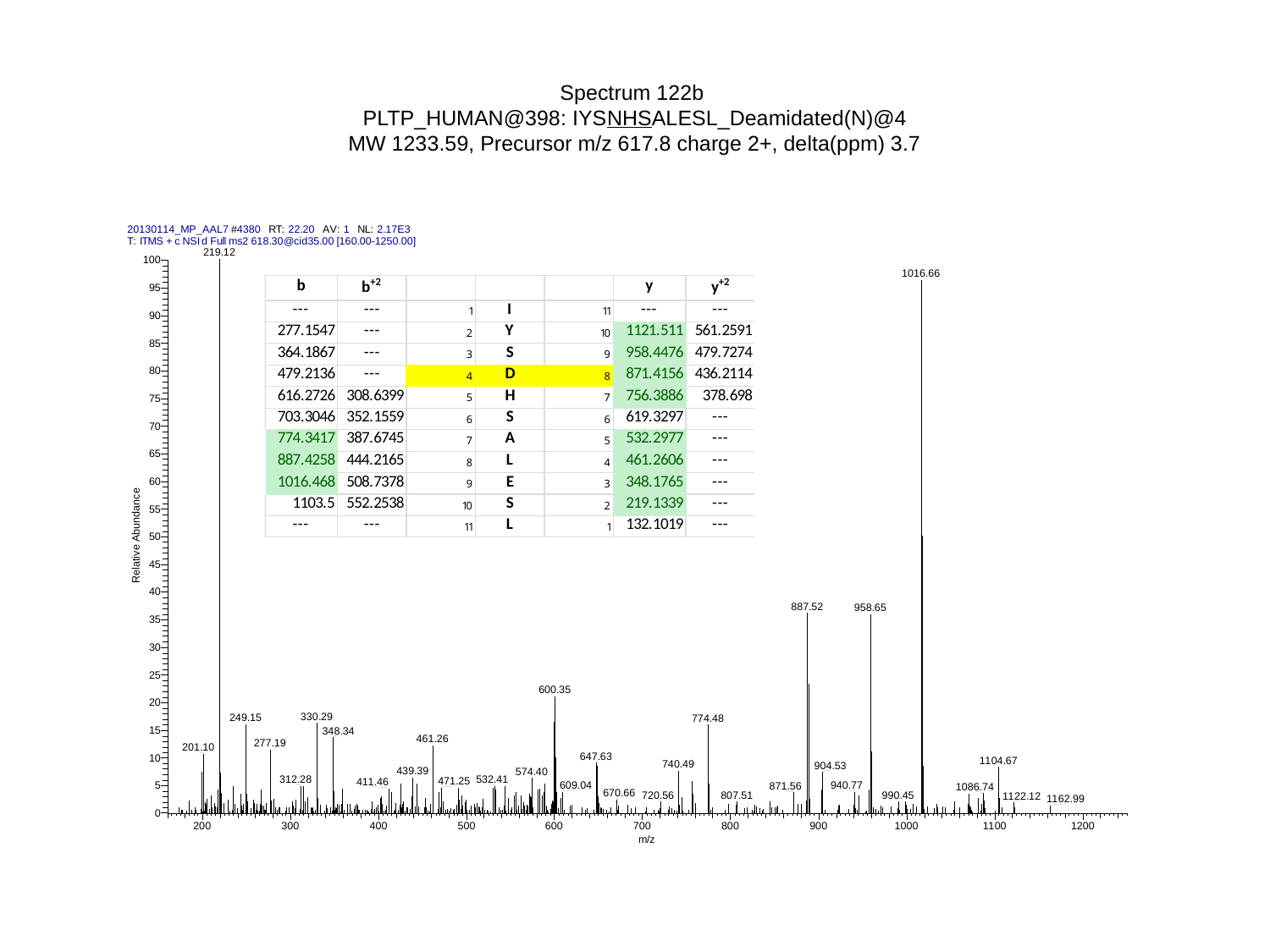

# Spectrum 122b PLTP_HUMAN@398: IYSNHSALESL_Deamidated(N)@4 MW 1233.59, Precursor m/z 617.8 charge 2+, delta(ppm) 3.7

## Slide 147
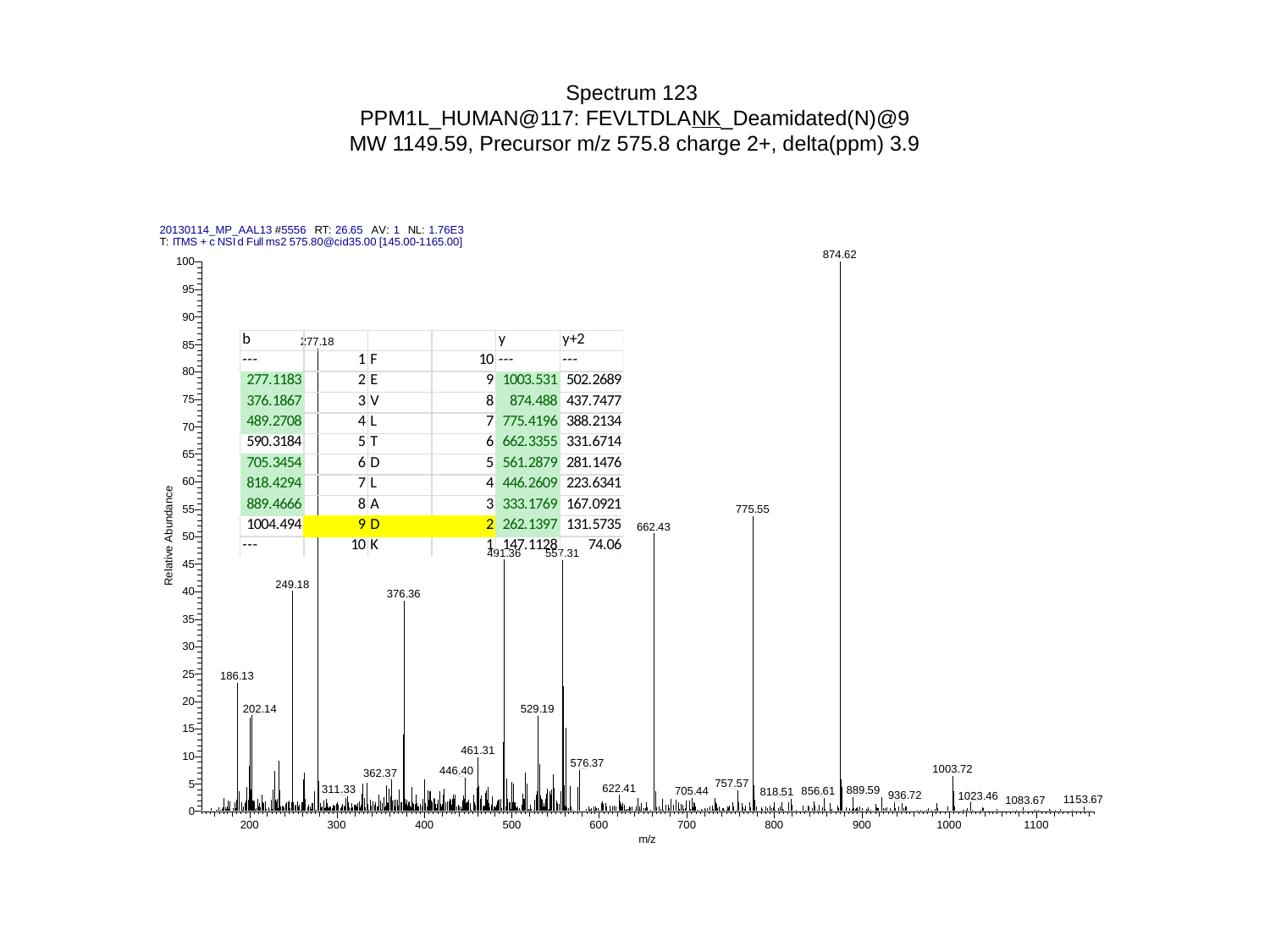

# Spectrum 123 PPM1L_HUMAN@117: FEVLTDLANK_Deamidated(N)@9 MW 1149.59, Precursor m/z 575.8 charge 2+, delta(ppm) 3.9

## Slide 148
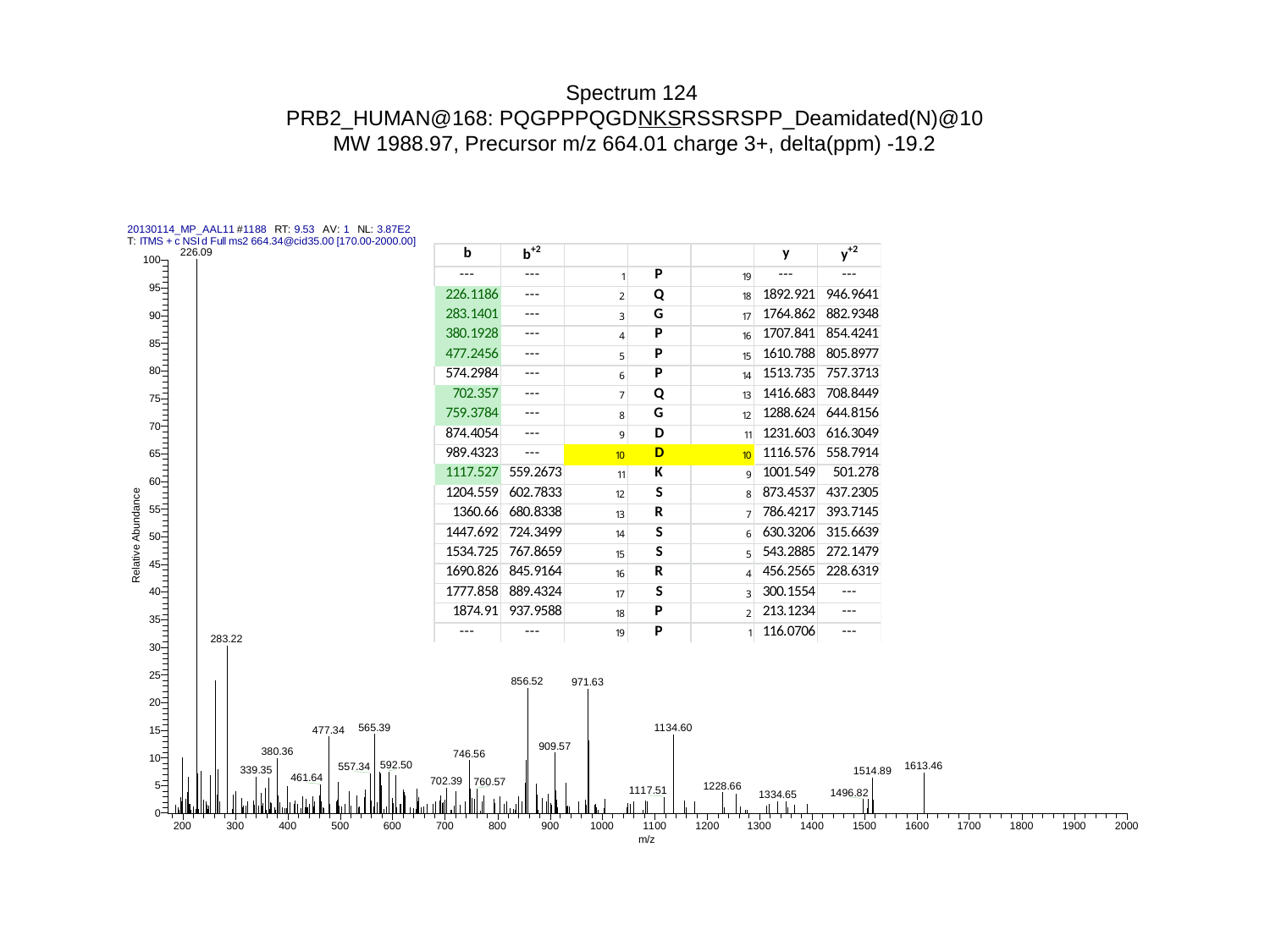

# Spectrum 124 PRB2_HUMAN@168: PQGPPPQGDNKSRSSRSPP_Deamidated(N)@10 MW 1988.97, Precursor m/z 664.01 charge 3+, delta(ppm) -19.2

## Slide 149
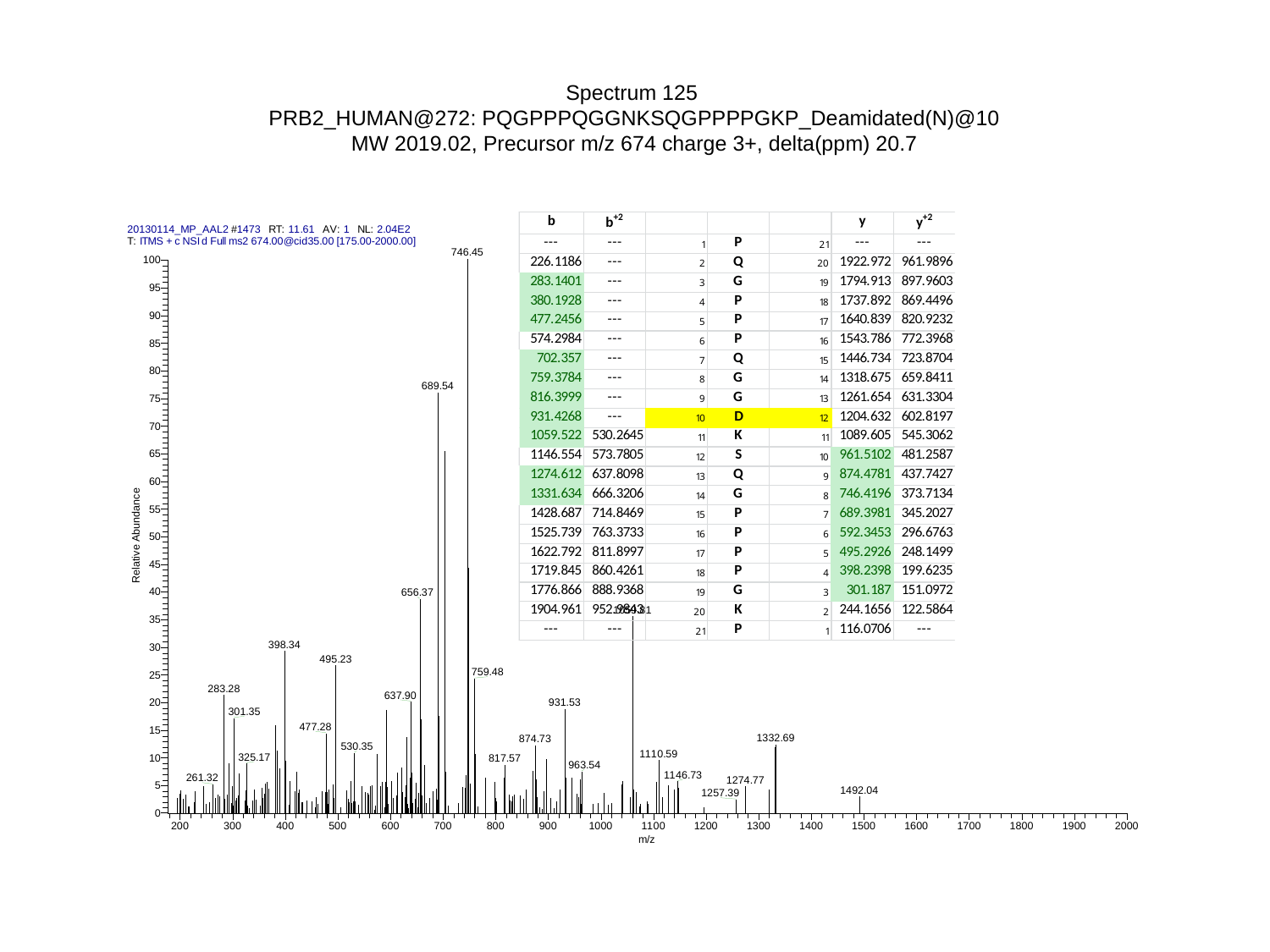

# Spectrum 125 PRB2_HUMAN@272: PQGPPPQGGNKSQGPPPPGKP_Deamidated(N)@10 MW 2019.02, Precursor m/z 674 charge 3+, delta(ppm) 20.7

## Slide 150
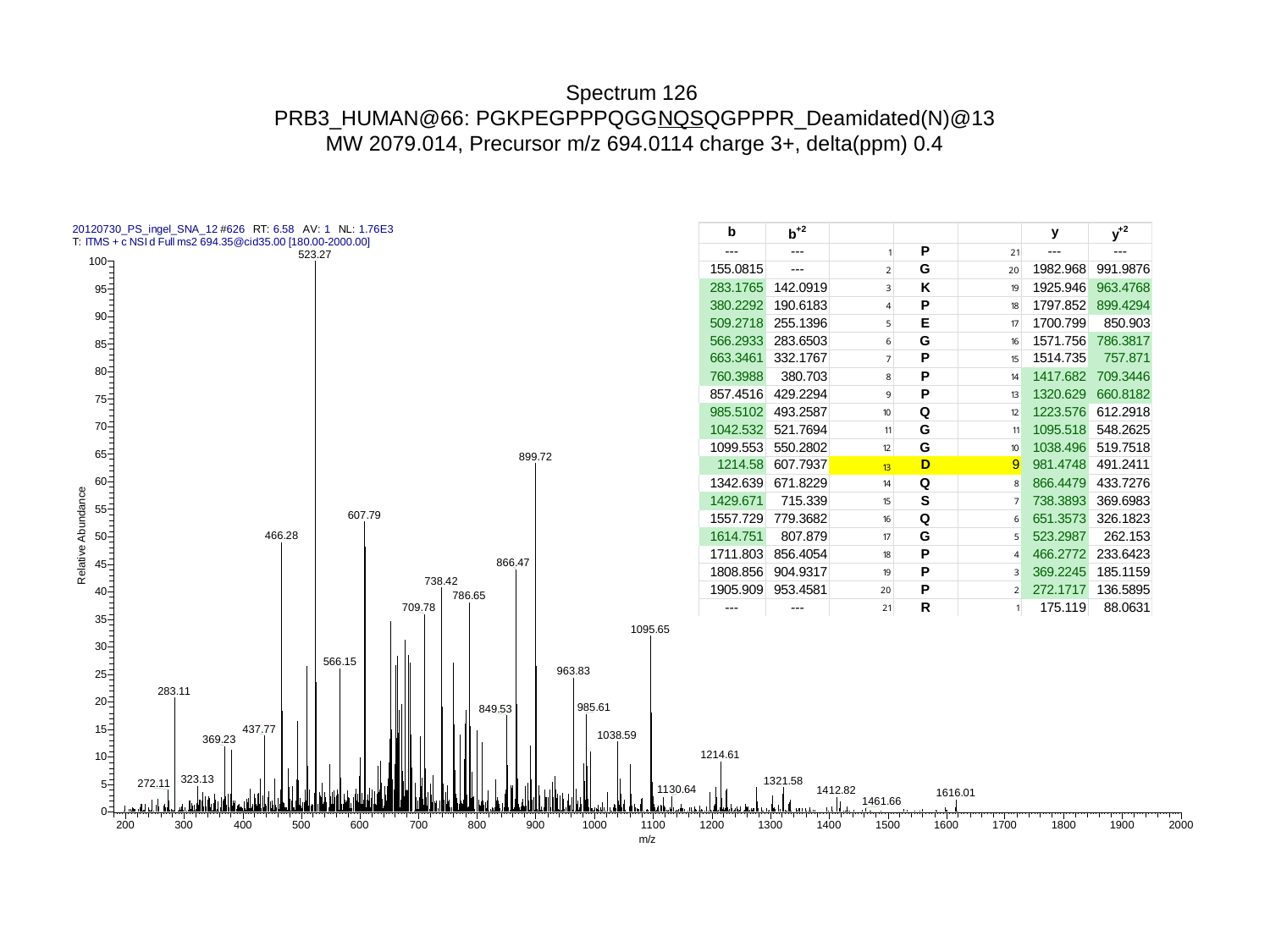

# Spectrum 126 PRB3_HUMAN@66: PGKPEGPPPQGGNQSQGPPPR_Deamidated(N)@13 MW 2079.014, Precursor m/z 694.0114 charge 3+, delta(ppm) 0.4

## Slide 151
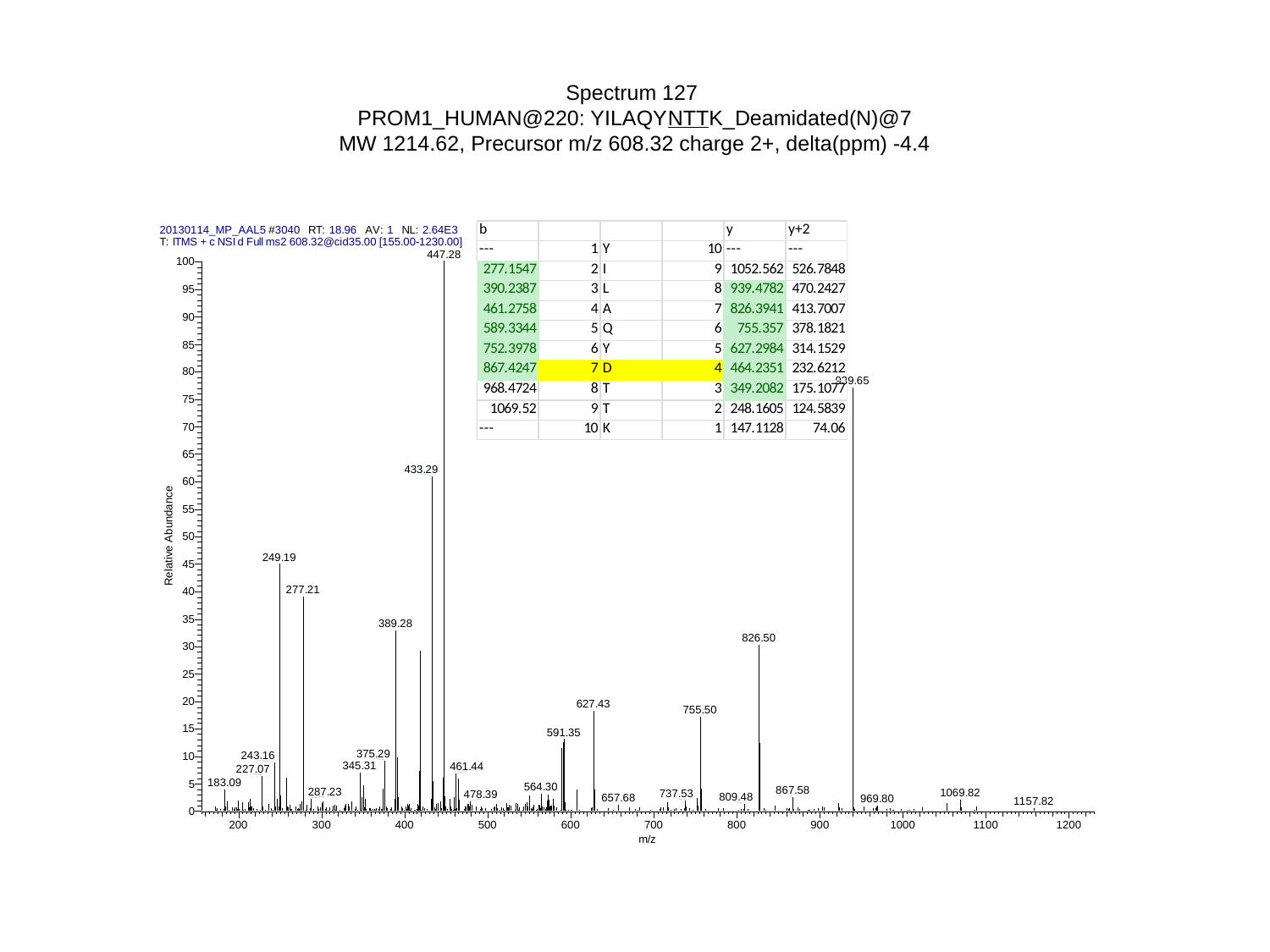

# Spectrum 127 PROM1_HUMAN@220: YILAQYNTTK_Deamidated(N)@7 MW 1214.62, Precursor m/z 608.32 charge 2+, delta(ppm) -4.4

## Slide 152
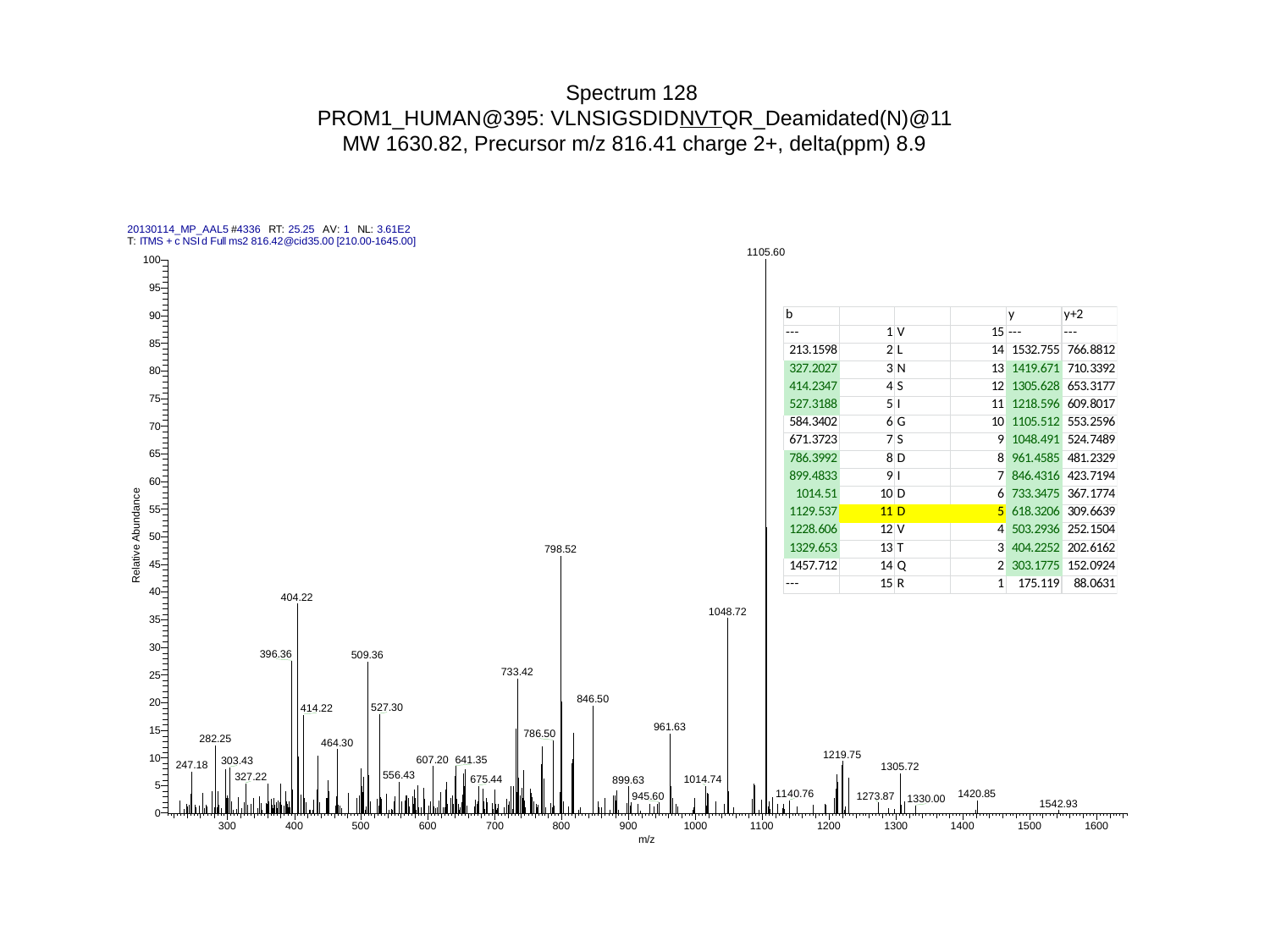

# Spectrum 128 PROM1_HUMAN@395: VLNSIGSDIDNVTQR_Deamidated(N)@11 MW 1630.82, Precursor m/z 816.41 charge 2+, delta(ppm) 8.9

## Slide 153
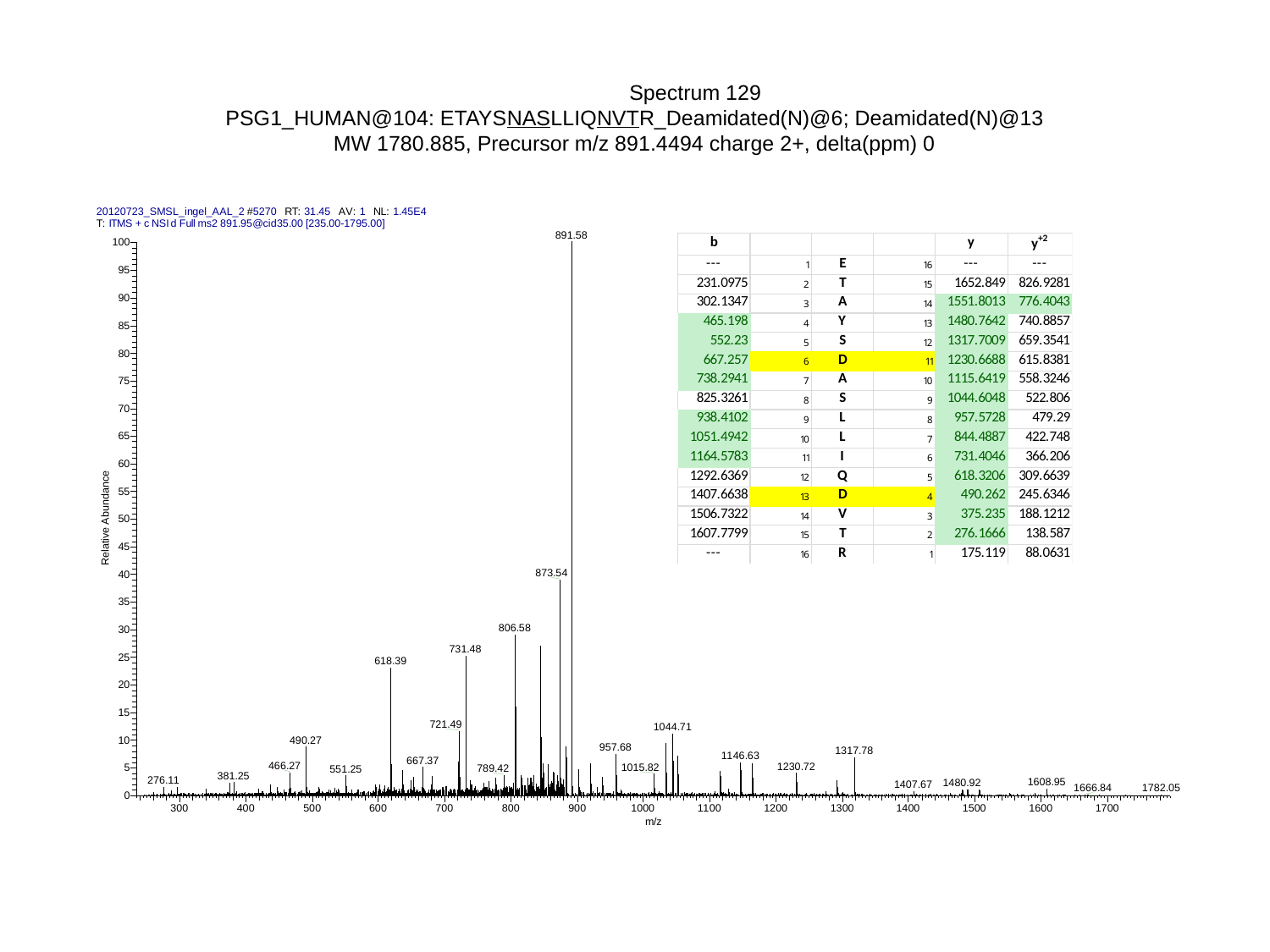

# Spectrum 129 PSG1_HUMAN@104: ETAYSNASLLIQNVTR_Deamidated(N)@6; Deamidated(N)@13 MW 1780.885, Precursor m/z 891.4494 charge 2+, delta(ppm) 0

## Slide 154
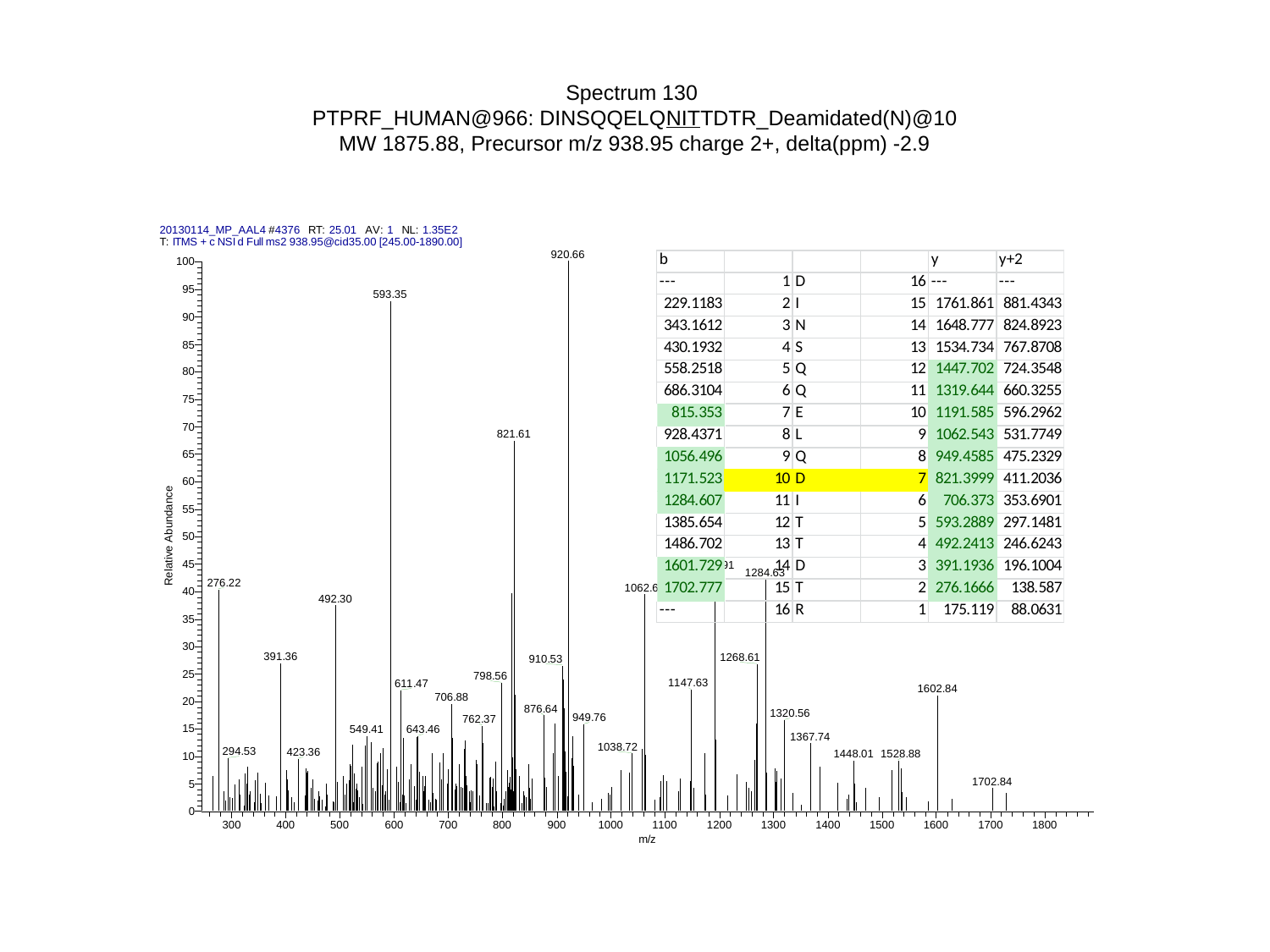

# Spectrum 130 PTPRF_HUMAN@966: DINSQQELQNITTDTR_Deamidated(N)@10 MW 1875.88, Precursor m/z 938.95 charge 2+, delta(ppm) -2.9

## Slide 155
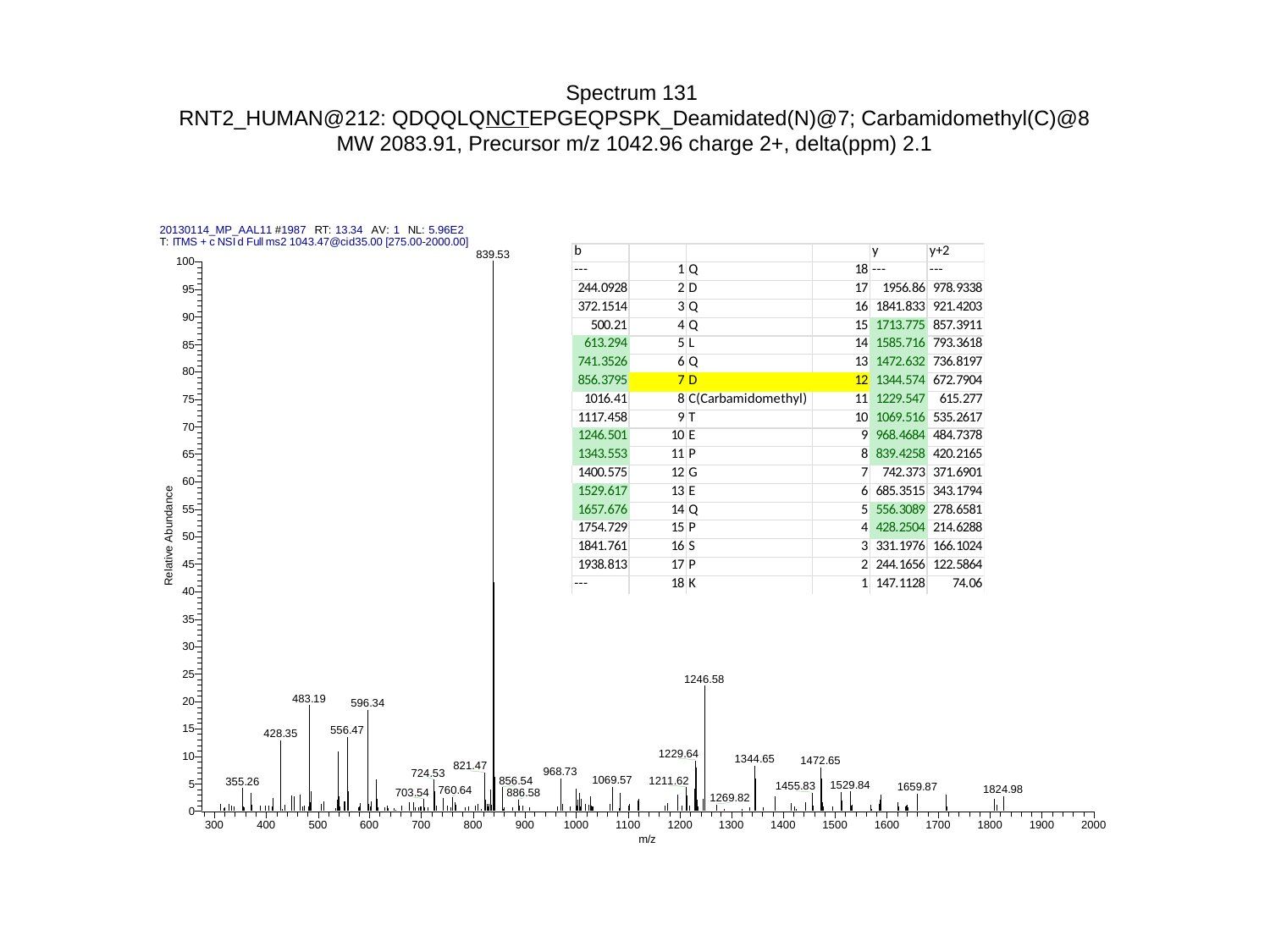

# Spectrum 131 RNT2_HUMAN@212: QDQQLQNCTEPGEQPSPK_Deamidated(N)@7; Carbamidomethyl(C)@8 MW 2083.91, Precursor m/z 1042.96 charge 2+, delta(ppm) 2.1

## Slide 156
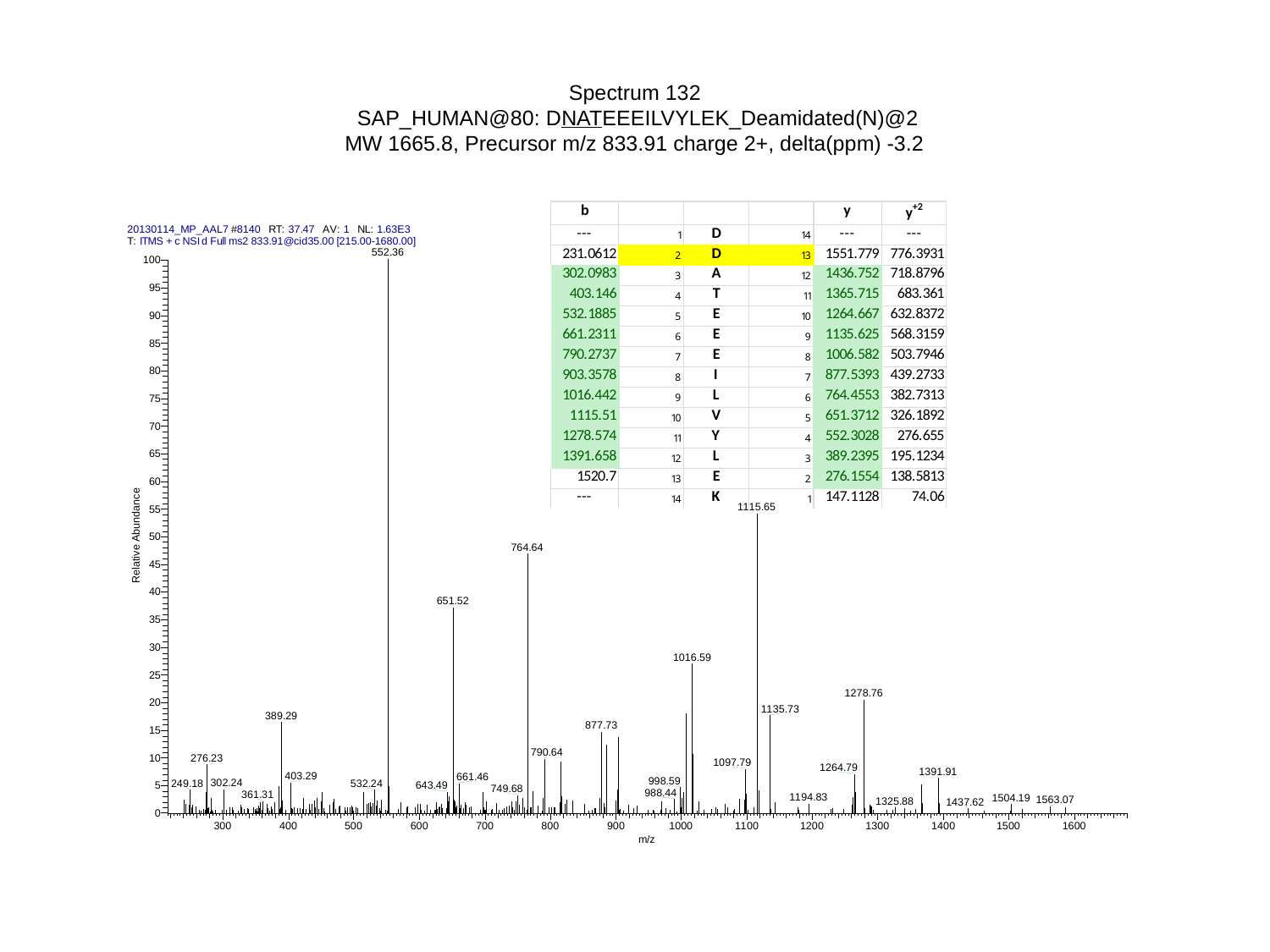

# Spectrum 132 SAP_HUMAN@80: DNATEEEILVYLEK_Deamidated(N)@2 MW 1665.8, Precursor m/z 833.91 charge 2+, delta(ppm) -3.2

## Slide 157
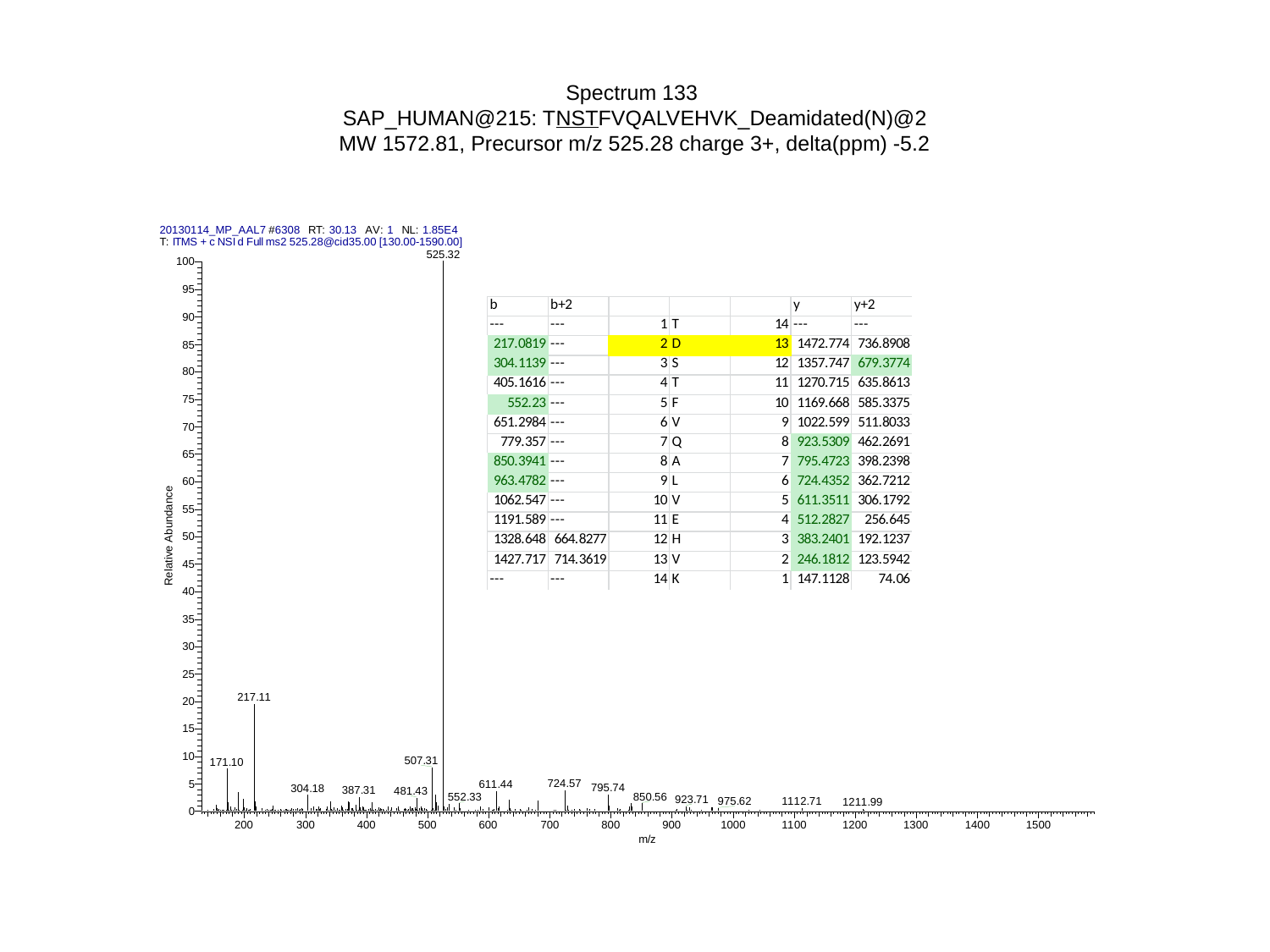

# Spectrum 133 SAP_HUMAN@215: TNSTFVQALVEHVK_Deamidated(N)@2 MW 1572.81, Precursor m/z 525.28 charge 3+, delta(ppm) -5.2

## Slide 158
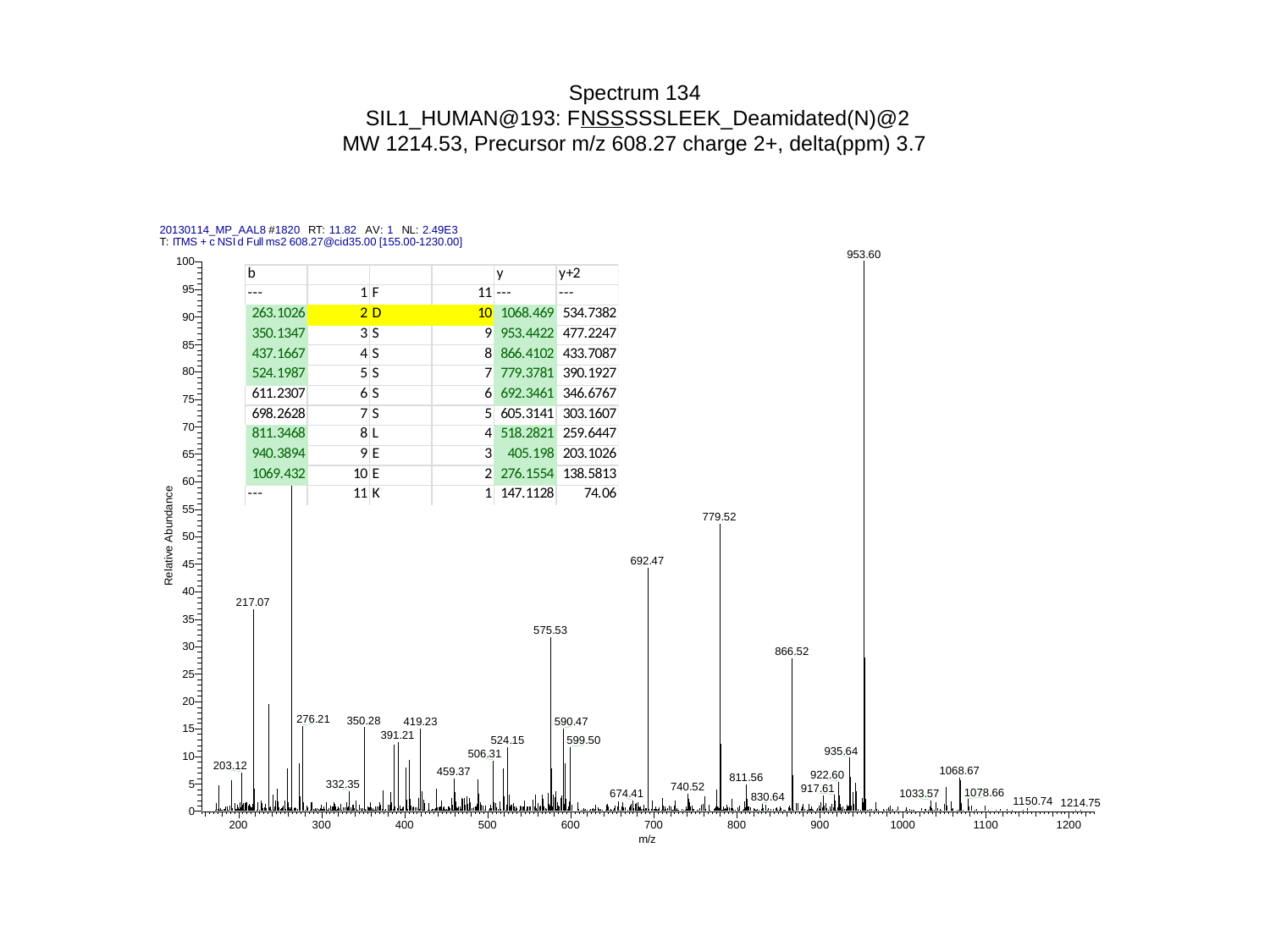

# Spectrum 134 SIL1_HUMAN@193: FNSSSSSLEEK_Deamidated(N)@2 MW 1214.53, Precursor m/z 608.27 charge 2+, delta(ppm) 3.7

## Slide 159
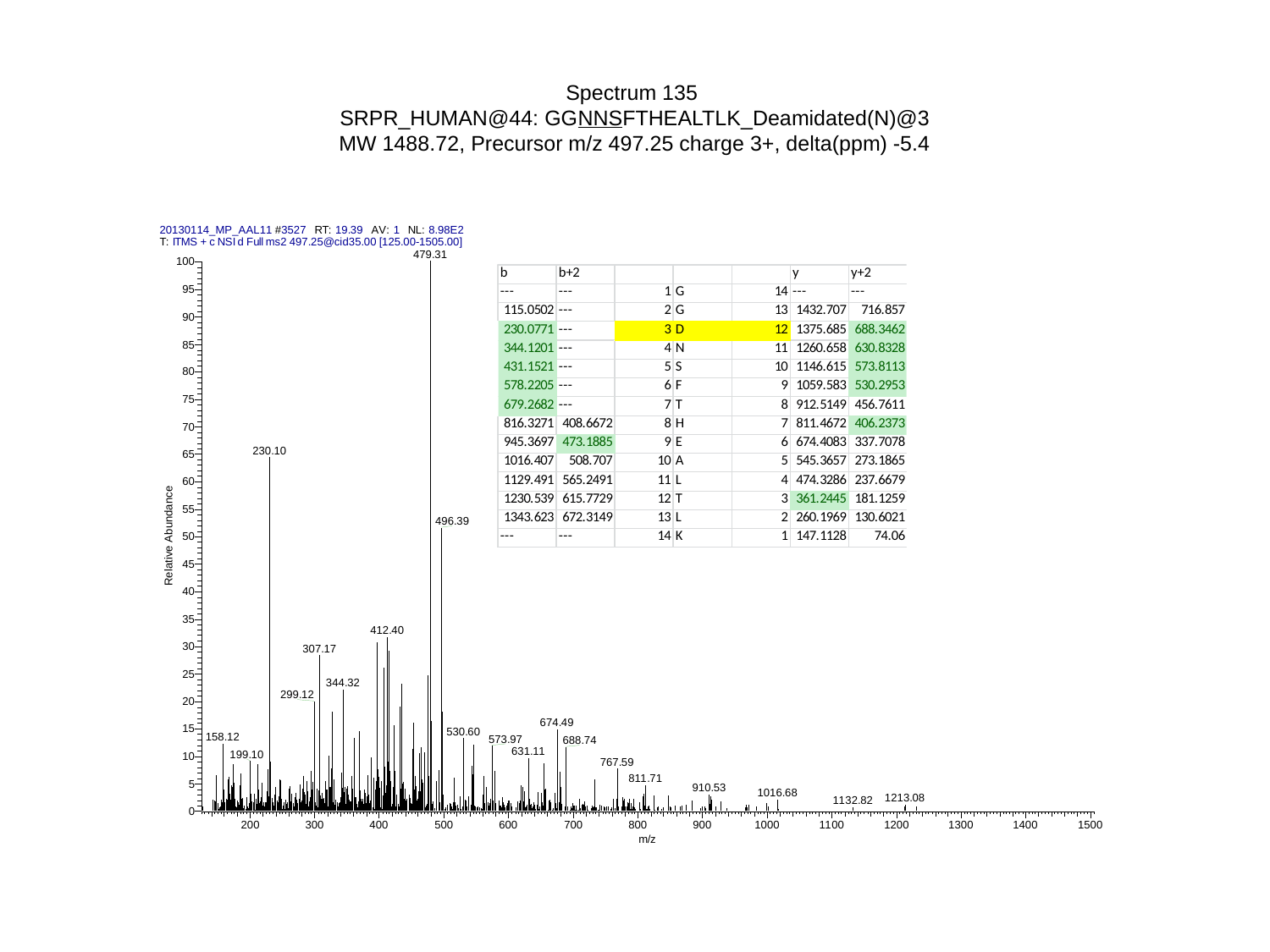

# Spectrum 135 SRPR_HUMAN@44: GGNNSFTHEALTLK_Deamidated(N)@3 MW 1488.72, Precursor m/z 497.25 charge 3+, delta(ppm) -5.4

## Slide 160
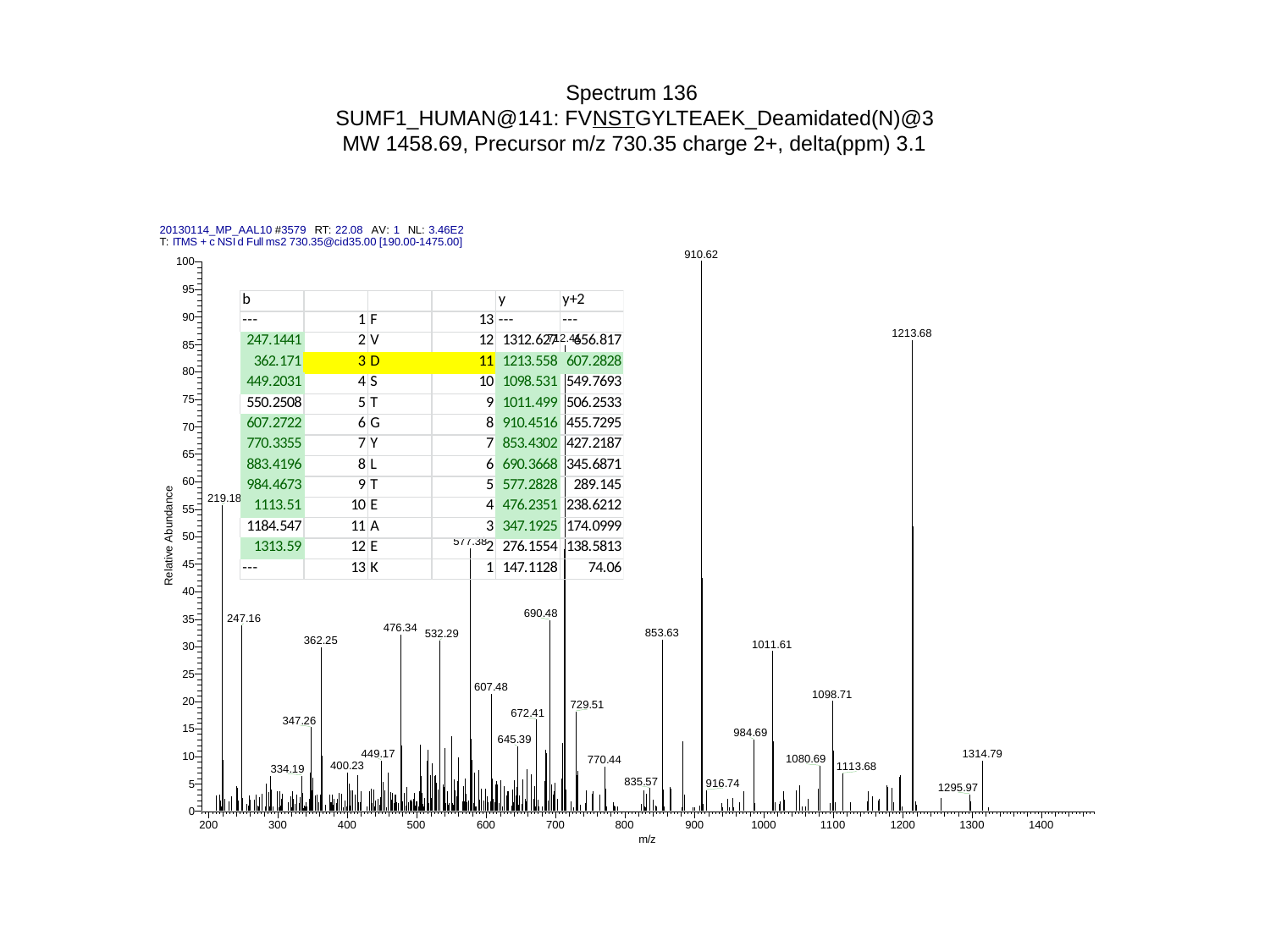

# Spectrum 136 SUMF1_HUMAN@141: FVNSTGYLTEAEK_Deamidated(N)@3 MW 1458.69, Precursor m/z 730.35 charge 2+, delta(ppm) 3.1

## Slide 161
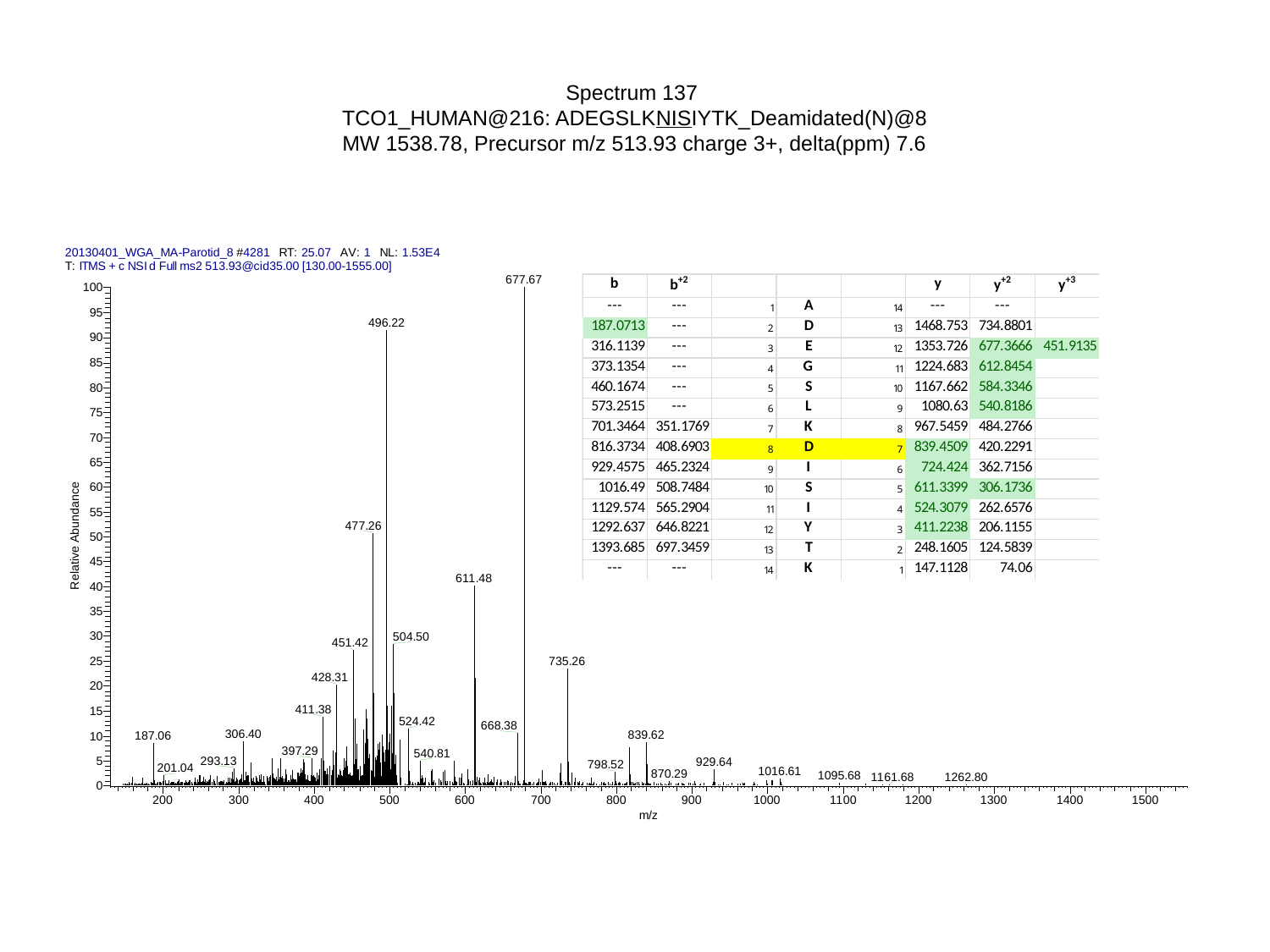

# Spectrum 137 TCO1_HUMAN@216: ADEGSLKNISIYTK_Deamidated(N)@8 MW 1538.78, Precursor m/z 513.93 charge 3+, delta(ppm) 7.6

## Slide 162
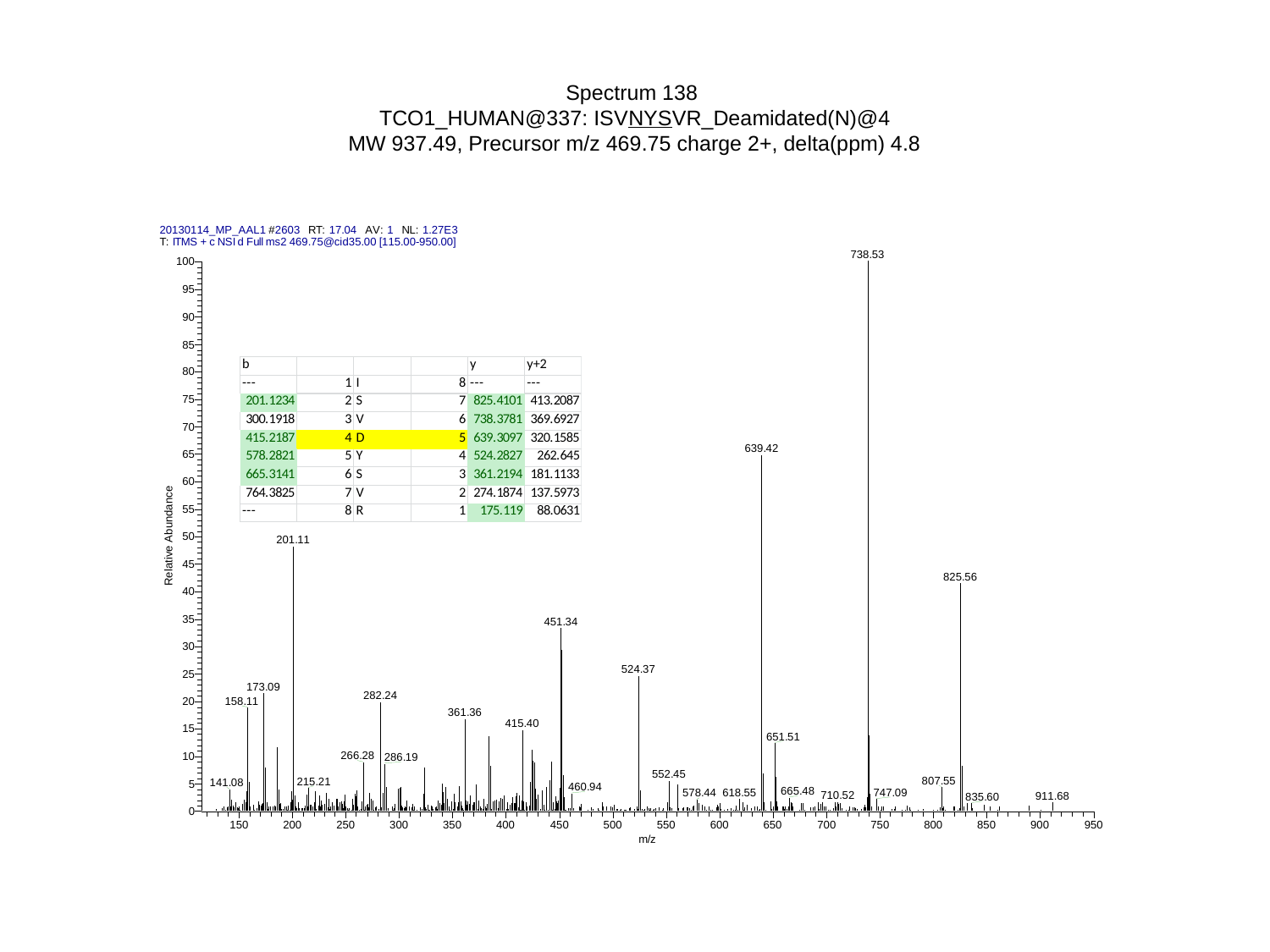

# Spectrum 138 TCO1_HUMAN@337: ISVNYSVR_Deamidated(N)@4 MW 937.49, Precursor m/z 469.75 charge 2+, delta(ppm) 4.8

## Slide 163
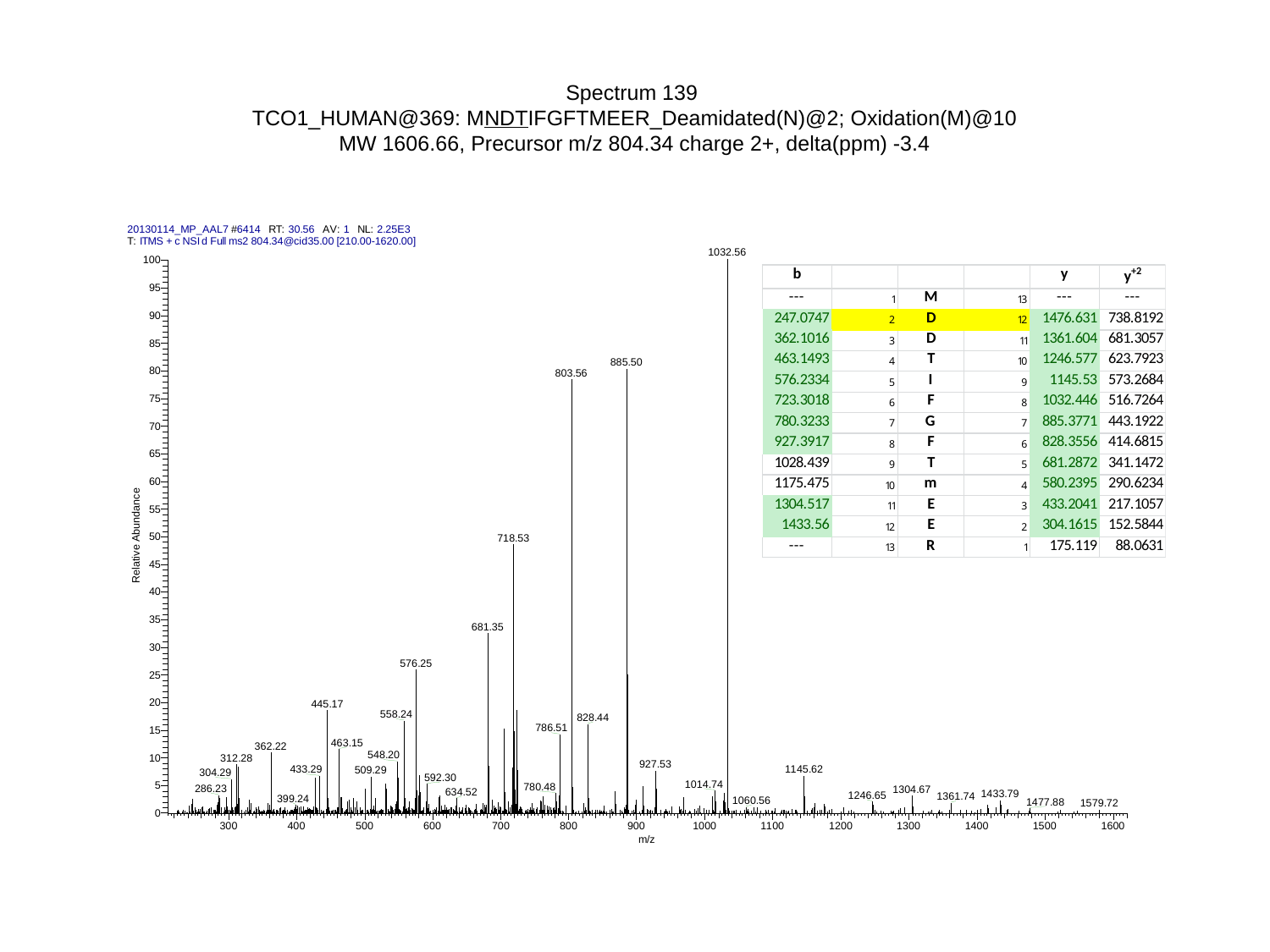

# Spectrum 139 TCO1_HUMAN@369: MNDTIFGFTMEER_Deamidated(N)@2; Oxidation(M)@10 MW 1606.66, Precursor m/z 804.34 charge 2+, delta(ppm) -3.4

## Slide 164
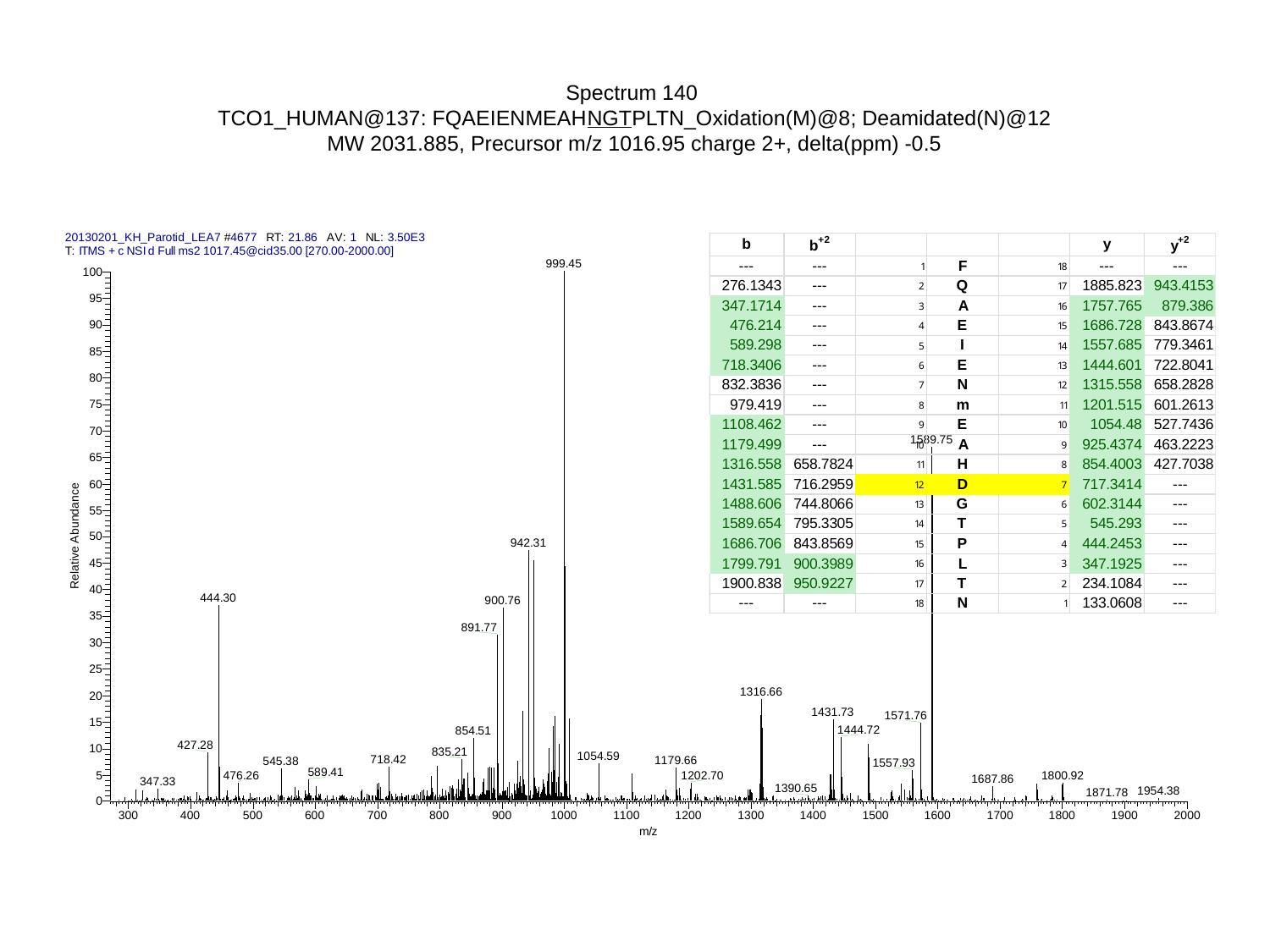

# Spectrum 140 TCO1_HUMAN@137: FQAEIENMEAHNGTPLTN_Oxidation(M)@8; Deamidated(N)@12 MW 2031.885, Precursor m/z 1016.95 charge 2+, delta(ppm) -0.5

## Slide 165
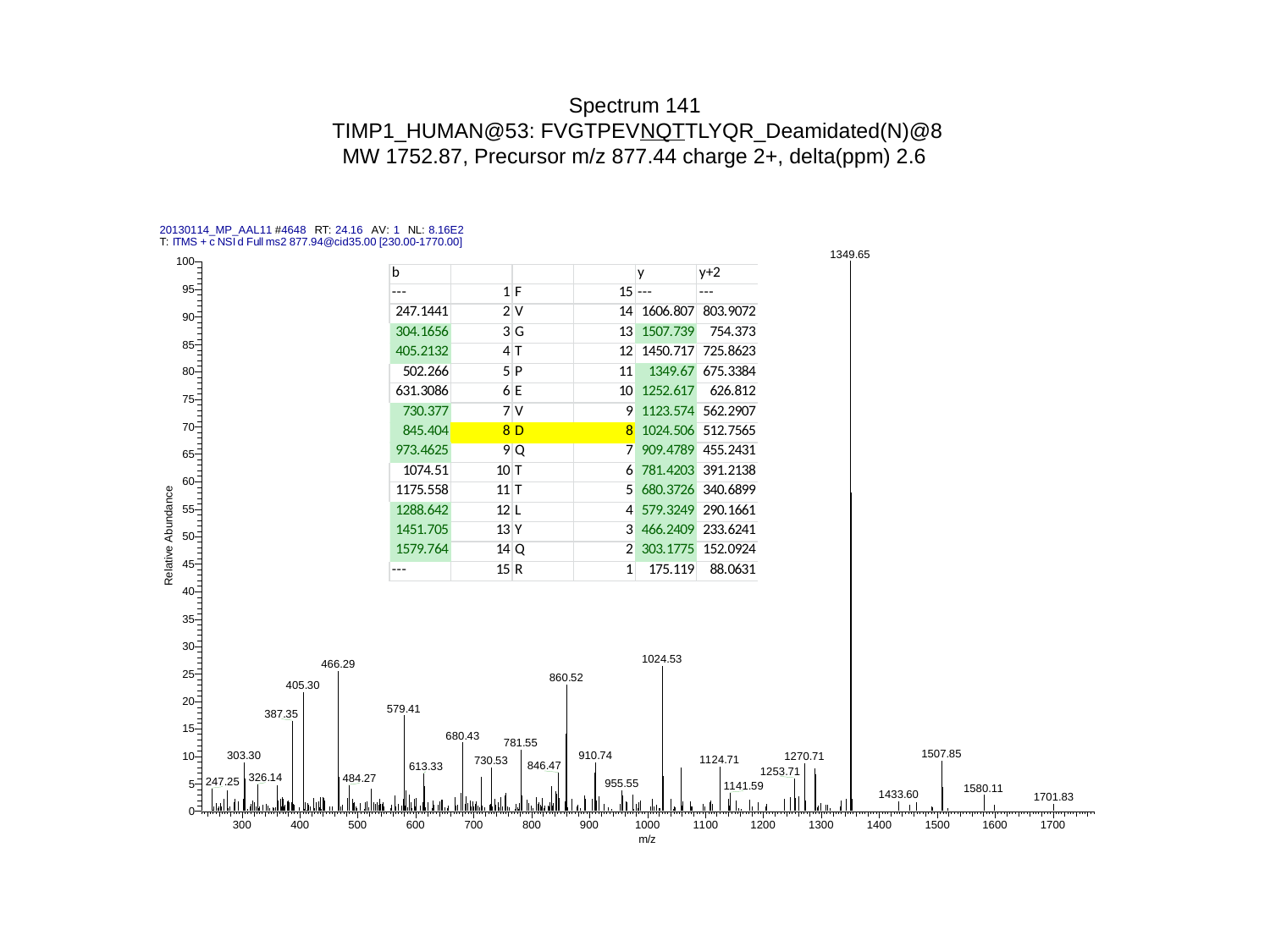

# Spectrum 141 TIMP1_HUMAN@53: FVGTPEVNQTTLYQR_Deamidated(N)@8 MW 1752.87, Precursor m/z 877.44 charge 2+, delta(ppm) 2.6

## Slide 166
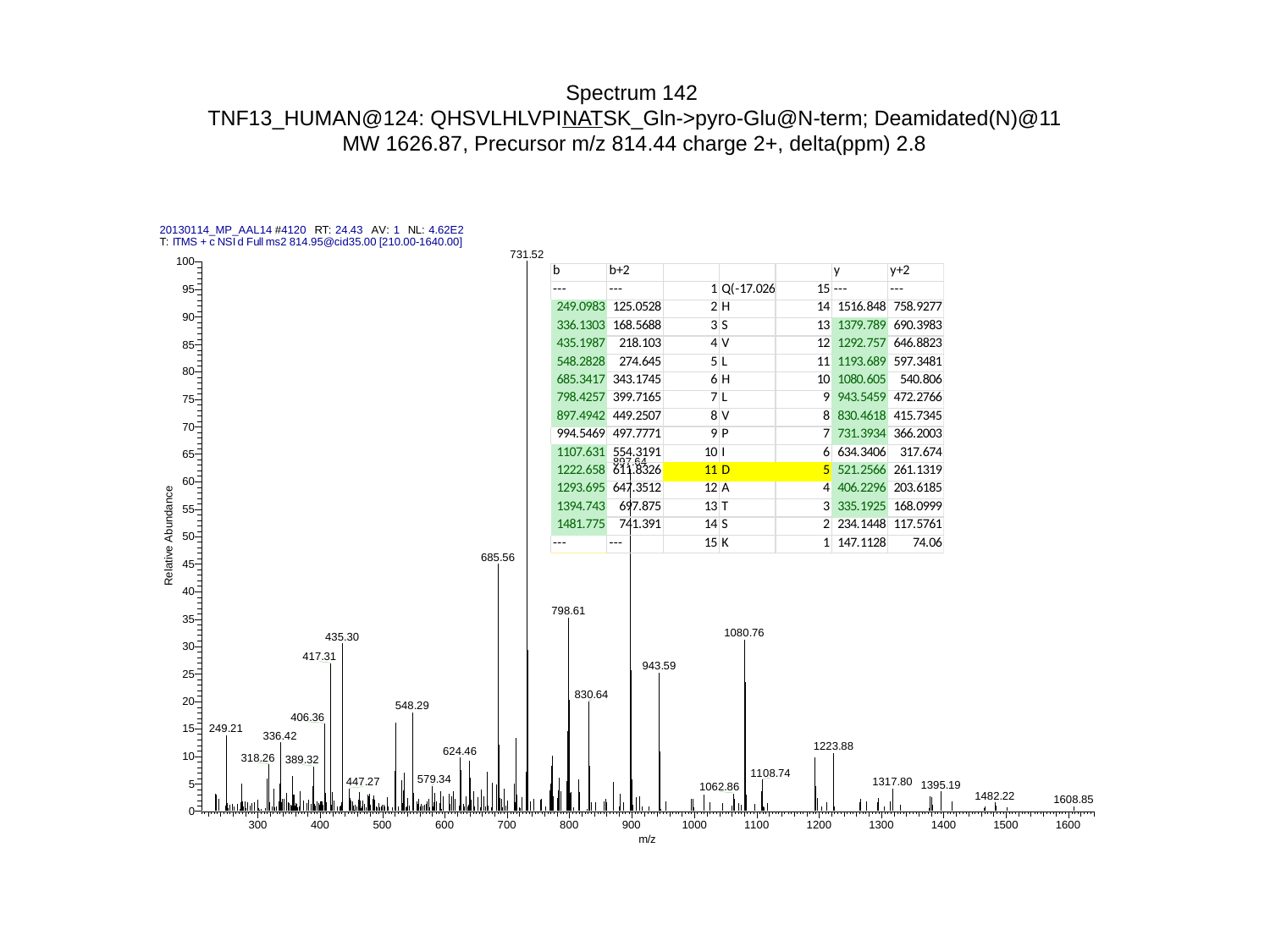

# Spectrum 142 TNF13_HUMAN@124: QHSVLHLVPINATSK_Gln->pyro-Glu@N-term; Deamidated(N)@11 MW 1626.87, Precursor m/z 814.44 charge 2+, delta(ppm) 2.8

## Slide 167
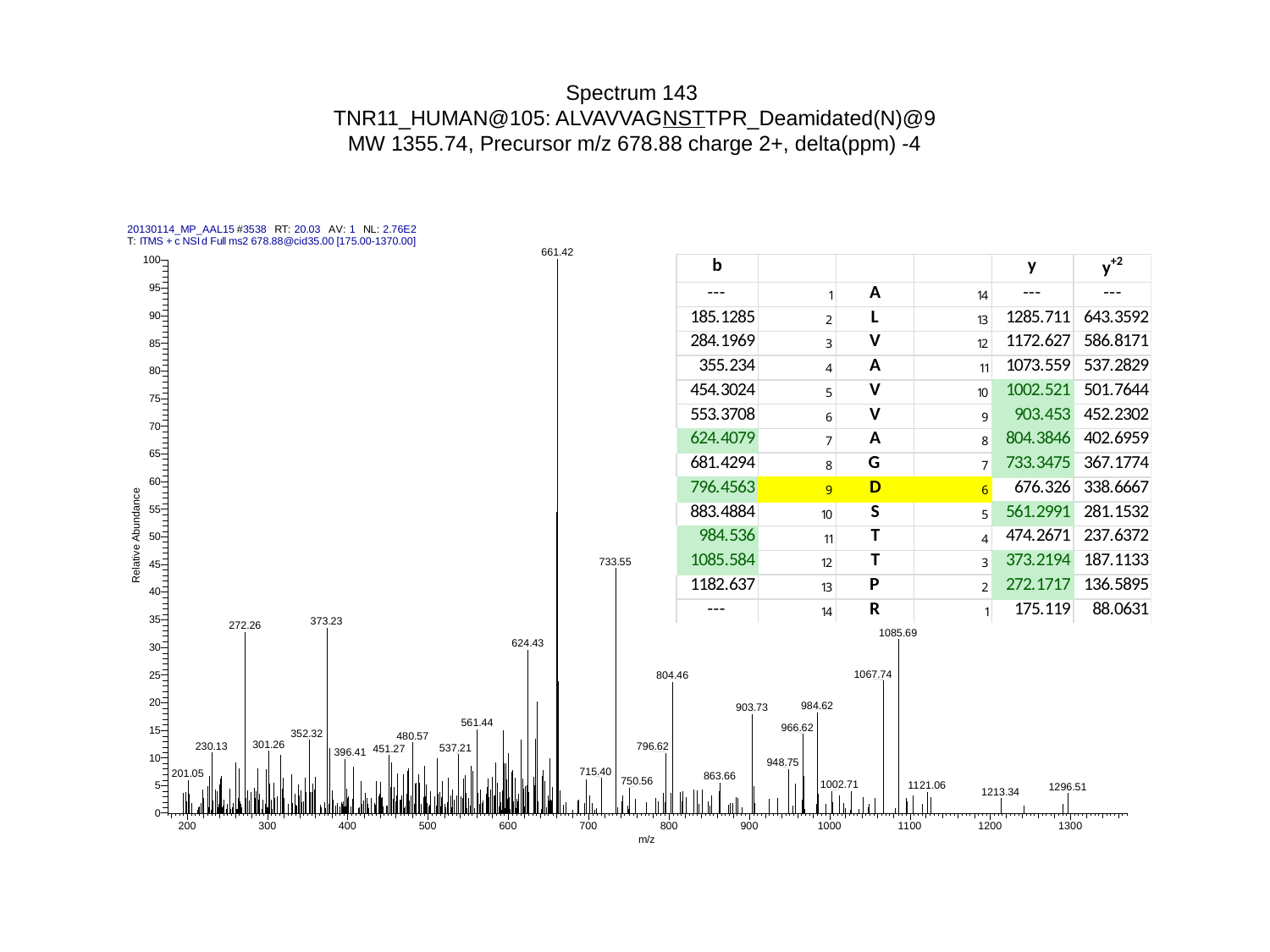

# Spectrum 143 TNR11_HUMAN@105: ALVAVVAGNSTTPR_Deamidated(N)@9 MW 1355.74, Precursor m/z 678.88 charge 2+, delta(ppm) -4

## Slide 168
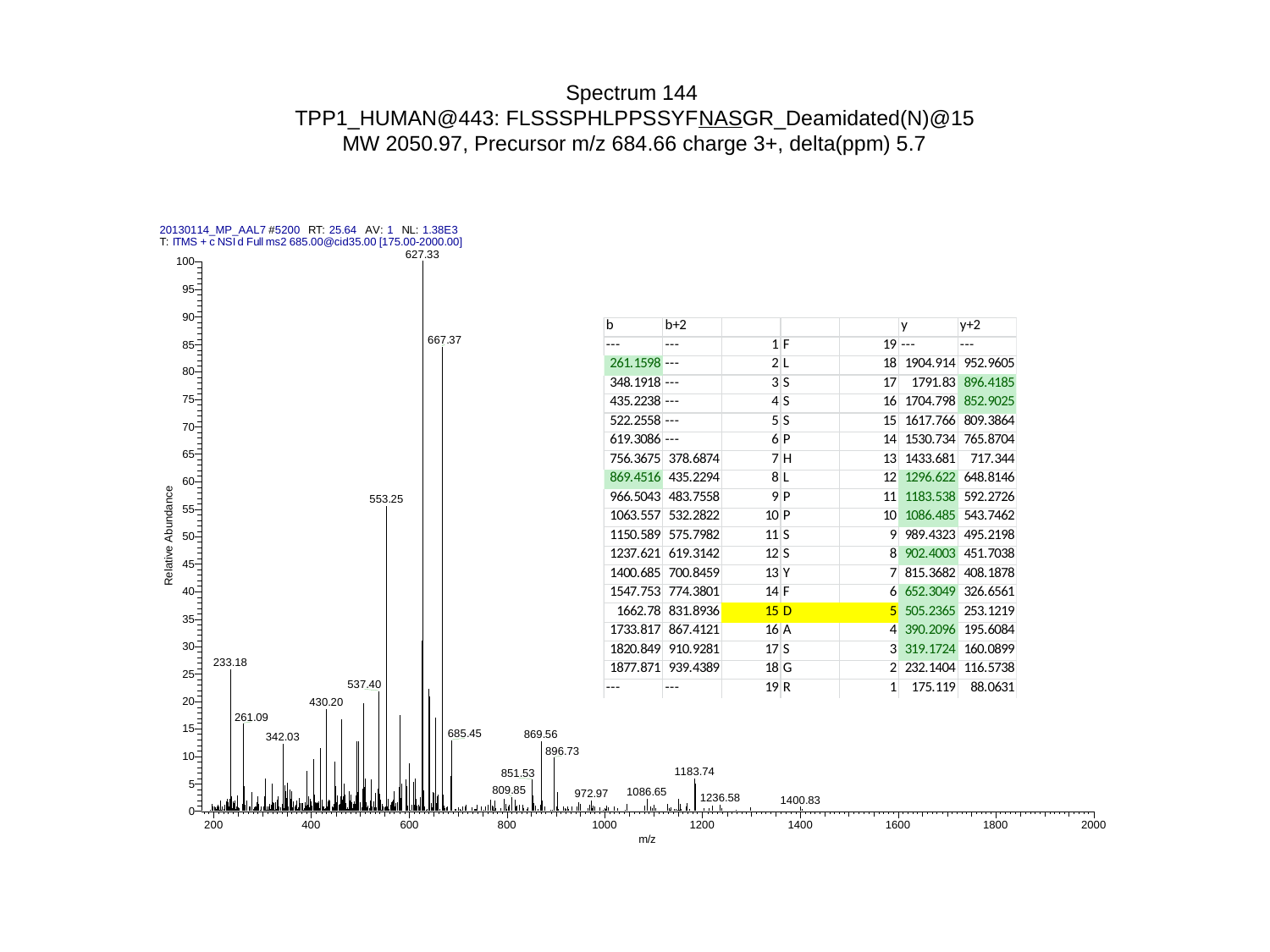

# Spectrum 144 TPP1_HUMAN@443: FLSSSPHLPPSSYFNASGR_Deamidated(N)@15 MW 2050.97, Precursor m/z 684.66 charge 3+, delta(ppm) 5.7

## Slide 169
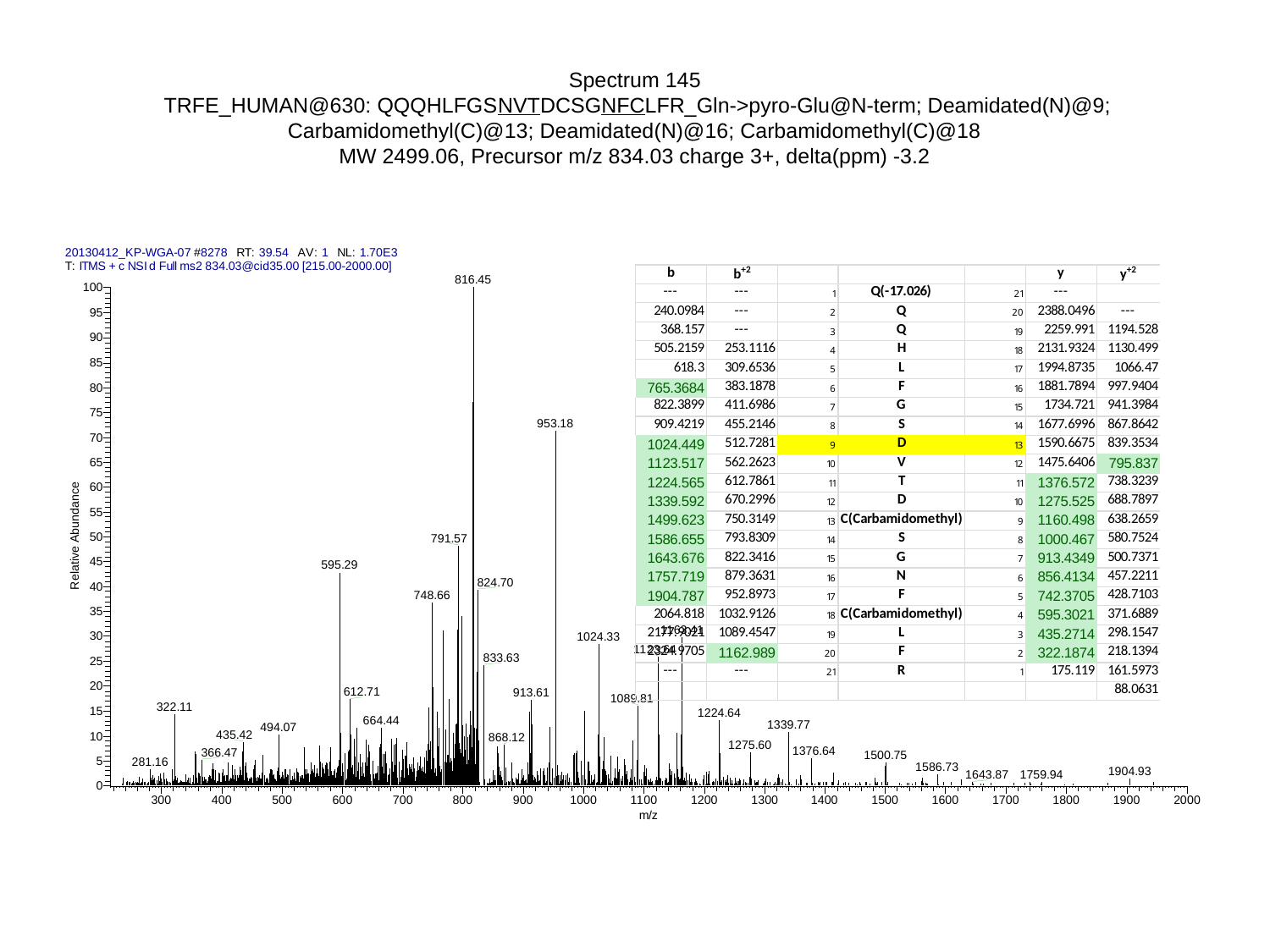

# Spectrum 145 TRFE_HUMAN@630: QQQHLFGSNVTDCSGNFCLFR_Gln->pyro-Glu@N-term; Deamidated(N)@9; Carbamidomethyl(C)@13; Deamidated(N)@16; Carbamidomethyl(C)@18 MW 2499.06, Precursor m/z 834.03 charge 3+, delta(ppm) -3.2

## Slide 170
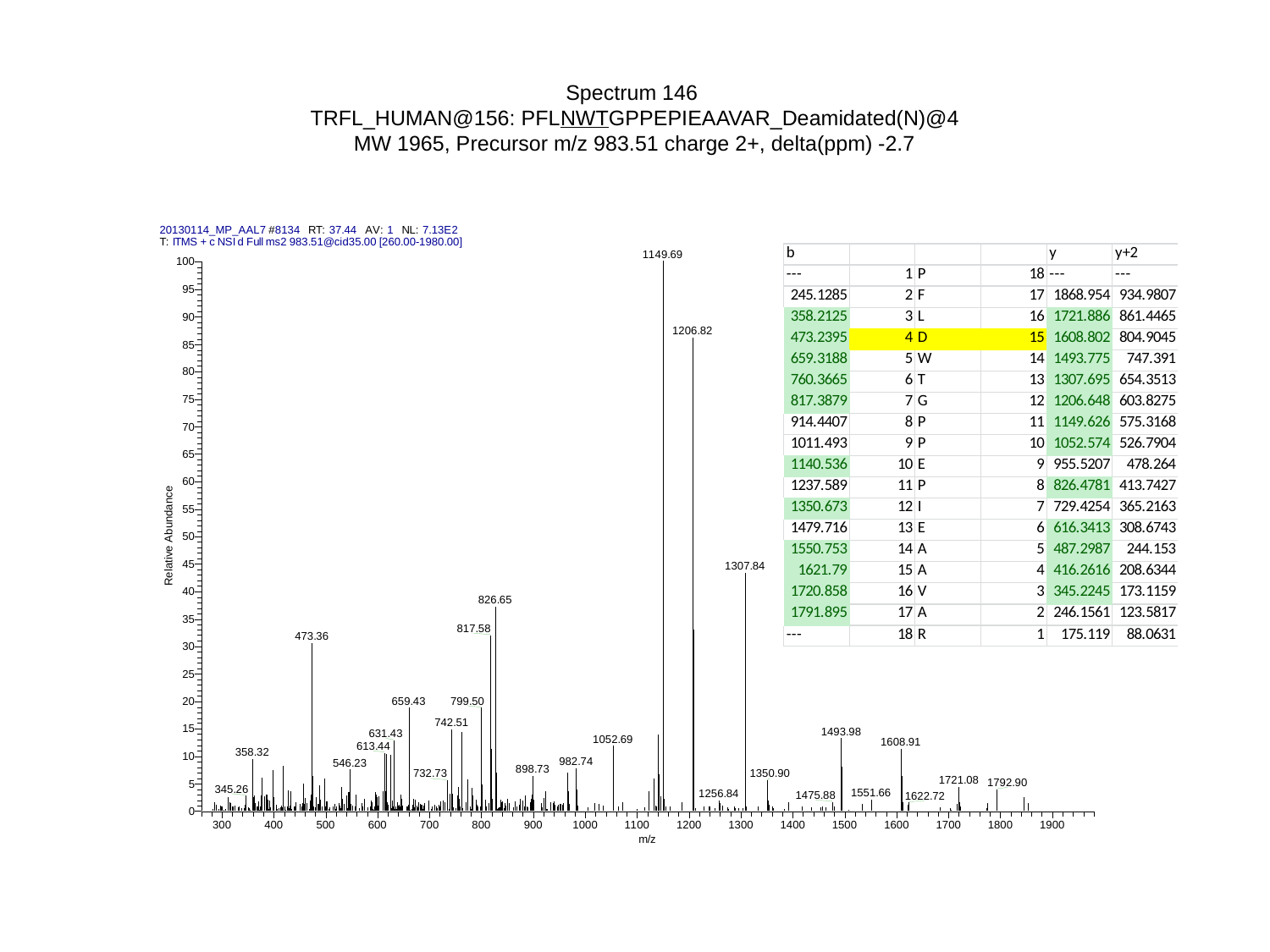

# Spectrum 146 TRFL_HUMAN@156: PFLNWTGPPEPIEAAVAR_Deamidated(N)@4 MW 1965, Precursor m/z 983.51 charge 2+, delta(ppm) -2.7

## Slide 171
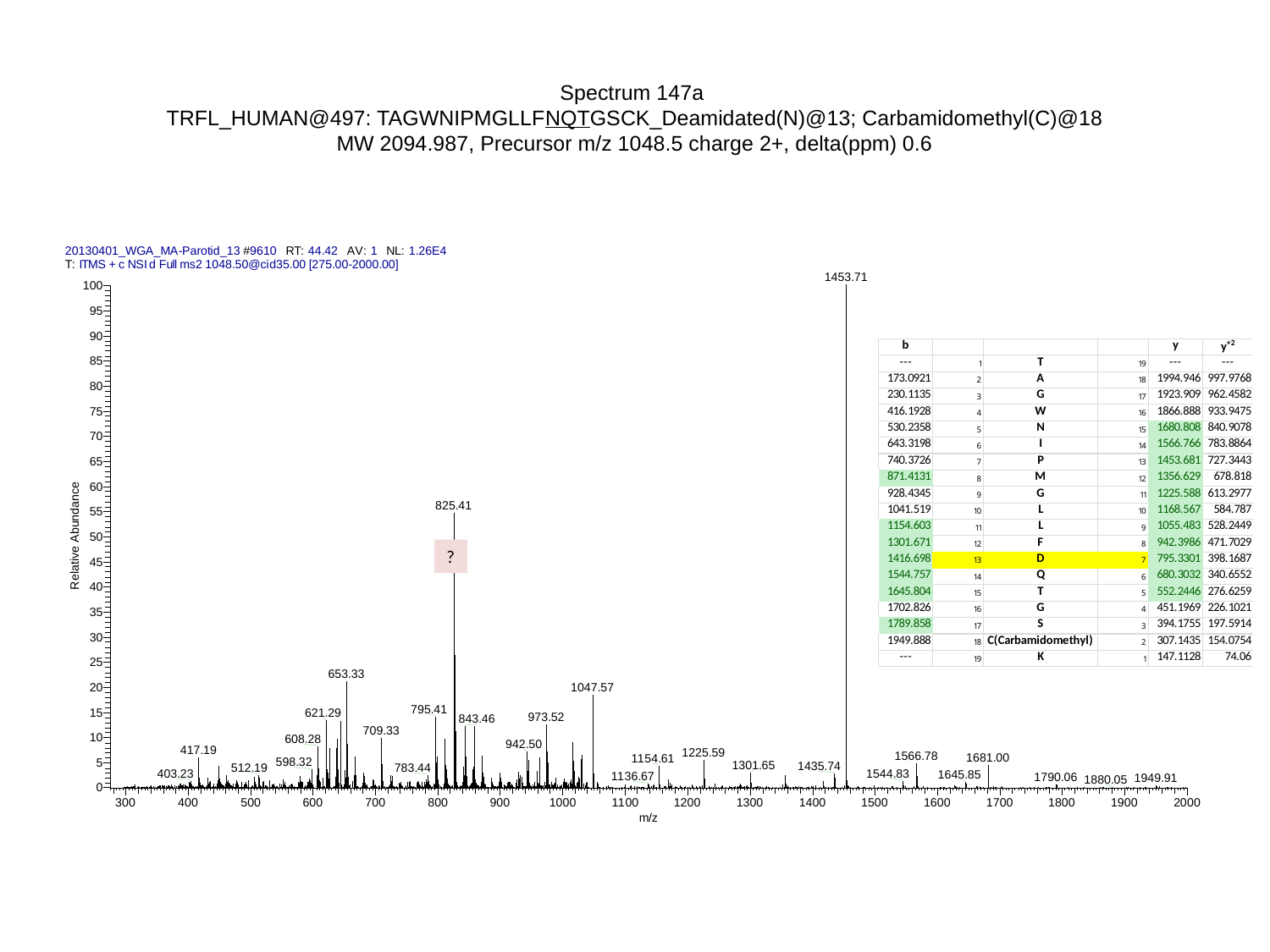

# Spectrum 147a TRFL_HUMAN@497: TAGWNIPMGLLFNQTGSCK_Deamidated(N)@13; Carbamidomethyl(C)@18 MW 2094.987, Precursor m/z 1048.5 charge 2+, delta(ppm) 0.6
?

## Slide 172
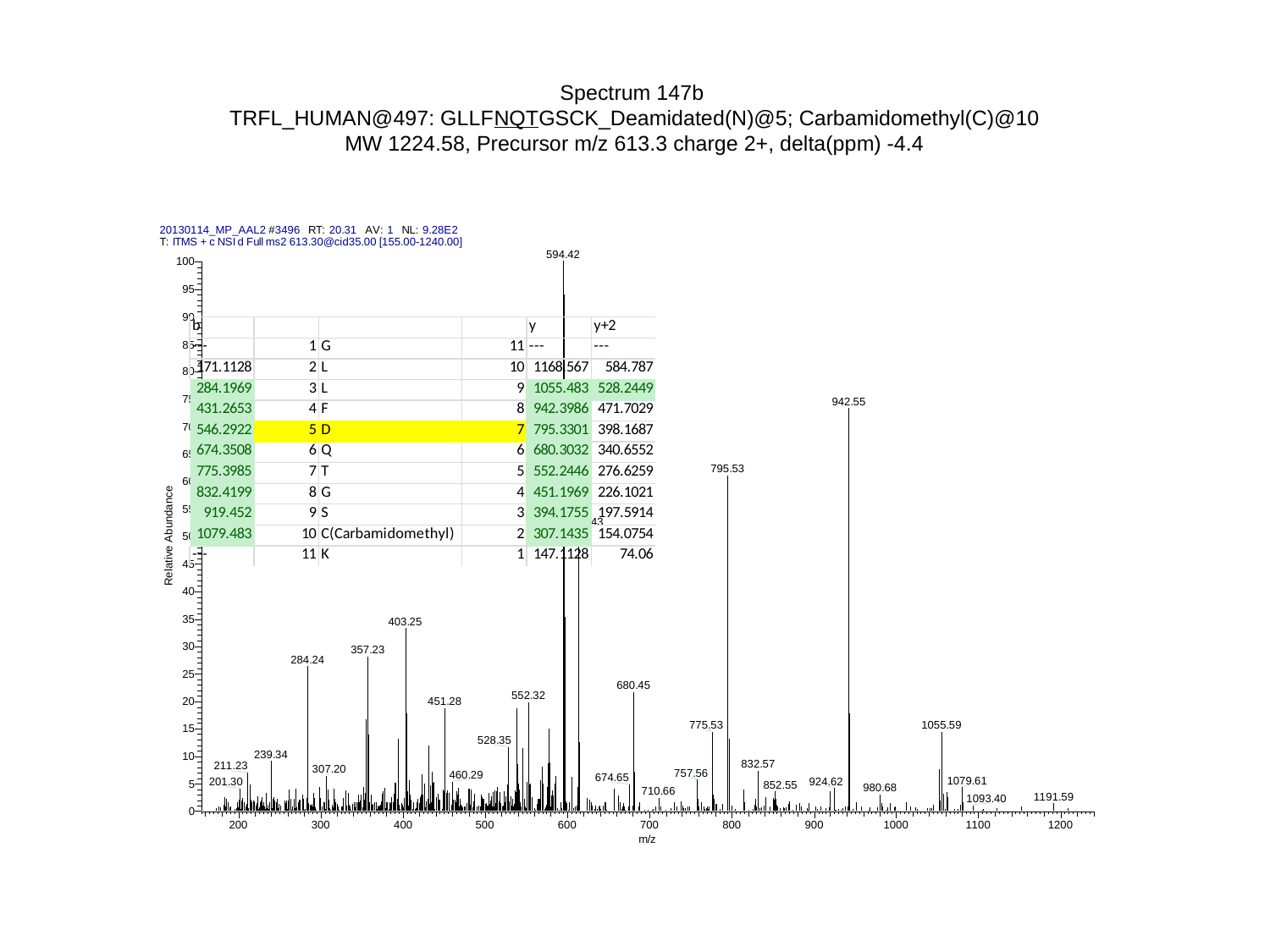

# Spectrum 147b TRFL_HUMAN@497: GLLFNQTGSCK_Deamidated(N)@5; Carbamidomethyl(C)@10 MW 1224.58, Precursor m/z 613.3 charge 2+, delta(ppm) -4.4

## Slide 173
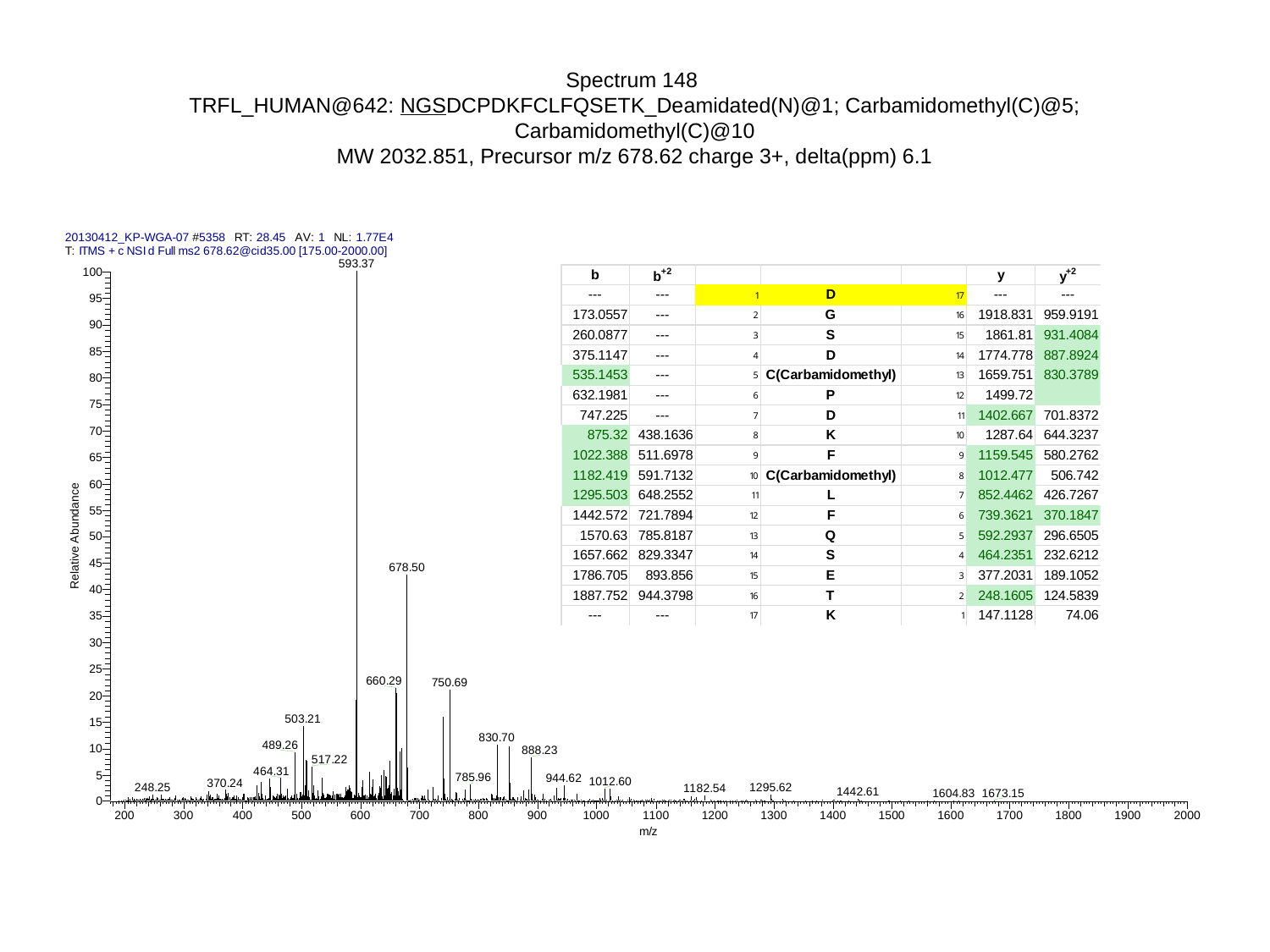

# Spectrum 148 TRFL_HUMAN@642: NGSDCPDKFCLFQSETK_Deamidated(N)@1; Carbamidomethyl(C)@5; Carbamidomethyl(C)@10 MW 2032.851, Precursor m/z 678.62 charge 3+, delta(ppm) 6.1

## Slide 174
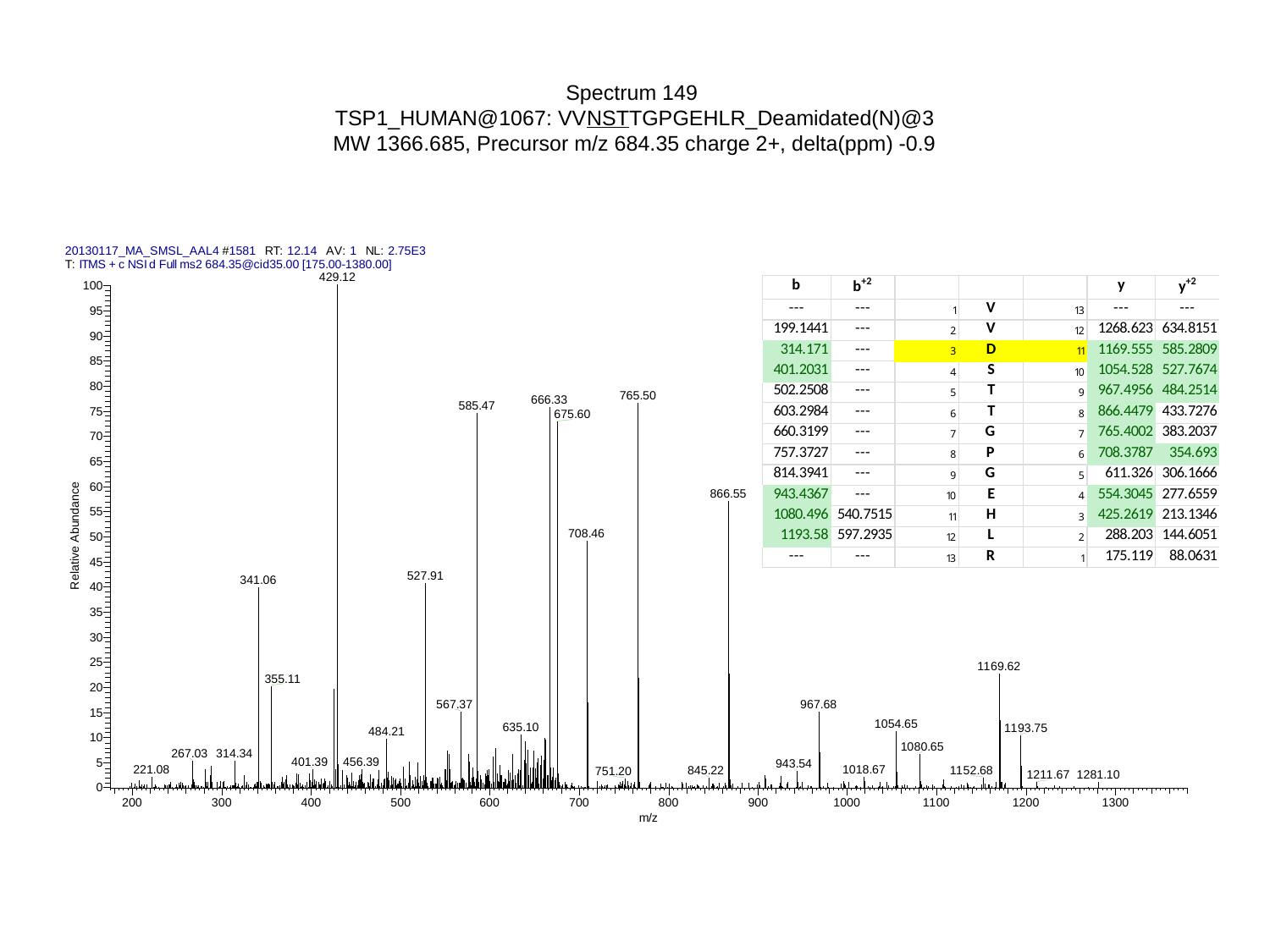

# Spectrum 149 TSP1_HUMAN@1067: VVNSTTGPGEHLR_Deamidated(N)@3 MW 1366.685, Precursor m/z 684.35 charge 2+, delta(ppm) -0.9

## Slide 175
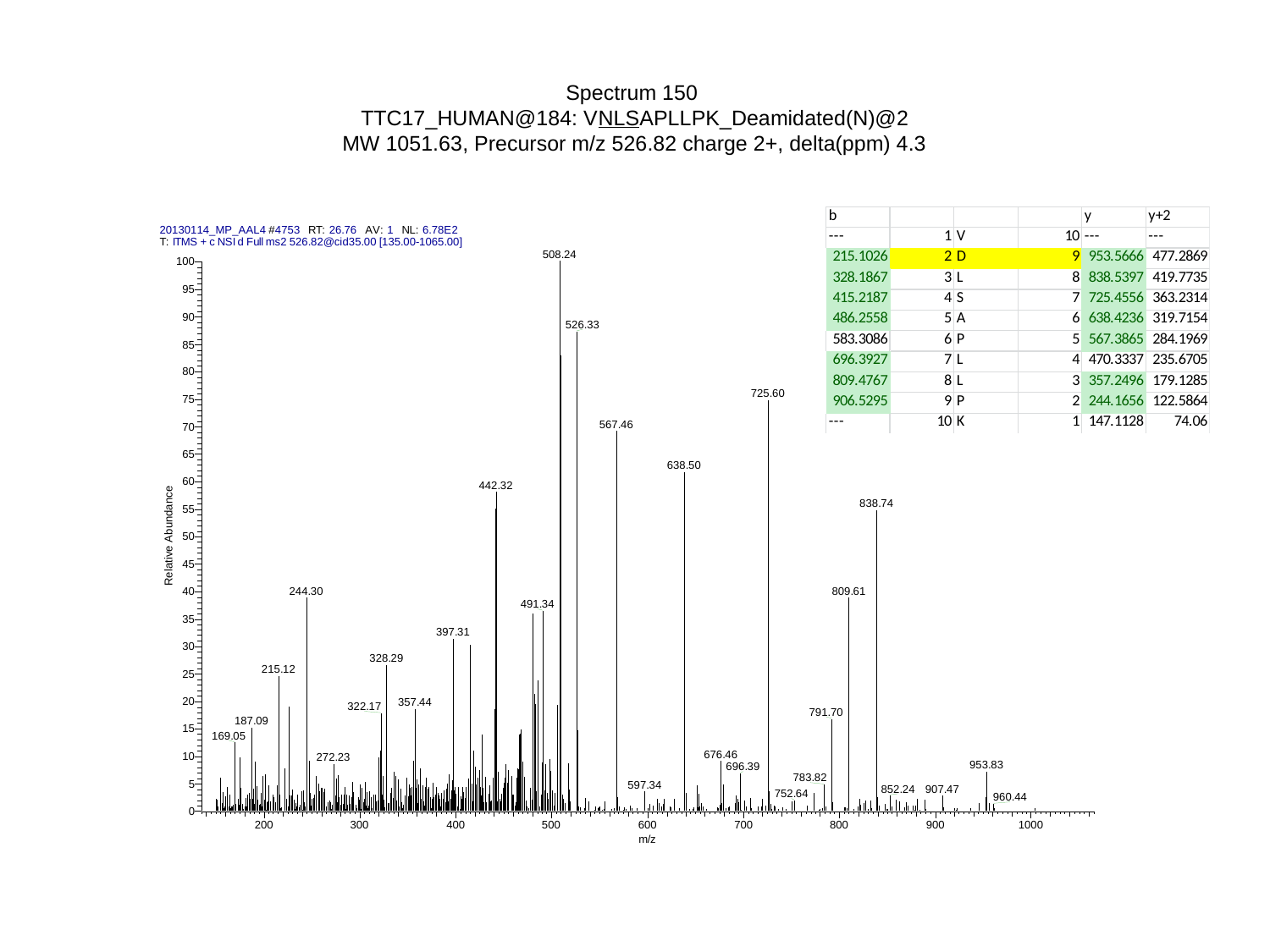

# Spectrum 150 TTC17_HUMAN@184: VNLSAPLLPK_Deamidated(N)@2 MW 1051.63, Precursor m/z 526.82 charge 2+, delta(ppm) 4.3

## Slide 176
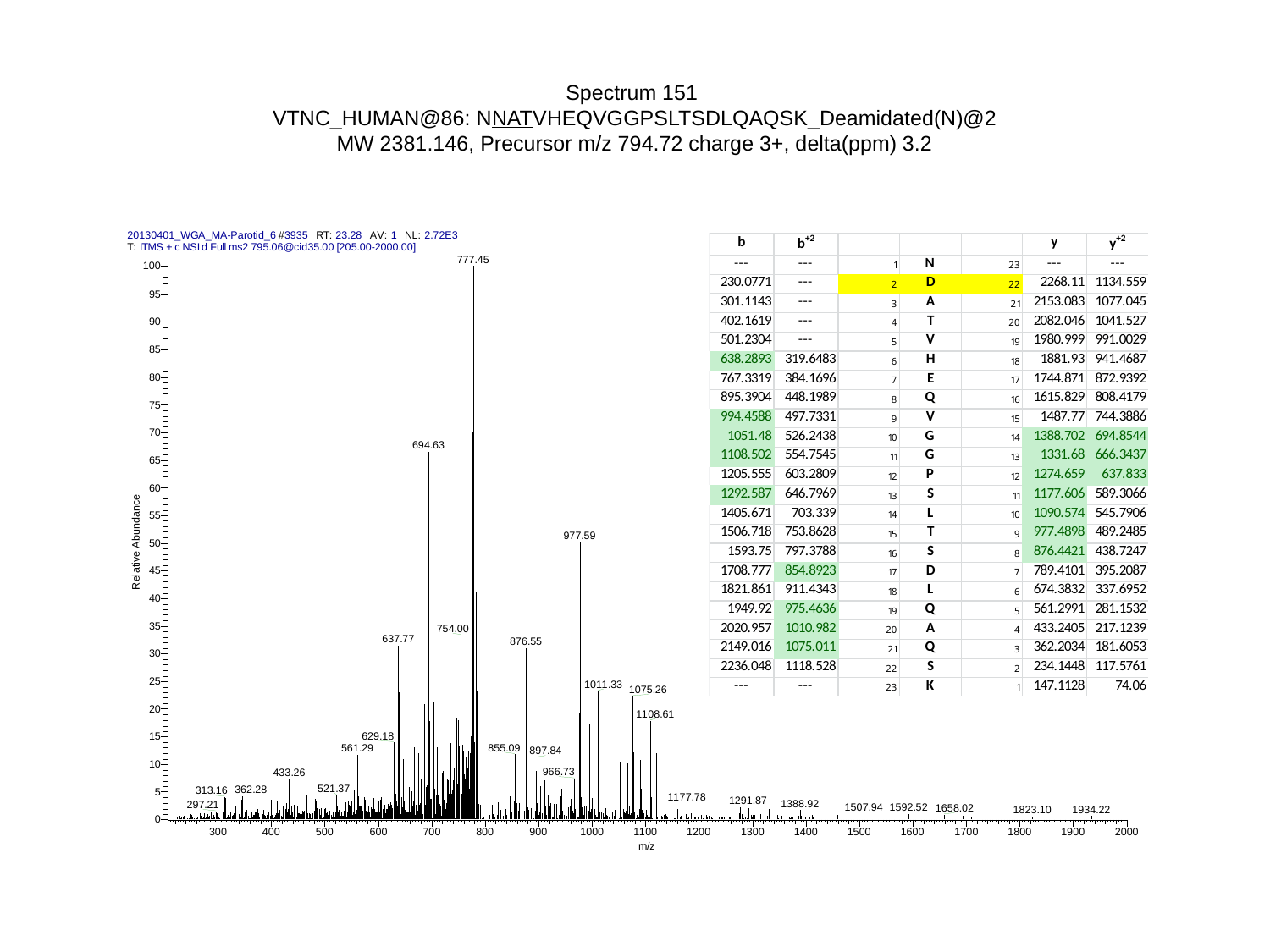

# Spectrum 151 VTNC_HUMAN@86: NNATVHEQVGGPSLTSDLQAQSK_Deamidated(N)@2 MW 2381.146, Precursor m/z 794.72 charge 3+, delta(ppm) 3.2

## Slide 177
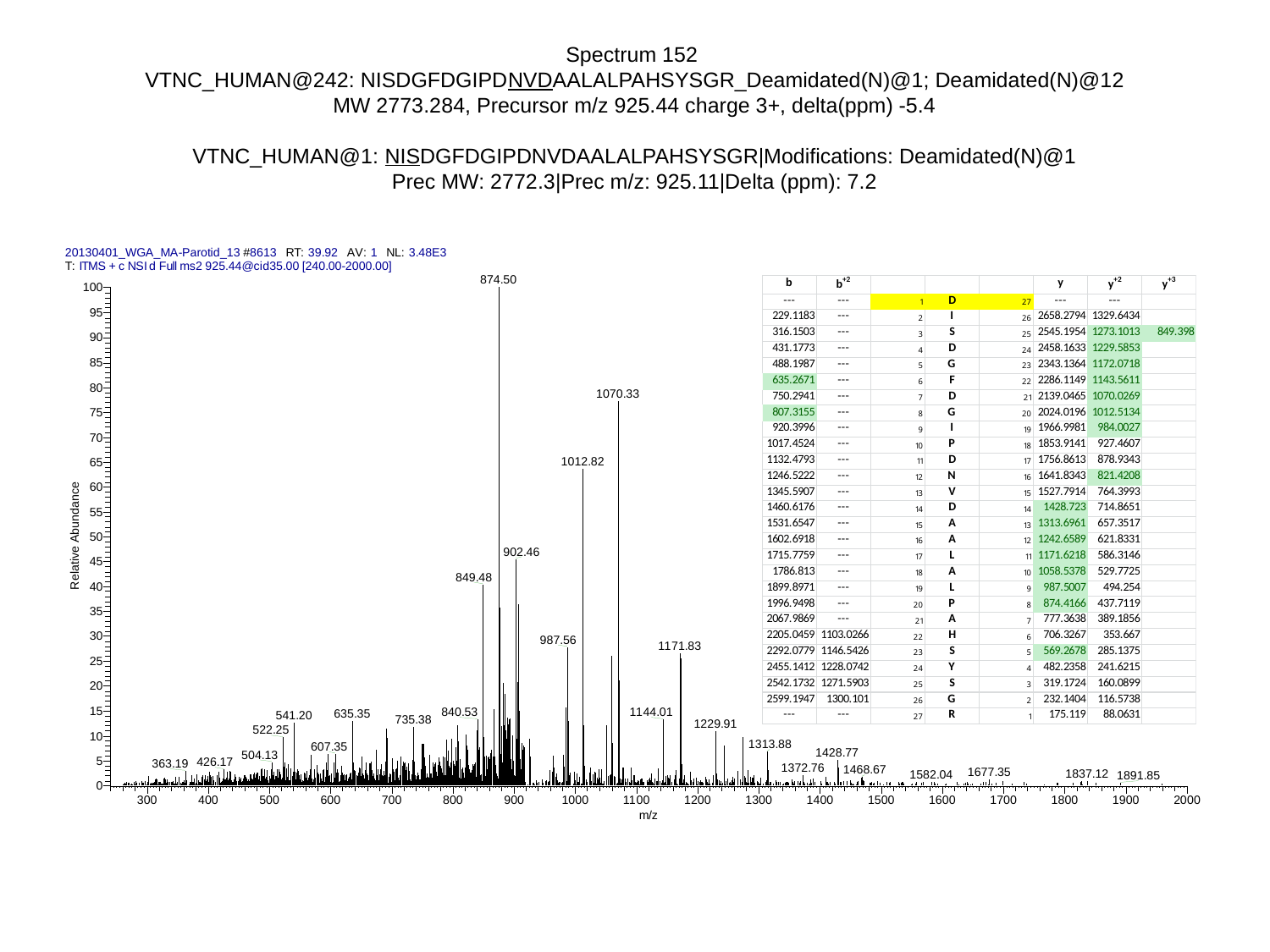

# Spectrum 152 VTNC_HUMAN@242: NISDGFDGIPDNVDAALALPAHSYSGR_Deamidated(N)@1; Deamidated(N)@12 MW 2773.284, Precursor m/z 925.44 charge 3+, delta(ppm) -5.4 VTNC_HUMAN@1: NISDGFDGIPDNVDAALALPAHSYSGR|Modifications: Deamidated(N)@1 Prec MW: 2772.3|Prec m/z: 925.11|Delta (ppm): 7.2

## Slide 178
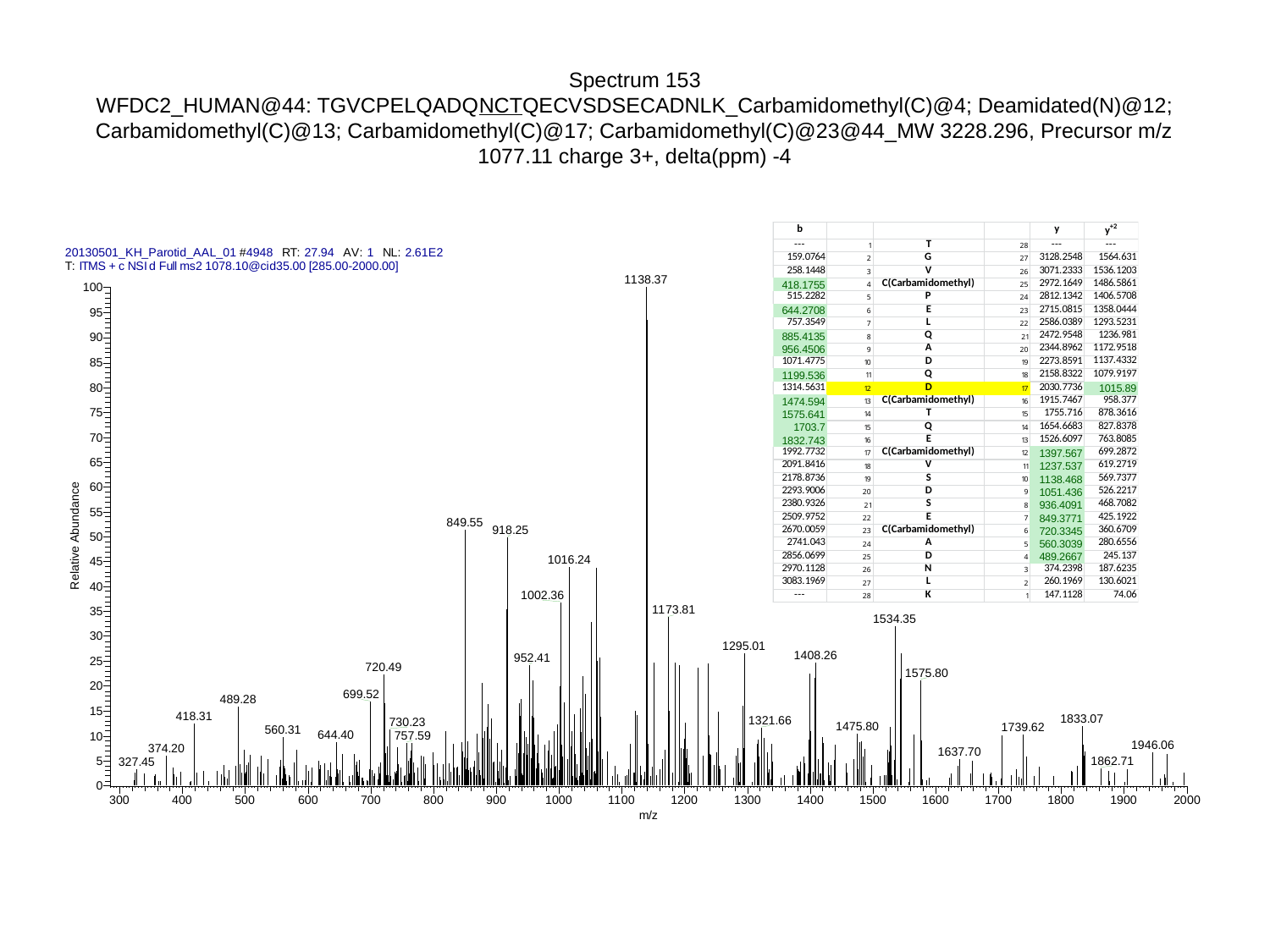

# Spectrum 153 WFDC2_HUMAN@44: TGVCPELQADQNCTQECVSDSECADNLK_Carbamidomethyl(C)@4; Deamidated(N)@12; Carbamidomethyl(C)@13; Carbamidomethyl(C)@17; Carbamidomethyl(C)@23@44_MW 3228.296, Precursor m/z 1077.11 charge 3+, delta(ppm) -4

## Slide 179
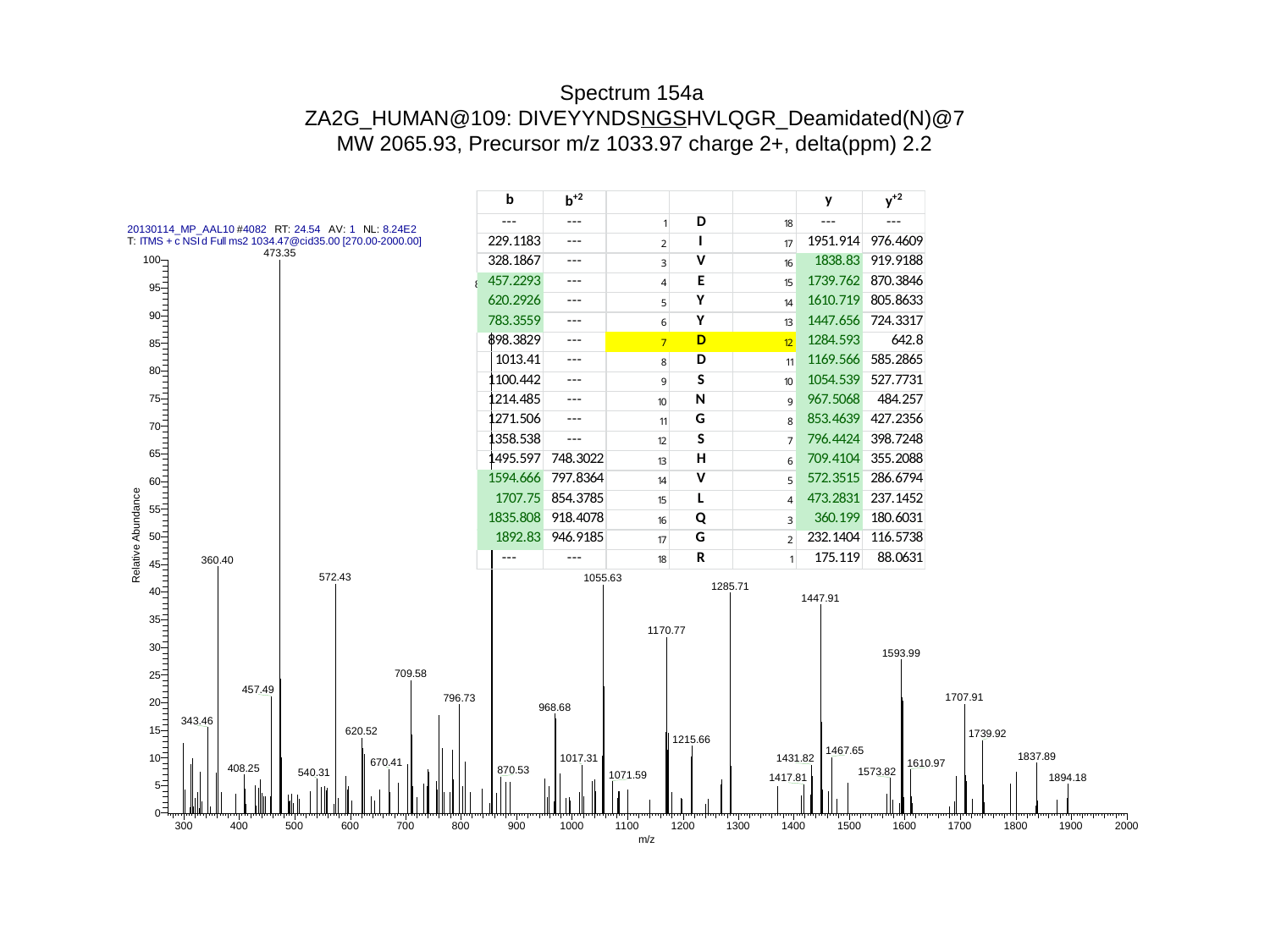

# Spectrum 154a ZA2G_HUMAN@109: DIVEYYNDSNGSHVLQGR_Deamidated(N)@7 MW 2065.93, Precursor m/z 1033.97 charge 2+, delta(ppm) 2.2

## Slide 180
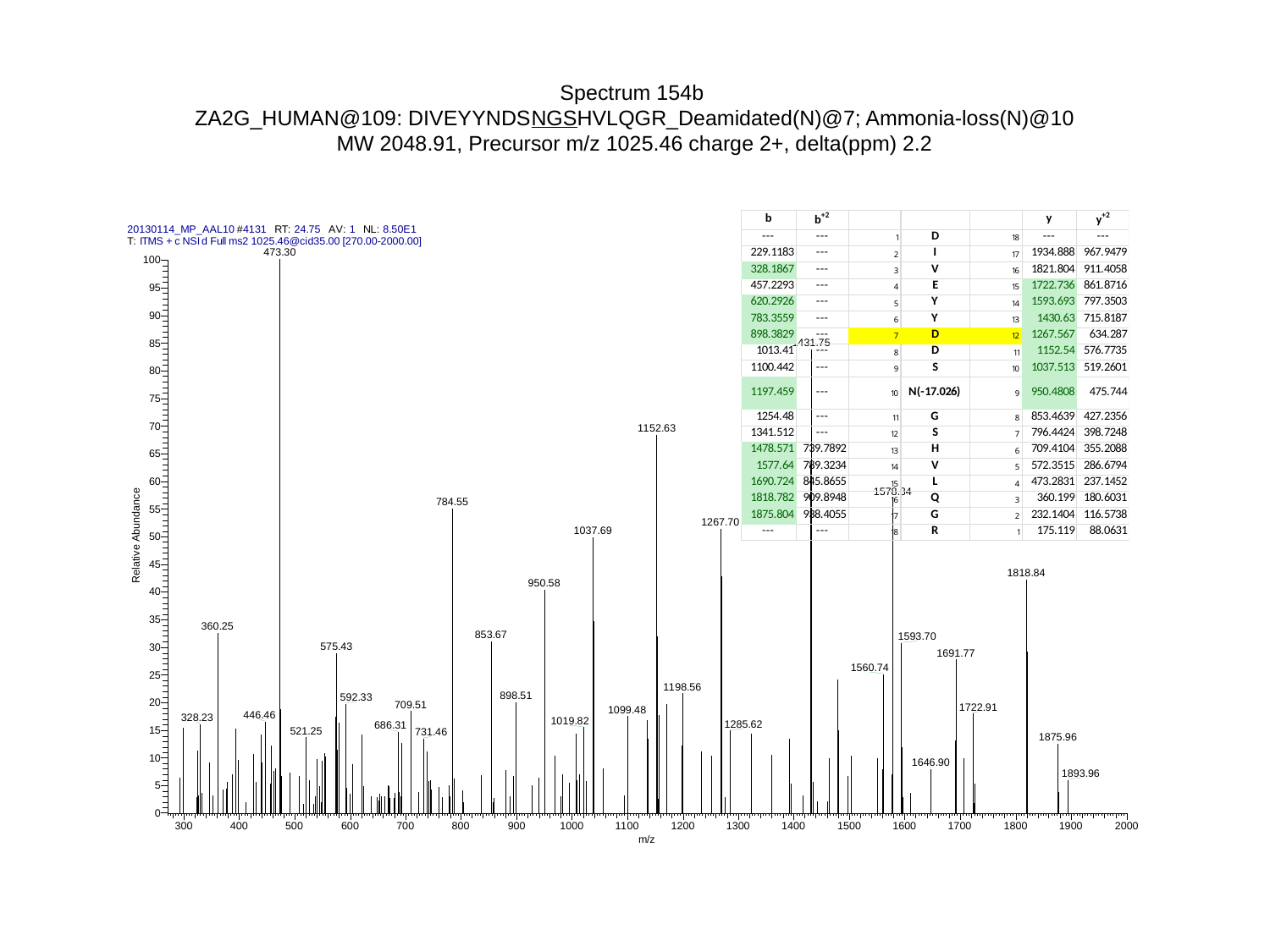

# Spectrum 154b ZA2G_HUMAN@109: DIVEYYNDSNGSHVLQGR_Deamidated(N)@7; Ammonia-loss(N)@10 MW 2048.91, Precursor m/z 1025.46 charge 2+, delta(ppm) 2.2

## Slide 181
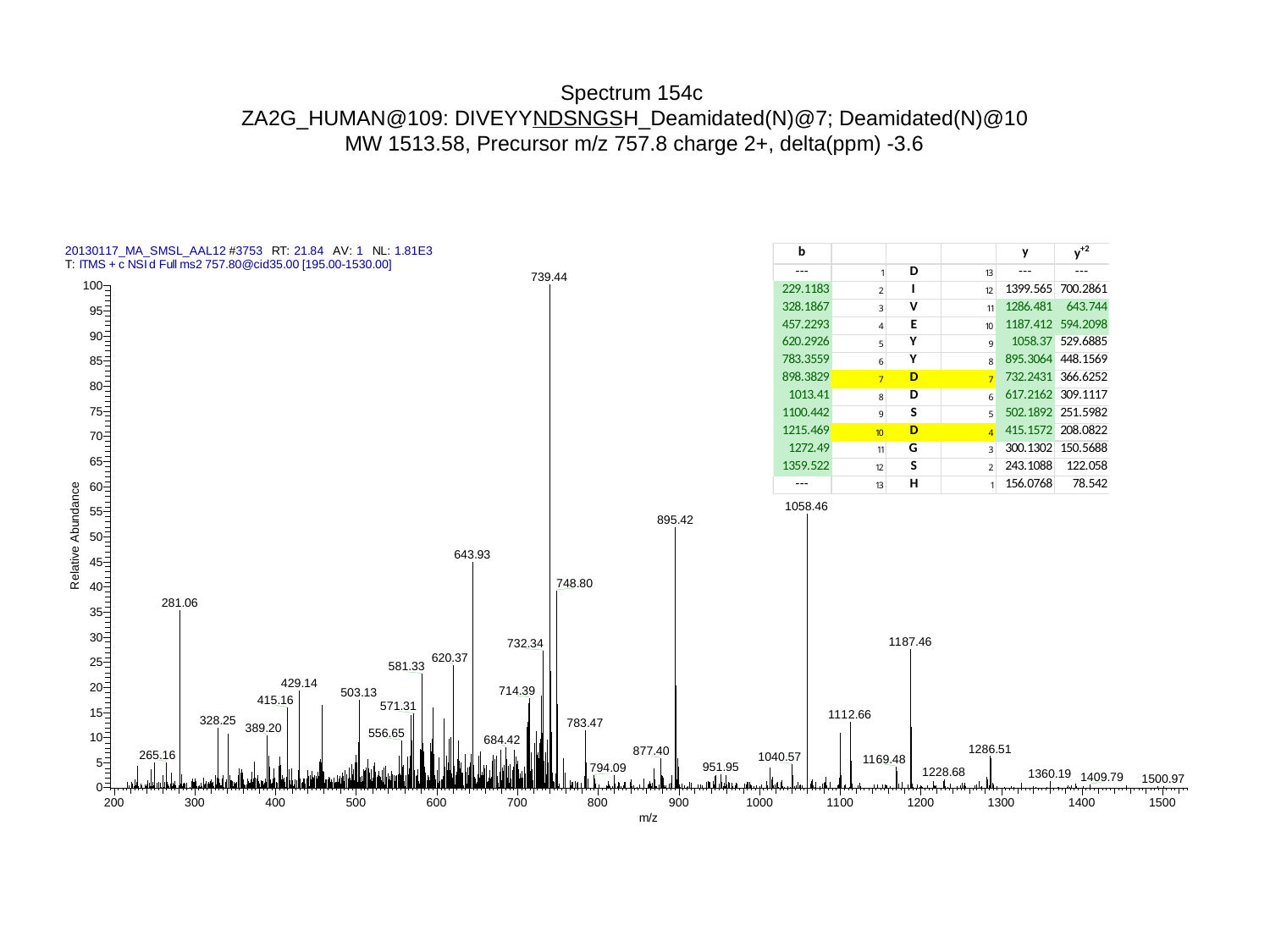

# Spectrum 154c ZA2G_HUMAN@109: DIVEYYNDSNGSH_Deamidated(N)@7; Deamidated(N)@10 MW 1513.58, Precursor m/z 757.8 charge 2+, delta(ppm) -3.6

## Slide 182
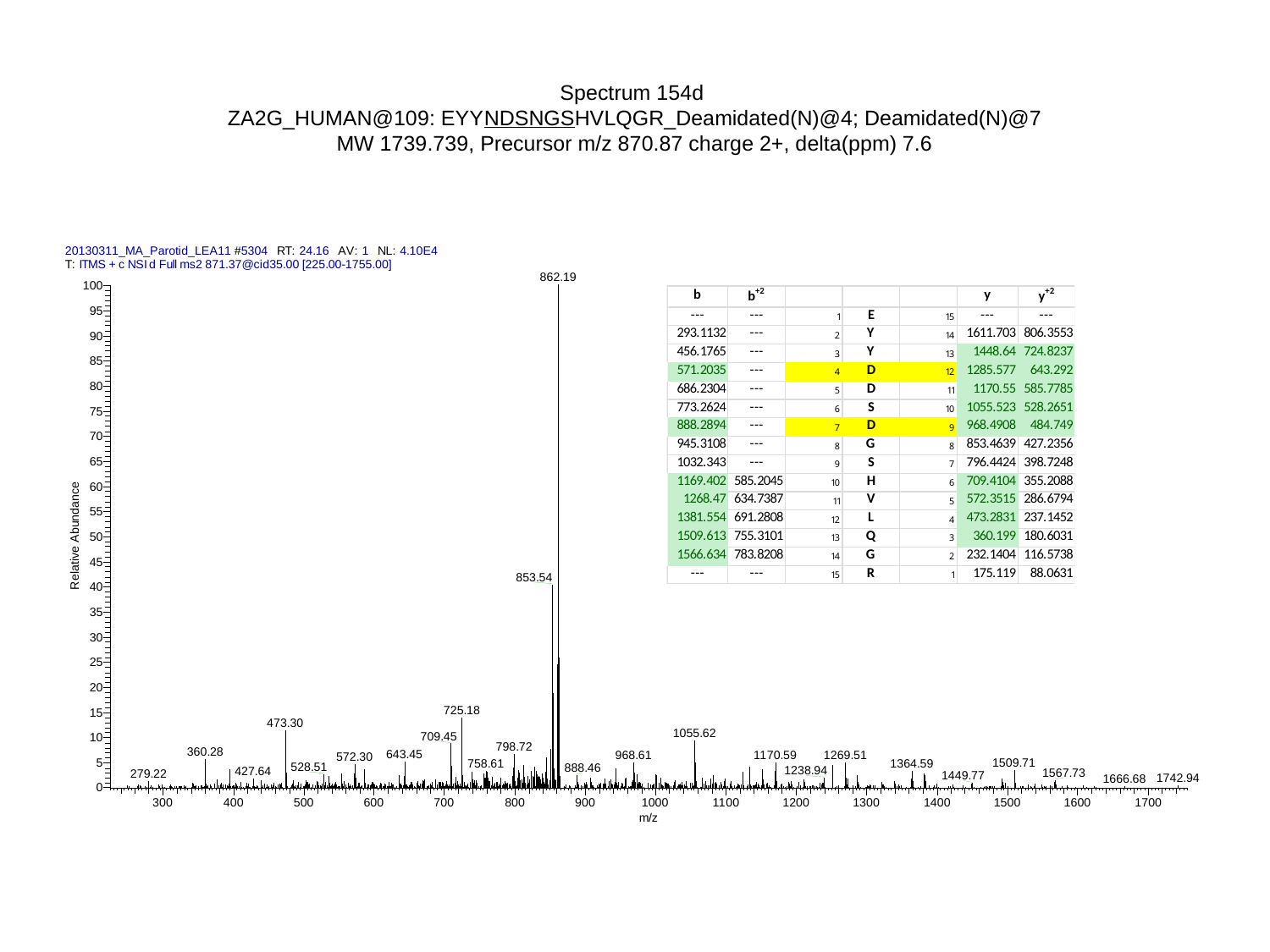

# Spectrum 154d ZA2G_HUMAN@109: EYYNDSNGSHVLQGR_Deamidated(N)@4; Deamidated(N)@7 MW 1739.739, Precursor m/z 870.87 charge 2+, delta(ppm) 7.6

## Slide 183
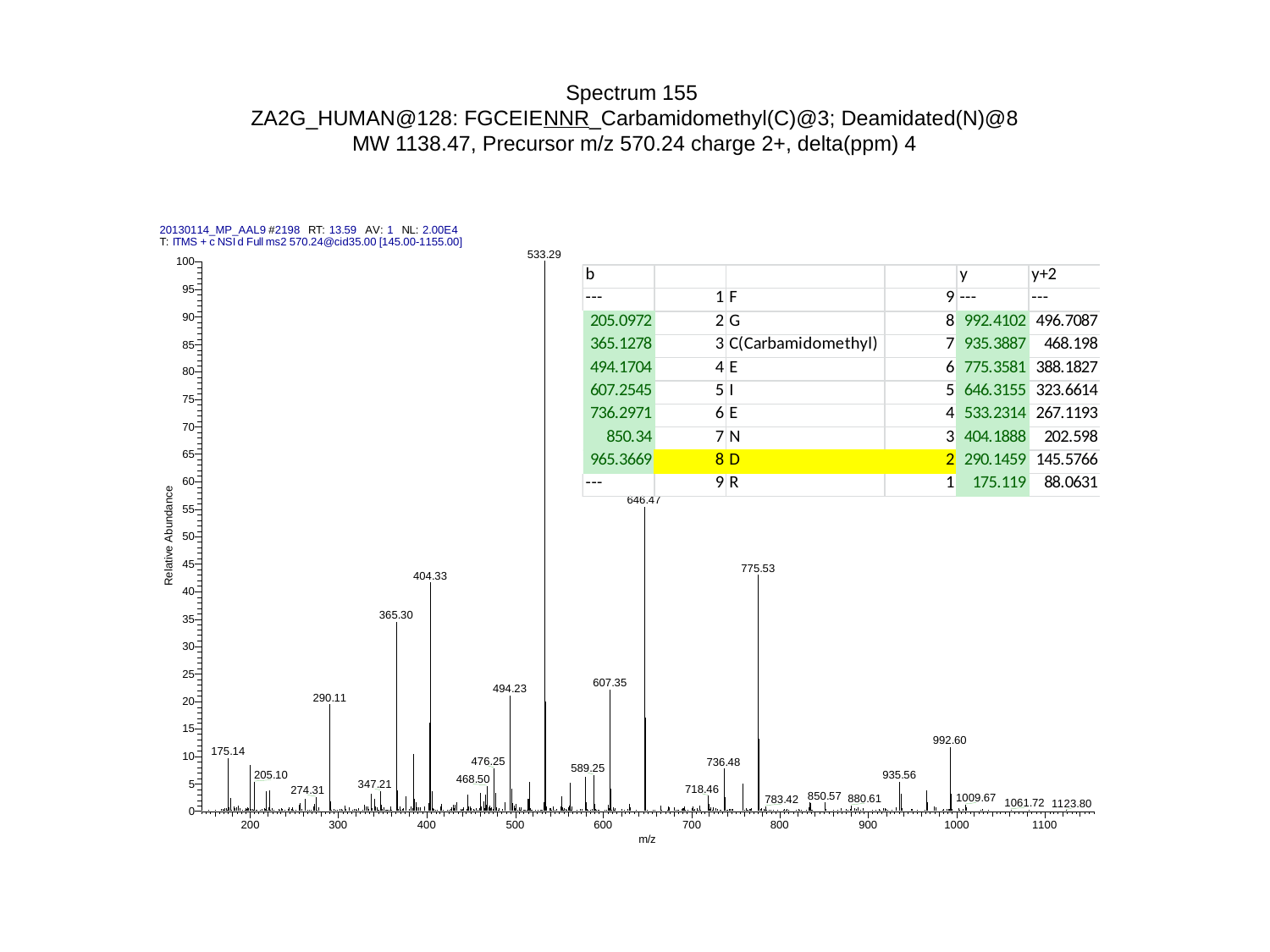

# Spectrum 155 ZA2G_HUMAN@128: FGCEIENNR_Carbamidomethyl(C)@3; Deamidated(N)@8 MW 1138.47, Precursor m/z 570.24 charge 2+, delta(ppm) 4

## Slide 184
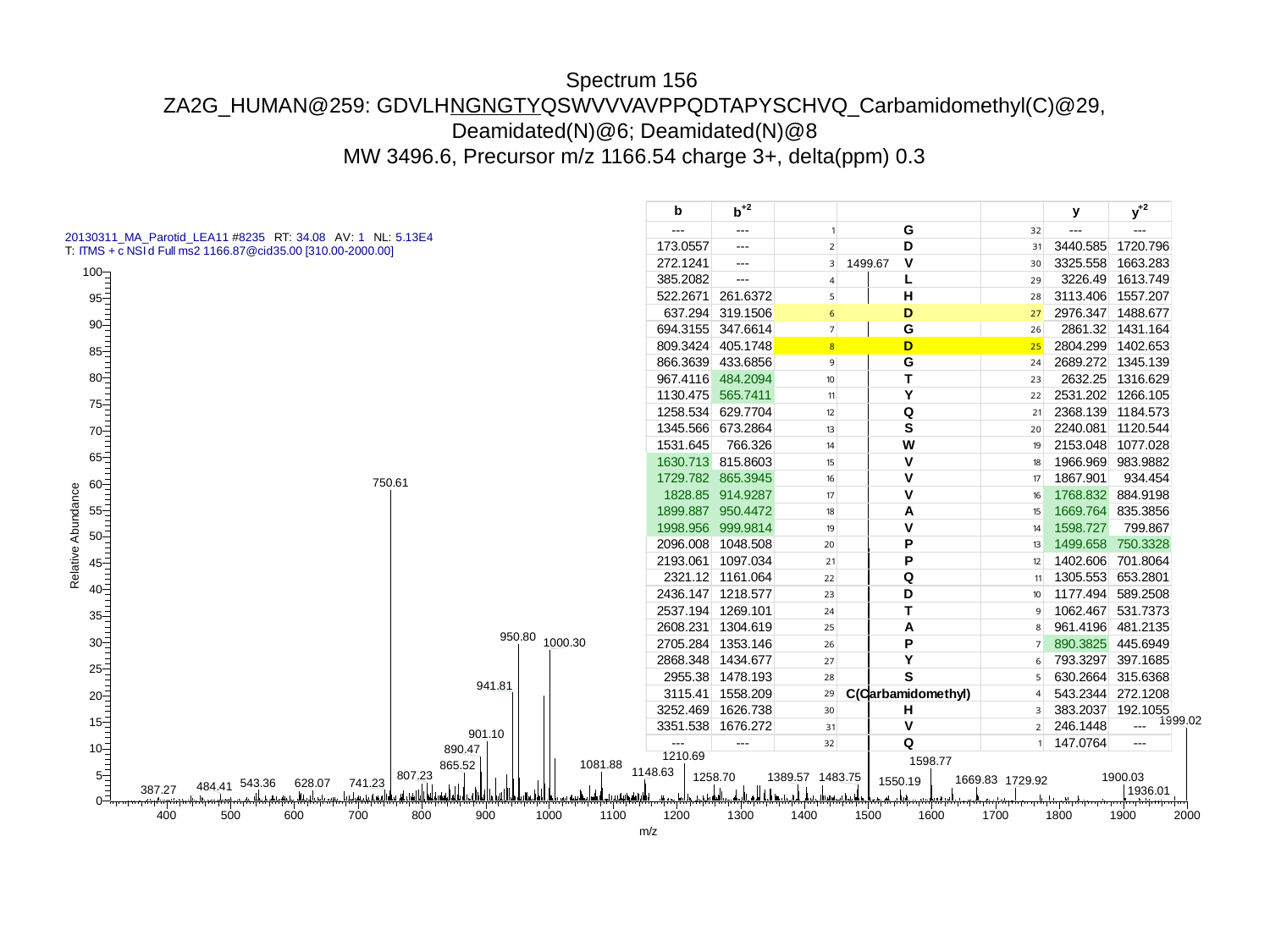

# Spectrum 156 ZA2G_HUMAN@259: GDVLHNGNGTYQSWVVVAVPPQDTAPYSCHVQ_Carbamidomethyl(C)@29, Deamidated(N)@6; Deamidated(N)@8 MW 3496.6, Precursor m/z 1166.54 charge 3+, delta(ppm) 0.3
