# Supplementary material for: Mass spectrometry-based analyses showing the effects of secretor and blood group status on salivary N-glycosylation
Source: Clin Proteomics. 2015 Dec 30;12:29. doi: 10.1186/s12014-015-9100-y (PMC4696288; doi:10.1186/s12014-015-9100-y)
Supplement: Supplementary file 4 — 10.1186/s12014-015-9100-y Spectral count distribution for N-glyco- and non-glycopeptides in the secretor vs. nonsecretor samples. Spectral counting suggested that higher copy numbers of N-glycosites were detected in the secretor vs. the nonsecretor sample (Figure S3A). This was despite the fact that the spectral count distribution of non-glycosylated peptides was virtually identical for both samples types (Figure S3B). [file 12014_2015_9100_MOESM4_ESM.pptx]

## Slide 1
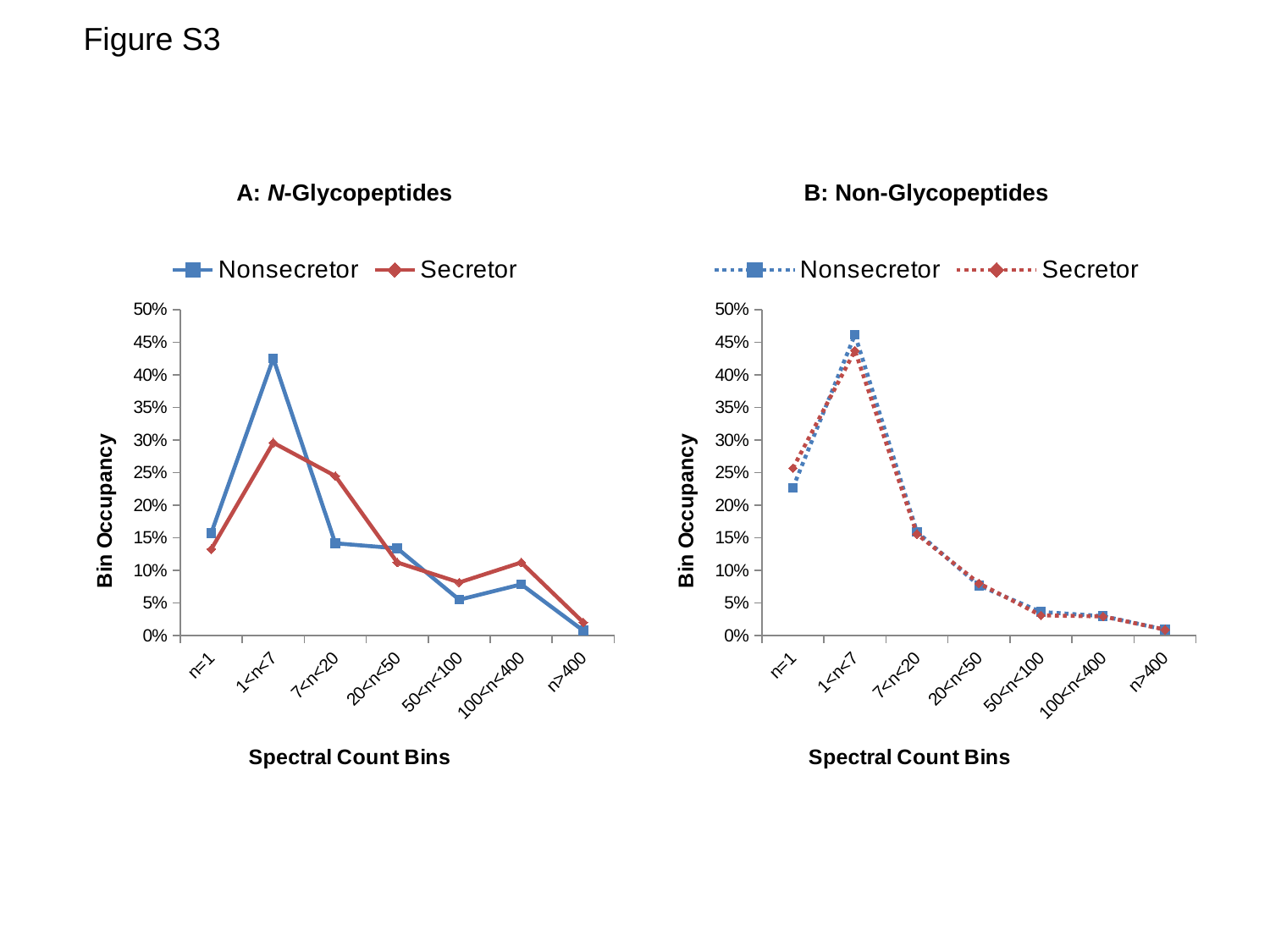

Figure S3
A: N-Glycopeptides
B: Non-Glycopeptides
### Chart
| Category | Nonsecretor | Secretor |
|---|---|---|
| n=1 | 0.15748031496063 | 0.13265306122449 |
| 1<n<7 | 0.425196850393701 | 0.295918367346939 |
| 7<n<20 | 0.141732283464567 | 0.244897959183673 |
| 20<n<50 | 0.133858267716535 | 0.112244897959184 |
| 50<n<100 | 0.0551181102362205 | 0.0816326530612245 |
| 100<n<400 | 0.078740157480315 | 0.112244897959184 |
| n>400 | 0.00787401574803149 | 0.0204081632653061 |
### Chart
| Category | Nonsecretor | Secretor |
|---|---|---|
| n=1 | 0.226832641770401 | 0.256830601092896 |
| 1<n<7 | 0.461502996772706 | 0.437158469945355 |
| 7<n<20 | 0.159059474412172 | 0.155737704918033 |
| 20<n<50 | 0.0769940064545874 | 0.080327868852459 |
| 50<n<100 | 0.0364223144306132 | 0.0311475409836066 |
| 100<n<400 | 0.0299677270631627 | 0.0295081967213115 |
| n>400 | 0.00922083909635777 | 0.0092896174863388 |
