# Supplementary material for: Mass spectrometry-based analyses showing the effects of secretor and blood group status on salivary N-glycosylation
Source: Clin Proteomics. 2015 Dec 30;12:29. doi: 10.1186/s12014-015-9100-y (PMC4696288; doi:10.1186/s12014-015-9100-y)
Supplement: Supplementary file 5 — 10.1186/s12014-015-9100-y N-Glycosite distributions binned according to spectral counts. (Left Panel) Similar distributions of donor-common N-glycosites were observed across all bins regardless of secretor status. (Right Panel) Lower abundance N-glycosites were unique to a particular donor. [file 12014_2015_9100_MOESM5_ESM.pptx]

## Slide 1
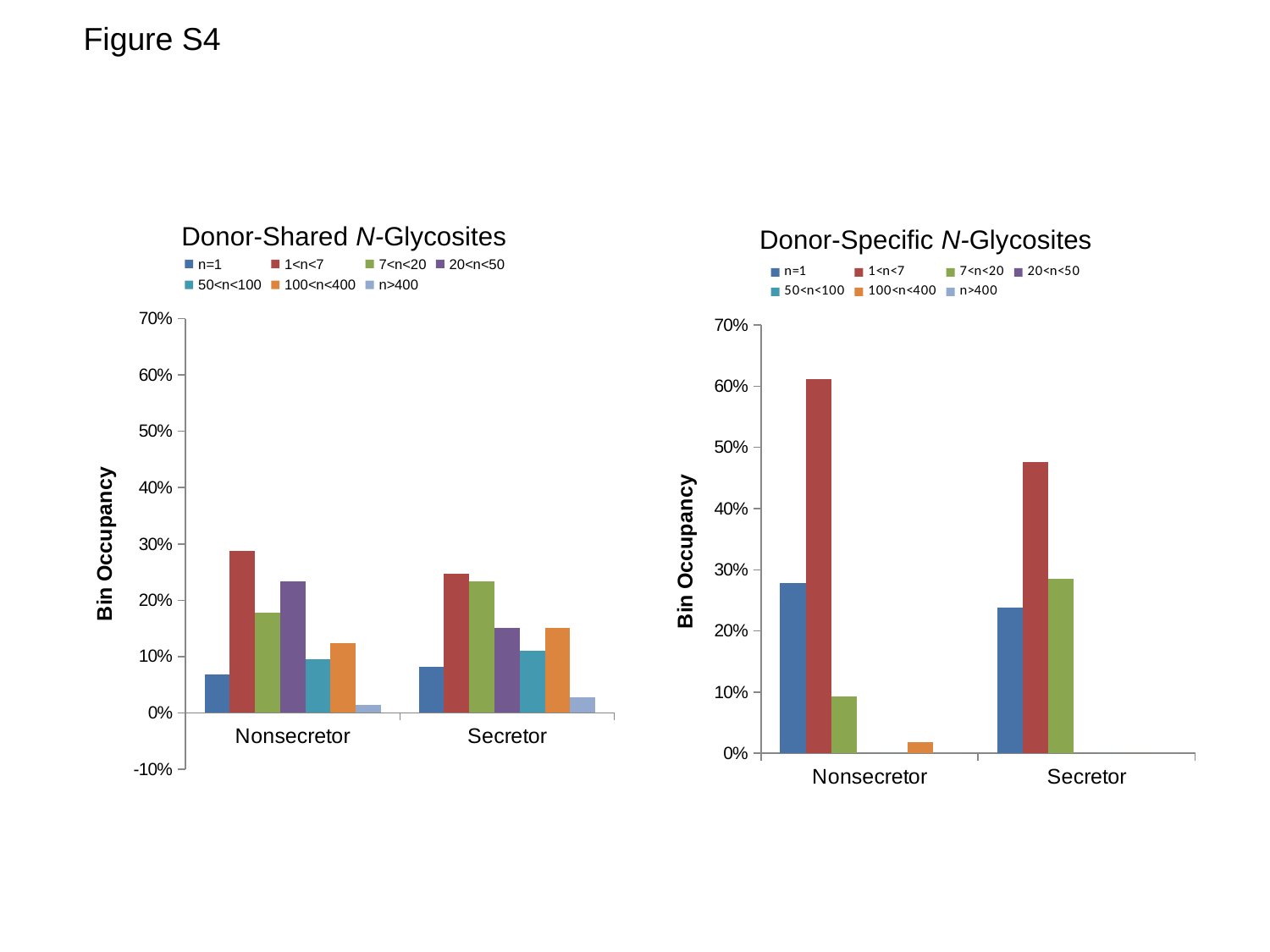

Figure S4
Donor-Shared N-Glycosites
Donor-Specific N-Glycosites
### Chart
| Category | n=1 | 1<n<7 | 7<n<20 | 20<n<50 | 50<n<100 | 100<n<400 | n>400 |
|---|---|---|---|---|---|---|---|
| Nonsecretor | 0.0684931506849315 | 0.287671232876712 | 0.178082191780822 | 0.232876712328767 | 0.0958904109589041 | 0.123287671232877 | 0.0136986301369863 |
| Secretor | 0.0821917808219178 | 0.246575342465753 | 0.232876712328767 | 0.150684931506849 | 0.10958904109589 | 0.150684931506849 | 0.0273972602739726 |
### Chart
| Category | n=1 | 1<n<7 | 7<n<20 | 20<n<50 | 50<n<100 | 100<n<400 | n>400 |
|---|---|---|---|---|---|---|---|
| Nonsecretor | 0.277777777777778 | 0.611111111111111 | 0.0925925925925926 | 0.0 | 0.0 | 0.0185185185185185 | 0.0 |
| Secretor | 0.238095238095238 | 0.476190476190476 | 0.285714285714286 | 0.0 | 0.0 | 0.0 | 0.0 |
