# Supplementary material for: Mass spectrometry-based analyses showing the effects of secretor and blood group status on salivary N-glycosylation
Source: Clin Proteomics. 2015 Dec 30;12:29. doi: 10.1186/s12014-015-9100-y (PMC4696288; doi:10.1186/s12014-015-9100-y)
Supplement: Supplementary file 6 — 10.1186/s12014-015-9100-y Efficiency of N-glycopeptide capture by lectin, saliva type and secretor status. Sites that were unique to a particular sample-lectin combination are shown in red and those that were shared among sample-lectin combinations are depicted in blue. (Left Panel) the number of N-glycosites and (Right Panel) the relative abundances in terms of spectral counts. Green stars denote >2 SD from the mean. In terms of unique N-glycosites detected, AAL captured the largest number in parotid saliva of the nonsecretor. In terms of relative abundances, JAC captured the most copies of unique N-glycosites from SMSL saliva. [file 12014_2015_9100_MOESM6_ESM.pptx]

## Slide 1
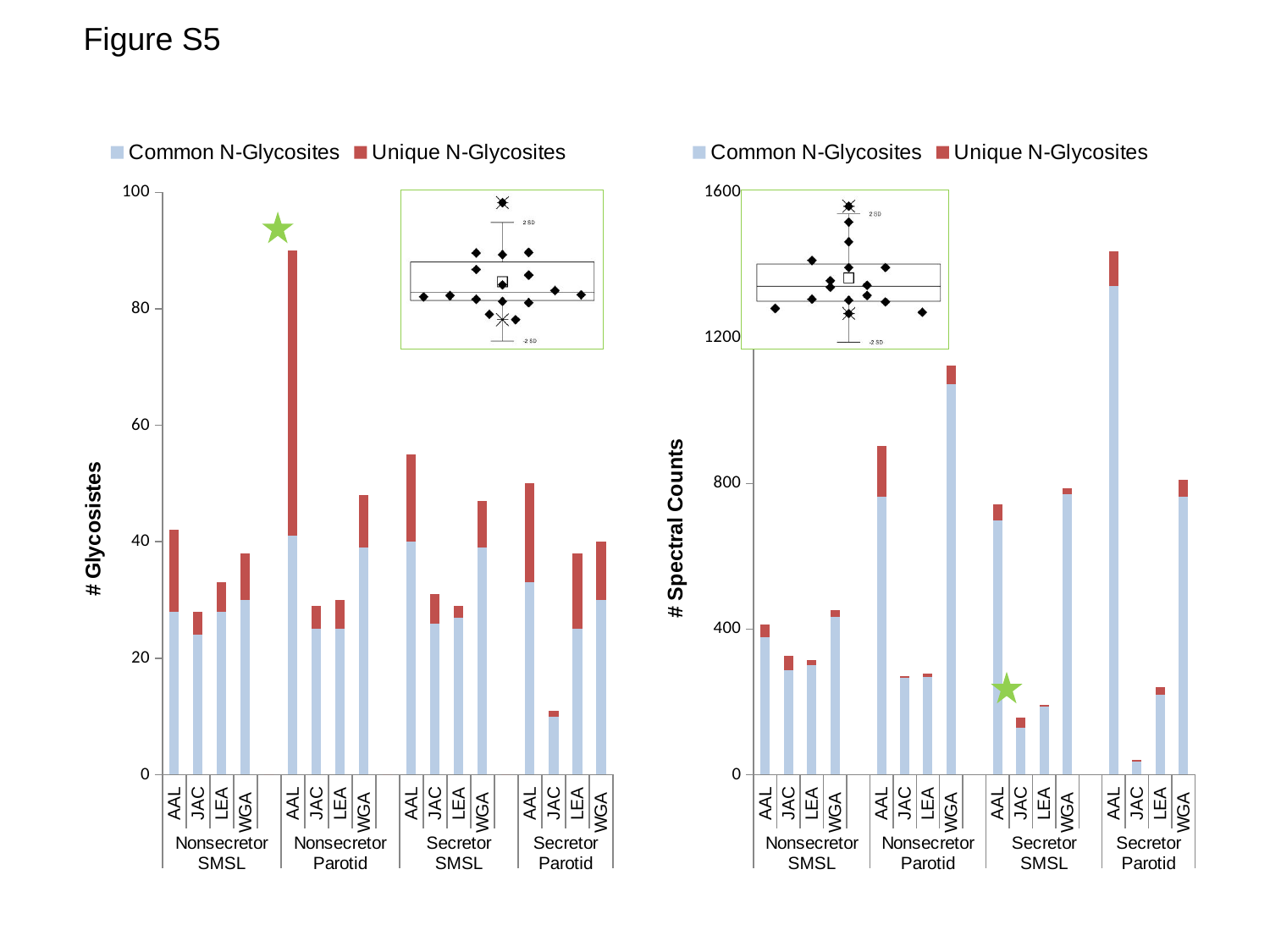

Figure S5
### Chart
| Category | Common N-Glycosites | Unique N-Glycosites |
|---|---|---|
| AAL | 28.0 | 14.0 |
| JAC | 24.0 | 4.0 |
| LEA | 28.0 | 5.0 |
| WGA | 30.0 | 8.0 |
| | None | 0.0 |
| AAL | 41.0 | 49.0 |
| JAC | 25.0 | 4.0 |
| LEA | 25.0 | 5.0 |
| WGA | 39.0 | 9.0 |
| | None | 0.0 |
| AAL | 40.0 | 15.0 |
| JAC | 26.0 | 5.0 |
| LEA | 27.0 | 2.0 |
| WGA | 39.0 | 8.0 |
| | None | 0.0 |
| AAL | 33.0 | 17.0 |
| JAC | 10.0 | 1.0 |
| LEA | 25.0 | 13.0 |
| WGA | 30.0 | 10.0 |
### Chart
| Category | Common N-Glycosites | Unique N-Glycosites |
|---|---|---|
| AAL | 377.0 | 36.0 |
| JAC | 286.0 | 41.0 |
| LEA | 302.0 | 12.0 |
| WGA | 433.0 | 18.0 |
| | None | None |
| AAL | 764.0 | 140.0 |
| JAC | 265.0 | 5.0 |
| LEA | 268.0 | 10.0 |
| WGA | 1073.0 | 51.0 |
| | None | None |
| AAL | 698.0 | 45.0 |
| JAC | 129.0 | 28.0 |
| LEA | 186.0 | 5.0 |
| WGA | 770.0 | 16.0 |
| | None | None |
| AAL | 1342.0 | 97.0 |
| JAC | 37.0 | 4.0 |
| LEA | 220.0 | 21.0 |
| WGA | 764.0 | 47.0 |
